# Gold(I)-catalyzed intramolecular cyclization/intermolecular cycloaddition cascade as a fast track to polycarbocycles and mechanistic insights

**DOI:** 10.1038/s41467-021-21335-9

**Published:** 2021-02-19

**Authors:** Cheng Zhang, Kemiao Hong, Chao Pei, Su Zhou, Wenhao Hu, A. Stephen K. Hashmi, Xinfang Xu

**Affiliations:** 1 Guangdong Provincial Key Laboratory of Chiral Molecule and Drug Discovery, School of Pharmaceutical Sciences, Sun Yat-sen University, Guangzhou, 510006 China; 2 Organisch-Chemisches Institut, Heidelberg University, 69120 Heidelberg, Germany

**Keywords:** Catalytic mechanisms, Optical materials, Reaction mechanisms, Synthetic chemistry methodology

## Abstract

Metal carbene is an active synthetic intermediate, which has shown versatile applications in synthetic chemistry. Although a variety of catalytic methods have been disclosed for the generation of carbene species from different precursors, there is an increasing demand for the development of efficient and practical approaches for the in-situ formation of metal carbene intermediates with structural diversity and unrevealed reactivity. Herein we report a gold-catalyzed cascade protocol for the assembly of polycarbocyclic frameworks in high yields under mild reaction conditions. Mechanistic studies indicate that the unique β-aryl gold-carbene species, generated via gold-promoted 6-*endo*-*dig* diazo-yne cyclization, is the key intermediate in this reaction, followed by a [4 + 2]-cycloaddition with external alkenes. In comparison to the well-documented metal carbene cycloadditions, this carbene intermediate serves as a 4-C synthon in a cycloaddition reaction. A variety of elusive π-conjugated polycyclic hydrocarbons (CPHs) with multiple substituents are readily accessible from the initially generated products by a mild oxidation procedure.

## Abstract (web summary)

Metal carbene is usually employed as a 1-carbon synthon or 3-carbon synthon in a variety of cycloaddition reactions. Here, the authors report a gold-catalyzed cascade protocol for the assembly of polycarbocyclic frameworks via a β-aryl gold-carbene intermediate which reacts as a 4-carbon synthon with alkenes in [4 + 2]-cycloadditions.

## Introduction

**Fig. 1:**
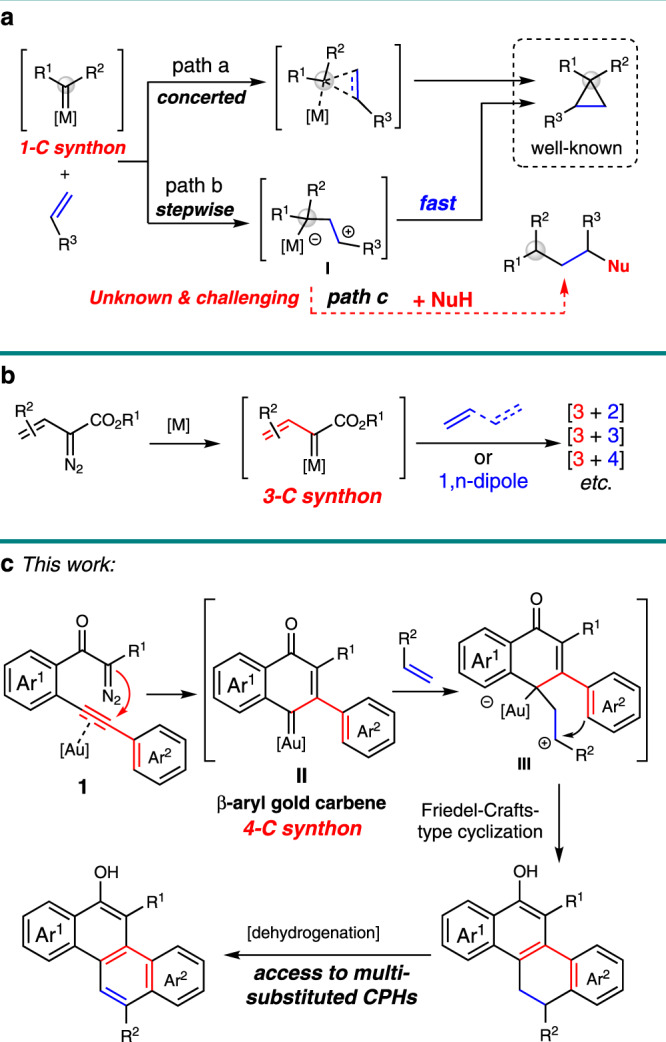
Catalytic metal carbene cycloadditions. **a** Concerted and stepwise cyclopropanation. **b** [3 + *n*]-Cycloadditions of vinyl/enol metal carbenes. **c** This work: gold(I)-catalyzed 6-*endo-dig* carbocyclization and stepwise [4+2] cycloaddition reaction.

Reactive metal carbene species participate in a broad range of applications for the effective formation of C–C and C–heteroatom bonds in synthetic organic chemistry^1–4^. The versatile reactivity of this species is mainly dependent on the choice of catalysts and the substituent(s) proximal to the carbene center^5,6^. The effect of these two variables can be quite pronounced, and the synthetic transformations could be expected by switching either of these parameters. For example, concerted and stepwise reaction pathways have been disclosed in the cyclopropanation reaction, depending on the type of metal catalysts that were used in these transformations with corresponding carbene precursors (Fig. 1a, path a vs path b)^7–9^. Moreover, various [4 + 1]-cycloaddition reactions of carbene species with α,β-unsaturated carbonyl compounds^10^, in situ generated *o*-QMs^11,12^, 1,3-dienes^13^, or other functionalized alkenes^14^ have been realized through different reaction pathways in the presence of corresponding metal catalysts. On the other hand, the vinyl metal carbene, which possesses two electrophilic sites, could function as 1- or 3-carbon building blocks. Davies et al. have demonstrated that addition^15^ or C–H insertion^16^ of this intermediate with enol ethers are the dominating transformations in the presence of dirhodium catalysts. But a [3 + 2]-cycloaddition could be enabled in the presence of gold catalyst with identical starting materials^17^. Meanwhile, a variety of [3 + *n*]-cycloadditions of vinyl/enol metal carbene species with corresponding dipolarophiles have been disclosed independently by Doyle^18^, Davies^19^, and Yoo^20^ (Fig. 1b). Despite these significant achievements, the carbene precursors in these cycloadditions have been severely limited to α-vinyl and α-silyl enol diazoacetates, and very rare example of using carbene species as a 4-carbon synthon has been disclosed^21^. Thus, the exploration of effective catalytic approaches for the access to different types of carbene intermediates with readily available materials is highly desirable, which would substantially broaden the substrate scope for the diversity synthesis, and more importantly, enabling methods for the practical synthesis.

Recently, the gold(I) complexes, which are versatile and selective catalysts for alkyne activation due to their strong Lewis acidity and potential to stabilize cationic reaction intermediates, have been employed in a plethora of synthetic transformations, allowing rapid and efficient assembly of structurally complex molecules^22–27^. In this regard, the three-center four-electron σ-bond model for the fundamental description of the gold-carbene intermediate has been proposed by Toste and Goddard in 2009^28^, however, the electronic nature of these gold intermediates, carbene or carbocation^29,30^, is still under exploration^31–34^. Beyond the terminological point^35^, the catalytic cycloadditions of gold carbene with olefins have attracted much attention. Cyclopropanation of olefins with a variety of carbene precursors, including diazo compounds, 1,*n*-enynes, yne-enones, propargyl esters, cyclopropenes, 1,3,5-cycloheptatrienes, and alkynes via oxidation/nitrene transfer processes, is the main focus in this area^36^. Gold-catalyzed [4 + 1]-^37^ and [3 + 2]-cycloadditions^38,39^ have been disclosed by Echavarren with 1,3,5-cycloheptatriene as the carbene precursor. Meanwhile, the gold-catalyzed formal [4 + 2]-cyclization of enynes with tethered alkene via cyclopropyl metal carbene intermediate has been studied by the same group^40^. Inspired by these advances and our recent study on gold-catalyzed diazo-yne carbocyclization^41–43^, we envisioned that different reactivity could be disclosed with these unique types of in situ generated carbene intermediates, which could not be formed through other approaches or precursors. For example, if an asynchronous reaction pathway is dominating in the reaction of carbene intermediate with alkenes (Fig. 1a, path b), then, transformations beyond the cyclopropanation might become possible by trapping the carbocation intermediate **I** (Fig. 1a, path c). However, the second C–C bond formation occurs through a rather low energy barrier in the stepwise cyclopropanation reaction, thus, the interception of the carbocation **I** with an external nucleophile (NuH) is still a challenge. Herein, we describe our recent results in this direction, a cycloaddition reaction of the cross-conjugated β-aryl gold-carbene **II** serving as a 4-C synthon, which generated in situ through a selective 6-*endo-dig* diazo-yne carbocyclization^44–47^. This reaction features an asynchronous process^48^: the gold-carbene intermediate reacts with an olefin to form the carbocation species **III**, which could be successfully intercepted by the tethering aryl group rather than the cyclopropanation, leading to a general access for the assembly of polycarbocyclic frameworks in high to excellent yields (Fig. 1c). Moreover, these products could be readily transformed into π-conjugated polycyclic hydrocarbons (CPHs) under mild oxidation procedure.

## Results

### Reaction optimization

Initially, the alkyne-containing 1,3-dicarbonyl diazo compound **1a** and styrene **2a** were chosen as the model substrates to optimize the reaction conditions (Table 1). A variety of metal catalysts, such as Rh^I^-, Rh^II^-, Cu^II^-, Pd^0^-, and Ag^I^-complexes were examined in dichloroethane (DCE) at different temperatures, which all led to the tricyclic product **3′** in high to excellent yields rather than the desired tetracyclic product **3** (entries 1–5). Given the fact that the formation of **3′** with these catalysts should go through a carbene/alkyne metathesis process (CAM)^49–58^, the gold-complexes, which have shown unique ability to selectively activate alkyne species with the pendant diazo group served as a latent functionality^41–43,59^, were then evaluated. Due to the competition between ligand and carbene for the contribution of electron density of gold center, the ligands of the gold catalysts have a significant influence on the bonding and reactivity of corresponding gold-complex intermediates^28–35^, and we have observed these dramatic influences in the outcome of the following optimization. The gold catalysts with trialkylphosphines as the ligands could produce mainly the polycarbocyclic product **3** in moderate conversions (entries 6–8, 29–36% yields), whereas, the triarylphosphines showed relatively lower reactivity (27–39% conversions), preferring to form the intramolecular cyclization product **3′** (entries 9–11). Further investigation of phosphine ligands with structural and electronic diversity implied that ligands bearing electron-donating substituents (entries 12 vs 13) and with appropriate steric hindrance (entry 14 vs entries 12, 16, and 17) gave better results, affording **3** in 90% NMR yield when JohnPhos (**L3**) was used as the ligand (entry 14, 84% isolated yield). A comparably good result was obtained by switching the counter anion of gold catalyst from SbF_6_^−^ to NTf_2_^−^ (entry 15). Based on these results, we set out to explore a statistical regression approach to interpretation and prediction of ligand effects. The calculated Au–Cl bond distance, which has been disclosed by Fey and co-workers^60^, might provide such a platform to quantify the steric and electronic properties of these ligands^61,62^. Our optimization results have shown good correlation with the calculated parameters of the Au–Cl bond distance of gold-complexes with corresponding ligands (see Supplementary Fig. 1 for details). Moreover, these results bring us to predict that electron-donating substituents with moderate steric hindrance on the phosphine ligand might further improve the yield. Thus, the ligand (Me_2_N)_3_P, which is similar to triisopropylphosphine (entry 8), but is much more flexible due to the additional freedom of nitrogen inversion and the three amino groups offer complementary donor functions^63,64^, was introduced. Gratifyingly, this ligand proved to the most effective one, delivering the desired product **3** in 89% isolated yield (entry 18).

**Table 1:** Optimization of the conditions.


Entry^a^	Cat.	Conv. (%)	Yield 3/3′ (%)^b^
1	Rh(COD)_2_BF_4_	>95	<5/89
2	Rh_2_(OAc)_4_	>95	<5/75
3	Cu(hfacac)_2_·H_2_O	>95	<5/95
4^c^	Pd_2_(dba)_3_·CHCl_3_	>95	<5/87
5^c^	AgSbF_6_	>95	<5/91
6	Cy_3_PAuCl + AgSbF_6_	63	36/<5
7	*t*Bu_3_PAuCl + AgSbF_6_	41	29/<5
8	*i*Pr_3_PAuCl + AgSbF_6_	58	32/<5
9	PPh_3_PAuCl + AgSbF_6_	30	<5/21
10	(*p*-CF_3_C_6_H_4_)_3_PAuCl + AgSbF_6_	39	<5/26
11	(*p*-OMeC_6_H_4_)_3_PAuCl + AgSbF_6_	27	9/11
12	**L1**AuCl + AgSbF_6_	11	<5/<5
13	**L2**AuCl + AgSbF_6_	>95	58/<5
14	**L3**Au(CH_3_CN)SbF_6_	>95	90(84)^d^/<5
15	**L3**Au(CH_3_CN)NTf_2_	>95	87/<5
16	**L4**AuCl + AgSbF_6_	>95	52/<5
17	**L5**AuCl + AgSbF_6_	>95	41/<5
18	(Me_2_N)_3_PAuCl + AgSbF_6_	>95	92(89)^d^/<5


^a^Reaction conditions: to a solution of metal catalyst (5 mol%) in DCE (0.5 mL), we added the solution of **1a** (63.6 mg, 0.2 mmol) and styrene **2a** (35.0 μL, 0.3 mmol) in DCE (0.5 mL) at 60 °C. The reaction mixture was stirred for 6 h under these conditions.

^b^Yields were determined by proton NMR with mesitylene as internal standard.

^c^The reaction was conducted at 80 °C for 12 h.

^d^The results in the parentheses are isolated yields.

### Substrate scope

**Fig. 2:**
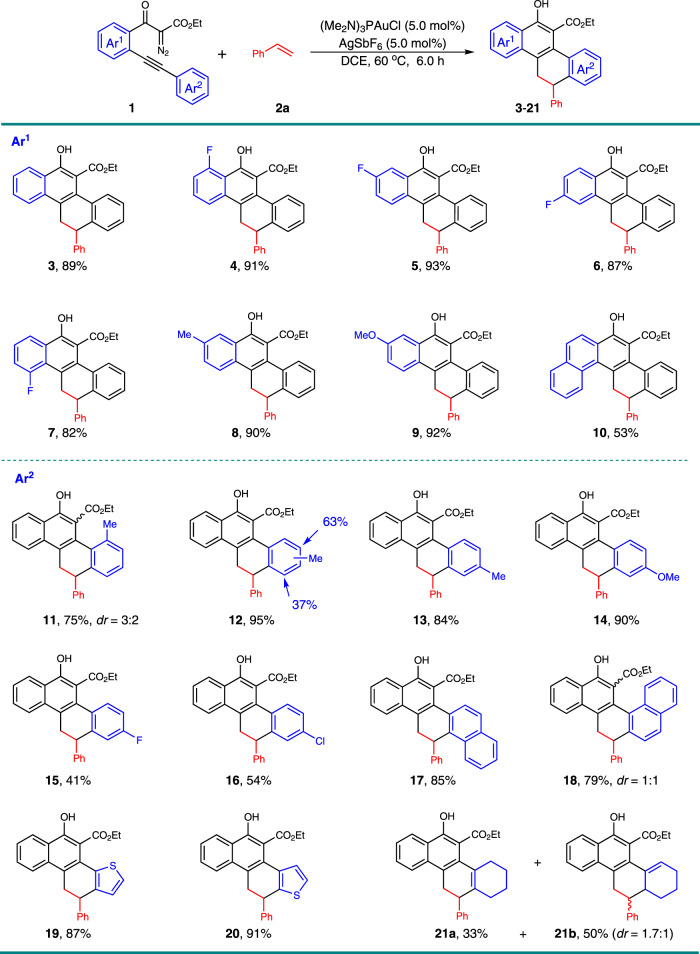
Scope with respect to the diazo compounds **1**. Reaction conditions: to a solution of (Me_2_N)_3_PAuCl (3.95 mg, 0.01 mmol), and AgSbF_6_ (3.4 mg, 0.01 mmol) in DCE (0.5 mL), we added a solution of **1** (0.2 mmol) and styrene **2a** (31.2 mg, 0.3 mmol) in DCE (0.5 mL) at 60 °C, then the reaction mixture was stirred for 6 h under these conditions. Isolated yields are listed.

Under the optimized reaction conditions, the scope of this gold-catalyzed [4 + 2]-cycloaddition with respect to the 1,3-dicarbonyl diazo compound **1** in combination with styrene **2a** was examined (Fig. 2). The substitutions on the aryl linkage (Ar^1^), including fluoro on the different positions (**4**–**7**), methyl (**8**), and methoxy (**9**) groups did not obviously affect the reactivity, and 82–93% isolated yield was obtained in these cases. The diazo compound with naphthyl group as the linkage provided the pentacyclic product **10** in 53% yield. Then, the nature of the alkyne terminus was investigated (Ar^2^). The steric hindrance resulting from the *ortho*- and *meta*-methyl substituents on the phenyl ring did not impact the reactivity a lot, delivering corresponding products **11** and **12** in 75% and 95% yield, respectively. Other diazo derivatives, containing different substituents on the *para*-position of the aryl ring, performed well under these conditions (**13**–**16**), although low yields were obtained in the halogen-substituted cases. This may due to the lower nucleophilicity of these aromatic rings. Naphthyl- and thienyl-alkynes reacted effectively under gold-catalysis, offering the corresponding polycyclic products all in high yields (**17**–**20**). The diastereomers of **18** resulted from the initially formed axial chirality in the diazo-yne cyclization step due to the hindered rotation of the naphthyl group and the later formed point chirality in the formal [4 + 2]-cycloaddition reaction, and the low *dr* may due to the lack of selectivity control of the electrophilic aromatic substitution step. The installation of cyclohexenyl group proximal to the alkyne motif instead of aryl led to the product **21** as two isomers in 33% and 50% yields, respectively, and with **21b** in 1.7:1 *dr*.

**Fig. 3:**
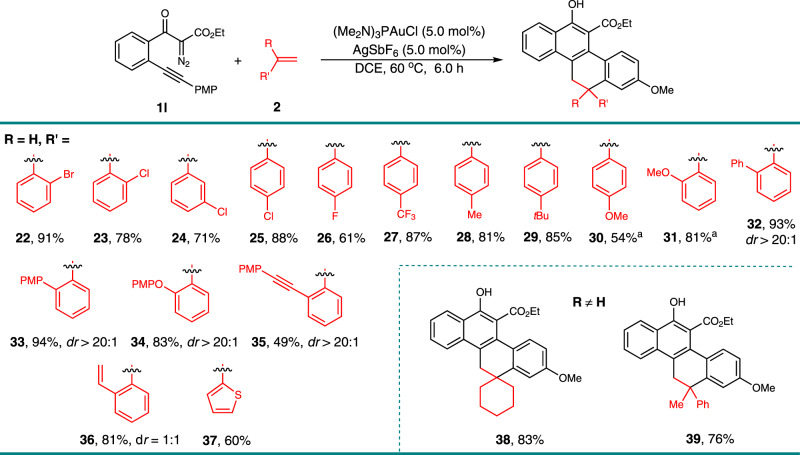
Scope with respect to the olefins **2**. Reaction condition: to a solution of (Me_2_N)_3_PAuCl (3.95 mg, 0.01 mmol), and AgSbF_6_ (3.4 mg, 0.01 mmol) in DCE (0.5 mL), we added a solution of **1** **l** (PMP = 4-MeOC_6_H_4_, 69.6 mg, 0.2 mmol) and olefins **2** (0.3 mmol) in DCE (0.5 mL) at 60 °C, then the reaction mixture was stirred for 6 h under these conditions. Isolated yields are listed. ^a^DCE (6.0 mL) was used.

To further explore the substrate scope of this gold-catalyzed [4 + 2]-cycloaddition, we next examined a variety of olefins **2** (Fig. 3). The electronic effects and the position of the substituent groups on the phenyl ring of the styrene had little influence; substrates containing bromo, chloro, fluoro, trifluoromethyl, methyl, *tert*-butyl, and methoxy groups effectively reacted with **1l** to form the tetracyclic products **22**–**30** in high yields. Relatively high yields were obtained when an electron-withdrawing substituent was incorporated in the arene. Olefins with one bulky *ortho*-substituent were also tolerated in this reaction, selectively affording the corresponding products **31–35** as signal diastereomer in 49–94% yields. Even 1,2-divinylbenzene could be used, interestingly only the mono-cycloaddition product **36** was generated in 81% yield as a mixture of two diastereomers. The diastereomers resulted from the additionally formed axial chirality due to the hindered rotation of the *ortho*-substituted aryl group in these products. The heteroaromatic substituted alkene, 2-vinylthiophene, delivered the corresponding product **37** in 60% yield. Despite the inefficiency of the reaction with internal alkenes, which only generated the intramolecular cyclization by-products from **1l**, the disubstituted terminal alkenes, methylenecyclohexane and 1-methyl-1-phenylethene, worked well, leading to the cycloadducts **38** and **39** in 83% and 76% yields, respectively. The structures of **23** and **34** were confirmed by single-crystal X-ray diffraction analysis.

### Development of the oxidation procedure for polycyclic aromatic hydrocarbons (PAHs)

**Fig. 4:**
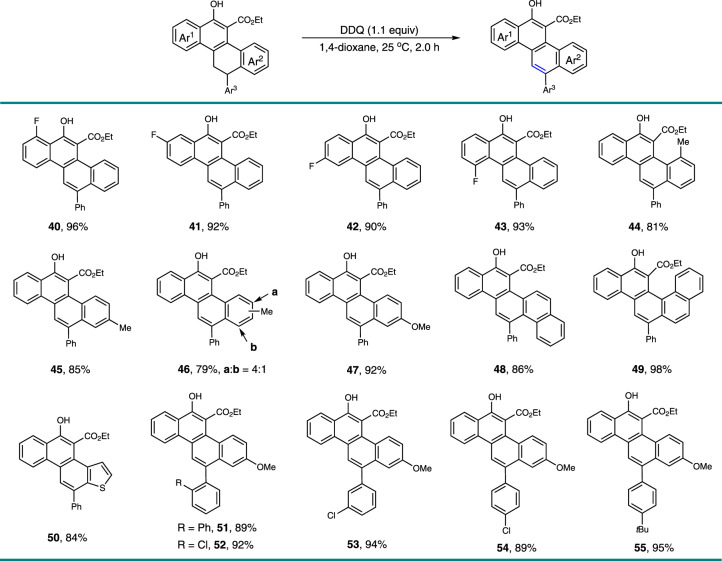
Preparation of polycyclic aromatic hydrocarbons (PAHs). Reaction condition: the generated formal [4+2]-cycloaddition adducts (0.10 mmol), 2,3-Dicyano-5,6-dichlorobenzoquinone (DDQ, 25.0 mg, 0.11 mmol), and 1,4-dioxane (6.0 mL) were added in sequence at 25 °C, and the reaction mixture was stirred for 12 h under these conditions. Isolated yields are listed.

With these polycarbocyclic products in hand, we then achieved their transformation to the corresponding CPHs. The aromatization occurred smoothly in the presence of 2,3-dichloro-5,6-dicyano-1,4-benzoquinone (DDQ) under mild and neutral conditions (Fig. 4). Various functional groups, such as phenolic hydroxyl, ester, methoxy, halogen, alkyl, and aryl were well tolerated, assembling multiple substituted CPHs **40**–**55** in high to excellent yields. In addition to the chrysene derivatives, a few of elusive CPHs, including picene (**48**, 86%), benzo[*c*]chrysene (**49**, 98%), and phenanthro[2,1-*b*]thiophene (**50**, 84%) were also readily prepared in high yields. It could be envisioned that additional different types of polyaromatic hydrocarbons with structural diversity would become accessible with this method by the manipulation of the structure of the substrates or via further synthetic transformations.

### Optical properties

**Fig. 5:**
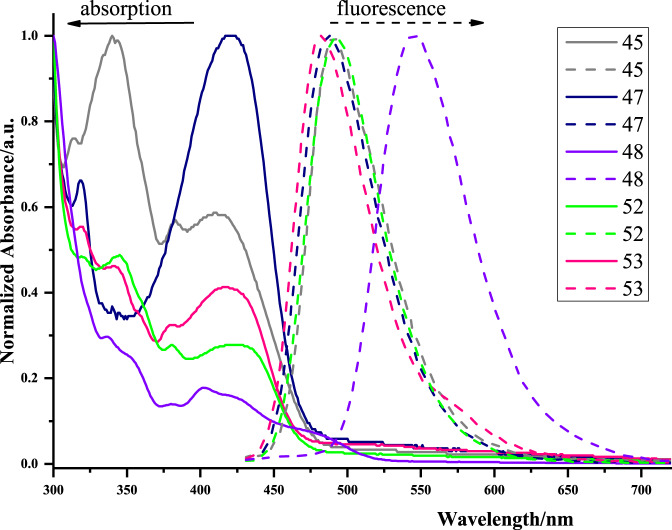
The UV/Vis absorption (solid lines) and emission spectra (broken lines) in DMSO. The extension of π-conjugated system to enlarge the maximum emission of PAHs.

After the construction of these elusive CPHs, we then investigated the optical properties of representative analogous in DMSO (Fig. 5). The absorption spectra of tested PAHs displayed the λ_max_ in the range of 402–421 nm. For the fluorescence spectra, compounds **45**, **47**, **52**, and **53** exhibited sky-blue lights with similar peaks at around 490 nm; whereas, the five-fused aromatic product **48** showed green light with a maximum emission of 550 nm due to the extension of the π-conjugated system.

### Mechanistic discussion

**Fig. 6:**
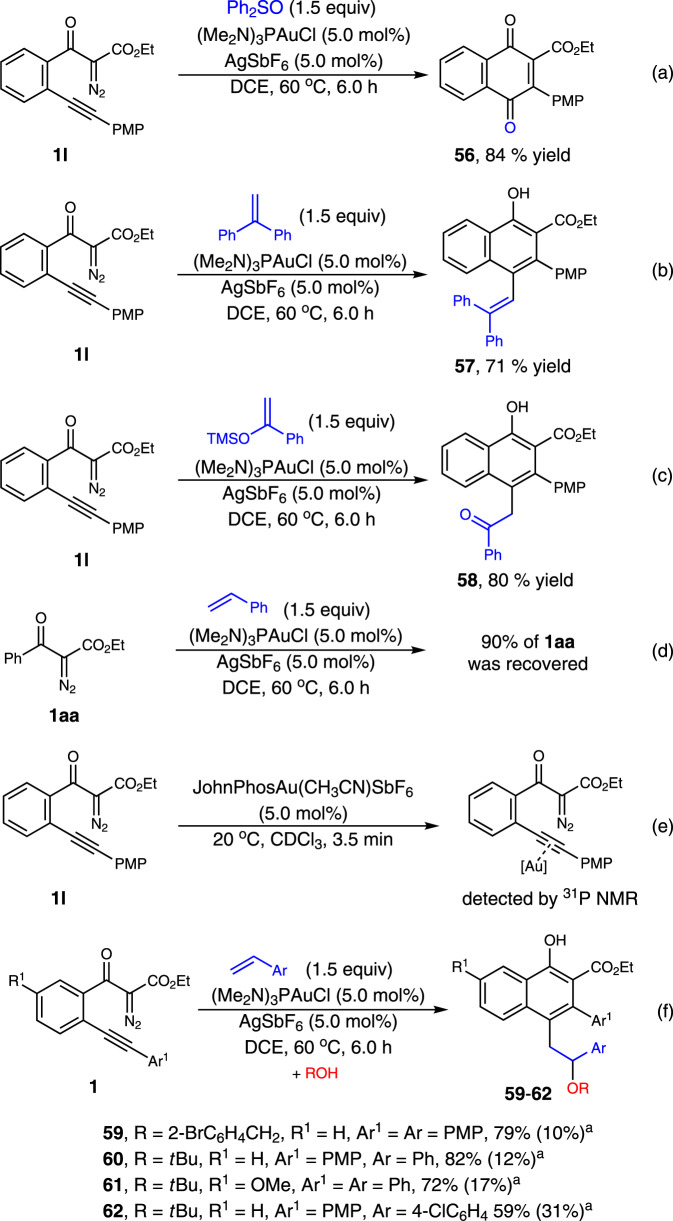
Mechanistic experiments. ^a^The data in parentheses are the yields of corresponding [4 + 2]-cycloadducts. **a** Control experiment with diphenyl sulfoxide. **b** Control experiment with 1,1-diphenylethylene. **c** Control experiment with 1-phenyl-1-trimethylsiloxyethylene. **d** Control experiment with 1,3-dicarbonyl diazo compound **1aa**. **e** Control experiment in the absence of alkene. **f** Three-component reaction of diazo compounds with alkenes and alcohols.

**Fig. 7:**
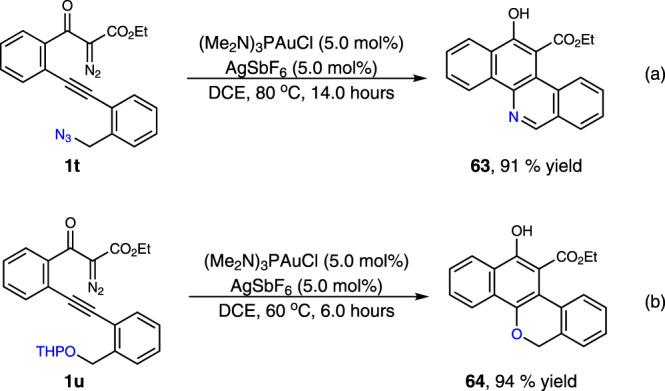
Synthetic applications of current strategy. **a** Synthesis of benzo[*c*]phenanthridine **63**. **b** Synthesis of 6*H*-dibenzo[*c*,*h*]chromene **64**.

Mechanistic experiments were performed to gain insights into the reaction pathway of this transformation (Fig. 6). To verify the existence of the on-ring β-aryl gold-carbene intermediate, the interception reaction with **1l** in the presence of diphenyl sulfoxide (1.5 equiv.), instead of styrene, was carried out under standard conditions, and the corresponding cyclic ketone product **56** was isolated in 84% yield (Fig. 6a). These results are also consistent with a direct 6-*endo*-*dig* diazo-yne carbocyclization process for the generation of this on-ring carbene intermediate, otherwise, the linear ketone product that furnished via direct carbonylation of the diazo group might be observed^44^. Evidence for the carbocation-like reactivity of this generated gold-complex was verified by the reactions of **1l** with two disubstituted terminal alkenes, 1,1-diphenylethylene and 1-phenyl-1-trimethylsiloxyethylene, delivering the coupling-type and addition-type products **57** and **58** in 71% and 80% yields, respectively (Fig. 6b, c). The comparison reaction with 1,3-dicarbonyl diazo compound **1aa** without the alkyne species turned out that only very slow decomposition of the diazo compound was observed under the current conditions (Fig. 6d). The ^31^P NMR analysis results, by mixing the gold catalyst (5.0 mol%) with **1l** (0.02 mmol) in CDCl_3_ at 20 °C suggested that, rather than direct decomposition of the diazo species^56–58^, the formation of a relatively stable Au-alkyne complex is favorable under these conditions (Fig. 6e, and see Supplementary Fig. 2 for details). Moreover, a non-concerted, stepwise mechanism of the cyclization process was well supported by the interception reaction with external alcohol. The identifiable three-component products **59**–**62** were isolated in 59–82% yield when the reaction was carried out in the presence of *o*-bromobenzyl alcohol or tertiary butanol (Fig. 6f). In addition, this protocol could also be applied for the preparation of benzo[*c*]phenanthridine **63** and 6*H*-dibenzo[*c*,*h*]chromene **64** from corresponding materials in 91% and 94% yields, respectively (Fig. 7a, b). All these results well rationalized the reaction mechanism, and underlined the synthetic potential of this method in diversity-oriented synthesis.

**Fig. 8:**
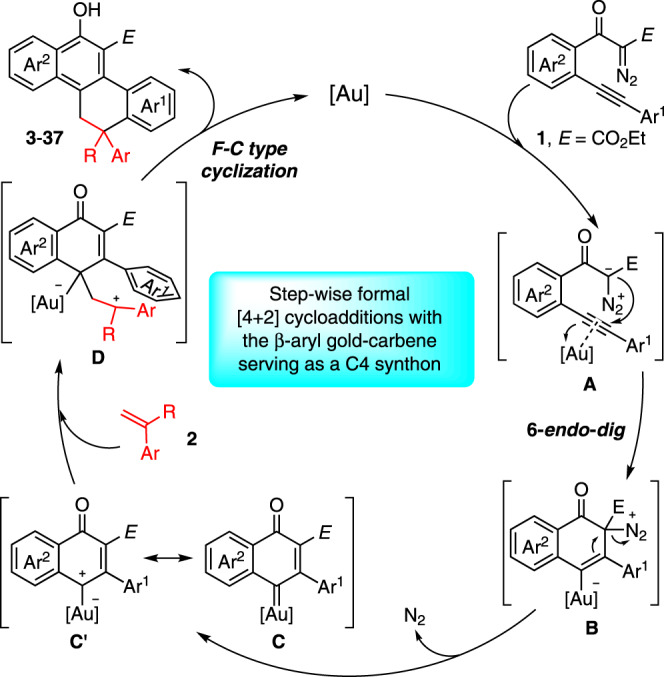
Proposed reaction mechanism. The formal [4 + 2]-cycloaddition of in situ generated β-aryl gold-carbene intermediate with alkenes.

Based on the above studies and the reported literature^41–48^, a possible reaction mechanism is depicted in Fig. 8. Initially, the gold-promoted 6-*endo*-*dig* carbocyclization of **1** formed intermediate **B** via **A**, which further led to the key intermediate vinyl gold-carbene **C** after the extrusion of N_2_. The resonance phenomenon of this gold-complex between carbene (**C**) and carbocation (**C′**) forms, which mainly depends on the ligands of the gold catalyst, might be existing. In this case, carbocation-like intermediate **C′** has been suggested based on the observations of the control experiments (Fig. 6). Subsequently, this gold intermediate reacted with an external alkene to form the carbocation intermediate **D**, followed by a Friedel-Crafts-type cyclization, furnishing the corresponding polycyclic products, and regenerating the gold catalyst.

### Applications

**Fig. 9:**
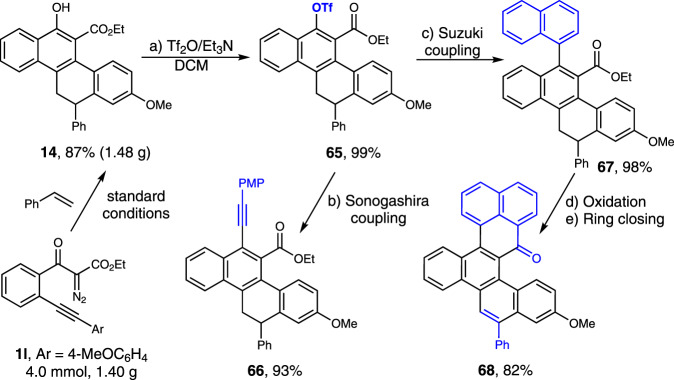
Synthetic transformations. The product **14** was prepared on a 4.0 mmol scale under optimal conditions. **a** Sulfonylation of the phenolic hydroxyl group. **b** Sonogashira coupling. **c** Suzuki coupling. **d** Oxidation. **e** Ring closing.

To demonstrate the utility of the current method, we performed the reaction on a gram scale (Fig. 9, 4.0 mmol), providing 1.48 g of **14** in 87% yield. Then, the cycloadduct **14** was subjected to further transformations. Sulfonylation of the phenolic hydroxyl group with trifluoromethanesulfonic anhydride (Tf_2_O) led to the coupling precursor **65** in quantitative yield. The following Sonogashira and Suzuki coupling reactions with terminal alkyne and naphthylboronic acid gave **66** and **67** in 93% and 98% yields, respectively. Oxidation of **67** with DDQ followed by an acid-promoted Friedel-Crafts-type intramolecular cyclization delivered the polycyclic hydrocarbon **68** in a total 82% yield for the two steps. We also studied the enantioselective version of this cascade reaction with a variety of chiral phosphine ligands. So far, only up to 16% *ee* with low reactivity (25% conversion and 15% yield) has been obtained with these tested ligands (see Supplementary Table 1 for details).

## Discussion

We have developed a gold-catalyzed diazo-yne cyclization/intermolecular [4 + 2]-cycloaddition reaction of alkyne-containing diazo compounds with alkenes. Mechanistic studies indicate that the β-aryl gold-carbene is the key intermediate in this cascade transformation, which is generated via gold-promoted 6-*endo*-*dig* diazo-yne cyclization and served as a 4-C synthon in following stepwise [4 + 2]-cycloaddition. A variety of polycarbocyclic frameworks are synthesized in good to high yields, and these generated products could be readily converted to elusive CPHs with multiple substituents under a mild oxidation procedure. Owing to their various useful properties including their intriguing pharmacological activities^65^, and electrical and optical properties^66^, the CPHs have attracted significant attention^67,68^. Preliminary photophysical studies have been conducted with representative CPHs, which emit sky-blue and green fluorescence. This protocol complements the common carbene/alkyne metathesis strategies in terms of chemoselectivity and reactivity patterns. Cascade transformations and synthetic applications could be envisioned in due course with the unique carbene species in situ generated under gold-catalysis.

## Methods

### General methods

See Supplementary Methods for further details.

### Typical procedure for the gold-catalyzed formal [4**+**2] cycloaddition

To a 10-mL oven-dried vial containing a magnetic stirring bar, (Me_2_N)_3_PAuCl (3.95 mg, 0.01 mmol), AgSbF_6_ (3.43 mg, 0.01 mmol), and DCE (0.5 mL) were added in sequence in a nitrogen-filled glove-box. The reaction mixture was stirred at 25 °C for 2 h. The solvent was removed and the residue was dissolved in DCE (0.5 mL). Then the mixture was filtered through a pad of Celite. The filtrate was added into a solution of **1** (0.2 mmol) and **2** (olefin, 0.3 mmol) in DCE (0.5 mL) at 60 °C, and the resulting reaction mixture was stirred under these conditions for 6 h. Then, the solvent was removed under reduced pressure and the crude product was purified by column chromatography on silica gel (eluent: Ethyl acetate/light petroleum ether = 1/30~1/10) to afford the polycyclic compounds **3**–**39** in good to high yields.

### Typical procedure for the oxidative aromatization

To a 10-mL oven-dried flask equipped with a magnetic stirring bar, the above-prepared polycyclic products (0.10 mmol), 2,3-Dicyano-5,6-dichlorobenzoquinone (DDQ, 25.0 mg, 0.11 mmol), and 1,4-dioxane (6.0 mL) were added in sequence. The reaction mixture was stirred at 25 °C for 12 h. Then, the solvent was removed under reduced pressure and the crude product was purified by column chromatography on silica gel (eluent: petroleum ether/ethyl acetate = 20:1) to give the PAHs **40**–**55** in high to excellent yields.

## Supplementary information

Supplementary Information

## Data Availability

Additional data supporting the findings described in this manuscript are available in the Supplementary Information. For full characterization data of new compounds and experimental details, see Supplementary Methods and Figures in Supplementary Information file. The X-ray crystallographic coordinates for structures **23** and **34** reported in this study have been deposited at the Cambridge Crystallographic Data Centre (CCDC), under deposition number 1828268 (**23**) and 1849634 (**34**). These data can be obtained free of charge from The Cambridge Crystallographic Data Centre via http://www.ccdc. cam.ac.uk/data_request/cif. All other data are available from the authors upon reasonable request. Source data are provided with this paper.

## Source data

Source Data
