# Supplementary material for: Gold(I)-catalyzed intramolecular cyclization/intermolecular cycloaddition cascade as a fast track to polycarbocycles and mechanistic insights
Source: Nat Commun. 2021 Feb 19;12:1182. doi: 10.1038/s41467-021-21335-9 (PMC7896061; doi:10.1038/s41467-021-21335-9)
Supplement: Supplementary file 1 — Supplementary Information [file 41467_2021_21335_MOESM1_ESM.pdf]

Supplementary Information

Gold(I)-catalyzed intramolecular cyclization/intermolecular cycloaddition cascade as a fast track to polycarbocycles and mechanistic insights

Cheng Zhang *et al*

Supplementary Methods

General Information

All the reactions were carried out under argon atmosphere using oven-dried glassware. Dichloroethane (DCE), ethyl diazoacetate, olefins, phosphine ligands, and metal catalysts were purchased from chemical companies and were used without further treatment. Flash column chromatography was performed using a silica gel (300-400 mesh). Analytical thin-layer chromatography was performed using glass plates precoated with 200-300 mesh silica gel impregnated with a fluorescent indicator (254 nm). All the new compounds were fully characterized. ^{1}H NMR and ^{13}C NMR spectra were recorded in $CDCl_3$, $CDCl_2CDCl_2$ or DMSO-d_6 using a 300/400/500/600/700 MHz spectrometer, and chemical shifts were reported in ppm with the solvent signals as the reference, and coupling constants (*J*) were given in Hz. The peak information was described as: s = singlet, br = broad, d = doublet, t = triplet, q = quartet, m = multiplet, and comp = composite. High-resolution mass spectra (HRMS) were recorded using a commercial apparatus (ESI Source, CI or EI Source).

General Procedure for the Preparation of Diazo Compounds 1

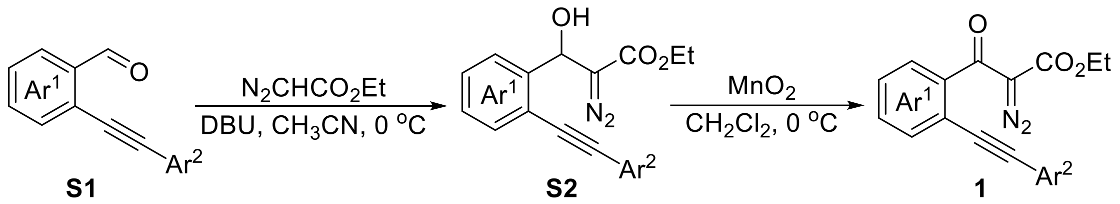

All of the materials **S1** were prepared were prepared according to literature procedures[1].

Synthesis of **S2**: To a solution of ethyl diazoacetate (EDA, 1.37 g, 12.0 mmol) in CH_3CN (10.0 mL), **S1** (10.0 mmol) and 1,8-diazabicyclo [5.4.0] undec-7-ene (DBU, 0.15 g, 1.0 mmol) were added at 0 ˚C in sequence. After the mixture was stirred at 25 ˚C for 12.0 hours, the reaction was quenched with saturated aqueous NH_4Cl (15 mL) and then extracted with CH_2Cl_2 (3 × 20.0 mL). The combined organic phase was dried over Na_2SO_4 and the solvent was evaporated under vacuum after filtration. The obtained product **S2** was directly used for the next step without further purification.

Synthesis of **1**: To a 50-mL oven-dried flask containing a magnetic stirring bar, the above obtained product **S2** in DCM (20.0 mL), was added manganese dioxide (MnO_2, 8.70 g, 100.0 mmol) slowly at 25 ˚C. The reaction mixture was stirred at overnight and then the solvent was evaporated under vacuum after filtering through a pad of Celite, and the resulting residue was purified by column chromatography on silica gel (eluent: petroleum ether/ ethyl acetate = 10:1) to give the pure diazoacetates **1** in good to high yields.

Characterization of Diazo Compounds 1.

Ethyl 2-diazo-3-oxo-3-(2-(phenylethynyl)phenyl)propanoate (1a).

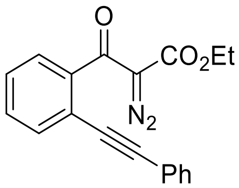

Yellow solid, 2.64 g, 83% yield. ^{1}H NMR (400 MHz, $CDCl_3$) (δ, ppm) 7.58 – 7.53 (m, 1H), 7.49 – 7.45 (comp, 2H), 7.45 – 7.36 (comp, 3H), 7.37 – 7.29 (comp, 3H), 4.15 (q, *J* = 7.1 Hz, 2H), 1.09 (t, *J* = 7.1 Hz, 3H). ^{13}C NMR (100 MHz, $CDCl_3$) (δ, ppm) 187.1, 160.9, 140.5, 132.0, 131.5, 130.4, 128.7, 128.4, 128.2, 127.1, 122.6, 121.0, 94.0, 86.6, 61.6, 13.9. HRMS (TOF MS ESI$^+$) calculated for $C_{19}H_{14}N_2NaO_3^+$ $[M+Na]^+$: 341.0897, found: 341.0902.

Ethyl 2-diazo-3-(2-fluoro-6-(phenylethynyl)phenyl)-3-oxopropanoate (**1b**).

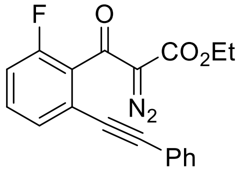

Yellow solid, 2.52 g, 75% yield. ^{1}H NMR (300 MHz, $CDCl_3$) (δ, ppm) 7.50 – 7.43 (comp, 2H), 7.42 – 7.29 (comp, 5H), 7.14 – 7.05 (m, 1H), 4.17 (q, *J* = 7.1 Hz, 2H), 1.13 (t, *J* = 7.1 Hz, 3H); ^{13}C NMR (75 MHz, $CDCl_3$) (δ, ppm) 182.9 (d, *J* = 1.3 Hz), 160.4, 157.1, 131.7, 131.2 (d, *J* = 9.1 Hz), 129.0, 128.5, 128.0 (d, *J* = 3.2 Hz), 122.7 (d, *J* = 4.9 Hz), 122.4, 115.8 (d, *J* = 21.5 Hz), 94.4, 85.4 (d, *J* = 4.0 Hz), 61.8, 14.0; ^{19}F NMR (283 MHz, $CDCl_3$) (δ, ppm) -115.68. HRMS (TOF MS ESI$^+$) calculated for $C_{19}H_{13}FN_2NaO_3^+$ $[M+Na]^+$: 359.0802, found: 359.0831.

Ethyl 2-diazo-3-(5-fluoro-2-(phenylethynyl)phenyl)-3-oxopropanoate (**1c**).

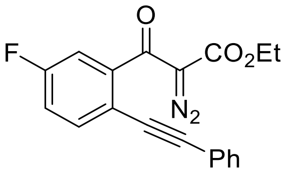

Yellow solid, 2.93 g, 87% yield. ^{1}H NMR (300 MHz, $CDCl_3$) (δ, ppm) 7.61 – 7.53 (m, 1H), 7.52 – 7.45 (comp, 2H), 7.43 – 7.31 (comp, 3H), 7.28 – 7.07 (m, 2H), 4.20 (q, *J* = 7.1 Hz, 2H), 1.15 (t, *J* = 7.1 Hz, 3H); ^{13}C NMR (75 MHz, $CDCl_3$) (δ, ppm) 185.8 (d, *J* = 2.0 Hz), 162.0 (d, *J* = 251.7 Hz), 160.7, 142.5 (d, *J* = 7.3 Hz), 134.0 (d, *J* = 8.1 Hz), 131.5, 128.8, 128. 5, 122.5, 117.6 (d, *J* = 22.0 Hz), 117.3 (d, *J* = 3.7 Hz), 114.6 (d, *J* = 24.1 Hz), 93.7 (d, *J* = 1.6 Hz), 85.6, 61.8, 14.0; ^{19}F NMR (283 MHz, $CDCl_3$) (δ, ppm) -109.87. HRMS (TOF MS ESI$^+$) calculated for $C_{19}H_{13}FN_2NaO_3^+$ $[M+Na]^+$: 359.0802, found: 359.0812.

Ethyl 2-diazo-3-(4-fluoro-2-(phenylethynyl)phenyl)-3-oxopropanoate (**1d**).

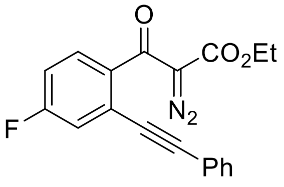

Yellow solid, 2.69 g, 80% yield. ^{1}H NMR (300 MHz, $CDCl_3$) (δ, ppm) 7.54 – 7.42 (comp, 3H), 7.42 – 7.31 (comp, 3H), 7.30 – 7.22 (m, 1H), 7.16 – 7.06 (m, 1H), 4.19 (q, *J* = 7.2 Hz, 2H), 1.14 (t, *J* = 7.1 Hz, 3H); ^{13}C NMR (75 MHz, $CDCl_3$) (δ, ppm) 186.1, 163.4 (d, *J* = 251.6 Hz), 160.9, 136.8 (d, *J* = 3.4 Hz), 131.6, 129.1, 129.7 (d, *J* = 9.4 Hz), 128.5, 123.6 (d, *J* = 10.3 Hz), 122.1, 118.8 (d, *J* = 23.5 Hz), 115.7 (d, *J* = 22.0 Hz), 95.0, 85.6 (d, *J* = 3.0 Hz), 61.7, 14.9; ^{19}F NMR (283 MHz, $CDCl_3$) (δ, ppm) -109.03. HRMS (TOF MS ESI$^+$) calculated for $C_{19}H_{13}FN_2NaO_3^+$ [M+Na]$^+$: 359.0802, found: 359.0808.

Ethyl 2-diazo-3-(3-fluoro-2-(phenylethynyl)phenyl)-3-oxopropanoate (**1e**).

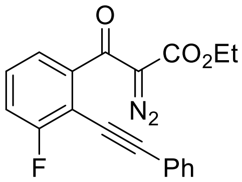

Yellow solid, 2.39 g, 71% yield. ^{1}H NMR (300 MHz, $CDCl_3$) (δ, ppm) 7.54 – 7.44 (comp, 2H), 7.42 – 7.29 (comp, 4H), 7.25 – 7.06 (m, 2H), 4.16 (q, *J* = 7.1 Hz, 2H), 1.11 (t, *J* = 7.1 Hz, 3H); ^{13}C NMR (75 MHz, $CDCl_3$) (δ, ppm) 185.9 (d, *J* = 3.2 Hz), 162.1 (d, *J* = 253.0 Hz), 160.7, 142.4, 131.7, 129.7 (d, *J* = 8.3 Hz), 129.1, 128.5, 122.8 (d, *J* = 3.6 Hz), 117.4 (d, *J* = 21.5 Hz), 110.2 (d, *J* = 17.9 Hz), 99.1 (d, *J* = 3.7 Hz), 80.0 (d, *J* = 0.6 Hz) , 61.8, 14.0; ^{19}F NMR (283 MHz, $CDCl_3$) (δ, ppm) -109.16. HRMS (TOF MS ESI$^+$) calculated for $C_{19}H_{13}FN_2NaO_3^+$ [M+Na]$^+$: 359.0802, found: 359.0822.

Ethyl 2-diazo-3-(5-methyl-2-(phenylethynyl)phenyl)-3-oxopropanoate (**1f**).

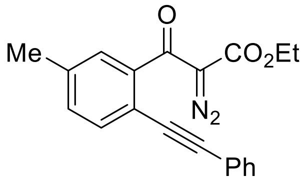

Yellow solid, 2.69 g, 81% yield. ^{1}H NMR (300 MHz, $CDCl_3$) (δ, ppm) 7.52 – 7.43 (comp, 2H), 7.42 – 7.26 (comp, 5H), 7.24 – 7.16 (m, 1H), 4.16 (q, *J* = 7.2 Hz, 2H), 2.37 (s, 3H), 1.11 (t, *J* = 7.2 Hz, 3H); ^{13}C NMR (75 MHz, $CDCl_3$) (δ, ppm) 187.1, 161.0, 140.9, 137.8, 132.6, 131.5, 129.2, 128.6, 128.4, 127.5, 122.8, 121.1, 93.6, 86.9, 61.6, 21.2, 14.0. HRMS (TOF MS ESI$^+$) calculated for $C_{20}H_{16}N_2NaO_3^+$ [M+Na]$^+$: 355.1053, found: 355.1070.

Ethyl 2-diazo-3-(5-methoxy-2-(phenylethynyl)phenyl)-3-oxopropanoate (**1g**).

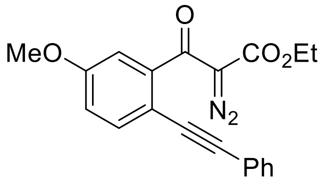

2.54 g, 73% yield. ^{1}H NMR (300 MHz, $CDCl_3$) (δ, ppm) 7.53 – 7.40 (comp, 3H), 7.38 – 7.26 (comp, 3H), 7.03 – 6.93 (m, 2H), 4.18 (q, *J* = 7.1 Hz, 2H), 3.84 (s, 3H), 1.13 (t, *J* = 7.1 Hz, 3H); ^{13}C NMR (75 MHz, $CDCl_3$) (δ, ppm) 186.9, 160.9, 159.5, 141.9, 133.5, 131.3, 128.4 128.3, 123.0, 116.6, 113.2, 112.4, 92.6, 86.6, 61.7, 55.5, 14.0. HRMS (TOF MS ESI$^+$) calculated for $C_{20}H_{16}N_2NaO_4^+$ [M+Na]$^+$: 371.1002, found: 371.1000.

Ethyl 2-diazo-3-oxo-3-(1-(phenylethynyl)naphthalen-2-yl)propanoate (**1h**).

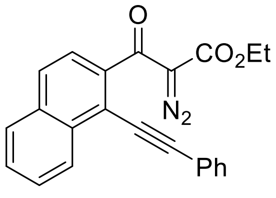

Yellow solid, 2.57 g, 70% yield. ^{1}H NMR (300 MHz, $CDCl_3$) (δ, ppm) 8.70 – 8.33 (m, 1H), 8.01 – 7.85 (comp, 2H), 7.73 – 7.54 (comp, 4H), 7.54 – 7.48 (m, 1H), 7.47 – 7.26 (comp, 3H), 4.13 (q, *J* = 7.1 Hz, 2H), 1.01 (t, *J* = 7.1 Hz, 3H); ^{13}C NMR (75 MHz, $CDCl_3$) (δ, ppm) 187.8, 161.1, 138.9, 133.9, 132.7, 131.7, 129.0, 128.8, 128.6, 128.4, 127.8, 127.7, 127.0, 123.8, 122.9, 119.1, 99.9, 84.8, 61.8, 14.0. HRMS (TOF MS ESI$^+$) calculated for $C_{23}H_{16}N_2NaO_3^+$ [M+Na]$^+$: 391.1053, found: 391.1060.

Ethyl 2-diazo-3-oxo-3-(2-(*o*-tolylethynyl)phenyl)propanoate (**1i**).

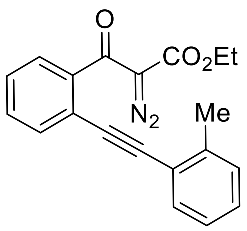

Yellow solid, 2.69 g, 81% yield. ^{1}H NMR (400 MHz, $CDCl_3$) (δ, ppm) 7.59 (d, *J* = 7.4 Hz, 1H), 7.54 – 7.31 (comp, 4H), 7.30 – 7.22 (comp, 2H), 7.22 – 7.11 (m, 1H), 4.17 (q, *J* = 7.1 Hz, 2H), 2.48 (s, 3H), 1.12 (t, *J* = 7.1 Hz, 3H). ^{13}C NMR (100 MHz, $CDCl_3$) (δ, ppm) 187.5, 160.8, 140.6, 140.1, 132.2, 132.1, 130.3, 129.6, 128.8, 128.2, 126.9, 125.7, 122.6, 121.2, 93.0, 90.5, 61.7, 20.6, 14.1. HRMS (TOF MS ESI$^+$) calculated for $C_{20}H_{16}N_2NaO_3^+$ [M+Na]$^+$: 355.1053, found: 355.1062.

Ethyl 2-diazo-3-oxo-3-(2-(*m*-tolylethynyl)phenyl)propanoate (**1j**).

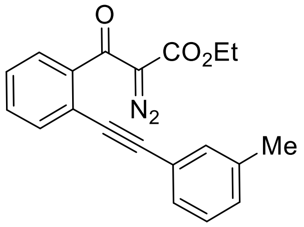

Yellow solid, 2.63 g, 79% yield. ^{1}H NMR (400 MHz, $CDCl_3$) (δ, ppm) 7.64 – 7.56 (m, 1H), 7.52 – 7.40 (comp, 3H), 7.38 – 7.24 (comp, 3H), 7.23 – 7.16 (m, 1H), 4.20 (q, *J* = 7.1 Hz, 2H), 2.38 (s, 3H), 1.14 (t, *J* = 7.1 Hz, 3H). ^{13}C NMR (100 MHz, $CDCl_3$) (δ, ppm) 187.3, 160.9, 140.6, 138.1, 132.1, 132.0, 130.4, 129.6, 128.7, 128.3, 128.2, 127.2, 122.5, 121.1, 94.3, 86.3, 61.7, 21.2, 14.0. HRMS (TOF MS ESI$^+$) calculated for $C_{20}H_{16}N_2NaO_3^+$ [M+Na]$^+$: 355.1053, found: 355.1060

Ethyl 2-diazo-3-oxo-3-(2-(*p*-tolylethynyl)phenyl)propanoate (**1k**).

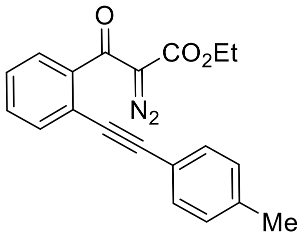

Yellow solid, 2.49 g, 75% yield. ^{1}H NMR (400 MHz, $CDCl_3$) (δ, ppm) 7.55 – 7.51 (m, 1H), 7.45 – 7.34 (comp, 5H), 7.18 – 7.10 (comp, 2H), 4.14 (q, *J* = 7.2 Hz, 3H), 2.35 (s, 3H), 1.09 (t, *J* = 7.1 Hz, 3H). ^{13}C NMR (100 MHz, $CDCl_3$) (δ, ppm) 187.3, 161.0, 140.5, 138.9, 131.9, 131.4, 130.4, 129.2, 128.1, 127.2, 121.3, 119.6, 94.3, 86.0, 61.6, 21.5, 14.0. HRMS (TOF MS ESI$^+$) calculated for $C_{20}H_{16}N_2NaO_3^+$ [M+Na]$^+$: 355.1053, found: 355.1065.

Ethyl 2-diazo-3-(2-((4-methoxyphenyl)ethynyl)phenyl)-3-oxopropanoate (**1l**).

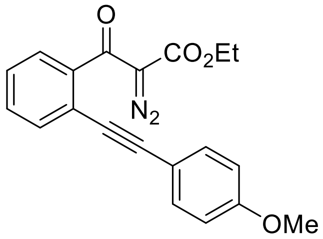

Yellow solid, 2.96 g, 85% yield. ^{1}H NMR (400 MHz, $CDCl_3$) (δ, ppm) 7.53 (d, *J* = 7.5 Hz, 1H), 7.49 – 7.29 (comp, 5H), 6.93 – 6.83 (comp, 2H), 4.16 (q, *J* = 7.1 Hz, 2H), 3.83 (s, 1H), 1.10 (t, *J* = 7.1 Hz, 3H). ^{13}C NMR (100 MHz, $CDCl_3$) (δ, ppm) 187.4, 161.1, 160. 0, 140.4, 133.1, 131.9, 130.5, 128.0, 127.2, 121.5, 114.8, 114.1, 94.3, 85.5, 61.7, 55.3, 14.0. HRMS (TOF MS ESI$^+$) calculated for $C_{20}H_{16}N_2NaO_4^+$ [M+Na]$^+$:371.1002, found: 371.1031.

Ethyl 2-diazo-3-(2-((4-fluorophenyl)ethynyl)phenyl)-3-oxopropanoate (**1m**).

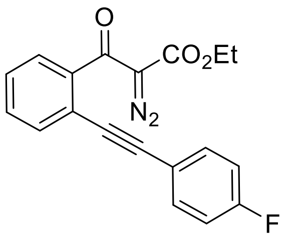

Yellow solid, 2.46 g, 73% yield. ^{1}H NMR (400 MHz, $CDCl_3$) (δ, ppm) 7.54 – 7.47 (m, 1H), 7.46 – 7.33 (comp, 5H), 7.05 – 6.95 (comp, 2H), 4.12 (q, *J* = 7.1 Hz, 2H), 1.06 (t, *J* = 7.1 Hz, 3H) ^{13}C NMR (100 MHz, $CDCl_3$) (δ, ppm) 187.0, 162.6 (d, *J* = 250.4 Hz), 160.8, 140.5, 133.4 (d, *J* = 8.4 Hz), 131.9, 130.3, 128.2, 127.2, 120.7, 118.7 (d, *J* = 3.5 Hz), 115.7 (d, *J* = 22.1 Hz), 92.8, 86.3, 61.6, 13.9. ^{19}F NMR (376 MHz, $CDCl_3$) (δ, ppm) -110.07. HRMS (TOF MS ESI$^+$) calculated for $C_{19}H_{13}FN_2NaO_3^+$ [M+Na]$^+$: 359.0802, found: 359.0822

Ethyl 3-(2-((4-chlorophenyl)ethynyl)phenyl)-2-diazo-3-oxopropanoate (1n).

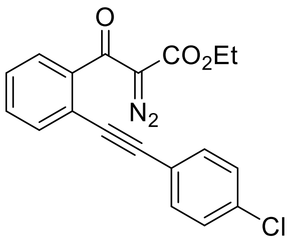

Yellow solid, 2.47 g, 70% yield. ^{1}H NMR (300 MHz, $CDCl_3$) (δ, ppm) 7.57 – 7.50 (m, 1H), 7.48 – 7.34 (comp, 5H), 7.35 – 7.26 (comp, 2H), 4.15 (q, *J* = 7.1 Hz, 2H), 1.09 (t, *J* = 7.1 Hz, 3H); ^{13}C NMR (75 MHz, $CDCl_3$) (δ, ppm) 187.1, 161.0, 140.6, 134.8, 132.8, 132.1, 130.5, 128.8, 128.5, 127.3, 121.2, 120.7, 92.8, 87.7, 61.8, 14.0. HRMS (TOF MS ESI$^+$) calculated for $C_{19}H_{13}ClN_2NaO_3^+$ [M+Na]$^+$: 375.0507, found: 375.0512.

Ethyl 2-diazo-3-(2-(naphthalen-2-ylethynyl)phenyl)-3-oxopropanoate (1o).

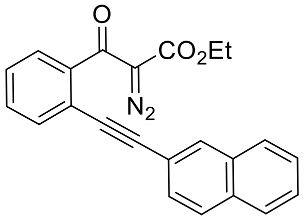

Yellow solid, 2.62 g, 71% yield. ^{1}H NMR (400 MHz, $CDCl_3$) (δ, ppm) 8.01 (s, 1H), 7.87 – 7.78 (comp, 3H), 7.65 – 7.59 (m, 1H), 7.56 – 7.40 (comp, 6H), 4.18 (q, *J* = 7.1 Hz, 2H), 1.11 (t, *J* = 7.1 Hz, 3H). ^{13}C NMR (100 MHz, $CDCl_3$) (δ, ppm) 187.4, 161.0, 140.7, 133.03, 132.98, 132.2, 131.7, 130.5, 128.4, 128.20, 128.16, 127.91, 127.87, 127.3, 127.0, 126.8, 121.2, 120.0, 94.5, 87.1, 61.8, 14.1. HRMS (TOF MS ESI$^+$) calculated for $C_{23}H_{16}N_2NaO_3^+$ [M+Na]$^+$: 391.1053, found 391.1051.

Ethyl 2-diazo-3-(2-(naphthalen-1-ylethynyl)phenyl)-3-oxopropanoate (1p).

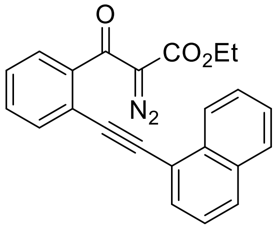

Yellow solid, 2.69 g, 73% yield. ^{1}H NMR (400 MHz, $CDCl_3$) (δ, ppm) 8.31 (d, *J* = 8.3 Hz, 1H), 7.94 – 7.81 (comp, 2H), 7.77 – 7.66 (comp, 2H), 7.64 – 7.58 (m, 1H), 7.58 – 7.41 (comp, 5H), 4.12 (q, *J* = 7.1 Hz, 2H), 1.04 (t, *J* = 7.1 Hz, 3H). ^{13}C NMR (100 MHz, $CDCl_3$) (δ, ppm) 187.6, 160.8, 140.8, 133.31, 133.26, 132.3, 130.7, 130.5, 129.3, 128.5, 128.4, 127.1, 126.6, 126.1, 125.4, 121.2, 120.5, 92.3, 91.6, 61.7, 14.0. HRMS (TOF MS ESI$^+$) calculated for $C_{23}H_{16}N_2NaO_3^+$ [M+Na]$^+$: 391.1053, found: 391.1062.

Ethyl 2-diazo-3-oxo-3-(2-(thiophen-2-ylethynyl)phenyl)propanoate (1q).

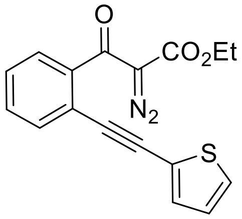

Yellow solid, 2.63 g, 81% yield. ^{1}H NMR (400 MHz, $CDCl_3$) (δ, ppm) 7.57 – 7.49 (m, 1H), 7.46 – 7.37 (comp, 3H), 7.33 – 7.27 (m, 1H), 7.25 – 7.19 (m, 1H), 7.03 – 6.96 (m, 1H), 4.16 (q, *J* = 7.1 Hz, 2H), 1.11 (t, *J* = 7.1 Hz, 3H). ^{13}C NMR (100 MHz, $CDCl_3$) (δ, ppm) 187.2, 160.9, 140.5, 132.4, 131.6, 130.5, 128.5, 128.0, 127.3, 122.6, 120.8, 90.5, 87.3, 61.7, 14.0. HRMS (TOF MS ESI$^+$) calculated for $C_{17}H_{12}N_2NaO_3S^+$ [M+Na]$^+$: 347.0461, found: 347.0464.

Ethyl 2-diazo-3-oxo-3-(2-(thiophen-3-ylethynyl)phenyl)propanoate (1r).

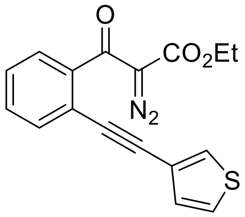

Yellow solid, 2.69 g, 83% yield. ^{1}H NMR (400 MHz, $CDCl_3$) (δ, ppm) 7.58 – 7.50 (comp, 2H), 7.47 – 7.38 (comp, 3H), 7.34 – 7.29 (m, 1H), 7.17 – 7.13 (m, 1H), 4.17 (q, *J* = 7.1 Hz, 2H), 1.11 (t, *J* = 7.1 Hz, 3H). ^{13}C NMR (100 MHz, $CDCl_3$) (δ, ppm) 187.1, 160.9, 140.5, 131.8, 130.4, 129.6, 129.2, 128.2, 127.2, 125.6, 121.6, 121.0, 89.2, 86.2, 61.6, 13.9. HRMS (TOF MS ESI$^+$) calculated for $C_{17}H_{12}N_2NaO_3S^+$ [M+Na]$^+$: 347.0461, found: 347.0469.

Ethyl 3-(2-(cyclohex-1-en-1-ylethynyl)phenyl)-2-diazo-3-oxopropanoate (**1s**).

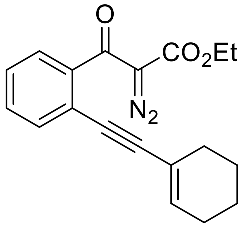

Yellow solid, 2.51 g, 78% yield. ^{1}H NMR (300 MHz, $CDCl_3$) (δ, ppm) 7.47 – 7.33 (comp, 4H), 6.24 – 6.14 (m, 1H), 4.19 (q, *J* = 7.1 Hz, 2H), 2.22 – 2.10 (comp, 4H), 1.75 – 1.59 (comp, 4H), 1.15 (t, *J* = 7.1 Hz, 3H); ^{13}C NMR (75 MHz, $CDCl_3$) (δ, ppm) 187.5, 161.1, 140.5, 136.1, 131.8, 130.4, 127.8, 127.1, 121.7, 120.5, 96.2, 84.1, 61.7, 29.0, 25.9, 22.3, 21.5, 14.1. HRMS (TOF MS ESI$^+$) calculated for $C_{19}H_{18}N_2NaO_3^+$ [M+Na]$^+$: 345.1210, found: 345.1212.

Ethyl 3-(2-((2-(azidomethyl)phenyl)ethynyl)phenyl)-2-diazo-3-oxopropanoate (**1t**).

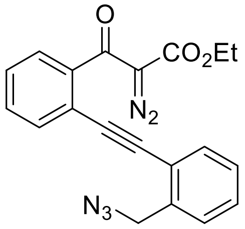

Yellow solid, 2.57 g, 69% yield. ^{1}H NMR (400 MHz, $CDCl_3$) (δ, ppm) 7.60 (d, *J* = 7.1 Hz, 1H), 7.55 – 7.49 (m, 1H), 7.49 – 7.43 (m, 1H), 7.43 – 7.26 (comp, 5H), 4.54 (s, 2H), 4.16 (q, *J* = 7.1 Hz, 2H), 1.12 (t, *J* = 7.1 Hz, 3H); ^{13}C NMR (100 MHz, $CDCl_3$) (δ, ppm) 187.0, 160.5, 140.4, 137.0, 132.5, 132.2, 130.2, 129.1, 128.6, 128.3, 128.2, 126.9, 122.2, 120.3, 91.4, 90.8, 61.5, 52.8, 13.9. HRMS (TOF MS ESI$^+$) calculated for $C_{20}H_{15}N_5NaO_3^+$ [M+Na]$^+$: 396.1067, found: 396.1071.

Ethyl 2-diazo-3-oxo-3-(2-((2-(((tetrahydro-2*H*-pyran-2-yl)oxy)methyl)phenyl)ethynyl)phenyl) propanoate (**1u**).

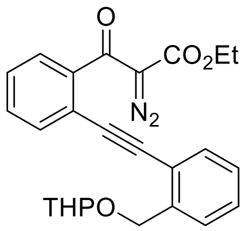

Yellow solid, 3.11 g, 72% yield. ^{1}H NMR (400 MHz, $CDCl_3$) (δ, ppm) 7.59 – 7.42 (comp, 2H), 7.41 – 7.34 (comp, 2H), 7.33 – 7.23 (comp, 3H), 7.22 – 7.15 (m, 1H), 4.75 (dd, *J* = 81.3, 13.0 Hz, 2H), 4.70 (t, *J* = 3.5 Hz, 1H), 4.07 (q, *J* = 7.1 Hz, 2H), 3.89 – 3.80 (m, 1H), 3.52 – 3.41 (m, 1H), 1.88 – 1.74 (m, 1H), 1.68 – 1.58 (m, 2H), 1.50 – 1.43 (m, 2H), 1.22 – 1.15 (m, 1H), 1.02 (t, *J* = 7.1 Hz, 3H); ^{13}C NMR (100 MHz, $CDCl_3$) (δ, ppm) 187.3, 160.9, 140.6, 140.5, 132.3, 132.3, 130.4, 129.0, 128.3, 127.6, 127.3, 127.1, 121.2, 121.1, 98.5, 91.7, 91.1, 67.2, 62.3, 61.7, 30.7, 25.6, 19.5, 14.1. HRMS (TOF MS ESI$^+$) calculated for $C_{25}H_{24}N_2NaO_5^+$ [M+Na]$^+$: 455.1577, found: 455.1574.

General Procedure for the Preparation of Au(I)-Catalysts

L1AuCl – **L7**AuCl were prepared according to literature procedures[2].

Preparation of $(Me_2N)_3PAuCl$: To a solution of the tris(dimethylamino)phosphine (163.2 mg, 1.0 mmol) in CH_2Cl_2 (5.0 mL), $(Me_2S)AuCl$ (294.6 mg, 1.0 mmol) was added under argon in the absence of light at 25 ºC, and the solution was stirring for 6 hours. After TLC indicated complete consumption of the starting materials, the reaction solution was removed under reduced pressure to give 395.0 mg $(Me_2N)_3PAuCl$ complex in quantitative yield, which was pure enough and used directly without additional treatment (stored in the glove box). The analysis of NMR is consistent with the literature[3]. ^1H NMR (300 MHz, $CDCl_3$) (δ, ppm) 2.66 (d, *J* = 11.7 Hz, 18H); ^{13}C NMR (75 MHz, $CDCl_3$) (δ, ppm) 37.8 (d, *J* = 9.2 Hz); ^{31}P NMR (122 MHz, $CDCl_3$) (δ, ppm) 110.75 (s).

Optimization of the Reaction Conditions

To a 10-mL oven-dried vial containing a magnetic stirring bar, the **Ln**AuCl complex (0.01 mmol), $AgSbF_6$ (3.34 mg, 0.01 mmol), and DCE (0.5 mL) were added in sequence in a nitrogen-filled glove-box. The reaction was stirred at 25 ˚C for 2.0 hours. The solvent was removed and the residue was dissolved in DCE (0.5 mL). The mixture was filtered through a pad of Celite. The filtrate was added into a solution of **1a** (63.6 mg, 0.2 mmol) and styrene (31.3 mg, 35.0 μL) in DCE (0.5 mL) at 60 ˚C, and the resulting reaction mixture was stirred under these conditions for 6.0 hours. Then, the crude the reaction mixture was subjected to ^1H NMR analysis with mesitylene as internal standard for the determination of the yields, and the results are summarized in Figure S1a. Moreover, these optimization results have shown good correlation with the calculated parameters of Au-Cl bond distance of gold-complexes with corresponding ligands (see Fig. S1b).

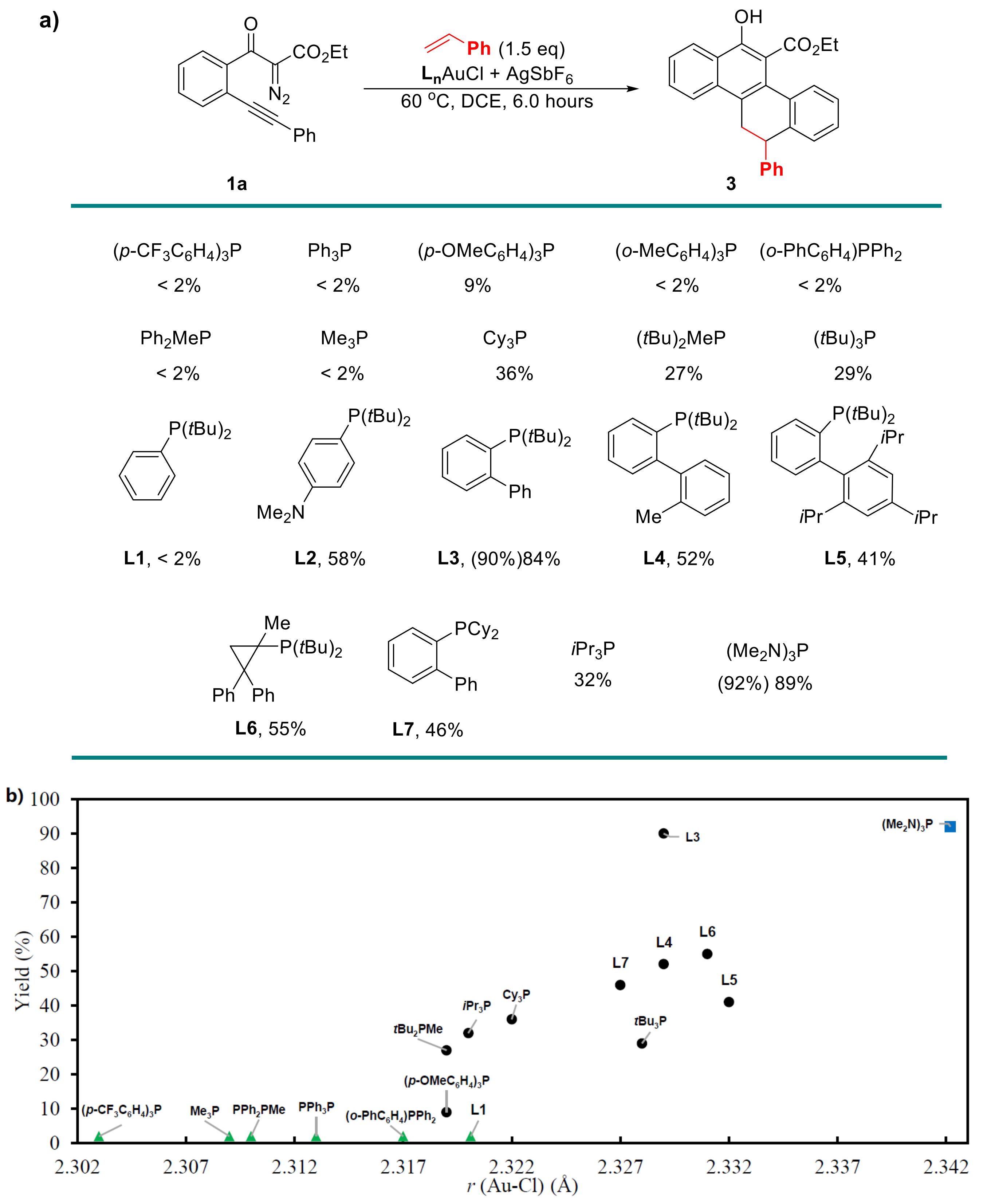

Supplementary Figure 1. **a**) Screening of phosphine ligands. **b**) Plot of yield versus distance of Au-Cl (*L*). *Note*: the calculated distance of Au-Cl bond may not correspond with the same absolute values as those reported in the literature[4].

General Procedure for the Gold-Catalyzed Formal [4+2] Cycloaddition:

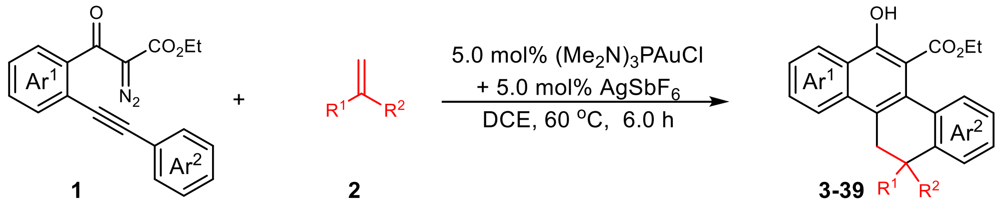

To a 10-mL oven-dried vial containing a magnetic stirring bar, $(Me_2N)_3PAuCl$ (3.95 mg, 0.01 mmol), $AgSbF_6$ (3.43 mg, 0.01 mmol), and DCE (0.5 mL) were added in sequence in a nitrogen-filled glove-box. The reaction mixture was stirred at 25 ˚C for 2.0 hours. The solvent was removed and the residue was dissolved in DCE (0.5 mL). Then the mixture was filtered through a pad of Celite. The filtrate was added into a solution of **1** (0.2 mmol) and **2** (olefin, 0.3 mmol) in DCE (0.5 mL) at 60 ˚C, and the resulting reaction mixture was stirred under these conditions for 6.0 hours. Then, the solvent was removed under reduced pressure and the crude product was purified by column chromatography on silica gel (eluent: Ethyl acetate/light petroleum ether =1/30~1/10) to afford the polycyclic compounds **3** – **39** in good to high yields. For **30** and **31**, 6.0 mL DCE was used in this cycloaddition; for **37**, 10.0 mol% of the catalyst was added in reaction.

Characterization of [4+2] Cycloaddition Products 3-39.

Ethyl 6-hydroxy-12-phenyl-11,12-dihydrochrysene-5-carboxylate (3).

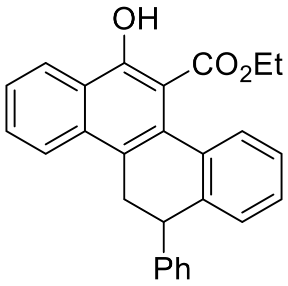

Yellow solid; m.p. 168.0 – 170.0 ˚C. 70.2 mg, 89% yield. ^{1}H NMR (400 MHz, $CDCl_3$) (δ, ppm) 11.13 (s, 1H), 8.55 – 8.35 (m, 1H), 7.94 (d, *J* = 8.4 Hz, 1H), 7.60 – 7.53 (m, 1H), 7.50 – 7.44 (m, 1H), 7.39 – 7.16 (comp, 8H), 7.04 (d, *J* = 7.3 Hz, 1H), 4.47 – 4.08 (comp, 3H), 3.68 – 3.11 (m, 2H), 1.08 (t, *J* = 7.1 Hz, 3H). ^{13}C NMR (100 MHz, $CDCl_3$) (δ, ppm) 172.1, 158.6, 142.8, 139.0, 135.5, 134.2, 131.2, 129.6, 129.4, 128.7, 128.4, 127.02, 126.98, 126.6, 125.81, 125.76, 124.5, 124.4, 123.2, 104.9, 61.4, 44.2, 32.3, 13.7. HRMS (TOF MS ESI$^+$) calculated for $C_{27}H_{22}NaO_3^+$ $[M+Na]^+$: 417.1461, found: 417.1450.

1-Ethoxy-3-phenyl-8*H*-indeno[1,2-*c*]furan-8-one (3').

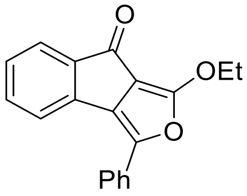

Yellow solid; m.p. 128.0 – 130.0 ˚C. 51.7 mg, 89% yield. ^{1}H NMR (300 MHz, $CDCl_3$) (δ, ppm) 7.82 – 7.72 (comp, 4H), 7.55 – 7.46 (comp, 3H), 7.42 – 7.30 (m, 2H), 4.90 (q, *J* = 7.1 Hz, 2H), 1.53 (t, *J* = 7.1 Hz, 3H); ^{13}C NMR (125 MHz, $CDCl_3$) (δ, ppm) 182.6, 157.4, 142.1, 138.2, 137.3, 133.5, 129.8, 129.0, 128.5, 128.3, 125.5, 125.3, 124.8, 122.5, 101.1, 70.9, 15.1. HRMS (TOF MS ESI$^+$) calculated for $C_{19}H_{14}NaO_3^+$ $[M+Na]^+$: 313.0835, found: 313.0850.

Ethyl 7-fluoro-6-hydroxy-12-phenyl-11,12-dihydrochrysene-5-carboxylate (4).

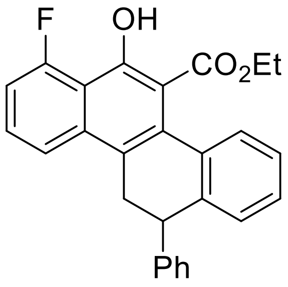

Yellow solid; m.p. 143.0 – 145.0 ˚C. 75.0 mg, 91% yield. ^{1}H NMR (300 MHz, $CDCl_3$) (δ, ppm) 11.12 (d, *J* = 2.1 Hz, 1H), 7.60 (d, *J* = 8.5 Hz, 1H), 7.36 – 7.28 (m, 1H), 7.21 – 7.07 (comp, 8H), 7.00 – 6.90 (comp, 2H), 4.25 – 4.04 (comp, 3H), 3.22 (d, *J* = 6.8 Hz, 2H), 0.94 (t, *J* = 7.1 Hz, 3H); ^{13}C NMR (75 MHz, $CDCl_3$) (δ, ppm) 171.7, 161.0 (d, *J* = 260.6 Hz), 158.1 (d, *J* = 3.9 Hz), 142.6, 139.0, 136.6 (d, *J* = 2.5 Hz), 135.2, 132.5 (d, *J* = 1.6 Hz), 129.7 (d, *J* = 9.6 Hz), 129.2, 128.7, 128.4, 127.4, 127.2, 126.7, 126.0, 125.4 (d, *J* = 2.4 Hz), 119.2 (d, *J* = 4.4 Hz), 114.3 (d, *J* = 8.8 Hz), 111.9 (d, *J* = 22.5 Hz), 106.3 (d, *J* = 1.9 Hz), 61.6, 44.1, 32.9, 13.6; ^{19}F NMR (283 MHz, $CDCl_3$) (δ, ppm) -109.47. HRMS (TOF MS

ESI$^+$) calculated for $C_{27}H_{21}FNaO_3^+$ [M+Na]$^+$: 435.1367, found: 435.1370.

Ethyl 8-fluoro-6-hydroxy-12-phenyl-11,12-dihydrochrysene-5-carboxylate (5).

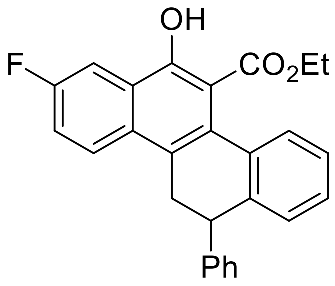

Yellow solid; m.p. 161.0 – 163.0 ˚C. 76.6 mg, 93% yield. ^{1}H NMR (300 MHz, $CDCl_3$) (δ, ppm) 10.83 (s, 1H), 7.86 (dd, *J* = 9.9, 2.7 Hz, 1H), 7.76 (dd, *J* = 9.3, 5.3 Hz, 1H), 7.20 – 7.03 (comp, 9H), 6.93 – 6.86 (m, 1H), 4.28 – 3.97 (comp, 3H), 3.19 (d, *J* = 6.9 Hz, 2H), 0.95 (t, *J* = 7.1 Hz, 3H); ^{13}C NMR (75 MHz, $CDCl_3$) (δ, ppm) 171.9, 160.7 (d, *J* = 246.9 Hz), 157.5 (d, *J* = 4.8 Hz), 142.6, 138.9, 135.3, 131.1 (d, *J* = 1.4 Hz), 130.6 (d, *J* = 2.6 Hz), 129.3, 128.7, 128.5, 127.1, 126.7, 125.9, 125.8, 125.7, 125.6, 125.5, 119.2 (d, *J* = 24.6 Hz), 108.7 (d, *J* = 22.4 Hz), 105.9, 61.6, 44.1, 32.5, 13.7; ^{19}F NMR (283 MHz, $CDCl_3$) (δ, ppm) -114.12. HRMS (TOF MS ESI$^+$) calculated for $C_{27}H_{21}FNaO_3^+$ [M+Na]$^+$: 435.1367, found: 435.1377.

Ethyl 9-fluoro-6-hydroxy-12-phenyl-11,12-dihydrochrysene-5-carboxylate (6).

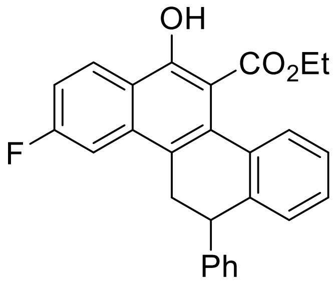

Yellow solid; m.p. 141.0 – 143.0 ˚C. 71.7 mg, 87% yield. ^{1}H NMR (300 MHz, $CDCl_3$) (δ, ppm) 11.01 (s, 1H), 8.23 (dd, *J* = 9.1, 6.0 Hz, 1H), 7.38 (dd, *J* = 11.2, 2.4 Hz, 1H), 7.18 – 7.00 (comp, 9H), 6.94 – 6.87 (m, 1H), 4.22 – 4.02 (comp, 3H), 3.12 (d, *J* = 6.8 Hz, 2H), 0.94 (t, *J* = 7.1 Hz, 3H); ^{13}C NMR (75 MHz, $CDCl_3$) (δ, ppm) 171.9, 163.6 (d, *J* = 249.6 Hz), 158.6 (d, *J* = 1.1 Hz), 142.5, 139.0, 136.0 (d, *J* = 9.2 Hz), 135.3, 132.7, 129.6, 128.7, 128.4, 127.5 (d, *J* = 9.7 Hz), 127.3, 127.2, 126.7, 125.9, 125.0 (d, *J* = 4.8 Hz), 121.3 (d, *J* = 1.1 Hz), 115.3 (d, *J* = 24.6 Hz), 107.6 (d, *J* = 22.2 Hz), 104.4 (d, *J* = 2.0 Hz), 61.5, 44.0, 32.5, 13.7; ^{19}F NMR (283 MHz, $CDCl_3$) (δ, ppm) -108.83. HRMS (TOF MS ESI$^+$) calculated for $C_{27}H_{21}FNaO_3^+$ [M+Na]$^+$: 435.1367, found: 435.1361.

Ethyl 10-fluoro-6-hydroxy-12-phenyl-11,12-dihydrochrysene-5-carboxylate (7).

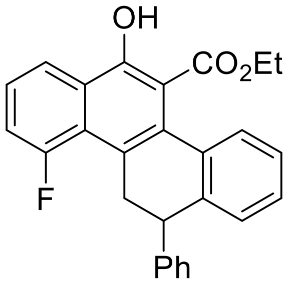

Yellow solid; m.p. 158.0 – 160.0 ˚C. 67.6 mg, 82% yield. ^{1}H NMR (400 MHz, $CDCl_3$) (δ, ppm) 10.90 (s, 1H), 8.03 (dd, *J* = 8.3, 0.9 Hz, 1H), 7.18 – 6.99 (comp, 10H), 6.95 – 6.86 (m, 1H), 4.20 – 3.99 (comp, 3H), 3.71 – 3.38 (m, 2H), 0.91 (t, *J* = 7.1 Hz, 3H); ^{13}C NMR (75 MHz, $CDCl_3$) (δ, ppm) 171.8, 160.2 (d, *J* = 252.8 Hz), 157.7 (d, *J* = 4.1 Hz), 142.8, 138.9, 135.4, 132.8 (d, *J* = 1.6 Hz), 129.3, 128.8, 128.3, 127.3, 126.9, 126.8 (d, *J* = 5.3 Hz), 126.5, 125.9, 125.5 (d, *J* = 9.1 Hz), 124.5 (d, *J* = 11.1 Hz), 124.2 (d, *J* = 5.0 Hz), 120.6 (d, *J* = 4.1 Hz), 115.4 (d, *J* = 24.0 Hz), 106.0 (d, *J* = 1.4 Hz), 61.6, 44.0 (d, *J* = 2.8 Hz), 34.6 (d, *J* = 16.0 Hz), 13.6; ^{19}F NMR (283 MHz, $CDCl_3$) (δ, ppm) -109.08. HRMS (TOF MS ESI$^+$) calculated for $C_{27}H_{21}FNaO_3^+$ [M+Na]$^+$: 435.1367, found: 435.1365.

Ethyl 6-hydroxy-8-methyl-12-phenyl-11,12-dihydrochrysene-5-carboxylate (8).

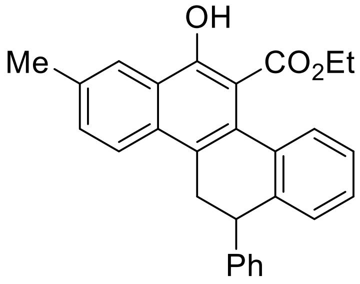

Yellow solid; m.p. 90.0 – 92.0 ˚C. 73.5 mg, 90% yield. ^{1}H NMR (300 MHz, $CDCl_3$) (δ, ppm) 11.07 (s, 1H), 8.27 (d, *J* = 8.5 Hz, 1H), 7.71 (s, 1H), 7.33 – 7.12 (comp, 9H), 7.02 – 6.92 (m, 1H), 4.32 – 4.11 (comp, 3H), 3.43 – 3.24 (m, 2H), 2.47 (s, 3H), 1.04 (t, *J* = 7.1 Hz, 3H); ^{13}C NMR (75 MHz, $CDCl_3$) (δ, ppm) 172.2, 158.8, 142.9, 139.9, 139.1, 135.7, 134.5, 131.4, 129.4, 128.8, 128.5, 127.9, 127.0, 126.9, 126.7, 125.8, 125.4, 124.5, 122.7, 122.5, 104.2, 61.3, 44.3, 32.4, 22.3, 13.7. HRMS (TOF MS ESI$^+$) calculated for $C_{28}H_{24}NaO_3^+$ [M+Na]$^+$: 431.1618, found: 431.1623.

Ethyl 6-hydroxy-8-methoxy-12-phenyl-11,12-dihydrochrysene-5-carboxylate (9).

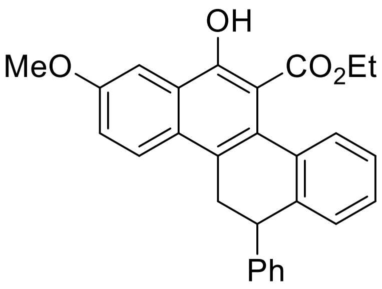

Yellow solid; m.p. 138.0 – 142.0 ˚C. 78.1 mg, 92% yield. ^{1}H NMR (300 MHz, $CDCl_3$) (δ, ppm) 10.89 (s, 1H), 7.69 (d, *J* = 9.2 Hz, 1H), 7.56 (d, *J* = 2.7 Hz, 1H), 7.21 – 7.00 (comp, 9H), 6.87 (d, *J* = 7.3 Hz, 1H), 4.22 – 4.01 (comp, 3H), 3.78 (s, 3H), 3.28 – 3.04 (m, 2H), 0.95 (t, *J* = 7.1 Hz, 3H); ^{13}C NMR (75 MHz, $CDCl_3$) (δ, ppm) 172.2, 157.8, 157.3, 142.8, 138.8, 135.6, 129.3, 129.2, 128.9, 128.8, 128.4, 127.0, 126.63, 126.60, 125.9, 125.8, 125.5, 125.0, 121.6, 105.5, 103.1, 61.4, 55.5, 44.3, 32.5, 13.7. HRMS (TOF MS ESI$^+$) calculated for $C_{28}H_{24}NaO_4^+$ $[M+Na]^+$: 447.1567, found: 447.1558.

Ethyl 14-hydroxy-8-phenyl-7,8-dihydrobenzo[*c*]chrysene-13-carboxylate (10).

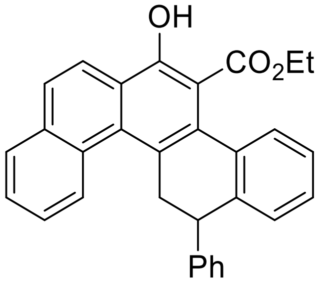

Yellow solid; m.p. 168.0 – 170.0 ˚C. 47.1 mg, 53% yield. ^{1}H NMR (400 MHz, $CDCl_3$) (δ, ppm) 12.18 (s, 1H), 8.39 (d, *J* = 9.0 Hz, 1H), 7.90 – 7.80 (comp, 2H), 7.57 – 7.51 (m, 1H), 7.39 – 7.27 (comp, 3H), 7.24 – 7.13 (comp, 5H), 7.04 – 6.96 (comp, 2H), 6.87 – 6.73 (m, 1H), 4.46 (t, *J* = 6.9 Hz, 1H), 3.93 (q, *J* = 7.2 Hz, 2H), 3.15 – 3.09 (m, 2H), 0.69 (t, *J* = 7.2 Hz, 3H); ^{13}C NMR (125 MHz, $CDCl_3$) (δ, ppm) 172.1, 158.8, 144.3, 142.1, 138.9, 138.0, 133.2, 131.8, 129.2, 129.0, 128.5, 128.3, 128.2, 127.8, 127.5, 126.61, 126.59, 126.55, 126.5, 126.0, 123.4, 121.3, 120.9, 108.5, 61.0, 44.9, 34.0, 13.1. HRMS (TOF MS ESI$^+$) calculated for $C_{31}H_{24}NaO_3^+$ $[M+Na]^+$: 467.1618, found: 467.1630.

Ethyl 6-hydroxy-4-methyl-12-phenyl-11,12-dihydrochrysene-5-carboxylate (11).

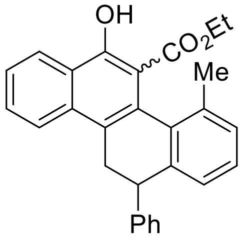

Yellow solid; m.p. 113.0 – 115.0 ˚C. 61.3 mg, 75% yield, *dr* = 3:2. ^{1}H NMR (300 MHz, $CDCl_3$) (δ, ppm) *with two isomers together*, 11.88 and 11.65 (s, 1H), 8.60 – 8.41 (comp, 1H), 8.09 – 8.02 (comp, 1H), 7.70 – 7.43 (comp, 4H), 7.31 – 7.26 (comp, 1H), 7.26 – 7.17 (comp, 2H), 7.12 – 7.00 (comp, 3H), 6.70 – 6.49 (comp, 1H), 4.45 – 4.07 (comp, 3H), 3.88 – 3.50 (comp, 1H), 3.26 – 2.99 (comp, 1H), 2.34 and 2.33 (s, 3H), 1.10 and 1.01 (t, *J* = 7.2 Hz, 3H); ^{13}C NMR (75 MHz, $CDCl_3$) (δ, ppm) *with two isomers together*, 172.0 and 171.8, 158.2 and 157.9, 143.0 and 142.5, 142.0 and 139.3, 136.7 and 135.7, 135.8 and 135.0, 134.8 and 134.0, 130.8 and 130.2, *129.7 – 123.2 included 25 peaks*: (129.7, 129.5, 129.4, 129.1, 128.8, 128.6, 128.1, 127.8, 127.0, 126.9, 126.5, 125.90, 125.86, 125.81, 125.77, 125.5, 124.6, 124.5, 124.2, 124.1, 123.5, 123.4, 123.3), 106.9 and 106.7, 61.6 and 61.5, 46.9 and 44.0, 32.7 and 32.1, 20.84, 13.6 and 13.4. HRMS (TOF MS ESI$^+$) calculated for $C_{28}H_{24}NaO_3^+$ $[M+Na]^+$: 431.1618, found: 431.1611.

Ethyl 6-hydroxy-3-methyl-12-phenyl-11,12-dihydrochrysene-5-carboxylate (**12a**) &
Ethyl 6-hydroxy-1-methyl-12-phenyl-11,12-dihydrochrysene-5-carboxylate (**12b**).

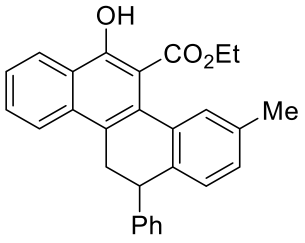

12a

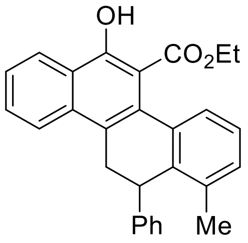

12b

Yellow oil, combined 77.6 mg in total with 95% yield, **12a**:**12b** = 63:37. **12a**: ^{1}H NMR (400 MHz, $CDCl_3$) (δ, ppm) 11.03 (s, 1H), 8.35 (d, *J* = 8.3 Hz, 1H), 7.89 – 7.82 (m, 1H), 7.51 – 7.42 (comp, 2H), 7.40 – 7.31 (m, 1H), 7.23 – 6.92 (comp, 6H), 6.84 (d, *J* = 7.5 Hz, 1H) 4.28 – 4.07 (comp, 3H), 3.32 – 3.22 (m, 2H) 2.28 (s, 3H), 1.04 – 0.97 (m, 3H); **12b**: ^{1}H NMR (400 MHz, $CDCl_3$) (δ, ppm) 10.86 (s, 1H), 8.29 (d, *J* = 8.3 Hz, 1H), 7.89 – 7.82 (m, 1H), 7.40 – 7.31 (comp, 2H), 7.23 – 6.92 (comp, 8H), 4.51 (d, *J* = 5.9 Hz, 1H), 4.24 – 4.07 (m, 2H), 3.66 (d, *J* = 16.3 Hz, 1H), 3.09 (dd, *J* = 16.2, 6.2

Hz, 1H), 2.23 (s, 3H), 1.04 – 0.97 (m, 3H); *Combined two isomers*: ^{13}C NMR (100 MHz, $CDCl_3$) (δ, ppm) 172.2 and 172.1, 158.5 and 158.2, 143.00 and 141.9,(34 carbon signals were crossed from 123.1 to 136.4) 136.4, 136.24, 136.22, 136.1, 135.4, 135.1, 135.0, 134.4, 134.2, 131.9, 131.3, 130.1, 129.6, 129.4, 129.0, 128.7, 128.4, 128.2, 128.0, 127.7, 127.5, 126.9, 126.6, 126.2, 125.7, 125.5, 124.5, 124.44, 124.37, 124.3, 124.0, 123.2, 123.1, 105.2 and 104.9, 61.3 and 61.2, 43.9 and 38.9, 32.5 and 32.4, 21.3 and 19.4, 13.69 and 13.66. HRMS (TOF MS ESI$^+$) calculated for $C_{28}H_{24}NaO_3^+$ $[M+Na]^+$: 431.1618, found: 431.1611.

Ethyl 6-hydroxy-2-methyl-12-phenyl-11,12-dihydrochrysene-5-carboxylate (13).

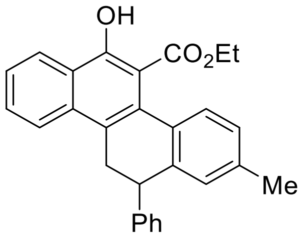

Yellow solid; m.p. 143.0 – 145.0 ˚C. 68.6 mg, 84% yield. ^{1}H NMR (400 MHz, $CDCl_3$) (δ, ppm) 11.05 (s, 1H), 8.48 – 8.39 (m, 1H), 7.97 (d, J = 8.4 Hz, 1H), 7.62 – 7.56 (m, 1H), 7.52 – 7.46 (m, 1H), 7.36 – 7.32 (comp, 2H), 7.31 – 7.26 (comp, 2H), 7.25 – 7.19 (m, 1H), 7.16 – 7.07 (comp, 2H), 6.88 (s, 1H), 4.37 – 4.20 (comp, 3H), 3.47 – 3.34 (m, 2H), 2.34 (s, 3H), 1.12 (t, J = 7.1 Hz, 3H). ^{13}C NMR (100 MHz, $CDCl_3$) (δ, ppm) 172.2, 158.4, 143.0, 138.8, 136.9, 134.3, 132.9, 131.3, 129.6, 129.4, 128.7, 128.4, 127.8, 126.7, 126.6, 125.6, 125.2, 124.6, 124.3, 123.2, 105.0, 61.4, 44.2, 32.5, 21.4, 13.8. HRMS (TOF MS ESI$^+$) calculated for $C_{28}H_{24}NaO_3^+$ $[M+Na]^+$: 431.1618, found: 431.1613.

Ethyl 12-(2-chlorophenyl)-6-hydroxy-2-methoxy-11,12-dihydrochrysene-5-carboxylate (14).

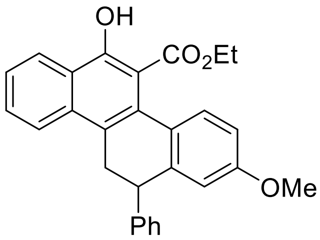

Yellow solid; m.p. 174.0 – 176.0 ˚C. 76.4 mg, 90% yield. ^{1}H NMR (400 MHz, $CDCl_3$) (δ, ppm) 11.10 (s, 1H), 8.43 (d, J = 8.2 Hz, 1H), 7.95 (d, J = 8.4 Hz, 1H), 7.61 – 7.55 (m, 1H), 7.50 – 7.44 (m, 1H), 7.38 – 7.33 (comp, 2H), 7.33 – 7.27 (comp, 2H), 7.26 – 7.21 (m, 1H), 7.17 (d, J = 8.5 Hz, 1H), 6.87 – 6.80 (m, 1H), 6.62 (s, 1H), 4.38 – 4.22 (comp, 3H), 3.79 (s, 3H), 3.47 – 3.29 (m, 2H), 1.15 (t, J = 7.1 Hz, 3H) ^{13}C NMR (100 MHz, $CDCl_3$) (δ, ppm) 172.2, 158.8, 158.5, 142.7, 140.8, 134.3, 131.0, 130.7, 129.6, 128.71, 128.65, 128.4, 126.7, 125.4, 124.5, 124.0, 123.0, 112.5, 111.4, 104.9, 61.4, 55.3, 44.6, 32.3, 13.9. HRMS (TOF MS ESI$^+$) calculated for $C_{28}H_{24}NaO_4^+$ $[M+Na]^+$: 447.1567, found: 447.1588.

Ethyl 2-fluoro-6-hydroxy-12-phenyl-11,12-dihydrochrysene-5-carboxylate (15).

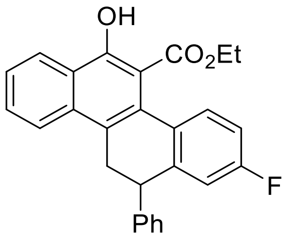

Yellow solid; m.p. 120.0 – 122.0 ˚C. 33.8 mg, 41% yield. ^{1}H NMR (400 MHz, $CDCl_3$) (δ, ppm) 11.16 (s, 1H), 8.43 (d, J = 7.7 Hz, 1H), 7.96 (d, J = 8.4 Hz, 1H), 7.62 – 7.58 (m, 1H), 7.53 – 7.48 (m, 1H), 7.37 – 7.30 (comp, 4H), 7.23 – 7.12 (comp, 2H), 6.97 – 6.92 (m, 1H), 6.76 – 6.66 (m, 1H), 4.36 – 4.16 (comp, 3H), 3.46 – 3.29 (comp, 2H), 1.11 (t, J = 7.1 Hz, 3H) ^{13}C NMR (100 MHz, $CDCl_3$) (δ, ppm) 172.0, 161.9 (d, J = 246.9 Hz), 158.9, 142.0, 134.2, 131.7 (d, J = 3.2 Hz), 130.9 (d, J = 8.1 Hz), 130.3, 129.9, 128.76, 128.67, 127.0, 125.9, 124.7, 124.4, 123.2, 113.9 (d, J = 22.2 Hz), 112.7 (d, J = 21.7 Hz), 104.7, 61.5, 44.5, 32.1, 13.8; ^{19}F NMR (376 MHz, $CDCl_3$) (δ, ppm) -114.89. HRMS (TOF MS ESI$^+$) calculated for $C_{27}H_{21}FNaO_3^+$ $[M+Na]^+$: 435.1367, found: 435.1379.

Ethyl 2-chloro-6-hydroxy-12-phenyl-11,12-dihydrochrysene-5-carboxylate (16).

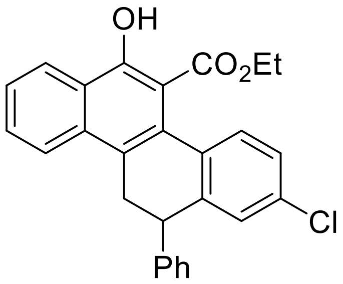

Yellow solid; m.p. 176.0 – 178.0 ˚C. 46.3 mg, 54% yield. ^{1}H NMR (300 MHz, $CDCl_3$) (δ, ppm) 11.18 (s, 1H), 8.49 – 8.37 (m, 1H), 7.96 (d, J = 8.4 Hz, 1H), 7.64 – 7.58 (m, 1H), 7.54 – 7.47 (m, 1H), 7.34 – 7.29 (comp, 4H), 7.26 – 7.20 (comp, 2H), 7.15 – 7.11 (m, 1H), 7.03 – 6.96 (m, 1H), 4.40 – 4.16 (comp, 3H), 3.46 – 3.29 (m, 2H), 1.12 (t, J = 7.1 Hz, 3H); ^{13}C NMR (100 MHz, $CDCl_3$) (δ, ppm) 171.8, 159.0, 141.9, 141.0, 134.2, 132.7, 130.7, 130.2, 129.9, 128.7, 127.1, 127.0, 126.1, 125.9, 124.7, 124.6,

123.3, 104.6, 61.6, 44.3, 32.2, 13.8. HRMS (TOF MS ESI+) calculated for $C_{27}H_{21}ClNaO_3^+$ $[M+Na]^+$: 451.1071, found: 451.1080.

Ethyl 14-hydroxy-6-phenyl-5,6-dihydrobenzo[*c*]tetraphene-13-carboxylate (17).

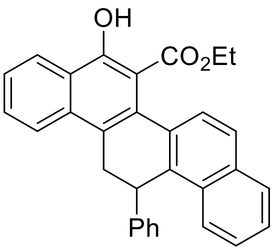

Yellow solid; m.p. 182.0 – 184.0 ˚C. 75.6 mg, 85% yield. ^{1}H NMR (400 MHz, $CDCl_3$) (δ, ppm) 11.17 (s, 1H), 8.51 – 8.30 (m, 1H), 8.11 – 8.03 (m, 1H), 8.00 (d, *J* = 8.5 Hz, 1H), 7.93 – 7.89 (m, 1H), 7.82 (d, *J* = 8.6 Hz, 1H), 7.64 – 7.57 (m, 1H), 7.53 – 7.48 (comp, 3H), 7.46 (s, 1H), 7.37 – 7.32 (comp, 2H), 7.15 – 7.05 (comp, 3H), 5.19 (d, *J* = 6.1 Hz, 1H), 4.40 – 4.31 (m, 1H), 4.22 – 4.14 (m, 1H), 3.88 (d, *J* = 16.4, 1H), 3.29 (dd, *J* = 16.4, 6.1 Hz, 1H), 0.99 (t, *J* = 7.1 Hz, 3H). ^{13}C NMR (100 MHz, $CDCl_3$) (δ, ppm) 171.9, 158.7, 142.0, 134.5, 133.9, 133.2, 132.9, 132.0, 131.3, 129.6, 128.4, 128.21, 128.15, 127.9, 126.5, 126.3, 125.80, 125.75, 125.7, 124. 6, 124.5, 124.1, 123.9, 123.2, 105.0, 61.4, 38.5, 32.5, 13.7. HRMS (TOF MS ESI+) calculated for $C_{31}H_{24}NaO_3^+$ $[M+Na]^+$: 467.1618, found: 467.1604.

Ethyl 8-hydroxy-14-phenyl-13,14-dihydrobenzo[c]chrysene-7-carboxylate (18).

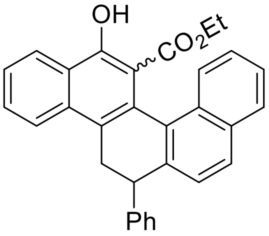

The desired product was isolated as a mixture of two diastereoisomers. Yellow solid; m.p. 165.0 – 170.0 ˚C. 70.2 mg, 79% yield, *dr* = 1:1. *One diastereoisomer*: ^{1}H NMR (400 MHz, $CDCl_3$) (δ, ppm) 11.66 (s, 1H), 8.55 (d, *J* = 8.4 Hz, 1H), 8.09 – 8.00 (comp, 2H), 7.82 – 7.77 (m, 1H), 7.67 – 7.62 (comp, 2H), 7.59 – 7.55 (m, 1H), 7.50 – 7.37 (comp, 7H), 6.92 (d, *J* = 8.4 Hz, 1H), 4.24 (dd, *J* = 14.9, 4.1 Hz, 1H), 3.68 – 3.55 (comp, 2H), 3.37 – 3.27 (m, 1H), 3.06 (t, *J* = 15.0 Hz, 1H), 0.47 (t, *J* = 7.0 Hz, 3H); ^{13}C NMR (100 MHz, $CDCl_3$) (δ, ppm) 171.7, 157.9, 143.0, 138.9, 134.1, 132.8, 132.4, 131.9, 129.7, 129.1, 129.0, 128.6, 128.5, 127.2, 127.0, 126.5, 125.9, 125.0, 124.7, 124.6, 124.4, 123.8, 123.7, 107.6, 61.2, 46.7, 33.3, 12.6. *The other diastereoisomer*: ^{1}H NMR (400 MHz, $CDCl_3$) (δ, ppm) 11.43 (s, 1H), 8.43 (d, *J* = 8.3 Hz, 1H), 8.13 – 8.05 (comp, 2H), 7.90 – 7.86 (m, 1H), 7.82 – 7.80 (m, 1H), 7.60 – 7.57 (m, 1H), 7.51 – 7.44 (comp, 4H), 7.22 – 7.17 (commp, 2H), 7.08 – 6.98 (comp, 3H), 4.50 (d, *J* = 4.9 Hz, 1H), 3.88 – 3.78 (m, 2H), 3.32 – 3.27 (m, 1H), 3.24 – 3.16 (m, 1H), 0.43 (t, *J* = 7.2 Hz, 3H); *Combined two isomers*: ^{13}C NMR (100 MHz, $CDCl_3$) (δ, ppm) 171.5, 157.5, 142.0, 136.7, 134.9, 134.0, 133.0, 132.1, 129.5, 129.0, 128.6, 128.2, 128.0, 127.7, 126.8, 126.6, 126.1, 125.8, 125.7, 125.1, 124.5, 124.3, 123.7, 123.5, 107.5, 61.1, 44.1, 32.5, 12.9. HRMS (TOF MS ESI+) calculated for $C_{31}H_{24}NaO_3^+$ $[M+Na]^+$: 467.1618, found: 467.1613.

Ethyl 5-hydroxy-11-phenyl-10,11-dihydrophenanthro[1,2-*b*]thiophene-4-carboxy late (19).

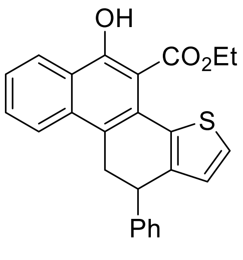

Yellow solid; m.p. 161.0 – 163.0 ˚C. 69.7 mg, 87% yield. ^{1}H NMR (400 MHz, $CDCl_3$) (δ, ppm) 10.94 (s, 1H), 8.47 – 8.39 (m, 1H), 7.97 (d, *J* = 8.5 Hz, 1H), 7.64 – 7.56 (m, 1H), 7.53 – 7.46 (m, 1H), 7.46 – 7.41 (comp, 2H), 7.40 – 7.33 (comp, 2H), 7.33 – 7.25 (comp, 2H), 6.68 (d, *J* = 5.1 Hz, 1H), 4.54 – 4.37 (comp, 3H), 3.54 (dd, *J* = 16.2, 6.4 Hz, 1H), 3.32 (dd, *J* = 16.2, 10.6 Hz, 1H), 1.31 (t, *J* = 7.1 Hz, 3H). ^{13}C NMR (100 MHz, $CDCl_3$) (δ, ppm) 171.0, 158.1, 143.3, 139.8, 136.8, 134.5, 129.8, 128.6, 128.4, 126.9, 126.8, 126.0, 125.6, 124.8, 124.7, 124.1, 124.0, 123.5, 104.9, 61.9, 41.2, 33.9, 13.9. HRMS (TOF MS ESI+) calculated for $C_{25}H_{20}NaO_3S^+$ $[M+Na]^+$: 423.1025, found 423.1020.

Ethyl 5-hydroxy-11-phenyl-10,11-dihydrophenanthro[2,1-*b*]thiophene-4-carboxy late (20).

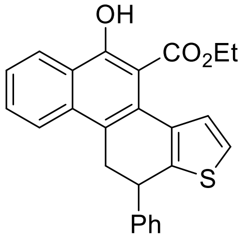

Yellow solid; m.p. 125.0 – 127.0 ˚C. 72.9 mg, 91% yield. ^{1}H NMR (400 MHz, $CDCl_3$) (δ, ppm) 11.06 (s, 1H), 8.42 (d, *J* = 7.9 Hz, 1H), 7.94 (d, *J* = 8.5 Hz, 1H), 7.61 – 7.54 (m, 1H), 7.52 – 7.40 (comp, 3H), 7.39 – 7.27 (comp, 3H), 7.08 (d, *J* = 5.2 Hz, 1H), 6.99 (d, *J* = 5.2 Hz, 1H), 4.48 (dd, *J* = 10.6, 6.1 Hz, 1H), 4.43 – 4.29 (m, 2H), 3.56 (dd, *J* = 16.0, 6.1 Hz, 1H), 3.33 (dd, *J* = 16.0, 10.7 Hz, 1H), 1.24 (t, *J* = 7.1 Hz, 3H). ^{13}C NMR (100 MHz, $CDCl_3$) (δ, ppm) 171.7, 158.3, 142.8, 140.7, 136.4, 134.7, 129.7, 128.7, 128.3, 128.1, 127.4, 125.4, 124.6, 123.9, 123. 6, 123. 3, 121.1, 105.0, 61.7, 40.9, 34.2, 13.9. HRMS (TOF MS ESI$^+$) calculated for $C_{25}H_{20}NaO_3S^+$ [M+Na]$^+$: 423.1025, found: 423.1018.

Ethyl 6-hydroxy-12-phenyl-1,2,3,11,12,12a-hexahydrochrysene-5-carboxylate (**22b**) &
Ethyl 6-hydroxy-12-phenyl-1,2,3,4,11,12-hexahydrochrysene-5-car boxylate (**22a**).

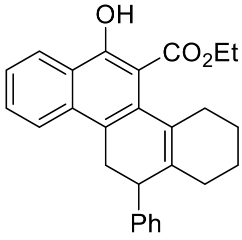

21a

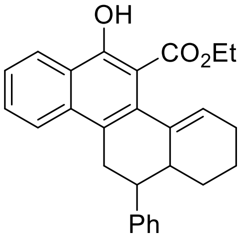

21b

Yellow solid; combined 66.2 mg in total with 83% yield, **21a**:**21b** (*dr* = 1.7:1) = 40:60. *Combined three isomers*: ^{1}H NMR (300 MHz, $CDCl_3$) (δ, ppm) 10.99 – 10.21 (comp, 1H), 8.38 – 8.04 (comp, 1H), 7.86 – 7.49 (comp, 1H), 7.43 – 6.78 (comp, 7H), 5.63 – 0.64 (comp, 16H). ^{13}C NMR (100 MHz, $CDCl_3$) (δ, ppm) 172.7, 172.4, 171.6, 157.6, 156.99, 156.98, 145.5, 144.0, 142.2, 137.6, 137.0, 134.9, 134.7, 134.3, 134.2, 133.8, 133.6, 133.5, 132.4, 129.29, 129.28, 129.2, 128.8, 128.7, 128.5, 128.4, 128.14, 128.06, 127.9, 126.63, 126.57, 126.3, 126.2, 125.3, 125.1, 124.8, 124.4, 124.3, 124.2, 124.1, 123.9, 123.4, 123.3, 123.23, 123.18, 123.0, 122.5, 106.3, 106.2, 105.3, 61.6, 61.2, 61.1, 49.3, 45.2, 44.0, 38.9, 35.3, 32.6, 30.3, 29.2, 29.0, 28.3, 27.0, 26.3, 26.0, 23.9, 23.4, 22.7, 21.8, 20.1, 14.2, 14.14, 14.12. HRMS (TOF MS ESI$^+$) calculated for $C_{27}H_{26}NaO_3^+$ [M+Na]$^+$: 421.1774, found: 421.1778.

Ethyl 12-(2-bromophenyl)-6-hydroxy-2-methoxy-11,12-dihydrochrysene-5-carboxylate (22).

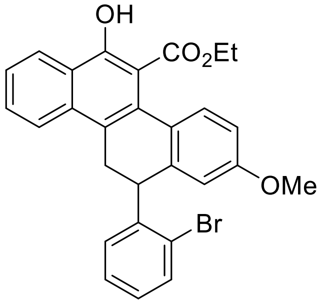

Yellow solid; m.p. 182.0 – 184.0 ˚C. 91.6 mg, 91% yield. ^{1}H NMR (400 MHz, $CDCl_3$) (δ, ppm) 11.02 (s, 1H), 8.47 – 8.34 (m, 1H), 7.94 (d, *J* = 8.5 Hz, 1H), 7.64 – 7.53 (comp, 2H), 7.50 – 7.41 (m, 1H), 7.26 – 7.21 (m, 1H), 7.20 (d, *J* = 8.5 Hz, 1H), 7.09 – 6.98 (comp, 2H), 6.90 – 6.83 (m, 1H), 6.68 (d, *J* = 2.5 Hz, 1H), 4.78 (t, *J* = 5.4 Hz, 1H), 4.44 – 4.33 (m, 1H), 4.31 – 4.21 (m, 1H), 3.81 (s, 3H), 3.67 (dd, *J* = 16.2, 5.4 Hz, 1H), 3.27 (dd, *J* = 16.2, 6.0 Hz, 1H), 1.16 (t, *J* = 7.1 Hz, 3H). ^{13}C NMR (100 MHz, $CDCl_3$) (δ, ppm) 172.1, 159.0, 158.5, 141.1, 139.3, 134.4, 133.1, 131.0, 130.7, 129.6, 129.3, 128.1, 127.2, 125.5, 124.8, 124.5, 124.1, 123.7, 123.1, 112.6, 112.3, 104.8, 61.4, 55.4, 43.6, 30.4, 13.9. HRMS (TOF MS ESI$^+$) calculated for $C_{28}H_{23}BrNaO_4^+$ [M+Na]$^+$: 525.0672, found: 525.0696.

Ethyl 12-(2-chlorophenyl)-6-hydroxy-2-methoxy-11,12-dihydrochrysene-5-carboxylate (23).

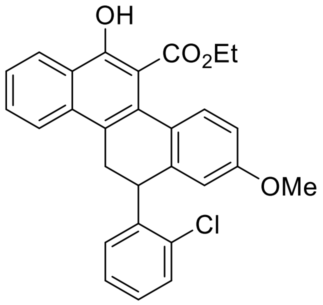

Yellow solid; m.p. 193.0 – 195.0 ˚C. 71.6 mg, 78% yield. ^{1}H NMR (400 MHz, $CDCl_3$) (δ, ppm) 11.02 (s, 1H), 8.41 – 8.36 (m, 1H), 7.93 (d, *J* = 8.5 Hz, 1H), 7.59 – 7.54 (m, 1H), 7.47 – 7.43 (m, 1H), 7.40 – 7.36 (m, 1H), 7.24 – 7.17 (m, 2H), 7.12 – 7.06 (m, 1H), 7.00 (t, *J* = 7.2 Hz, 1H), 6.89 – 6.83 (m, 1H), 6.68 (d, *J* = 2.5 Hz, 1H), 4.81 (t, *J* = 5.6 Hz, 1H), 4.44 – 4.20 (m, 2H), 3.81 (s, 3H), 3.66 (dd, *J* = 16.2, 5.5 Hz, 1H), 3.27 (dd, *J* = 16.2, 6.0 Hz, 1H), 1.16 (t, *J* = 7.1 Hz, 3H). ^{13}C NMR (100 MHz, $CDCl_3$) (δ, ppm) 172.1, 159.0, 158.5, 139.5, 139.3, 134.5, 134.1, 131.0, 130.8, 130.6, 129.8, 129.6, 129.4, 127.8, 126.5, 125.5, 124.5, 124.1, 123.8, 123.1, 112.5, 112.2, 104.8, 61.4, 55.4, 41.1, 30.2,

13.9. HRMS (TOF MS ESI+) calculated for $C_{28}H_{23}ClNaO_4^+$ [M+Na]+: 481.1177, found: 481.1186.

Ethyl 12-(3-chlorophenyl)-6-hydroxy-2-methoxy-11,12-dihydrochrysene-5-carboxylate (24).

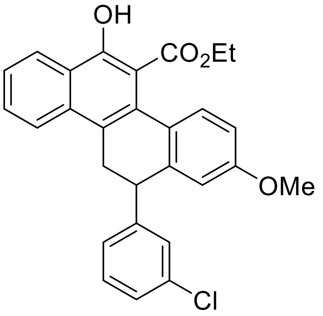

Yellow solid; m.p. 178.0 – 180.0 ˚C. 65.2 mg, 71% yield. ^{1}H NMR (400 MHz, $CDCl_3$) (δ, ppm) 11.10 (s, 1H), 8.43 – 8.38 (m, 1H), 7.92 (d, *J* = 8.5 Hz, 1H), 7.62 – 7.54 (m, 1H), 7.53 – 7.42 (m, 1H), 7.27 – 7.21 (comp, 2H), 7.20 – 7.13 (comp, 3H), 6.91 – 6.78 (m, 1H), 6.65 (d, *J* = 2.4 Hz, 1H), 4.34 – 4.25 (comp, 3H), 3.81 (s, 3H), 3.43 (dd, *J* = 16.0, 6.5 Hz, 1H), 3.32 (dd, *J* = 16.0, 5.6 Hz, 1H), 1.16 (t, *J* = 7.1 Hz, 3H). ^{13}C NMR (100 MHz, $CDCl_3$) (δ, ppm) 172.1, 158.9, 158.6, 144.9, 139.5, 134.3, 134.2, 131.0, 130.9, 129.7, 129.6, 128.8, 128.7, 126.8, 126.7, 125.5, 124.6, 124.1, 123.7, 123.0, 112.6, 111.9, 104.8, 61.6, 55.4, 44.1, 32.3, 13.9. HRMS (TOF MS ESI+) calculated for $C_{28}H_{23}ClNaO_4^+$ [M+Na]+: 481.1177, found: 481.1169.

Ethyl 12-(4-chlorophenyl)-6-hydroxy-2-methoxy-11,12-dihydrochrysene-5-carboxylate (25).

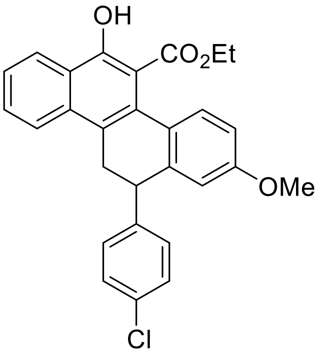

Yellow solid; m.p. 180.0 – 182.0 ˚C. 80.8 mg, 88% yield. ^{1}H NMR (400 MHz, $CDCl_3$) (δ, ppm) 11.08 (s, 1H), 8.42 (d, *J* = 8.2 Hz, 1H), 7.91 (d, *J* = 8.4 Hz, 1H), 7.61 – 7.53 (m, 1H), 7.51 – 7.41 (m, 1H), 7.27 – 7.21 (comp, 4H), 7.17 (d, *J* = 8.6 Hz, 1H), 6.89 – 6.81 (m, 1H), 6.62 (d, *J* = 2.0 Hz, 1H), 4.39 – 4.22 (comp, 3H), 3.80 (s, 3H), 3.40 – 3.28 (m, 2H), 1.14 (t, *J* = 7.1 Hz, 3H). ^{13}C NMR (100 MHz, $CDCl_3$) (δ, ppm) 172.0, 158.9, 158.5, 141.2, 140.0, 134.2, 132.3, 131.0, 130.8, 130.0, 129.7, 128.6, 128.5, 125.5, 124.6, 124.0, 123.9, 122.9, 112.5, 111.6, 104.8, 61.4, 55.4, 43.9, 32.2, 13.9. HRMS (TOF MS ESI+) calculated for $C_{28}H_{23}ClNaO_4^+$ [M+Na]+: 481.1177, found: 481.1163.

Ethyl 12-(4-fluorophenyl)-6-hydroxy-2-methoxy-11,12-dihydrochrysene-5-carboxylate (26).

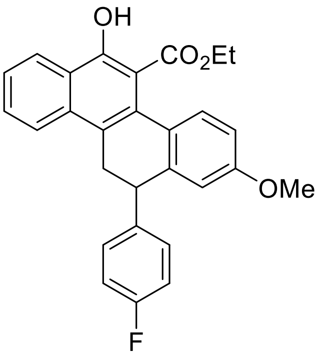

Yellow solid; m.p. 109.0 – 111.0 ˚C. 54.0 mg, 61% yield. ^{1}H NMR (400 MHz, $CDCl_3$) (δ, ppm) 11.03 (s, 1H), 8.40 (d, *J* = 7.5 Hz, 1H), 7.94 (d, *J* = 8.5 Hz, 1H), 7.61 – 7.56 (m, 1H), 7.49 – 7.44 (m, 1H), 7.30 – 7.25 (comp, 2H), 7.14 (d, *J* = 8.5 Hz, 1H), 6.98 – 6.91 (comp, 2H), 6.86 – 6.79 (m, 1H), 6.58 (d, *J* = 2.3 Hz, 1H), 4.36 – 4.18 (comp, 3H), 3.78 (s, 3H), 3.48 – 3.16 (m, 2H), 1.11 (t, *J* = 7.1 Hz, 3H). ^{13}C NMR (100 MHz, $CDCl_3$) (δ, ppm) 172.1, 158.7 (d, *J* = 35.0 Hz), 140.5, 138.3 (d, *J* = 3.1 Hz), 134.3, 131.0, 130.8, 130.1 (d, *J* = 8.0 Hz), 129.7, 128.6, 125.5, 124.6, 124.2, 124.1, 123.0, 115.4, 115.1, 112.5, 111.6, 104.9, 61.5, 55.4, 43.9, 32.5, 13.9. ^{19}F NMR (376 MHz, $CDCl_3$) (δ, ppm) -116.66. HRMS (TOF MS ESI+) calculated for $C_{28}H_{23}FNaO_4^+$ [M+Na]+: 465.1473, found: 465.1481.

Ethyl 6-hydroxy-2-methoxy-12-(4-(trifluoromethyl)phenyl)-11,12-dihydrochrysene-5-carboxylate (27).

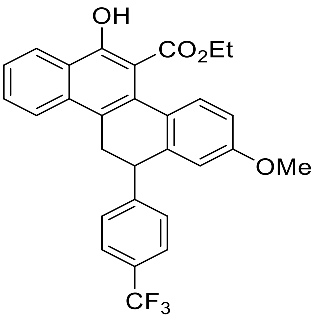

Yellow solid; m.p. 138.0 – 140.0 ˚C. 85.7 mg, 87% yield. ^{1}H NMR (400 MHz, $CDCl_3$) (δ, ppm) 11.05 (s, 1H), 8.45 – 8.36 (m, 1H), 7.92 (d, *J* = 8.5 Hz, 1H), 7.62 – 7.56 (m, 1H), 7.51 – 7.40 (comp, 5H), 7.18 (d, *J* = 8.5 Hz, 1H), 6.89 – 6.82 (m, 1H), 6.61 (d, *J* = 2.2 Hz, 1H), 4.38 – 4.20 (comp, 3H), 3.80 (s, 3H), 3.48 – 3.32 (m, 2H), 1.14 (t, *J* = 7.1 Hz, 3H). ^{13}C NMR (100 MHz, $CDCl_3$) (δ, ppm) 172.0, 159.0, 158.6, 146.9, 139.5, 134.3, 131.0, 130.9, 129.8, 129.0, 128.9 (q, *J* = 32.4 Hz), 128.7, 125.6, 125.3 (q, *J* = 3.6 Hz), 124.7, 124.3 (d, *J* = 272.0 Hz), 124.1, 122.9, 112.7, 111.8, 104.8, 61.5, 55.4, 44.3, 32.3, 13.9. ^{19}F NMR (376 MHz, $CDCl_3$) (δ, ppm) -62.38. HRMS (TOF MS ESI+) calculated

for $C_{29}H_{23}F_3NaO_4^+$ $[M+Na]^+$: 515.1441, found: 515.1419.

Ethyl 6-hydroxy-2-methoxy-12-(*p*-tolyl)-11,12-dihydrochrysene-5-carboxylate (28).

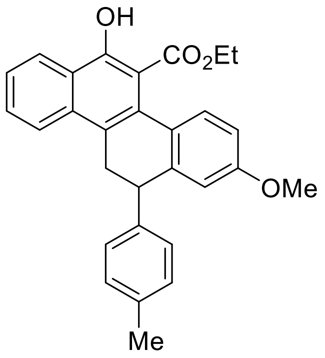

Yellow solid; m.p. 178.0 – 180.0 ˚C.71.0 mg, 81% yield. ^{1}H NMR (400 MHz, $CDCl_3$) (δ, ppm) 11.08 (s, 1H), 8.43 – 8.39 (m, 1H), 7.93 (d, *J* = 8.4 Hz, 1H), 7.59 – 7.54 (m, 1H), 7.48 – 7.43 (m, 1H), 7.24 – 7.21 (comp, 2H), 7.16 – 7.13 (m, 1H), 7.11 – 7.07 (comp, 2H), 6.83 – 6.79 (m, 1H), 6.63 – 6.59 (m, 1H), 4.35 – 4.23 (comp, 3H), 3.77 (s, 3H), 3.40 – 3.29 (m, 2H), 2.31 (s, 3H), 1.14 (t, *J* = 7.1 Hz, 3H); ^{13}C NMR (100 MHz, $CDCl_3$) (δ, ppm) 172.2, 158.8, 158.5, 141.0, 139.6, 136.2, 134.3, 131.1, 130.7, 129.6, 129.2, 128.7, 128.6, 125.4, 124.6, 124.5, 124.0, 123.1, 112.5, 111.4, 104.9, 61.4, 55.3, 44.2, 32.4, 21.1, 13.9. HRMS (TOF MS ESI$^+$) calculated for $C_{29}H_{26}NaO_4^+$ $[M+Na]^+$: 461.1723, found: 461.1686.

Ethyl 12-(4-(*tert*-butyl)phenyl)-6-hydroxy-2-methoxy-11,12-dihydrochrysene-5- carboxylate (29).

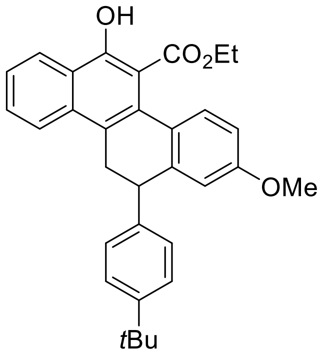

Yellow solid; m.p. 232.0 – 234.0 ˚C. 81.7 mg, 85% yield. ^{1}H NMR (400 MHz, $CDCl_3$) (δ, ppm) 11.10 (s, 1H), 8.43 (d, *J* = 8.3 Hz, 1H), 7.95 (d, *J* = 8.4 Hz, 1H), 7.61 – 7.54 (m, 1H), 7.49 – 7.44 (m, 1H), 7.34 – 7.25 (comp, 4H), 7.16 (d, *J* = 8.5 Hz, 1H), 6.88 – 6.77 (m, 1H), 6.63 (s, 1H), 4.37 – 4.23 (comp, 3H), 3.79 (s, 3H), 3.43 – 3.28 (m, 2H), 1.33 (s, 9H), 1.16 (t, *J* = 6.7 Hz, 3H). ^{13}C NMR (100 MHz, $CDCl_3$) (δ, ppm) 172.2, 158.8, 158.5, 149.4, 141.1, 139.5, 134.4, 131.1, 130.7, 129.6, 128.7, 128.4, 125.4, 124.8, 124.5, 124.0, 123.1, 112.7, 111.2, 104.9, 61.4, 55.4, 44.2, 34.5, 32.4, 31.5, 13.9. HRMS (TOF MS ESI$^+$) calculated for $C_{32}H_{32}NaO_4^+$ $[M+Na]^+$: 503.2193, found: 503.2171.

Ethyl 6-hydroxy-2-methoxy-12-(4-methoxyphenyl)-11,12-dihydrochrysene-5-carboxylate (30).

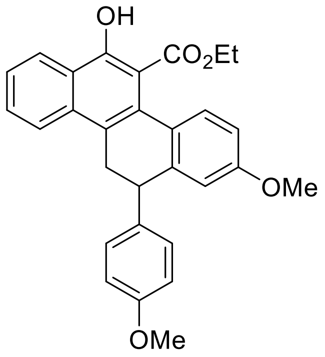

Yellow solid; m.p. 167.0 – 169.0 ˚C. 49.1 mg, 54% yield. ^{1}H NMR (400 MHz, $CDCl_3$) (δ, ppm) 11.03 (s, 1H), 8.39 (d, *J* = 7.9 Hz, 1H), 7.95 (d, *J* = 8.0 Hz, 1H), 7.57 (t, *J* = 7.6 Hz, 1H), 7.46 (t, *J* = 7.5 Hz, 1H), 7.26 – 7.22 (comp, 3H), 7.13 (d, *J* = 8.4 Hz, 1H), 6.84 – 6.77 (comp, 3H), 6.57 (s, 1H), 4.31 – 4.20 (comp, 3H), 3.77 (comp, 6H), 3.38 – 3.28 (m, 2H), 1.11 (t, *J* = 7.1 Hz, 3H). ^{13}C NMR (100 MHz, $CDCl_3$) (δ, ppm) 172.2, 158.8, 158.5, 158.4, 134.7, 134.4, 131.1, 130.7, 129.7, 129.6, 128.6, 125.4, 124.7, 124.6, 124.1, 123.1, 113.9, 112.4, 111.4, 104.9, 61.4, 55.41, 55.37, 43.9, 32.5, 13.9. HRMS (TOF MS ESI$^+$) calculated for $C_{29}H_{26}NaO_5^+$ $[M+Na]^+$: 477.1672, found: 477.1675.

Ethyl 6-hydroxy-2-methoxy-12-(2-methoxyphenyl)-11,12-dihydrochrysene-5-carboxylate (31).

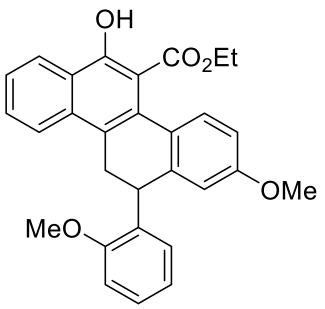

Yellow solid; m.p. 158.0 – 159.0 ˚C. 73.7 mg, 81% yield. ^{1}H NMR (300 MHz, $CDCl_3$) (δ, ppm) 11.03 (s, 1H), 8.40 (dd, *J* = 8.4, 0.9 Hz, 1H), 7.94 (d, *J* = 8.4 Hz, 1H), 7.60 – 7.52 (m, 1H), 7.48 – 7.40 (m, 1H), 7.20 – 7.07 (comp, 3H), 6.90 (d, *J* = 7.7 Hz, 1H), 6.83 (dd, *J* = 8.6, 2.7 Hz, 1H), 6.75 (t, *J* = 7.5 Hz, 1H), 6.68 (d, *J* = 2.6 Hz, 1H), 4.77 (t, *J* = 6.0 Hz, 1H), 4.42 – 4.20 (m, 2H), 3.93 (s, 3H), 3.80 (s, 3H), 3.59 (dd, *J* = 16.1, 6.2 Hz, 1H), 3.23 (dd, *J* = 16.1, 6.0 Hz, 1H), 1.16 (t, *J* = 7.1 Hz, 3H). ^{13}C NMR (75 MHz, $CDCl_3$) (δ, ppm) 172.2, 158.9, 158.3, 157.4, 140.6, 134.6, 131.0, 130.6, 130.4, 129.9, 129.4, 127.6, 125.2, 124.9, 124.5, 124.0, 123.2, 120.3, 112.4, 111.8, 110.5, 104.9, 61.4, 55.6, 55.4, 37.9, 30.4, 13.9. HRMS (EI) calculated for $C_{29}H_{26}O_5$ (*m/z*): 454.1780, found: 454.1791.

Ethyl 12-([1,1'-biphenyl]-2-yl)-6-hydroxy-2-methoxy-11,12-dihydrochrysene-5-carboxylate (32).

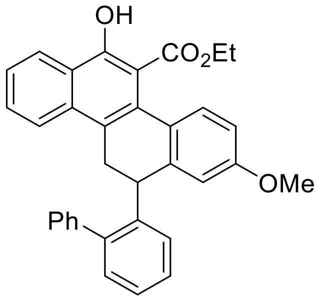

Yellow solid; m.p. 185.0 – 186.0 ˚C. 93.1 mg, 93% yield. ^{1}H NMR (300 MHz, $CDCl_3$) (δ, ppm) 11.17 (s, 1H), 8.46 (d, *J* = 8.1 Hz, 1H), 7.83 (d, *J* = 8.4 Hz, 1H), 7.59 – 7.36 (comp, 11H), 7.20 (d, *J* = 8.5 Hz, 1H), 6.89 (dd, *J* = 8.6, 2.6 Hz, 1H), 6.70 (d, *J* = 2.3 Hz, 1H), 4.70 – 4.53 (m, 1H), 4.43 – 4.27 (m, 2H), 3.83 (d, *J* = 8.3 Hz, 3H), 3.38 – 3.07 (m, 2H), 1.19 (t, *J* = 7.1 Hz, 3H); ^{13}C NMR (75 MHz, $CDCl_3$) (δ, ppm) 172.0, 158.8, 158.3, 142.7, 141.9, 141.7, 139.8, 134.0, 130.9, 130.6, 130.5, 129.4, 129.2, 128.91, 128.85, 128.2, 127.5, 127.1, 126.6, 125.2, 125.1, 124.4, 123.8, 122.9, 112.8, 110.7, 104.8, 61.2, 55.2, 40.9, 31.9, 13.7. HRMS (EI) calculated for $C_{34}H_{28}O_4$ (*m/z*): 500.1988, found: 500.1200

Ethyl 12-(2-chlorophenyl)-6-hydroxy-2-methoxy-11,12-dihydrochrysene-5-carboxylate (33).

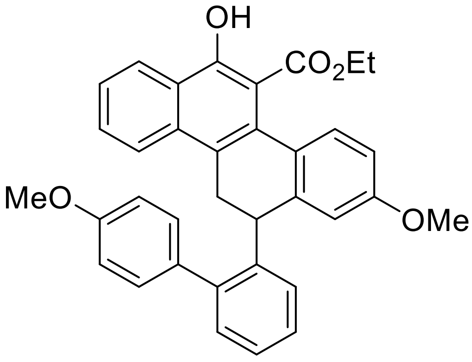

Yellow solid; m.p. 188.0 – 190.0 ˚C. 99.8 mg, 94% yield. ^{1}H NMR (400 MHz, $CDCl_3$) (δ, ppm) 11.08 (s, 1H), 8.48 – 8.37 (m, 1H), 7.84 (d, *J* = 8.4 Hz, 1H), 7.58 – 7.52 (m, 1H), 7.49 – 7.42 (comp, 2H), 7.40 – 7.28 (comp, 5H), 7.15 (d, *J* = 8.4 Hz, 1H), 7.00 – 6.87 (comp, 2H) 6.86 – 6.78 (m, 1H), 6.62 (d, *J* = 2.5 Hz, 1H), 4.60 – 4.48 (m, 1H), 4.36 – 4.23 (m, 2H), 3.88 – 3.74 (comp, 6H), 3.38 – 3.04 (m, 2H), 1.15 (t, *J* = 7.1 Hz, 3H). ^{13}C NMR (100 MHz, $CDCl_3$) (δ, ppm) 172.1, 158.80, 158.77, 158.4, 142.4, 142.1, 140.0, 134.1, 134.0, 131.0, 130.7, 130.5, 130.2, 129.5, 128.9, 127.3, 126.6, 125.3, 124.5, 123.9, 123.0, 113.7, 112.8, 110.7, 104.8, 61.2, 55.32, 55.25, 41.0, 32.0, 13.8. HRMS (TOF MS ESI$^+$) calculated for $C_{35}H_{30}NaO_5^+$ $[M+Na]^+$: 553.1985, found: 553.1985.

Ethyl 6-hydroxy-2-methoxy-12-(2-(4-methoxyphenoxy)phenyl)-11,12-dihydrochrysene-5- carboxylate (34).

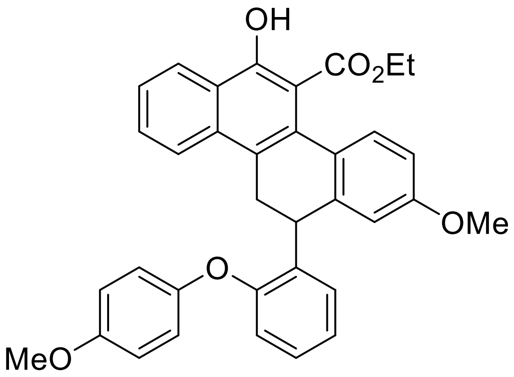

Yellow solid; m.p. 148.0 – 150.0 ˚C. 90.7 mg, 83% yield. ^{1}H NMR (400 MHz, $CDCl_3$) (δ, ppm) 11.16 (s, 1H), 8.50 (d, *J* = 7.6 Hz, 1H), 7.97 (d, *J* = 8.4 Hz, 1H), 7.65 – 7.60 (m, 1H), 7.55 – 7.50 (m, 1H), 7.32 – 7.27 (comp, 2H), 7.18 – 7.09 (comp, 3H), 7.04 – 6.99 (comp, 2H), 6.98 – 6.88 (comp, 4H), 4.95 (t, *J* = 5.3 Hz, 1H), 4.53 – 4.44 (m, 1H), 4.42 – 4.32 (m, 1H), 3.94 – 3.87 (comp, 6H), 3.87 – 3.79 (m, 1H), 3.31 (dd, *J* = 16.1, 5.8 Hz, 1H), 1.27 (t, *J* = 7.1 Hz, 3H). ^{13}C NMR (100 MHz, $CDCl_3$) (δ, ppm) 172.1, 158.9, 158.3, 155.9, 155.7, 151.1, 139.9, 134.5, 132.6, 131.0, 130.6, 130.5, 129.4, 129.3, 127.6, 125.2, 124.4, 124.2, 124.0, 123.1, 122.6, 120.0, 117.8, 115.0, 112.6, 111.9, 104.8, 61.3, 55.7, 55.3, 30.5, 13.8. HRMS (TOF MS ESI$^+$) calculated for $C_{35}H_{30}NaO_6^+$ $[M+Na]^+$: 569.1935, found: 569.1933.

Ethyl 6-hydroxy-2-methoxy-12-(2-((4-methoxyphenyl)ethynyl)phenyl)-11,12-dihydrochrysene-5-carboxylate (35).

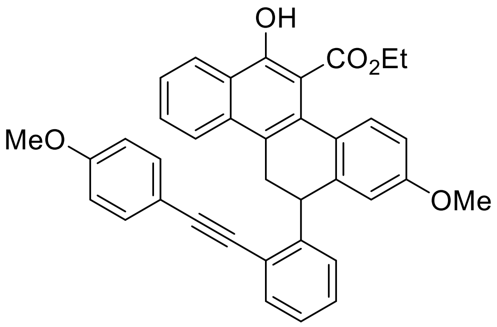

Yellow solid; m.p. 82.0 – 84.0 ˚C. 54.4 mg, 49% yield. ^{1}H NMR (400 MHz, $CDCl_3$) (δ, ppm) 11.01 (s, 1H), 8.39 (d, *J* = 8.0 Hz, 1H), 7.94 (d, *J* = 8.5 Hz, 1H), 7.58 – 7.41 (comp, 5H), 7.24 – 7.05 (comp, 4H), 6.93 – 6.82 (comp, 3H), 6.72 (d, *J* = 2.2 Hz, 1H), 4.94 (t, *J* = 5.8 Hz, 1H), 4.44 – 4.31 (m, 1H), 4.29 – 4.20 (m, 1H), 3.88 – 3.75 (comp, 7H), 3.35 (dd, *J* = 16.1, 5.8 Hz, 1H), 1.14 (t, *J* = 7.1 Hz, 3H). ^{13}C NMR (100 MHz, $CDCl_3$) (δ, ppm) 172.2, 159.9, 159.0, 158.4, 144.0, 140.0, 134.5, 133.1, 132.4, 131.1, 130.6, 129.6, 129.3, 128.9, 127.9, 126.4, 125.4, 124.5, 124.3, 124.1, 123.22, 123.20, 115.6, 114.2, 112.5, 112.1, 104.9, 94.5, 87.1, 61.4, 55.5, 55.4, 42.6, 30.8, 13.9. HRMS (TOF MS ESI$^+$) calculated for $C_{37}H_{30}NaO_5^+$ [M+Na]$^+$: 577.1985, found: 577.1962.

Ethyl 6-hydroxy-2-methoxy-12-(2-vinylphenyl)-11,12-dihydrochrysene-5-carbox ylate (36).

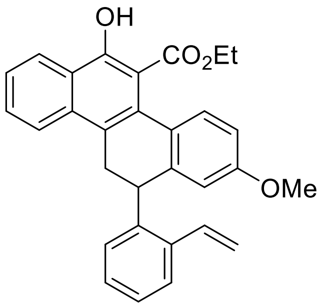

Yellow solid; m.p. 148.0 – 150.0 ˚C. 73.0 mg, 81% yield, *dr* 1:1. *Two diastereoisomers:* ^{1}H NMR (400 MHz, $CDCl_3$) (δ, ppm) 11.08 and 11.06 (two singlets, 1H), 8.45 – 8.36 (comp, 1H), 7.98 – 7.92 (comp, 1H), 7.60 – 7.55 (comp, 1H), 7.49 – 7.44 (comp, 1H), 7.38 – 7.32 (comp, 1H), 7.32 – 7.27 (comp, 2H), 7.26 – 7.23 (comp, 1H), 7.17 – 7.14 (comp, 1H), 6.83 – 6.80 (comp, 1H), 6.70 – 6.64 (comp, 1H), 6.62 – 6.59 (comp, 1H), 5.73 – 5.67 (comp, 1H), 5.23 – 5.18 (comp, 1H), 4.34 – 4.22 (comp, 3H), 3.78 and 3.77 (two singlets, 2H), 3.44 – 3.31 (comp, 2H), 1.138 and 1.135 (two triplets, *t*, *J* = 7.1 Hz, 3H). ^{13}C NMR (100 MHz, $CDCl_3$) (δ, ppm) 172.24 and 172.18, 158.8, 158.6 and 158.5, 142.95 and 142.40, 140.7 and 140.6, 137.8 and 136.1, 137.0 and 136.6, 134.32, 131.1 and 131.0, 130.8 and 130.7, 129.7, 128.9, 128.66 and 128.63, 128.3 and 126.8, 126.3, 125.4, 124.58 and 124.55, 124.06 and 124.05, 123.09 and 123.05, 114.0 and 113.5, 112.53 and 112.48, 111.54 and 111.50,104.87 and 104.86, 61.5 and 61.4, 55.40 and 55.39, 44.7 and 44.4, 32.34 and 32.33, 13.9. HRMS (TOF MS ESI$^+$) calculated for $C_{30}H_{26}NaO_4^+$ [M+Na]$^+$: 473.1723, found: 473.1717.

Ethyl 5-hydroxy-11-(thiophen-2-yl)-10,11-dihydrophenanthro[1,2-*b*]thiophene-4-carboxylate (**37**).

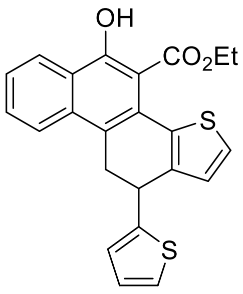

Yellow liquid. 48.8 mg, 60% yield. ^{1}H NMR (500 MHz, $CDCl_3$) (δ, ppm) 10.95 (s, 1H), 8.40 (d, *J* = 8.4 Hz, 1H), 7.98 (d, *J* = 8.4 Hz, 1H), 7.60 (t, *J* = 7.6 Hz, 1H), 7.48 (t, *J* = 7.6 Hz, 1H), 7.27 (d, *J* = 5.0 Hz, 1H), 7.17 (d, *J* = 5.0 Hz, 1H), 6.96 – 6.93 (comp, 2H), 6.80 (d, *J* = 5.0 Hz, 1H), 4.68 (dd, *J* = 9.1, 6.2 Hz, 1H), 4.46 – 4.35 (m, 2H), 3.53 (dd, *J* = 16.2, 6.2 Hz, 1H), 3.41 (dd, *J* = 16.2, 9.1 Hz, 1H), 1.26 (t, *J* = 7.1 Hz, 3H); ^{13}C NMR (125 MHz, $CDCl_3$) (δ, ppm) 171.0, 158.3, 146.9, 139.7, 136.6, 134.5, 130.0, 126.77, 126.75, 125.7, 125.6, 125.0, 125.0, 124.7, 124.2, 123.9, 123.6, 123.5, 104.7, 61.9, 36.4, 34.6, 13.9. HRMS (TOF MS ESI$^+$) calculated for $C_{23}H_{19}O_3S_2^+$ [M+H]$^+$: 407.0770, found 407.0775; For $C_{23}H_{18}NaO_3S_2^+$ [M+Na]$^+$: 429.0590, found 429.0590.

Ethyl 5'-hydroxy-10'*H*-spiro[cyclohexane-1,11'-phenanthro[2,1-*b*]thiophene]-4'-carboxylate (**38**)

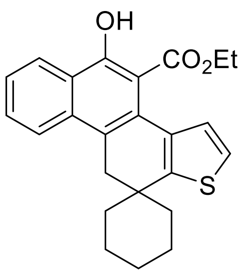

Yellow liquid. 65.2 mg, 83% yield. ^{1}H NMR (300 MHz, $CDCl_3$) (δ, ppm) 10.86 (s, 1H), 8.30 (dd, *J* = 8.4, 1.0 Hz, 1H), 7.92 (d, *J* = 8.4 Hz, 1H), 7.48 (ddd, *J* = 8.4, 6.9, 1.4 Hz, 1H), 7.34 (ddd, *J* = 8.4, 6.9, 1.0 Hz, 1H), 6.90 (d, *J* = 5.2 Hz, 1H), 6.77 (d, *J* = 5.2 Hz, 1H), 4.18 (q, *J* = 7.1 Hz, 2H), 2.98 (s, 2H), 1.74 – 1.65 (comp, 4H), 1.62 – 1.30 (comp, 6H), 1.04 (t, *J* = 7.1 Hz, 3H); ^{13}C NMR (75 MHz, $CDCl_3$) (δ, ppm) 171.7, 157.9, 148.3, 135.2, 134.8, 129.6, 128.3, 128.1, 125.1, 124.6, 123.9, 123.2, 123.0, 119.9, 105.0, 61.5, 36.4, 36.2, 35.3, 26.3, 22.6, 13.7.

HRMS (TOF MS ESI$^+$) calculated for $C_{24}H_{25}O_3S^+$ [M+H]$^+$: 393.1519, found 393.1519.

Ethyl 6-hydroxy-2-methoxy-12-methyl-12-phenyl-11,12-dihydrochrysene-5-carb oxylate (39).

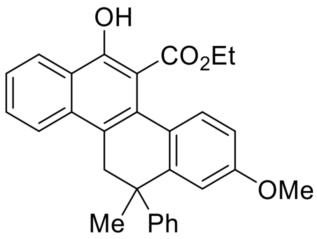

Yellow solid; m.p. 152.0 – 154.0 ˚C. 66.7 mg, 76% yield. ^{1}H NMR (400 MHz, $CDCl_3$) (δ, ppm) 10.99 (s, 1H), 8.37 (d, J = 8.3 Hz, 1H), 8.06 (d, J = 8.3 Hz, 1H), 7.67 – 7.58 (m, 1H), 7.45 (t, J = 7.6 Hz, 1H), 7.23 – 7.17 (comp, 2H), 7.16 – 7.12 (m, 1H), 7.11 – 7.00 (comp, 4H), 6.89 – 6.83 (m, 1H), 4.27 – 4.10 (m, 2H), 3.92 – 3.82 (comp, 4H), 2.94 (d, J = 16.1 Hz, 1H), 1.87 (s, 3H), 1.14 – 1.01 (m, 3H). ^{13}C NMR (100 MHz, $CDCl_3$) (δ, ppm) 172.0, 158.9, 158.2, 146.9, 144.6, 134.2, 131.5, 131.1, 129.6, 129.1, 127.7, 126.8, 125.9, 125.2, 124.5, 124.4, 123.9, 122.8, 111.4, 110.6, 104.7, 61.1, 55.5, 42.4, 38.7, 28.7, 13.8. HRMS (TOF MS ESI$^+$) calculated for $C_{29}H_{26}NaO_4^+$ [M+Na]$^+$: 461.1723, found: 461.1714.

General Procedure for the Optimization of Asymmetric [4+2] Cycloaddition.

Supplementary Table 1. Asymmetric catalysis studies.[a]

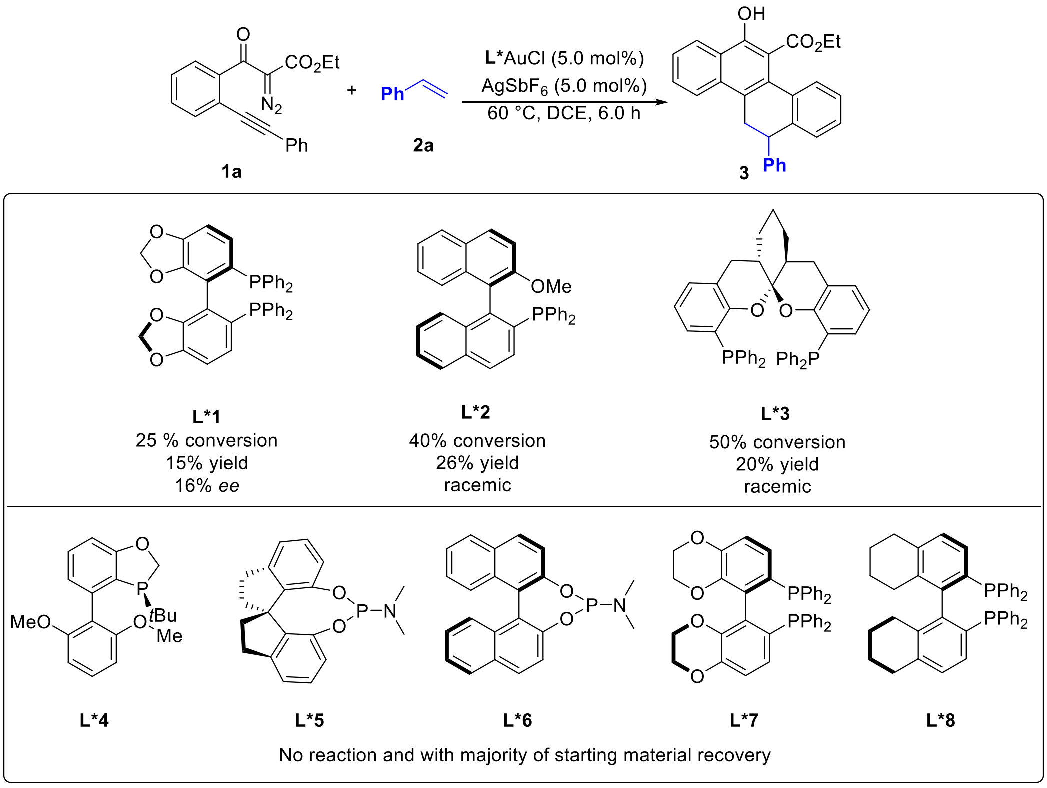

[a]To a 10-mL oven-dried vial containing a magnetic stirring bar, the L*AuCl complex (0.01 mmol for **L*2**, and **L*4-L*6**; 0.005 for the others), $AgSbF_6$ (3.43 mg, 0.01 mmol), and DCE (0.5 mL) were added in sequence in a nitrogen-filled glove-box. The reaction was stirred at 25 ˚C for 2.0 hours. The solvent was removed and the residue was dissolved in DCE (0.5 mL). The mixture was filtered through a pad of Celite. The filtrate was added into a solution of **1a** (31.8 mg, 0.1 mmol) and styrene (15.6 mg, 0.15 mmol) in DCE (0.5 mL) at 60 °C, and the resulting reaction mixture was stirred under these conditions for 6.0 hours. Then, the crude the reaction mixture was subjected to ^{1}H NMR analysis with mesitylene as internal standard for the determination of the yields and conversion of **1a**. The yields and conversions were determined by proton NMR with mesitylene as internal standard based on limited reagent **1a**. The *ee* values were Determined by chiral HPLC analysis.

General Procedure for the Synthesis of Polycyclic Aromatic Hydrocarbons

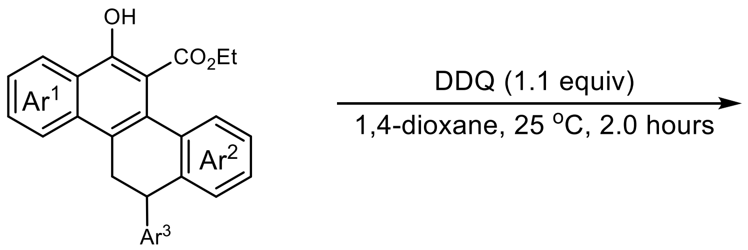

To a 10-mL oven-dried flask equipped with a magnetic stirring bar, the above prepared polycyclic products (0.10 mmol), 2,3-Dicyano-5,6-dichlorobenzoquinone (DDQ, 25.0 mg, 0.11 mmol), and 1,4-dioxane (6.0 mL) were added in sequence. The reaction mixture was stirred at 25 ˚C for 12.0 hours. Then, the solvent was removed under reduced pressure and the crude product was purified by column chromatography on silica gel (eluent: petroleum ether/ethyl acetate = 20:1) to give the polycyclic aromatic hydrocarbons **40-55** in high to excellent yields.

Characterization of the polycyclic aromatic hydrocarbons 40-55.

Ethyl 7-fluoro-6-hydroxy-12-phenylchrysene-5-carboxylate (40).

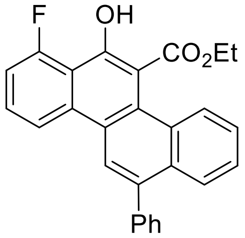

Yellow solid, m.p. 157 – 158 ˚C. 39.4 mg, 96% yield. ^{1}H NMR (300 MHz, $CDCl_3$) (δ, ppm) 11.20 (s, 1H), 8.46 – 8.26 (comp, 2H), 8.17 – 8.03 (m, 1H), 8.01 – 7.87 (m, 1H), 7.81 – 7.39 (comp, 8H), 7.32 – 7.24 (m, 1H), 4.41 (q, *J* = 7.1 Hz, 2H), 1.20 (t, *J* = 7.1 Hz, 3H); ^{13}C NMR (75 MHz, $CDCl_3$) (δ, ppm) 172.2, 161.1 (d, *J* = 260.0 Hz), 157.7 (d, *J* = 3.8 Hz), 140.8, 138.3, 135.7 (d, *J* = 2.1 Hz), 132.0, 130.28 (d, *J* = 9.7 Hz), 130.26, 129.7, 129.2, 128.5, 127.6, 127.0 (d, *J* = 1.0 Hz), 126.6, 126.2, 124.6, 122.9 (d, *J* = 2.5 Hz), 121.3, 119.2 (d, *J* = 4.2 Hz), 114.4 (d, *J* = 8.3 Hz), 113.5 (d, *J* = 22.6 Hz), 105.2 (d, *J* = 1.9 Hz), 62.0, 13.9; ^{19}F NMR (283 MHz, $CDCl_3$) (δ, ppm) -109.46. HRMS (EI) calculated for $C_{27}H_{19}FO_3$ (*m/z*): 410.1318, found 410.1324.

Ethyl 8-fluoro-6-hydroxy-12-phenylchrysene-5-carboxylate (**41**).

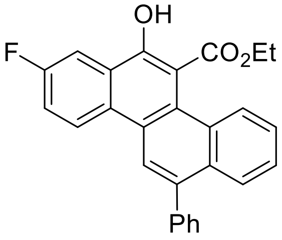

Yellow solid, m.p. 178 – 179 ˚C. 37.8 mg, 92% yield. ^{1}H NMR (400 MHz, $CDCl_3$) (δ, ppm) 11.06 (s, 1H), 8.61 (dd, *J* = 9.2, 5.1 Hz, 1H), 8.38 (s, 1H), 8.17 (dd, *J* = 9.7, 2.8 Hz, 1H), 8.08 – 8.00 (m, 1H), 7.98 – 7.92 (m, 1H), 7.68 – 7.61 (m, 2H), 7.59 – 7.54 (m, 2H), 7.53 – 7.45 (comp, 4H), 4.40 (q, *J* = 7.1 Hz, 2H), 1.19 (t, *J* = 7.1 Hz, 3H); ^{13}C NMR (100 MHz, $CDCl_3$) (δ, ppm) 172.5, 161.6 (d, *J* = 247.7 Hz), 157.7 (d, *J* = 4.0 Hz), 140.9, 138.4, 131.7, 130.3, 130.0, 129.9 (d, *J* = 1.9 Hz), 129.3, 128.6, 127.6, 126.5 (d, *J* = 8.7 Hz), 126.3 (d, *J* = 1.5 Hz), 125.9 (d, *J* = 1.6 Hz), 125.7 (d, *J* = 8.5 Hz), 124.5, 123.4 (d, *J* = 0.5 Hz), 121.0, 119.2 (d, *J* = 23.9 Hz), 109.6 (d, *J* = 22.6 Hz), 104.9, 62.1, 13.9; ^{19}F NMR (283 MHz, $CDCl_3$) (δ, ppm) -113.28. HRMS (EI) calculated for $C_{27}H_{19}FO_3$ (*m/z*): 410.1318, found 410.1314.

Ethyl 9-fluoro-6-hydroxy-12-phenylchrysene-5-carboxylate (**42**).

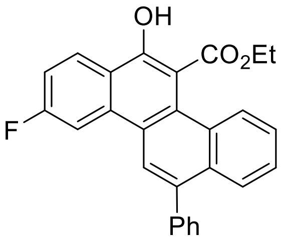

Yellow solid, m.p. 198 – 199 ˚C. 36.9 mg, 90% yield. ^{1}H NMR (300 MHz, $CDCl_3$) (δ, ppm) 11.26 (s, 1H), 8.55 (dd, *J* = 9.0, 6.0 Hz, 1H), 8.26 (s, 1H), 8.19 (dd, *J* = 11.1, 2.4 Hz, 1H), 8.10 – 8.00 (m, 1H), 7.99 – 7.89 (m, 1H), 7.67 – 7.45 (comp, 7H), 7.42 – 7.32 (m, 1H), 4.39 (q, *J* = 7.1 Hz, 2H), 1.19 (t, *J* = 7.1 Hz, 3H); ^{13}C NMR (100 MHz, $CDCl_3$) (δ, ppm) 172.5, 164.2 (d, *J* = 250.0 Hz), 158.7, 140.8, 138.1, 135.4 (d, *J* = 9.2 Hz), 132.1, 130.3, 130.0, 129.6, 128.6, 127.7 (d, *J* = 9.6 Hz), 127.6, 127.4, 126.6, 126.3, 124.4, 122.9 (d, *J* = 4.1 Hz), 121.6 (d, *J* = 1.3 Hz), 121.1, 116.0 (d, *J* = 23.8 Hz), 108.4 (d, *J* = 22.9 Hz), 103.3

(d, *J* = 1.7 Hz), 61.9, 14.0; ^{19}F NMR (283 MHz, $CDCl_3$) (δ, ppm) -108.13. HRMS (EI) calculated for $C_{27}H_{19}FO_3$ (*m/z*): 410.1318, found 410.1328.

Ethyl 10-fluoro-6-hydroxy-12-phenylchrysene-5-carboxylate (43).

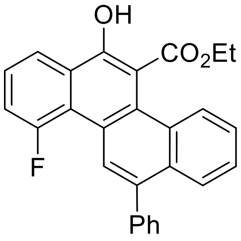

Yellow solid, m.p. 182 – 183 ˚C. 38.2 mg, 93% yield. ^{1}H NMR (400 MHz, $CDCl_3$) (δ, ppm) 11.22 (s, 1H), 8.97 (d, *J* = 3.7 Hz, 1H), 8.48 – 8.37 (m, 1H), 8.07 – 8.01 (m, 1H), 8.01 – 7.95 (m, 1H), 7.69 – 7.64 (comp, 2H), 7.62 – 7.54 (comp, 3H), 7.52 – 7.45 (comp, 4H), 4.38 (q, *J* = 7.1 Hz, 2H), 1.17 (t, *J* = 7.1 Hz, 3H); ^{13}C NMR (100 MHz, $CDCl_3$) (δ, ppm) 172.5, 160.9 (d, *J* = 253.4 Hz), 158.0 (d, *J* = 4.2 Hz), 141.1, 137.9 (d, *J* = 2.3 Hz), 131.5 (d, *J* = 1.9 Hz), 130.4, 129.8, 129.6, 128.5, 127.430 (d, *J* = 4.8 Hz), 127.429, 127.1 (d, *J* = 9.9 Hz), 127.0 (d, *J* = 0.6 Hz), 126.5, 126.0, 125.1 (d, *J* = 26.0 Hz), 124.2, 122.5 (d, *J* = 9.1 Hz), 122.1 (d, *J* = 5.9 Hz), 121.0 (d, *J* = 3.7 Hz), 117.2 (d, *J* = 25.3 Hz), 104.6 (d, *J* = 0.6 Hz), 62.0, 13.9; ^{19}F NMR (283 MHz, $CDCl_3$) (δ, ppm) -108.61. HRMS (EI) calculated for $C_{27}H_{19}FO_3$ (*m/z*): 410.1318, found 410.1304.

Ethyl 6-hydroxy-4-methyl-12-phenylchrysene-5-carboxylate (**44**).

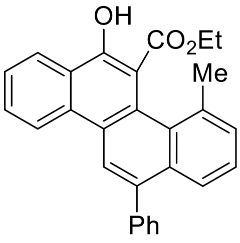

Yellow solid, m.p. 151 – 153 ˚C. 32.9 mg, 81% yield. ^{1}H NMR (300 MHz, $CDCl_3$) (δ, ppm) 12.31 (s, 1H), 8.71 – 8.57 (comp, 2H), 8.38 (s, 1H), 7.83 – 7.75 (comp, 2H), 7.72 – 7.63 (comp, 3H), 7.61 – 7.50 (comp, 3H), 7.42 (t, *J* = 7.6 Hz, 1H), 7.32 (d, *J* = 6.9 Hz, 1H), 4.33 – 4.15 (m, 2H), 2.70 (s, 3H), 1.04 (t, *J* = 7.2 Hz, 3H); ^{13}C NMR (75 MHz, $CDCl_3$) (δ, ppm) 172.3, 158.6, 141.3, 138.0, 136.8, 133.4, 132.3, 131.8, 130.4, 130.3, 128.4, 127.6, 127.4, 126.8, 125.9, 125.1, 124.94, 124.86, 124.3, 123.6, 123.2, 120.6, 106.0, 61.7, 22.8, 13.6. HRMS (EI) calculated for $C_{28}H_{22}O_3$ (*m/z*): 406.1569, found 406.1564.

Ethyl 6-hydroxy-2-methyl-12-phenylchrysene-5-carboxylate (**45**).

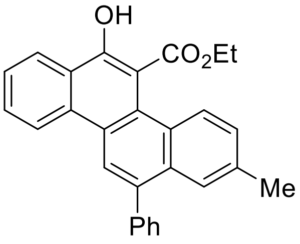

Yellow solid, m.p. 154.0 – 155.0 ˚C. 34.6 mg, 85% yield. ^{1}H NMR (400 MHz, $CDCl_3$) (δ, ppm) 11.16 (s, 1H), 8.62 (d, *J* = 8.4 Hz, 1H), 8.56 – 8.54 (m, 1H), 8.43 (s, 1H), 7.95 (d, *J* = 8.6 Hz, 1H), 7.79 – 7.75 (m, 1H), 7.71 (d, *J* = 1.6 Hz, 1H), 7.68 – 7.62 (comp, 3H), 7.59 – 7.54 (m, 2H), 7.52 – 7.48 (m, 1H), 7.32 (dd, *J* = 8.6, 1.8 Hz, 1H), 4.39 (q, *J* = 7.1 Hz, 2H), 2.47 (s, 3H), 1.21 (t, *J* = 7.1 Hz, 3H); ^{13}C NMR (100 MHz, $CDCl_3$) (δ, ppm) 172.7, 158.8, 141.2, 137.6, 136.1, 133.4, 132.0, 130.4, 130.2, 129.4, 128.5, 128.1, 127.4, 126.8, 126.5, 126.3, 125.4, 124.8, 124.8, 123.1, 123.0, 121.3, 103.8, 61.9, 21.9, 14.1. HRMS (TOF MS CI^+) calculated for $C_{28}H_{23}O_3^+$ $[M+H]^+$: 407.1642, found 407.1635.

Ethyl 6-hydroxy-3-methyl- 12-phenylchrysene-5-carboxylate (**46a**) &
Ethyl 6-hydroxy-1-methyl-12-phenylchrysene-5-carboxylate (**46b**).

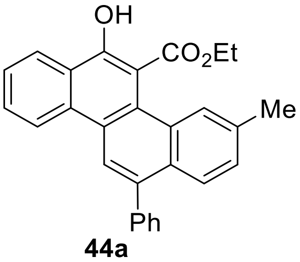

44a

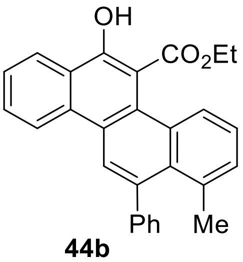

44b

Yellow solid. Combined 32.1 mg in total with 79% yield, **42a**:**42b** = 4:1. *Combined two isomers*: ^{1}H NMR (300 MHz, $CDCl_3$) (δ, ppm) 11.27 – 11.01 (comp, 1H), 8.54 – 8.39 (comp, 2H), 8.31 – 8.17 (comp, 1H), 7.82 – 7.16 (comp, 10H), 4.37 – 4.17 (comp, 2H), 2.46 – 1.98 (comp, 3H), 1.14 – 1.03 (comp, 3H); ^{13}C NMR (75 MHz, $CDCl_3$) (δ, ppm) 172.8, 172.7, 159.0, 158.7, 141.2, 137.8, 137.7, 133.7, 133.4, 130.3, 130.2, 130.2, 130.1, 130.0, 129.7, 129.0, 128.5, 128.1, 127.9, 127.5, 127.0, 126.9, 126.1, 126.0, 124.9, 124.8, 124.8, 123.94, 123.86, 123.8, 123.1, 120.4, 104.0, 1038, 61.8, 25.6, 22.0, 14.0, 13.9. HRMS (EI) calculated for $C_{28}H_{22}O_3$ (*m/z*): 406.1569, found 406.1577.

Ethyl 6-hydroxy-2-methoxy-12-phenylchrysene-5-carboxylate (**47**).

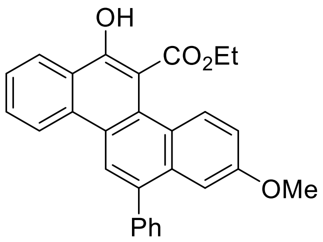

Yellow solid, m.p. 173.0 – 174.0 ˚C. 38.6 mg, 92% yield. ^{1}H NMR (400 MHz, $CDCl_3$) (δ, ppm) 11.24 (s, 1H), 8.63 – 8.50 (m, 2H), 8.44 (s, 1H), 7.97 (d, *J* = 9.2 Hz, 1H), 7.77 – 7.73 (m, 1H), 7.68 – 7.61 (comp, 3H), 7.59 – 7.53 (m, 2H), 7.53 – 7.46 (m, 1H), 7.32 (d, *J* = 2.7 Hz, 1H), 7.15 (dd, *J* = 9.2, 2.7 Hz, 1H), 4.40 (q, *J* = 7.1 Hz, 2H), 3.81 (s, 3H), 1.22 (t, *J* = 7.1 Hz, 3H); ^{13}C NMR (100 MHz, $CDCl_3$) (δ, ppm) 172.7, 159.0, 157.9, 141.2, 137.2, 133.5, 133.3, 131.0, 130.2, 130.2, 128.6, 127.5, 126.6, 126.5, 124.9, 124.8, 124.5, 122.8, 122.3, 121.9, 115.5, 105.6, 103.5, 61.9, 55.4, 14.1. HRMS (TOF MS CI$^+$) calculated for $C_{28}H_{23}O_4^+$ [M+H]$^+$: 423.1591, found 423.1588.

Ethyl 5-hydroxy-13-phenylpicene-6-carboxylate (**48**).

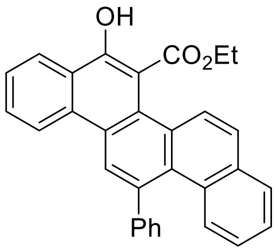

Yellow solid, m.p. 191.0 – 193.0 ˚C. 38.0 mg, 86% yield. ^{1}H NMR (400 MHz, $CDCl_3$) (δ, ppm) 11.49 (s, 1H), 8.64 (d, *J* = 8.3 Hz, 1H), 8.58 – 8.56 (m, 1H), 8.47 (s, 1H), 7.95 (d, *J* = 9.0 Hz, 1H), 7.88 (dd, *J* = 8.0, 1.4 Hz, 1H), 7.82 – 7.65 (comp, 4H), 7.56 – 7.41 (comp, 6H), 7.16 – 7.12 (m, 1H), 4.34 (q, *J* = 7.1 Hz, 2H), 1.09 (t, *J* = 7.1 Hz, 3H); ^{13}C NMR (100 MHz, $CDCl_3$) (δ, ppm) 172.7, 159.7, 145.6, 137.7, 133.2, 133.0, 130.5, 130.2, 129.9, 129.6, 129.09, 129.06, 128.9, 127.9, 127.8, 127.3, 127.2, 126.7, 126.2, 125.1, 125.0, 124.8, 124.6, 124.1, 123.2, 103.5, 61.9, 14.0. HRMS (TOF MS CI$^+$) calculated for $C_{31}H_{23}O_3^+$ [M+H]$^+$: 443.1642, found 443.1650.

Ethyl 8-hydroxy-14-phenylbenzo[*c*]chrysene-7-carboxylate (**49**).

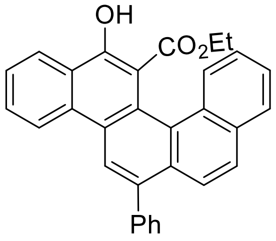

Yellow solid, m.p. 186 – 187 ˚C. 43.4 mg, 98% yield. ^{1}H NMR (300 MHz, $CDCl_3$) (δ, ppm) 12.02 (s, 1H), 8.88 – 8.70 (m, 1H), 8.72 – 8.60 (comp, 2H), 8.58 (s, 1H), 7.97 – 7.87 (comp, 2H), 7.83 – 7.51 (comp, 10H), 3.78 – 3.67 (m, 1H), 3.36 – 3.24 (m, 1H), 0.42 (t, *J* = 7.2 Hz, 3H); ^{13}C NMR (75 MHz, $CDCl_3$) (δ, ppm) 172.0, 158.9, 141.1, 137.6, 133.3, 133.1, 131.4, 130.6, 130.3, 129.7, 128.5, 128.0, 127.5, 127.12, 127.08, 126.3, 126.1, 125.8, 125.2, 125.1, 125.0, 124.6, 124.2, 123.2, 122.0, 106.0, 61.4, 12.9. HRMS (EI) calculated for $C_{31}H_{22}O_3$ (*m/z*): 442.1569, found 442.1562.

Ethyl 5-hydroxy-11-phenylphenanthro[2,1-*b*]thiophene-4-carboxylate (**50**).

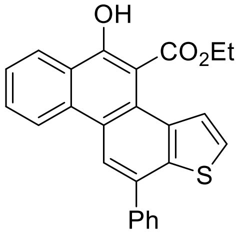

Yellow solid, m.p. 155 – 156 ˚C. 33.8 mg, 84% yield. ^{1}H NMR (500 MHz, $CDCl_3$) (δ, ppm) 11.17 (s, 1H), 8.67 (d, *J* = 8.4 Hz, 1H), 8.53 (d, *J* = 8.1 Hz, 1H), 8.50 (s, 1H), 7.86 (d, *J* = 7.6 Hz, 2H), 7.78 (t, *J* = 7.6 Hz, 1H), 7.66 (t, *J* = 7.5 Hz, 1H), 7.63 – 7.54 (comp, 3H), 7.54 – 7.44 (comp, 2H), 4.50 (q, *J* = 7.1 Hz, 2H), 1.33 (t, *J* = 7.1 Hz, 3H); ^{13}C NMR (125 MHz, $CDCl_3$) (δ, ppm) 171.9, 158.9, 140.8, 139.9, 135.8, 134.2, 133.9, 130.4, 129.0, 128.7, 128.3, 126.8, 126.7, 124.9, 124.52, 124.47, 124.3, 124.2, 123.1, 119.0, 103.6, 62.0, 14.2. HRMS (EI) calculated for $C_{25}H_{18}O_3S$ (*m/z*): 398.0977, found 398.0989.

Ethyl 12-([1,1'-biphenyl]-2-yl)-6-hydroxy-2-methoxychrysene-5-carboxylate (**51**).

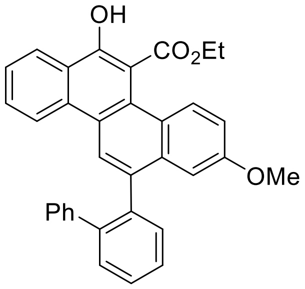

Yellow solid, m.p. 138 – 139 ˚C. 44.4 mg, 89% yield. ^{1}H NMR (400 MHz, $CDCl_3$) (δ, ppm) 11.15 (s, 1H), 8.51 (dd, *J* = 8.2, 1.0 Hz, 1H), 8.39 (d, *J* = 8.2 Hz, 1H), 8.33 (s, 1H), 7.83 (d, *J* = 9.1 Hz, 1H), 7.74 – 7.49 (comp, 6H), 7.19 – 7.12 (m, 2H), 7.07 – 6.90 (comp, 5H), 4.60 – 4.14 (m, 2H), 3.74 (s, 3H), 1.11 (t, *J* = 7.1 Hz, 3H); ^{13}C NMR (100 MHz, $CDCl_3$) (δ, ppm) 172.6, 158.8, 157.6, 142.1, 141.5, 139.4, 136.4, 133.5, 133.4, 131.9,

130.6, 130.4, 130.1, 129.1, 128.2, 127.8, 127.6, 126.6, 126.4, 124.78, 124.77, 124.4, 123.2, 122.8, 122.1, 115.4, 105.5, 103.6, 61.6, 55.3, 13.9. HRMS (EI) calculated for $C_{34}H_{26}O_4$ (*m/z*): 498.1831, found 498.1834.

Ethyl 12-(2-chlorophenyl)-6-hydroxy-2-methoxychrysene-5-carboxylate (**52**).

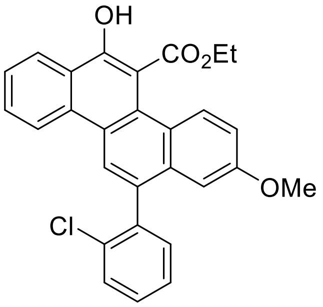

Yellow solid, m.p. 207 – 209 ˚C. 41.8 mg, 92% yield. ^{1}H NMR (400 MHz, $CDCl_3$) (δ, ppm) 11.24 (s, 1H), 8.61 – 8.49 (m, 2H), 8.42 (s, 1H), 7.98 (d, *J* = 9.2 Hz, 1H), 7.77 – 7.73 (m, 1H), 7.66 – 7.60 (m, 2H), 7.55 – 7.50 (m, 1H), 7.48 – 7.41 (m, 2H), 7.15 (dd, *J* = 9.2, 2.7 Hz, 1H), 6.89 (d, *J* = 2.6 Hz, 1H), 4.39 (t, *J* = 7.2 Hz, 2H), 3.79 (s, 3H), 1.22 (t, *J* = 7.2 Hz, 3H); ^{13}C NMR (100 MHz, $CDCl_3$) (δ, ppm) 172.7, 159.2, 157.9, 139.7, 134.5, 134.4, 133.5, 133.2, 132.5, 131.0, 130.3, 129.9, 129.3, 127.1, 127.0, 126.6, 124.8, 124.6, 124.5, 122.8, 122.4, 122.1, 115.5, 105.5, 103.6, 61.8, 55.3, 14.1. HRMS (TOF MS CI$^+$) calculated for $C_{28}H_{22}ClO_4^+$ $[M+H]^+$: 457.1201, found 457.1208.

Ethyl 12-(3-chlorophenyl)-6-hydroxy-2-methoxychrysene-5-carboxylate (**53**).

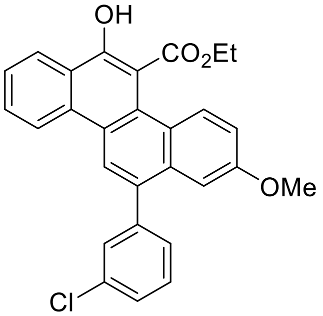

Yellow solid, m.p. 214 – 216 ˚C. 43.0 mg, 94% yield. ^{1}H NMR (400 MHz, $CDCl_3$) (δ, ppm) 11.25 (s, 1H), 8.63 – 8.49 (m, 2H), 8.40 (s, 1H), 7.95 (d, *J* = 9.2 Hz, 1H), 7.79 – 7.74 (m, 1H), 7.70 – 7.59 (m, 2H), 7.57 – 7.45 (comp, 3H), 7.24 (d, *J* = 2.7 Hz, 1H), 7.15 (dd, *J* = 9.2, 2.7 Hz, 1H), 4.39 (q, *J* = 7.1 Hz, 2H), 3.82 (s, 3H), 1.21 (t, *J* = 7.1 Hz, 3H); ^{13}C NMR (100 MHz, $CDCl_3$) (δ, ppm) 172.6, 159.3, 158.0, 143.0, 135.6, 134.6, 133.4, 133.0, 131.2, 130.4, 130.2, 129.8, 128.4, 127.7, 127.0, 126.7, 124.94, 124.89, 124.5, 122.8, 122.1, 122.0, 115.6, 105.3, 103.4, 61.9, 55.4, 14.1. HRMS (TOF MS CI$^+$) calculated for $C_{28}H_{22}ClO_4^+$ $[M+H]^+$: 457.1201, found 457.1212.

Ethyl 12-(4-chlorophenyl)-6-hydroxy-2-methoxychrysene-5-carboxylate (**54**).

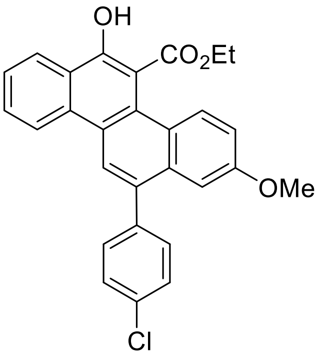

Yellow solid, m.p. 107 – 109 ˚C. 40.7 mg, 89% yield. ^{1}H NMR (300 MHz, $CDCl_3$) (δ, ppm) 11.25 (s, 1H), 8.62 – 8.47 (comp, 2H), 8.37 (s, 1H), 7.95 (d, *J* = 9.2 Hz, 1H), 7.78 – 7.70 (m, 1H), 7.65 – 7.50 (comp, 5H), 7.23 (d, *J* = 2.6 Hz, 1H), 7.15 (dd, *J* = 9.2, 2.6 Hz, 1H), 4.39 (q, *J* = 7.1 Hz, 2H), 3.82 (s, 3H), 1.22 (t, *J* = 7.1 Hz, 3H); ^{13}C NMR (75 MHz, $CDCl_3$) (δ, ppm) 172.6, 159.2, 158.0, 139.7, 135.8, 133.54, 133.35, 133.0, 131.5, 131.1, 130.3, 128.8, 126.8, 126.6, 124.93, 124.86 124.5, 122.7, 122.1, 121.9, 115.6, 105.3, 103.4, 61.9, 55.4, 14.1. HRMS (EI) calculated for $C_{28}H_{21}ClO_4$ (*m/z*): 456.1128, found 456.1622.

Ethyl 12-(4-(*tert*-butyl)phenyl)-6-hydroxy-2-methoxychrysene-5-carboxylate (**55**).

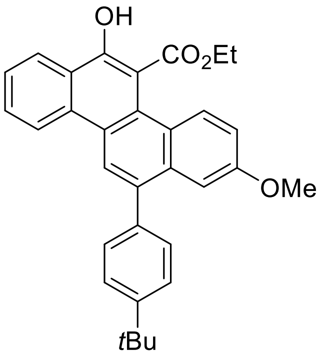

Yellow solid, m.p. 193 – 194 ˚C. 45.2 mg, 95% yield. ^{1}H NMR (400 MHz, $CDCl_3$) (δ, ppm) 11.23 (s, 1H), 8.65 – 8.51 (m, 2H), 8.44 (s, 1H), 7.97 (d, *J* = 9.2 Hz, 1H), 7.77 – 7.72 (m, 1H), 7.68 – 7.54 (comp, 5H), 7.40 (d, *J* = 2.7 Hz, 1H), 7.16 (dd, *J* = 9.2, 2.7 Hz, 1H), 4.41 (q, *J* = 7.1 Hz, 2H), 3.84 (s, 3H), 1.47 (s, 9H), 1.23 (t, *J* = 7.1 Hz, 3H); ^{13}C NMR (100 MHz, $CDCl_3$) (δ, ppm) 172.7, 158.9, 157.8, 150.4, 138.2, 137.1, 133.5, 133.4, 131.0, 130.2, 129.8, 126.5, 126.4, 125.5, 125.0, 124.8, 124.5, 122.8, 122.3, 121.9, 115.2, 106.0, 103.5, 61.8, 55.4, 34.8, 31.6, 14.1. HRMS (TOF MS CI$^+$) calculated for $C_{32}H_{31}O_4^+$ $[M+H]^+$: 479.2217, found 479.2224.

General Procedure of Mechanistic Experiments and Synthesis of 56-58

Synthesis of 56

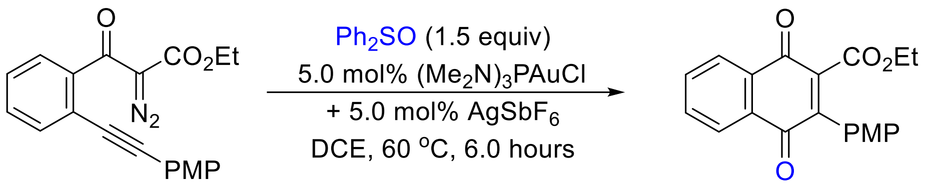

1l **56**, 84 % yield

To a 10-mL oven-dried vial containing a magnetic stirring bar, $(Me_2N)_3PAuCl$ (3.95 mg, 0.01 mmol), $AgSbF_6$ (3.43 mg, 0.01 mmol), and DCE (0.5 mL) were added in sequence in a nitrogen-filled glove-box. The reaction mixture was stirred at 25 ˚C for 2.0 hours. The solvent was removed and the residue was dissolved in DCE (0.5 mL). Then the mixture was filtered through a pad of Celite. The filtrate was added into a solution of **1l** (69.9 mg, 0.2 mmol) and phenyl sulfoxide (60.7 mg, 0.3 mmol) in DCE (0.5 mL) at 60 ˚C, and the resulting reaction mixture was stirred under these conditions for 6.0 hours. Then, the solvent was removed under reduced pressure and the crude product was purified by column chromatography on silica gel (solvents: petroleum ether/ethyl acetate = 10: 1) to give 56.5 mg **56** in 84% yield. White solid; m. p. 55.0 – 57.0 ˚C. ^1H NMR (400 MHz, $CDCl_3$) (δ, ppm) 8.16 – 8.10 (comp, 2H), 7.80 – 7.75 (comp, 2H), 7.39 – 7.34 (comp, 2H), 6.97 – 6.93 (m, 2H), 4.22 (q, J = 7.1 Hz, 2H), 3.84 (s, 3H), 1.13 (t, J = 7.1 Hz, 3H); ^{13}C NMR (100 MHz, $CDCl_3$) (δ, ppm) 184.5, 182.2, 164.6, 161.1, 144.1, 138.8, 134.3, 134.3, 131.8, 131.6, 131.3, 127.1, 126.4, 123.7, 113.8, 62.0, 55.5, 14.0. HRMS (TOF MS ESI$^+$) calculated for $C_{20}H_{16}NaO_5^+$ $[M+Na]^+$: 359.0890, found: 359.0894.

Synthesis of 57

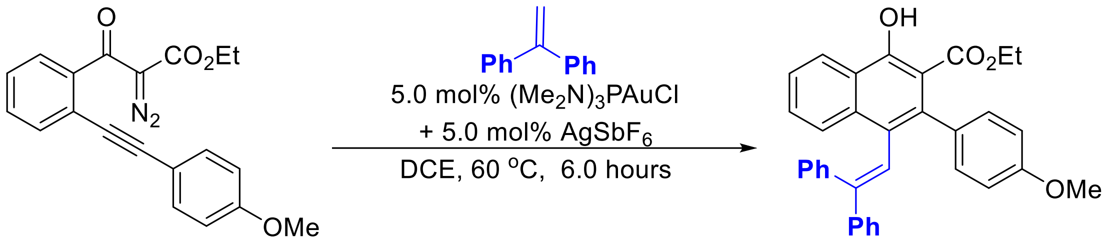

1l **57**, 71%

To a 10-mL oven-dried vial containing a magnetic stirring bar, $(Me_2N)_3PAuCl$ (3.95 mg, 0.01 mmol), $AgSbF_6$ (3.43 mg, 0.01 mmol), and DCE (0.5 mL) were added in sequence in a nitrogen-filled glove-box. The reaction mixture was stirred at 25 ˚C for 2.0 hours. The solvent was removed and the residue was dissolved in DCE (0.5 mL). Then the mixture was filtered through a pad of Celite. The filtrate was added into a solution of **1l** (69.9 mg, 0.2 mmol) and 1,1-diphenylethylene (54.0 mg, 0.3 mmol) in DCE (0.5 mL) at 60 ˚C, and the resulting reaction mixture was stirred under these conditions for 6.0 hours. Then, the solvent was removed under reduced pressure and the crude product was purified by column chromatography on silica gel (solvents: petroleum ether/ethyl acetate = 10: 1) to give 71.1 mg **57** in 71% yield. White solid; m. p. 155.0 – 157.0 ˚C. ^1H NMR (400 MHz, $CDCl_3$) (δ, ppm) 12.11 (s, 1H), 8.47 (d, J = 8.0 Hz, 1H), 8.04 (d, J = 8.0 Hz, 1H), 7.64 – 7.47 (comp, 2H), 7.34 – 7.26 (comp, 3H), 7.22 – 7.08 (comp, 3H), 7.05 – 6.99 (m, 1H), 6.99 – 6.82 (comp, 3H), 6.81 (s, 1H), 6.70 – 6.44 (comp, 3H), 6.39 – 6.27 (m, 1H), 3.91 (q, J = 7.1 Hz, 2H), 3.79 (s, 3H), 0.72 (t, J = 7.1 Hz, 3H) ^{13}C NMR (100 MHz, $CDCl_3$) (δ, ppm) 172.2, 160.3, 158.1, 145.1, 143.8, 139.8, 136.7, 135.4, 134.7, 130.1, 129.8, 128.5, 128.1, 127.4, 127.0, 126.9, 126.3, 126.1, 125.7, 124.2, 124.1, 112.7, 107.3, 61.1, 55.5, 13.2. HRMS (TOF MS ESI$^+$) calculated for $C_{34}H_{28}NaO_4^+$ $[M+Na]^+$: 523.1880, found: 523.1877.

Synthesis of 58

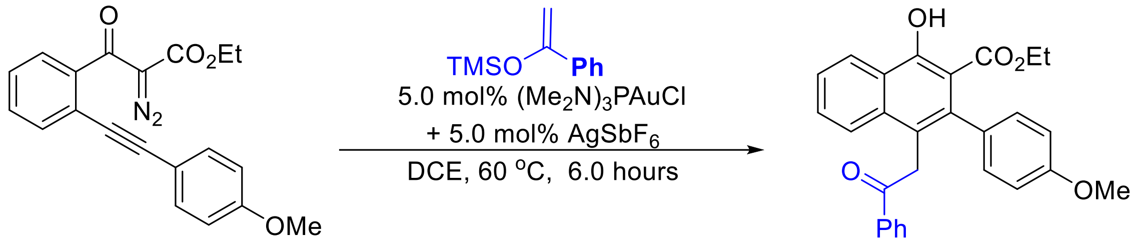

1l **58**, 80 % yield

To a 10-mL oven-dried vial containing a magnetic stirring bar, $(Me_2N)_3PAuCl$ (3.95 mg, 0.01 mmol), $AgSbF_6$ (3.43 mg, 0.01 mmol), and DCE (0.5 mL) were added in sequence in a nitrogen-filled glove-box. The reaction mixture was stirred at 25 ˚C for 2.0 hours. The solvent was removed and the residue was dissolved in DCE (0.5 mL). Then the mixture was filtered through a pad of Celite. The filtrate was added into a solution of **1l** (69.9 mg, 0.2 mmol) and 1-phenyl-1-trimethylsiloxyethene (57.7 mg, 0.3 mmol) in DCE (0.5 mL) at 60 ˚C, and the resulting reaction mixture was stirred under these conditions for 6.0 hours. Then, the solvent was removed under reduced pressure and the crude product was purified by column chromatography on silica gel (solvents: petroleum ether/ethyl acetate = 10: 1) to give 70.5 mg **58** in 80% yield. Yellow oil. ^{1}H NMR (400 MHz, $CDCl_3$) (δ, ppm) 12.49 (s, 1H), 8.53 (d, *J* = 8.1 Hz, 1H), 7.94 – 7.87 (comp, 2H), 7.60 – 7.50 (comp, 4H), 7.48 – 7.42 (comp, 2H), 7.13 – 7.02 (comp, 2H), 6.86 – 6.77 (comp, 2H), 4.42 (s, 2H), 3.96 (q, *J* = 7.2 Hz, 2H), 3.78 (d, *J* = 2.6 Hz, 3H), 0.76 (t, *J* = 7.1 Hz, 3H); ^{13}C NMR (100 MHz, $CDCl_3$) (δ, ppm) 198.2, 172.2, 160.9, 158.6, 138.8, 136.9, 135.4, 135.0, 133.3, 130.3, 129.9, 128.7, 128.2, 125.6, 124.84, 124.75, 124.3, 121.9, 113.3, 107.0, 61.1, 55.5, 40.1, 13.3. HRMS (TOF MS ESI$^+$) calculated for $C_{28}H_{24}NaO_5^+$ $[M+Na]^+$: 463.1516, found: 463.1529.

General Procedure for the Synthesis of 59-62

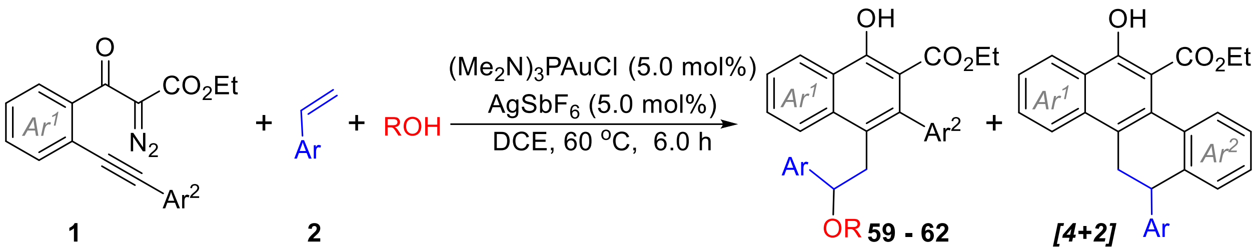

1 **2** **59 - 62** ***[4+2]***

Synthesis of **59**: To a 10-mL oven-dried vial containing a magnetic stirring bar, $(Me_2N)_3PAuCl$ (3.95 mg, 0.01 mmol), $AgSbF_6$ (3.43 mg, 0.01 mmol), and DCE (0.5 mL) were added in sequence in a nitrogen-filled glove-box. The reaction mixture was stirred at 25 ˚C for 2.0 hours. The solvent was removed and the residue was dissolved in DCE (0.5 mL). Then the mixture was filtered through a pad of Celite. The filtrate was added into a solution of **1l** (69.9 mg, 0.2 mmol), 2-bromobenzyl alcohol (56.1 mg, 0.3 mmol), and 4-methoxystyrene (40.2 mg, 0.3 mmol) in DCE (0.5 mL) at 60 ˚C, and the resulting reaction mixture was stirred under these conditions for 6.0 hours. Then, the solvent was removed under reduced pressure and the crude product was purified by column chromatography on silica gel to give **59**.

Synthesis of **60-62**: To a 10-mL oven-dried vial containing a magnetic stirring bar, $(Me_2N)_3PAuCl$ (3.95 mg, 0.01 mmol), $AgSbF_6$ (3.43 mg, 0.01 mmol), and DCE (0.5 mL) were added in sequence in a nitrogen-filled glove-box. The reaction mixture was stirred at 25 ˚C for 2.0 hours. The solvent was removed and the residue was dissolved in DCE

(0.5 mL). Then the mixture was filtered through a pad of Celite. The filtrate was added into a solution of **1** (0.2 mmol) and olefin **2** (0.3 mmol) in the mixture of *tert*-butanol (0.3 mL) and DCE (0.3 mL) at 60 ˚C, and the resulting reaction mixture was stirred under these conditions for 6.0 hours. Then, the solvent was removed under reduced pressure and the crude product was purified by column chromatography on silica gel to give corresponding products **60-62** and cycloadducts.

Characterization of the Three-component Products 59-62.

Ethyl 4-(2-((2-bromobenzyl)oxy)-2-(4-methoxyphenyl)ethyl)-1-hydroxy-3-(4-methoxyphenyl)-2-naphthoate (**59**).

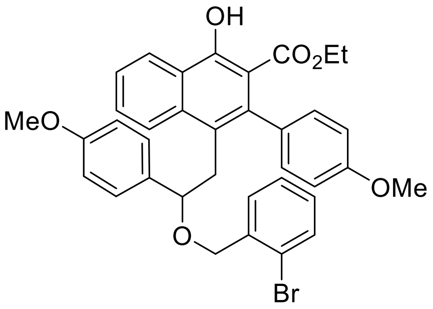

White solid; m. p. 86.0 – 88.0 ˚C. 101.4 mg, 79% yield. ^{1}H NMR (400 MHz, $CDCl_3$) (δ, ppm) 12.31 (s, 1H), 8.54 – 8.49 (m, 1H), 8.23 – 8.07 (m, 1H), 7.66 – 7.60 (m, 1H), 7.56 – 7.51 (m, 1H), 7.43 – 7.38 (m, 1H), 7.15 – 7.10 (m, 1H), 7.07 – 7.01 (comp, 2H), 6.94 – 6.86 (comp, 4H), 6.82 – 6.75 (comp, 3H), 6.64 – 6.49 (m, 1H), 4.41 (dd, *J* = 8.0, 5.6 Hz, 1H), 4.20 (dd, *J* = 85.6, 13.4 Hz, 2H), 3.93 (q, *J* = 7.1 Hz, 2H), 3.88 (s, 3H), 3.79 (s, 3H), 3.45 (dd, *J* = 14.4, 8.0 Hz, 1H), 3.08 (dd, *J* = 14.4, 5.6 Hz, 1H), 0.76 (t, *J* = 7.1 Hz, 3H). ^{13}C NMR (100 MHz, $CDCl_3$) (δ, ppm) 172.3, 159.9, 159.1, 158.4, 138.2, 138.0, 135.8, 134.7, 134.2, 132.2, 130.7, 130.6, 129.4, 129.3, 128.6, 127.6, 127.1, 125.5, 125.4, 125.0, 124.7, 124.3, 122.6, 113.8, 113.0, 113.0, 107.2, 81.9, 69.9, 61.0, 55.6, 55.4, 37.8, 13.3. HRMS (TOF MS ESI^{+}) calculated for $C_{36}H_{33}BrNaO_6^{+}$ $[M+Na]^{+}$: 663.1353, found: 663.1351.

Ethyl 4-(2-(*tert*-butoxy)-2-phenylethyl)-1-hydroxy-3-(4-methoxyphenyl)-2-naphthoate (**60**).

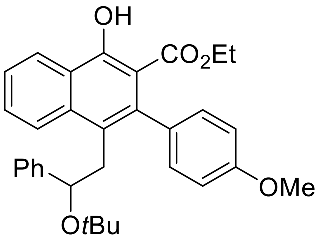

Yellow liquid. 90.6 mg, (95% yield, **60**:**14** = 87:13). ^{1}H NMR (300 MHz, $CDCl_3$) (δ, ppm) 12.36 (s, 1H), 8.54 (dd, *J* = 8.3, 1.0 Hz, 1H), 8.46 – 8.16 (br, 1H), 7.76 – 7.66 (m, 1H), 7.62 – 7.55 (m, 1H), 7.29 – 7.15 (comp, 4H), 7.09 – 6.86 (comp, 5H), 4.69 – 4.44 (m, 1H), 4.01 (q, *J* = 7.1 Hz, 2H), 3.96 (s, 3H), 3.26 (dd, *J* = 14.2, 9.2 Hz, 1H), 3.02 (dd, *J* = 14.2, 3.9 Hz, 1H), 0.84 (t, *J* = 7.2 Hz, 3H), 0.75 (s, 9H); ^{13}C NMR (75 MHz, $CDCl_3$) (δ, ppm) 172.4, 159.8, 158.6, 146.7, 137.9, 136.2, 135.1, 130.8, 130.6, 129.0, 127.9, 126.4, 126.1, 125.8, 125.3, 124.5, 123.9, 113.4, 113.2, 107.1, 74.9, 74.0, 61.0, 55.7, 39.1, 28.3, 13.3. HRMS (TOF MS ESI^{-}) calculated for $C_{32}H_{33}O_5^{-}$ $[M-H]^{-}$: 497.2333, found 479.2334.

Ethyl 4-(2-(*tert*-butoxy)-2-phenylethyl)-1-hydroxy-7-methoxy-3-phenyl-2-naphthoate (**61**).

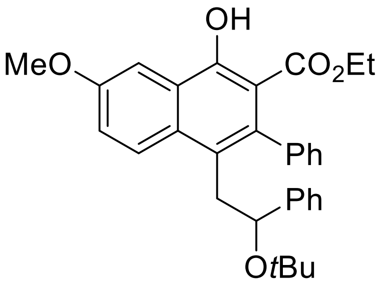

Yellow liquid. 86.3 mg, (89% yield, **61**:**9** = 81:19). ^{1}H NMR (300 MHz, $CDCl_3$) (δ, ppm) 12.30 (s, 1H), 8.43 – 7.97 (br, 1H), 7.77 (d, *J* = 2.8 Hz, 1H), 7.41 – 7.36 (comp, 3H), 7.32 – 7.25 (m, 2H), 7.16 – 7.07 (comp, 4H), 6.97 – 6.74 (br, 2H), 4.42 (d, *J* = 5.9 Hz, 1H), 4.00 (s, 3H), 3.93 (q, *J* = 7.2 Hz, 2H), 3.14 (dd, *J* = 14.2, 9.6 Hz, 1H), 2.90 (dd, *J* = 14.2, 2.7 Hz, 1H), 0.71 (t, *J* = 7.2 Hz, 4H), 0.67 (s, 9H); ^{13}C NMR (175 MHz, $CDCl_3$) (δ, ppm) 172.4, 158.7, 157.4, 146.8, 142.8, 135.9, 131.8, 129.8, 127.9, 127.7, 126.40, 126.37, 125.62, 125.55, 121.2, 107.2, 101.8, 75.1, 74.0, 61.0, 55.6, 39.3, 28.3, 13.1. HRMS (TOF MS ESI^{-}) calculated for $C_{32}H_{33}O_5^{-}$ $[M-H]^{-}$: 497.2333, found 479.2334.

Ethyl 4-(2-(*tert*-butoxy)-2-(4-chlorophenyl)ethyl)-1-hydroxy-3-(4-methoxyphenyl)-2-naphthoate (**62**).

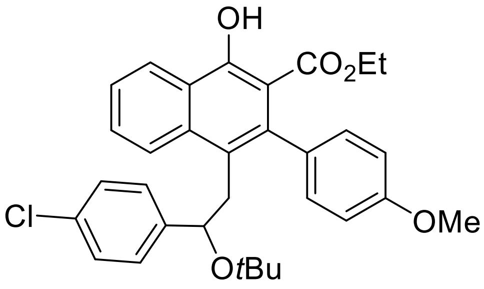

Yellow liquid. 91.0 mg, (90% yield, **62**:**25** = 66:34). ^{1}H NMR (300 MHz, $CDCl_3$) (δ, ppm) 12.36 (s, 1H), 8.54 (dd, *J* = 8.3, 0.9 Hz, 1H), 8.45 – 8.05 (br, 1H), 7.75 – 7.64 (m, 1H), 7.60 – 7.54 (m, 1H), 7.26 – 7.23 (m, 1H), 7.17 – 7.13 (m, 2H), 7.02 – 6.95 (m, 2H), 6.95 – 6.81 (br, 3H), 4.63 – 4.42 (m, 1H), 4.00 (q, *J* = 7.2 Hz, 2H), 3.94 (s, 3H), 3.24 (dd, *J* = 14.2, 8.9 Hz, 1H), 2.99 (dd, *J* = 14.2, 4.1 Hz, 1H), 0.82 (t, *J* = 7.2 Hz, 3H), 0.75 (s, 9H); ^{13}C NMR (175 MHz, $CDCl_3$) (δ, ppm) 172.3, 159.9, 158.6, 145.2, 137.8, 136.1, 134.9, 132.0, 130.5, 129.1, 128.0, 125.4, 124.5, 124.0, 113.4, 113.2, 107.1, 74.4, 74.2, 61.0, 55.7, 39.0, 28.3, 13.3. HRMS (TOF MS ESI^-) calculated for $C_{32}H_{32}ClO_5^-$ $[M-H]^-$: 531.1944, found 531.1945.

Control Experiment with Diazo Compound 1aa and Styrene:

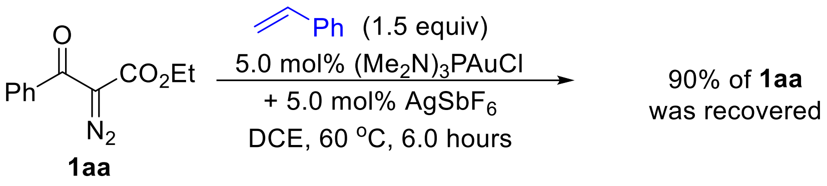

To a 10-mL oven-dried vial containing a magnetic stirring bar, $(Me_2N)_3PAuCl$ (3.95 mg, 0.01 mmol), $AgSbF_6$ (3.43 mg, 0.01 mmol), and DCE (0.5 mL) were added in sequence in a nitrogen-filled glove-box. The reaction mixture was stirred at 25 ˚C for 2.0 hours. The solvent was removed and the residue was dissolved in DCE (0.5 mL). Then the mixture was filtered through a pad of Celite. The filtrate was added into a solution of **1aa** (43.6 mg, 0.2 mmol) and styrene (31.2 mg, 0.3 mmol) in DCE (0.5 mL) at 60 ˚C, and the resulting reaction mixture was stirred under these conditions for 6.0 hours. Then, the solvent was removed under reduced pressure and the crude product was purified by column chromatography on silica gel (solvents: petroleum ether/ethyl acetate = 10: 1) to recover 39.3 mg of **1aa** (90%).

^{31}P NMR Spectra of Gold-complex with 1aa, A, or 1l:

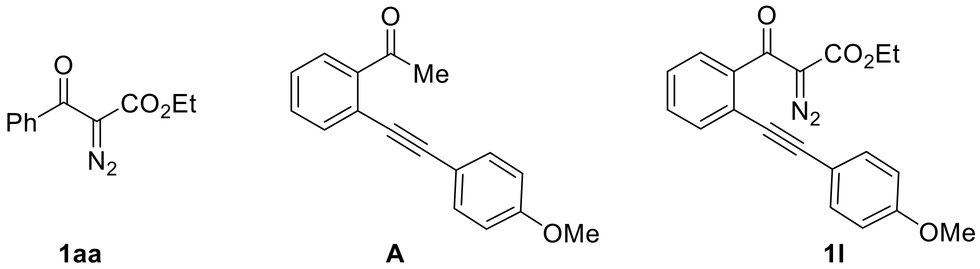

In order to explore the initiation step of this gold-catalyzed [4+2] cycloaddition, diazo compound **1aa** without the alkyne species, internal alkyne **A**, and **1l** were selected to mix with the gold catalyst. Considering the stability of the catalyst, $JohnphosAu(CH_3CN)SbF_6$ was chosen as the catalyst.

<u>*Experimental procedure*</u>: To a dried NMR tube, **1aa** (43.6 mg, 0.2 mmol) in $CDCl_3$ (1.0 mL) was added $JohnphosAu(CH_3CN)SbF_6$ (7.6 mg, 0.01 mmol, 5.0 mol%). Then the mixture was subjected to ^{31}P NMR analysis after 3.5 minutes at 25 ˚C (Figure S2-1). The experimental procedure with **A** (Figure S2-2), with **1aa** and **A** (Figure S2-3), or with **1l** (Figure S2-4) were similar to that of **1aa** (Figure S5-1). For the ^{31}P NMR of $JohnphosAu(CH_3CN)SbF_6$, see Figure S2-5. In all these ^{31}P NMR spectra, the external 85% phosphoric acid was used as ^{31}P standard.

These results implied that the gold catalyst first coordinated with the alkynyl group, instead of direct decomposition of the diazo species.

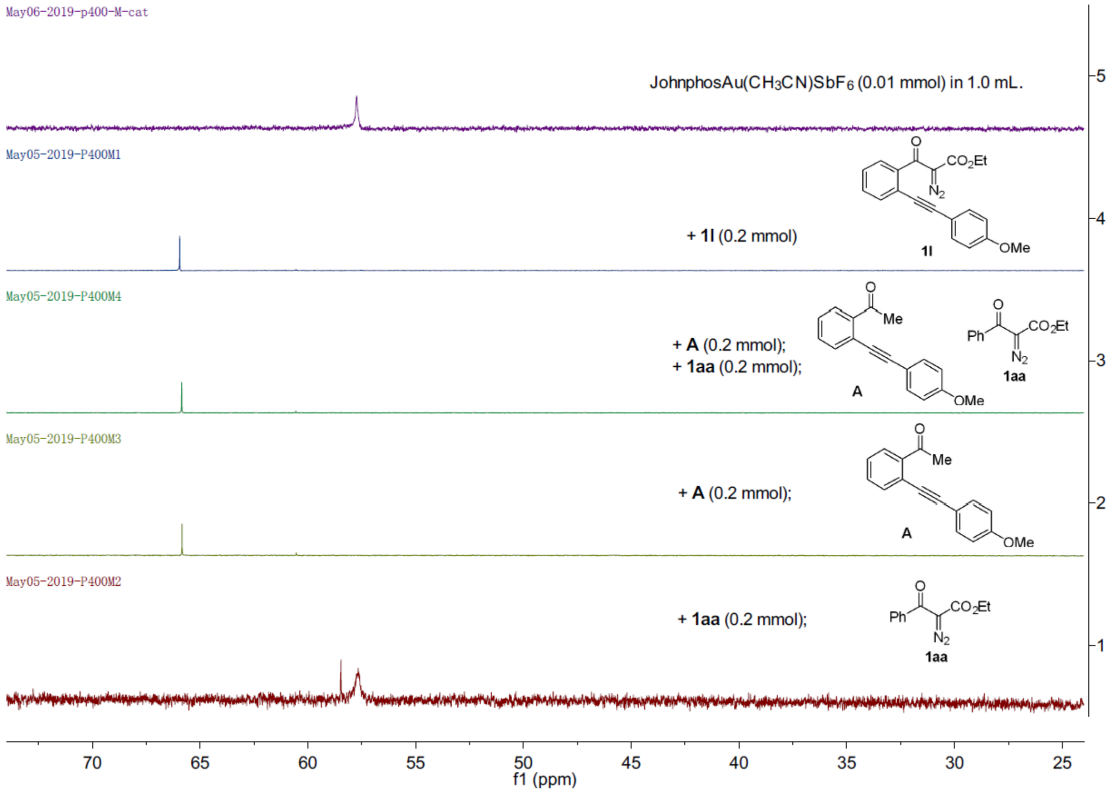

Supplementary Figure 2. The ^{31}P NMR spectra of gold catalyst with **1l**, **A**, or **1aa**, separately or in combination.

Synthetic Applications of Current Strategy

Synthesis of 63

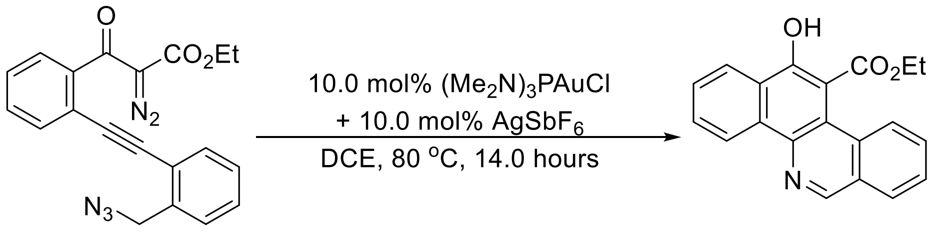

1t **63**, 91 % yield

To a 10-mL oven-dried vial containing a magnetic stirring bar, $(Me_2N)_3PAuCl$ (3.95 mg, 0.01 mmol), $AgSbF_6$ (3.43 mg, 0.01 mmol), and DCE (0.5 mL) were added in sequence in a nitrogen-filled glove-box. The reaction mixture was stirred at 25 ˚C for 2.0 hours. The solvent was removed and the residue was dissolved in DCE (0.5 mL). Then the mixture was filtered through a pad of Celite. The filtrate was added into a solution of **1t** (74.6 mg, 0.2 mmol) in DCE (0.5 mL) at 80 ˚C, and the resulting reaction mixture was stirred under these conditions for 14 hours. Then, the solvent was removed under reduced pressure and the crude product was purified by column chromatography on silica gel (solvents: petroleum ether/ethyl acetate = 5: 1) to give 57.8 mg **63** in 91% yield. White solid; m. p. 221.0 – 223.0 ˚C. ^1H NMR (400 MHz, $CDCl_3$) (δ, ppm) 11.34 (s, 1H), 9.46 – 9.10 (comp, 2H), 8.50 (d, J = 8.1 Hz, 1H), 8.06 (d, J = 7.3 Hz, 1H), 7.98 (d, J = 8.0 Hz, 1H), 7.92 – 7.79 (m, 1H), 7.78 – 7.55 (comp, 3H), 4.40 (q, J = 7.1 Hz, 2H), 1.19 (t, J = 7.1 Hz, 3H); ^{13}C NMR (100 MHz, $CDCl_3$) (δ, ppm) 171.9, 159.7, 150.3, 137.6, 134.9, 132.3, 130.6, 128.4, 127.9, 127.8, 127.7, 126.9, 125.5, 124.9, 124.1, 119.6, 102.2, 61.9, 13.9. HRMS (TOF MS ESI^+) calculated for $C_{20}H_{15}NNaO_3^+$ $[M+Na]^+$: 340.0944, found: 340.0947.

Synthesis of 64

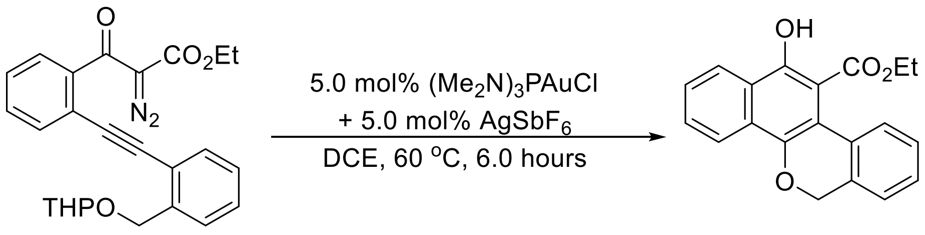

1u **64**, 94 % yield

To a 10-mL oven-dried vial containing a magnetic stirring bar, $(Me_2N)_3PAuCl$ (3.95 mg, 0.01 mmol), $AgSbF_6$ (3.43 mg, 0.01 mmol), and DCE (0.5 mL) were added in sequence in a nitrogen-filled glove-box. The reaction mixture was stirred at 25 ˚C for 2.0 hours. The solvent was removed and the residue was dissolved in DCE (0.5 mL). Then the mixture was filtered through a pad of Celite. The filtrate was added into a solution of **1u** (86.4 mg, 0.2 mmol) in DCE (0.5 mL) at 60 ˚C, and the resulting reaction mixture was stirred under these conditions for 6.0 hours. Then, the solvent was removed under reduced pressure and the crude product was purified by column chromatography on silica gel (solvents: petroleum ether/ethyl acetate = 10: 1) to give 60.2 mg **64** in 94% yield. ^1H NMR (400 MHz, $CDCl_3$) (δ, ppm) 11.30 (s, 1H), 8.45 – 8.27 (m, 1H), 8.26 – 8.03 (m, 1H), 7.65 – 7.58 (m, 1H), 7.58 – 7.52 (m, 1H), 7.31 – 7.26 (m, 1H), 7.26 – 7.23 (comp, 2H), 7.15 (d, J = 7.3 Hz, 1H), 5.16 (s, 2H), 4.32 (q, J = 7.1 Hz, 2H), 1.16 (t, J = 7.1 Hz, 3H); ^{13}C NMR (100 MHz, $CDCl_3$) (δ, ppm) 171.6, 156.1, 145.8, 131.0, 130.5, 129.7, 128.4, 127.4, 127.3, 127.2, 126.7, 125.7, 124.6, 124.3, 122.2, 115.1, 102.8, 69.5, 61.6, 13.9. HRMS (TOF MS ESI^+) calculated for $C_{20}H_{16}NaO_4^+$ $[M+Na]^+$: 343.0941, found: 343.0947. The analysis of NMR is consistent with the literature.[4]

General Procedure of the Scale Up and Synthesis of 65-68.

Gram scale reaction for the synthesis of 14.

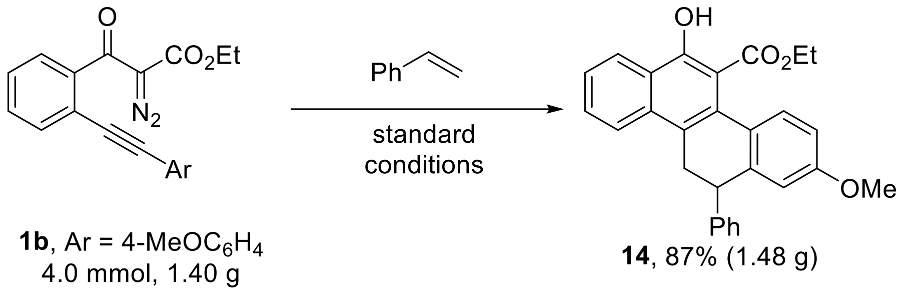

To a 10-mL oven-dried vial containing a magnetic stirring bar, $(Me_2N)_3PAuCl$ (79 mg, 0.2 mmol), $AgSbF_6$ (68.63 mg, 0.2 mmol), and DCE (10 mL) were added in sequence in a nitrogen-filled glove-box. The reaction mixture was stirred at 25 ˚C for 2.0 hours. The solvent was removed and the residue was dissolved in DCE (10 mL). Then the mixture was filtered through a pad of Celite. The filtrate was added into a solution of **1b** (1.40 g, 4.0 mmol) and **2a** (0.624 g, 6.0 mmol) in DCE (10 mL) at 60 ˚C, and the resulting reaction mixture was stirred under these conditions for 6.0 hours. Then, the solvent was removed under reduced pressure and the crude product was purified by column chromatography on silica gel (eluent: Ethyl acetate/light petroleum ether =1/30~1/10) to afford 1.48 g **14** in 87% yield.

Synthesis of 65

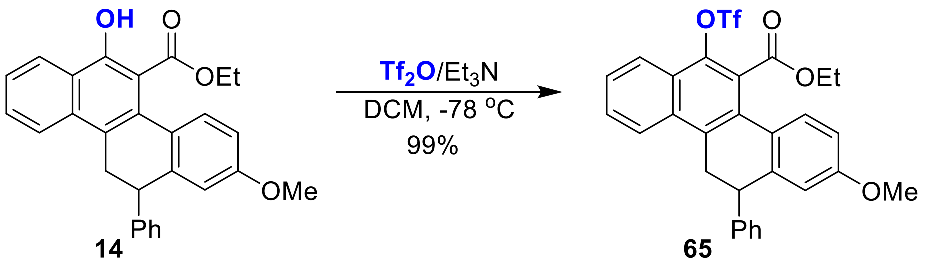

To a 10-mL oven-dried vial containing a magnetic stirring bar, triethylamine (Et_3N, 253.0 µL, 1.5 mmol), and **14** (424.5 mg, 1.0 mmol) in dry dichloromethane (DCM, 3.0 mL), was added trifluoromethanesulfonic anhydride (Tf_2O, 208.0 µL, 1.5 mmol) in 10 minutes *via* a syringe at -78 ˚C. The reaction mixture was warmed to room temperature slowly in 3.0 hours. Until **14** was completely consumed (determined by TLC), the solvent was removed under reduced pressure to afford the crude product, which was purified by flash column chromatography on silica gel (eluent: ethyl acetate/hexane = 1:100) to give 551.0 mg pure product **65** in 99% yield. ^1H NMR (300 MHz, $CDCl_3$) (δ, ppm) 8.21 – 8.05 (comp, 2H), 7.65 – 7.56 (comp, 2H), 7.43 (d, *J* = 8.6 Hz, 1H), 7.38 – 7.26 (comp, 5H), 6.82 (dd, *J* = 8.6, 2.2 Hz, 1H), 6.50 (d, *J* = 2.2 Hz, 1H), 4.44 – 4.22 (comp, 3H), 3.74 (s, 3H), 3.51 (ddd, *J* = 26.6, 15.8, 8.0 Hz, 2H), 1.24 (t, *J* = 7.2 Hz, 3H); ^{13}C NMR (125 MHz, $CDCl_3$) (δ, ppm) 166.7, 159.7, 142.4, 141.84, 141.78, 134.5, 133.2, 130.6, 128.9, 128.83, 128.79, 128.1, 127.7, 127.2, 126.6, 125.3, 123.6, 123.2, 122.8, 113.5, 111.8, 62.5, 55.4, 44.5, 33.1, 13.8; ^{19}F NMR (283 MHz, $CDCl_3$) (δ, ppm) -72.9. HRMS (TOF MS ESI^+) calculated for $C_{29}H_{24}F_3O_6S^+$ $[M+H]^+$: 557.1240, found 557.1237.

Synthesis of 66

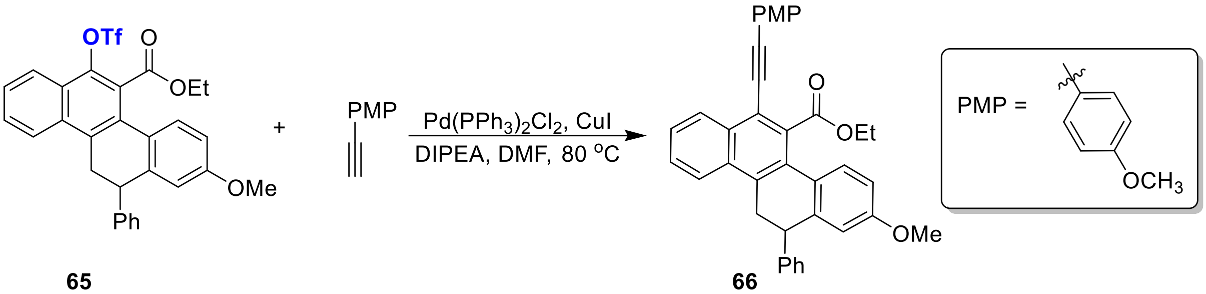

65 **66**

To a 10-mL oven-dried vial containing a magnetic stirring bar, **65** (55.6 mg, 0.1 mmol), 4-ethynylanisole (20.0 mg, 0.15 mmol), $Pd(PPh_3)_2Cl_2$ (7.0 mg, 0.01 mmol), CuI (1.0 mg, 0.005 mmol), diisopropylethylamine (DIPEA, 52.0 μL, 0.3 mmol), and DMF (1.0 mL) were added in sequence under argon atmosphere. The reaction was stirred at 80 ˚C for 12.0 hours until **65** was completely consumed (determined by TLC). The reaction mixture was purified by flash column chromatography on silica gel without additional treatment (eluent: ethyl acetate/hexane = 1:40) to give 50.0 mg **66** in 93% yield. White solid; m. p. 190.0 – 192.0 ˚C. ^{1}H NMR (500 MHz, $CDCl_3$) (δ, ppm) 8.51 (d, *J* = 7.5 Hz, 1H), 8.06 (d, *J* = 8.0 Hz, 1H), 7.64 (d, *J* = 8.6 Hz, 1H), 7.59 (d, *J* = 8.3 Hz, 2H), 7.57 – 7.51 (comp, 2H), 7.33 – 7.27 (comp, 4H), 7.26 – 7.23 (m, 1H), 6.94 (d, *J* = 8.3 Hz, 2H), 6.84 (d, *J* = 8.6 Hz, 1H), 6.54 (s, 1H), 4.52 – 4.39 (m, 2H), 4.25 (dd, *J* = 9.5, 4.8 Hz, 1H), 3.86 (s, 3H), 3.75 (s, 3H), 3.61 (dd, *J* = 15.6, 4.8 Hz, 1H), 3.50 (dd, *J* = 15.6, 10.1 Hz, 1H), 1.35 (t, *J* = 7.1 Hz, 3H); ^{13}C NMR (125 MHz, $CDCl_3$) (δ, ppm) 170.4, 160.0, 159.4, 142.31, 142.29, 133.8, 133.3, 132.8, 131.8, 131.7, 129.5, 128.73, 128.70, 127.8, 127.73, 127.68, 127.2, 126.9, 126.8, 123.8, 119.4, 115.5, 114.2, 113.6, 111.9, 98.6, 84.2, 61.8, 55.5, 55.3, 44.7, 33.0, 14.3. HRMS (TOF MS ESI$^+$) calculated for $C_{37}H_{31}O_4^+$ [M+H]$^+$: 539.2217, found: 539.2210.

Synthesis of 67

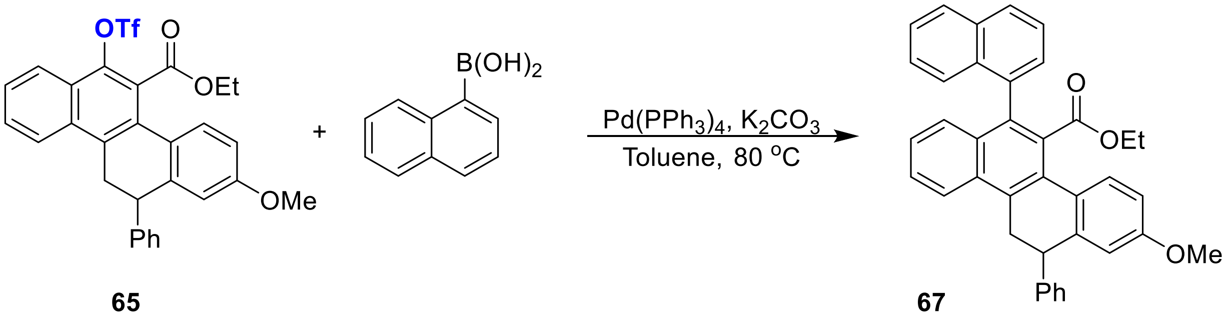

65 **67**

To a 10-mL oven-dried vial containing a magnetic stirring bar, **65** (111.2 mg, 0.2 mmol), 1-naphthylboronic acid (51.6 mg, 0.3 mmol), $Pd(PPh_3)_4$ (23.1 mg, 0.02 mmol), K_2CO_3 (55.3mg, 0.4 mmol), and toluene (2.0 mL) were added in sequence under argon atmosphere. The reaction was stirred at 80 ˚C for 6.0 hours until **65** was completely consumed (determined by TLC). The reaction mixture was purified by flash column chromatography on silica gel without additional treatment (eluent: ethyl acetate/hexane = 1:40) to give 104.8 mg **67** in 98% yield. White solid; m. p. 217.5 – 219.5 ˚C. ^{1}H NMR (500 MHz, $CDCl_3$) (δ, ppm) with three diastereoisomers: 8.17 – 6.48 (comp, 17H), 4.42 – 3.44 (comp, 8H), 1.31 – 0.52 (comp, 3H), ^{13}C NMR (125 MHz, $CDCl_3$) (δ, ppm) with three diastereoisomers: (170.9, 170.28, 170.25), (159.23, 159.22, 159.17), 143.0, 142.6, 142.52, 142.48, 142.2, 141.7, 136.4, 136.10, 136.07, 135.9, 133.7, 133.6, 133.5, 133.4, 133.32, 133.30, 132.9, 132.6, 131.8, 131.7, 131.6, 131.5, 130.8, 130.6, 130.4, 129.6, 129.4, 129.27, 129.26, 129.1, 128.81, 128.79, 128.76, 128.7, 128.63, 128.58, 128.3, 128.2, 128.1, 128.04, 128.01, 127.99, 127.97, 127.8, 127.7, 127.44, 127.41, 127.21, 127.20, 127.10, 127.0, 126.9, 126.8, 126.2, 126.12, 126.05, 125.94,

125.89, 125.3, 125.2, (123.63, 123.56, 123.5), (113.7, 113.5, 113.2), (111.8, 111.6, 111.3), (61.5, 60.8, 60.7), (55.32, 55.29, 55.2), (45.2, 44.8, 44.7), (33.1, 33.0, 32.8), (14.2, 13.3, 13.2). HRMS (TOF MS ESI$^+$) calculated for $C_{38}H_{31}O_3^+$ [M+H]$^+$: 535.2268, found: 535.2260.

Synthesis of 68

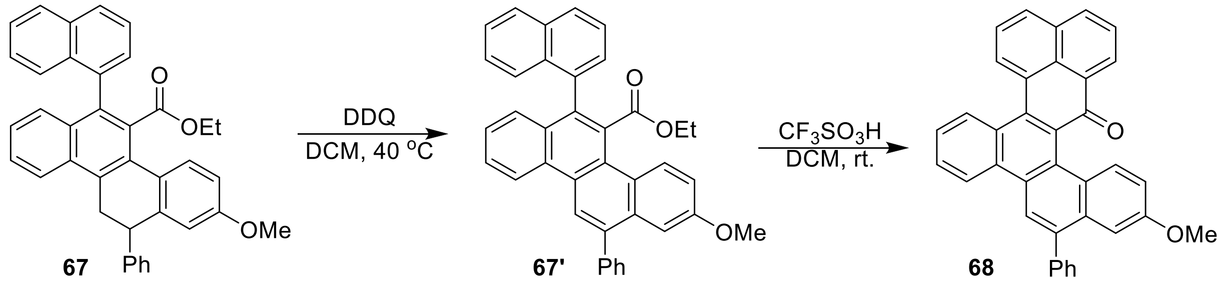

To a 10-mL oven-dried flask equipped with a magnetic stirring bar, **67** (53.5 mg, 0.1 mmol), DDQ (45.4 mg, 0.20 mmol), and DCM (3.0 mL) were added in sequence. The reaction mixture was stirred at 40 ˚C for 12 hours. Upon completion (monitored by TLC), the solvent was evaporated under vacuum after filtering through a pad of Celite. The obtained crude **67'** was directly used for the next step without further purification.

To a 10-mL oven-dried round-bottom flask containing a magnetic stirring bar, the above obtained **67'**, trifluoromethanesulfonic acid (87.0 μL, 1.0 mmol), and DCM (1.0 mL) were added in sequence under argon at 25 ˚C. Then the reaction mixture was stirred for 12 hours. Then, water (50 mL) was added to the reaction mixture and stirred for 1-2 hours. The yellow solid precipitated out and was filtered under vacuum. The crude product was purified by column chromatography on silica gel (ethyl acetate/petroleum ether = 1/3) to give 39.9 mg **68** in 82% yield based on **67**. Yellow solid; m. p. 279.0 – 281.0 ˚C. ^{1}H NMR (500 MHz, $Cl_2CDCDCl_2$) (δ, ppm) 9.70 (s, 1H), 9.04 (d, *J* = 7.6 Hz, 1H), 8.83 (d, *J* = 5.7 Hz, 1H), 8.72 (d, *J* = 6.4 Hz, 1H), 8.67 (d, *J* = 8.6 Hz, 1H), 8.59 (s, 1H), 8.47 (d, *J* = 7.9 Hz, 1H), 8.06 (d, *J* = 5.1 Hz, 1H), 7.97 (d, *J* = 7.9 Hz, 1H), 7.91 (t, *J* = 6.7 Hz, 1H), 7.78 – 7.71 (comp, 2H), 7.69 – 7.56 (comp, 5H), 7.47 – 7.36 (comp, 2H), 3.86 (s, 3H); ^{13}C NMR (125 MHz, $Cl_2CDCDCl_2$) (δ, ppm) 183.6, 158.2, 140.7, 140.4, 137.0, 136.7, 132.6, 129.9, 129.8, 129.5, 129.4, 129.0, 128.7, 128.6, 128.2, 128.1, 128.0, 127.9, 127.8, 127.1, 127.02, 126.96, 126.9, 126.6, 125.5, 124.8, 124.5, 122.8, 122.3, 117.9, 107.5, 55.4. HRMS (TOF MS ESI$^+$) calculated for $C_{36}H_{23}O_2^+$ [M+H]$^+$: 487.1693, found 487.1676.

Optical and Photophysical Properties

The optical and photophysical properties of these obtained π-conjugated polycyclic hydrocarbons (CPHs) **45**, **47**, **48**, **52**, and **53** were examined in DMSO solution (0.2–0.3 mg/L, in quartz cuvettes with a layer thickness of 1 cm). The UV-vis absorption spectra were recorded on a Shimadzu UV-1800 spectrophotometer. The f and excitation spectra were measured on a Shimadzu RF-5301PC spectroscope (Supplementary Figure 3).

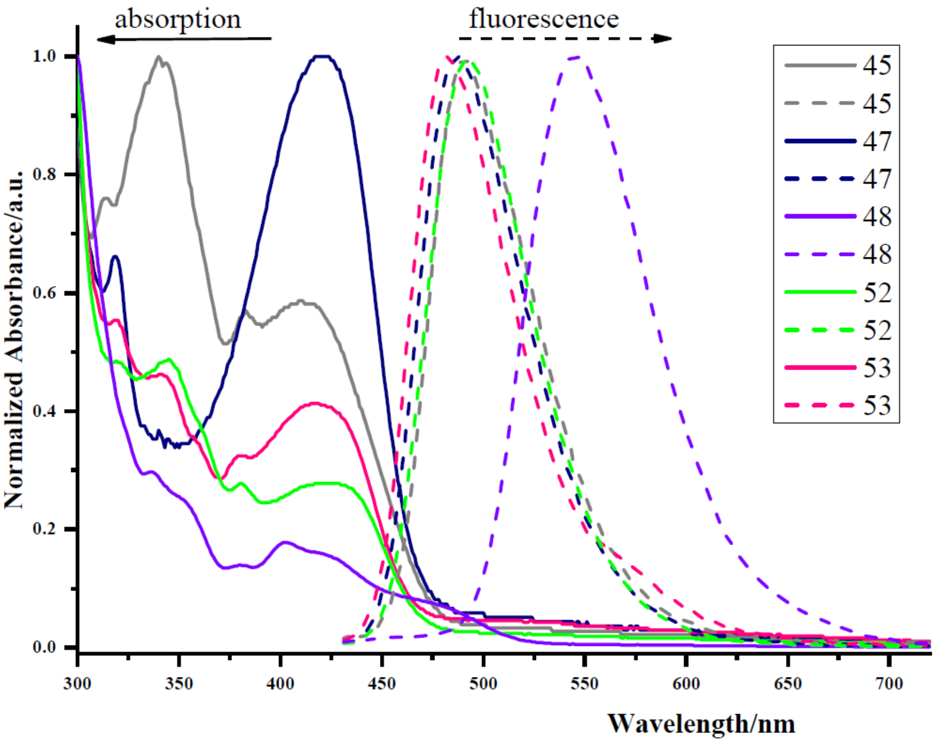

Supplementary Figure 3. The UV/Vis absorption (solid lines) and emission spectra (broken lines) in DMSO.

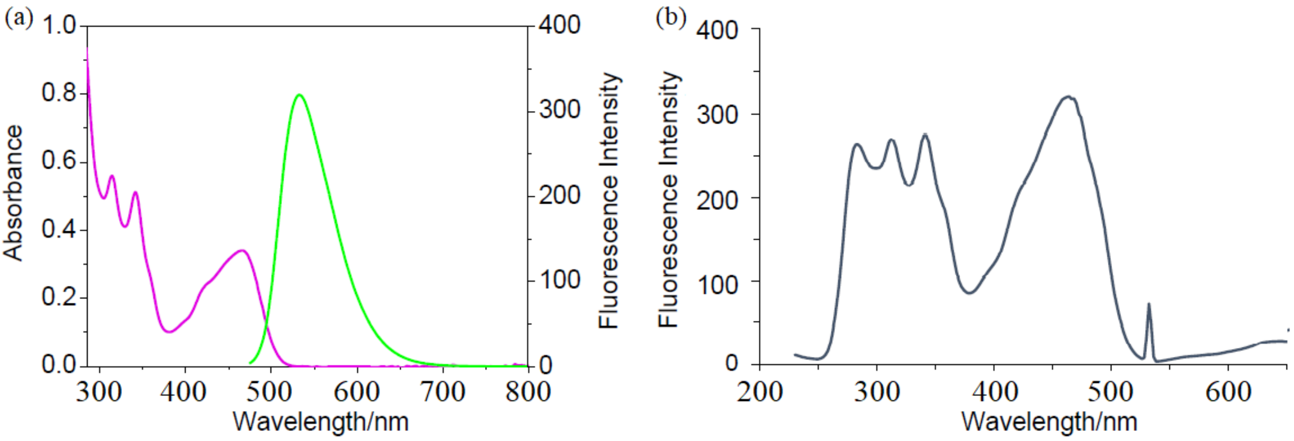

Supplementary Figure 4. **(a)** UV/Vis absorption and fluorescence emission spectra of compound **68** in TCE at room temperature. **(b)** Fluorescence excitation spectra of compound **68** in TCE at room temperature. λ_{ex} = 465 nm; λ_{em} = 533 nm; bandwidth: 3 nm.

NMR Spectra of New Compounds

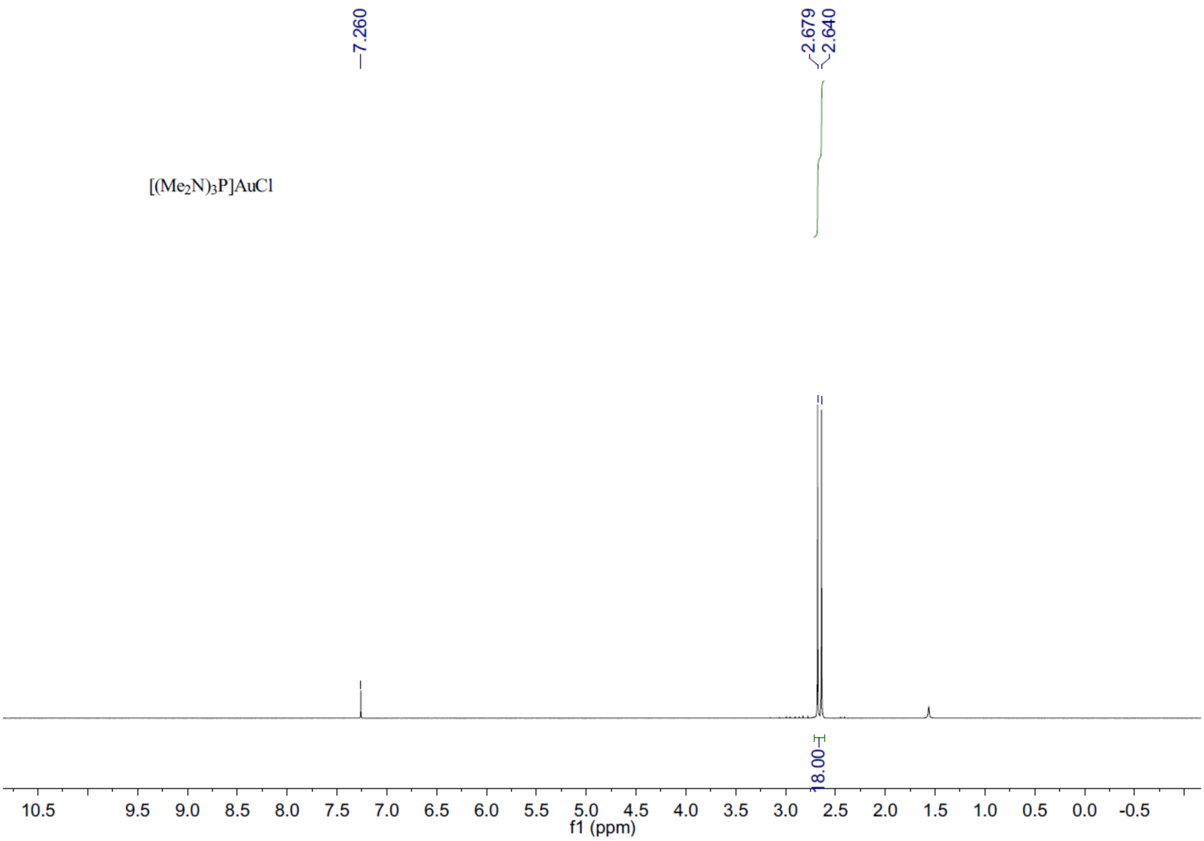

Supplementary Figure 5. ^{1}H NMR (300 MHz, $CDCl_3$) spectrum for compound $(Me_2N)_3PAuCl$.

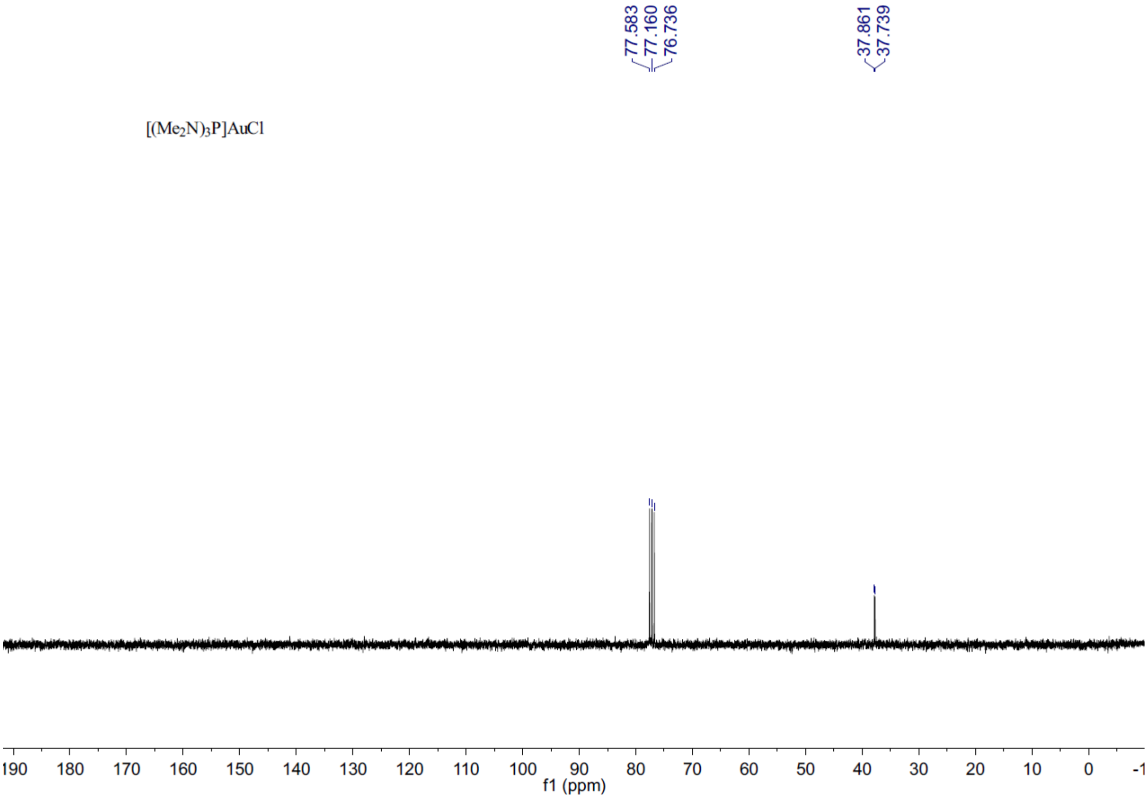

Supplementary Figure 6. ^{13}C NMR (75 MHz, $CDCl_3$) spectrum for compound $(Me_2N)_3PAuCl$.

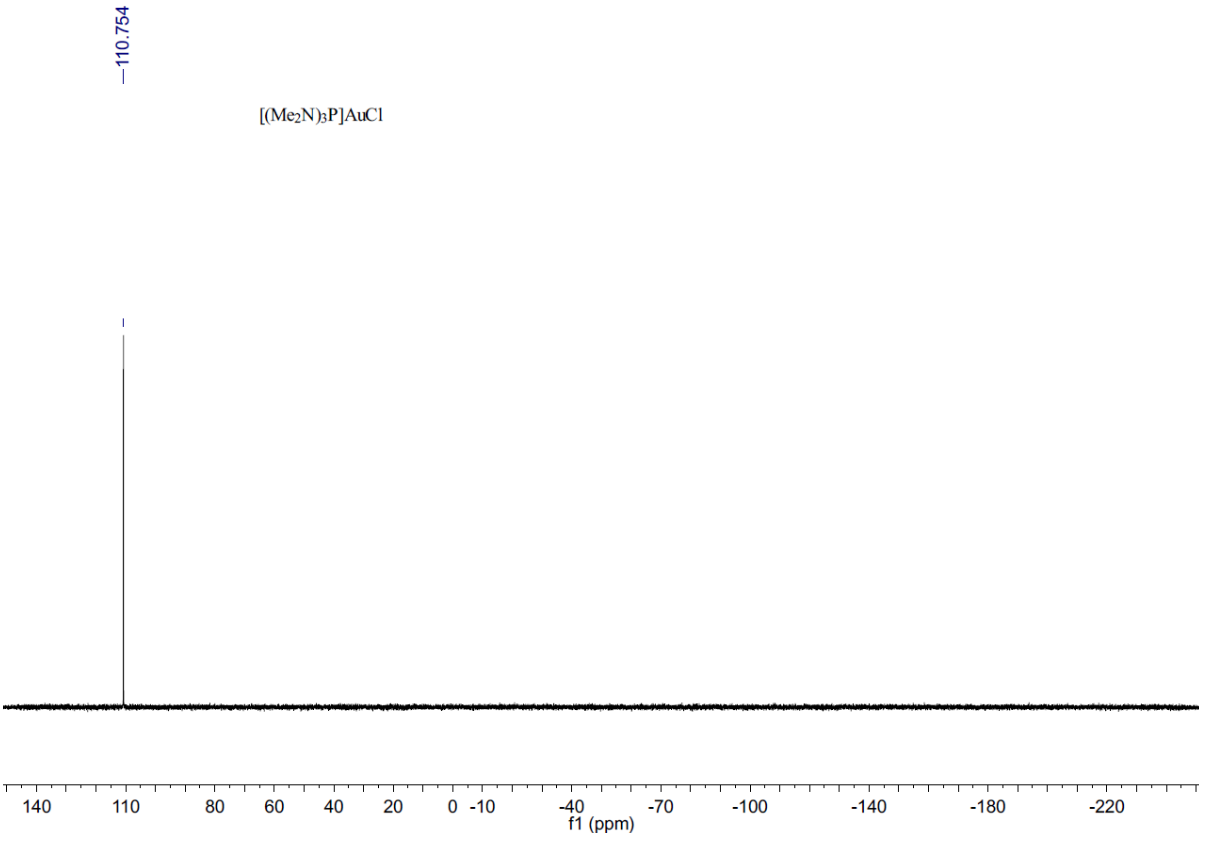

Supplementary Figure 7. ^{13}C NMR (75 MHz, $CDCl_3$) spectrum for compound $(Me_2N)_3PAuCl$.

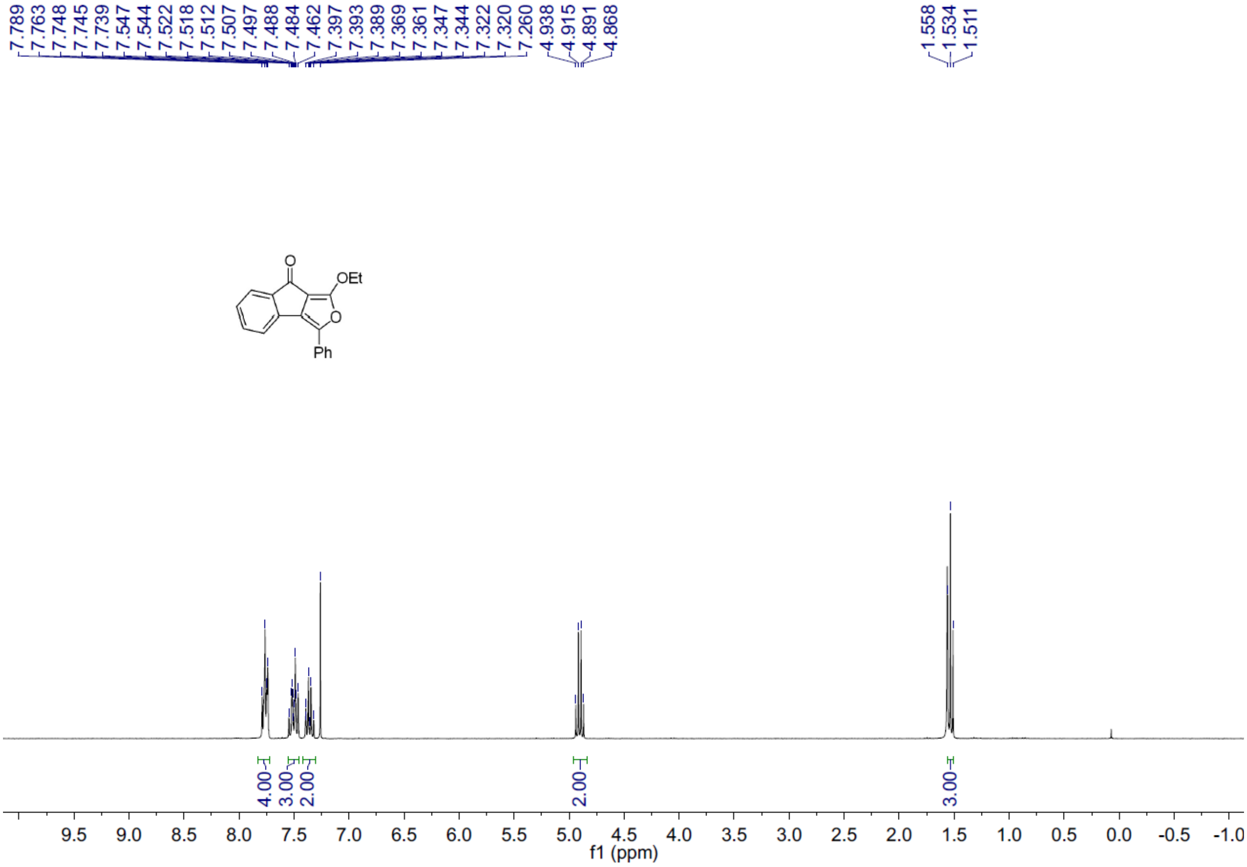

Supplementary Figure 8. ^{1}H NMR (300 MHz, $CDCl_3$) spectrum for compound 3’.

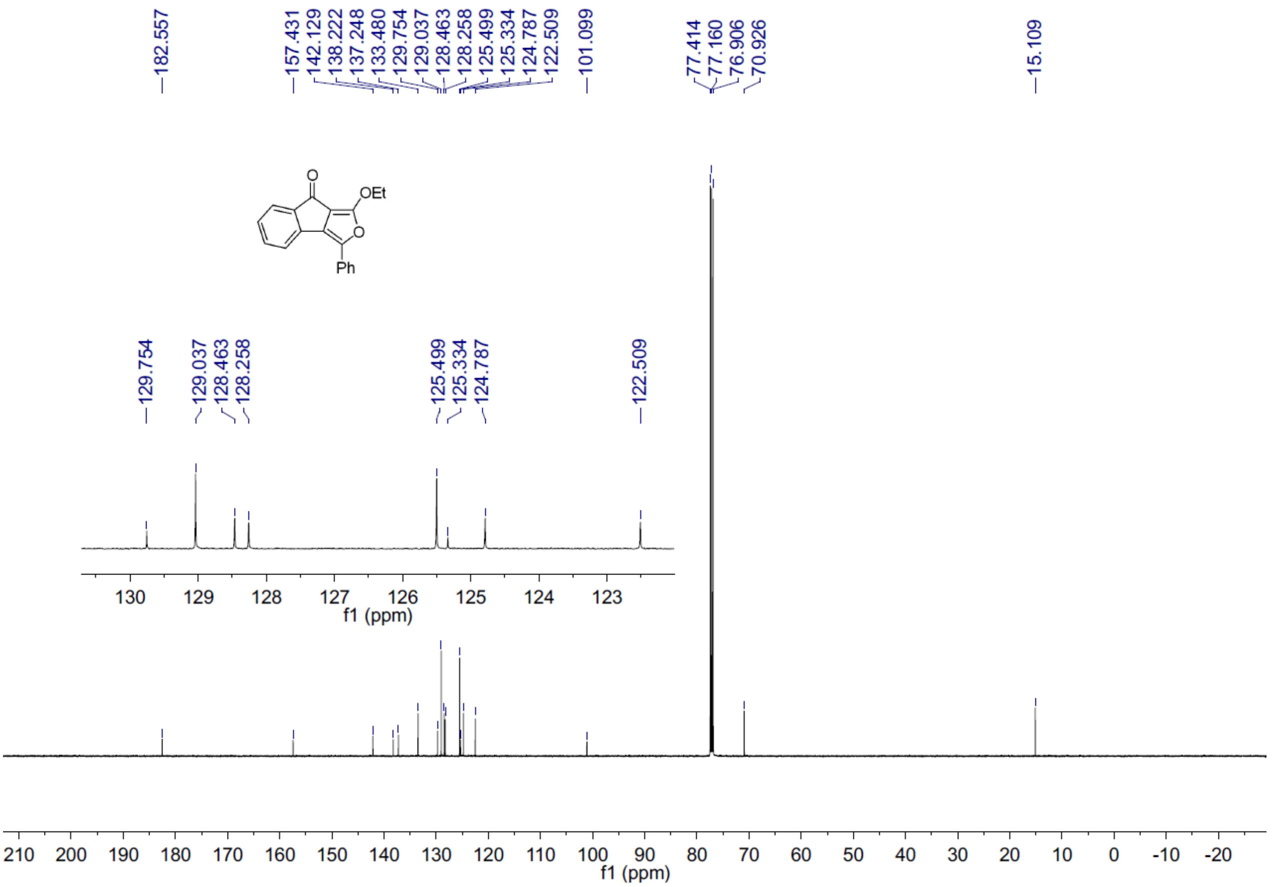

Supplementary Figure 9. ^{13}C NMR (125 MHz, $CDCl_3$) spectrum for compound 3'.

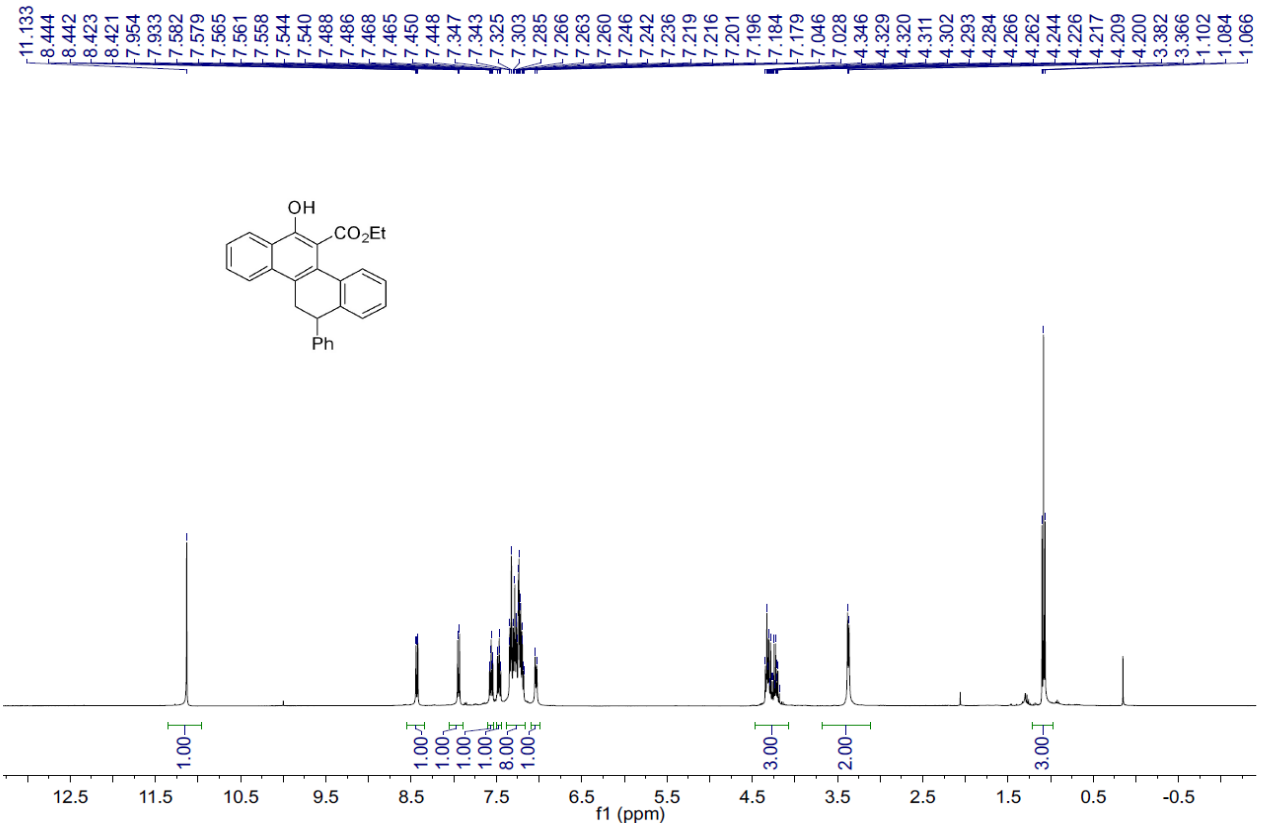

Supplementary Figure 10. ^{1}H NMR (400 MHz, $CDCl_3$) spectrum for compound 3.

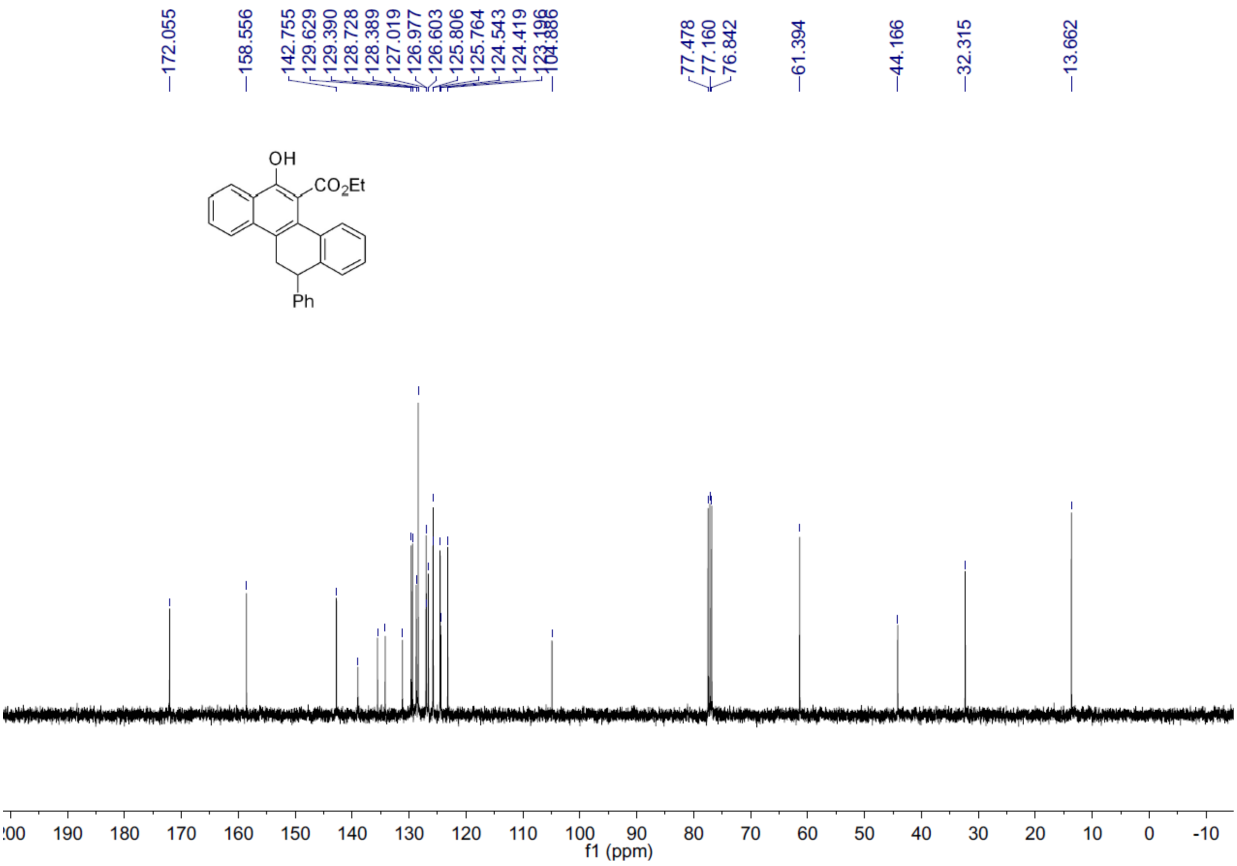

Supplementary Figure 11. ^{13}**C NMR (100 MHz, CDCl$_3$) spectrum for compound 3.**

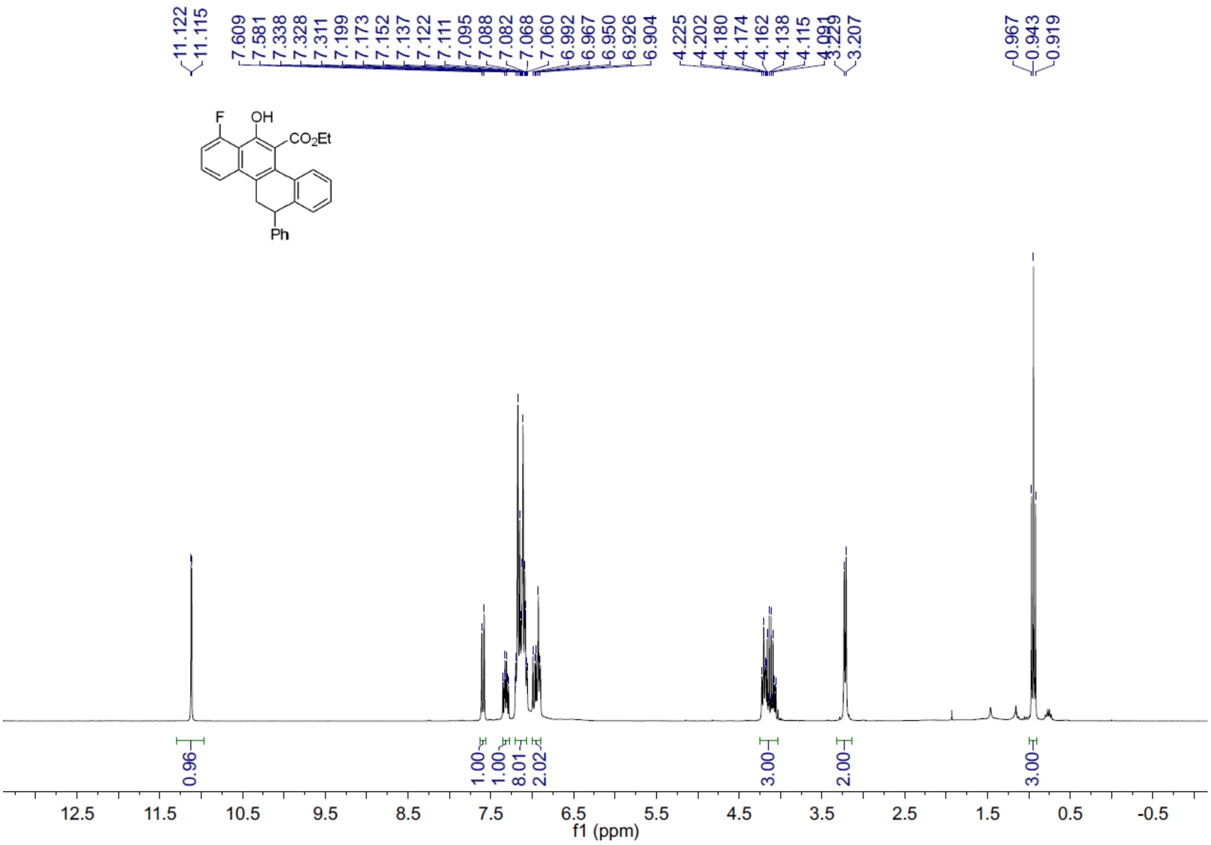

Supplementary Figure 12. ^{1}**H NMR (300 MHz, CDCl$_3$) spectrum for compound 4.**

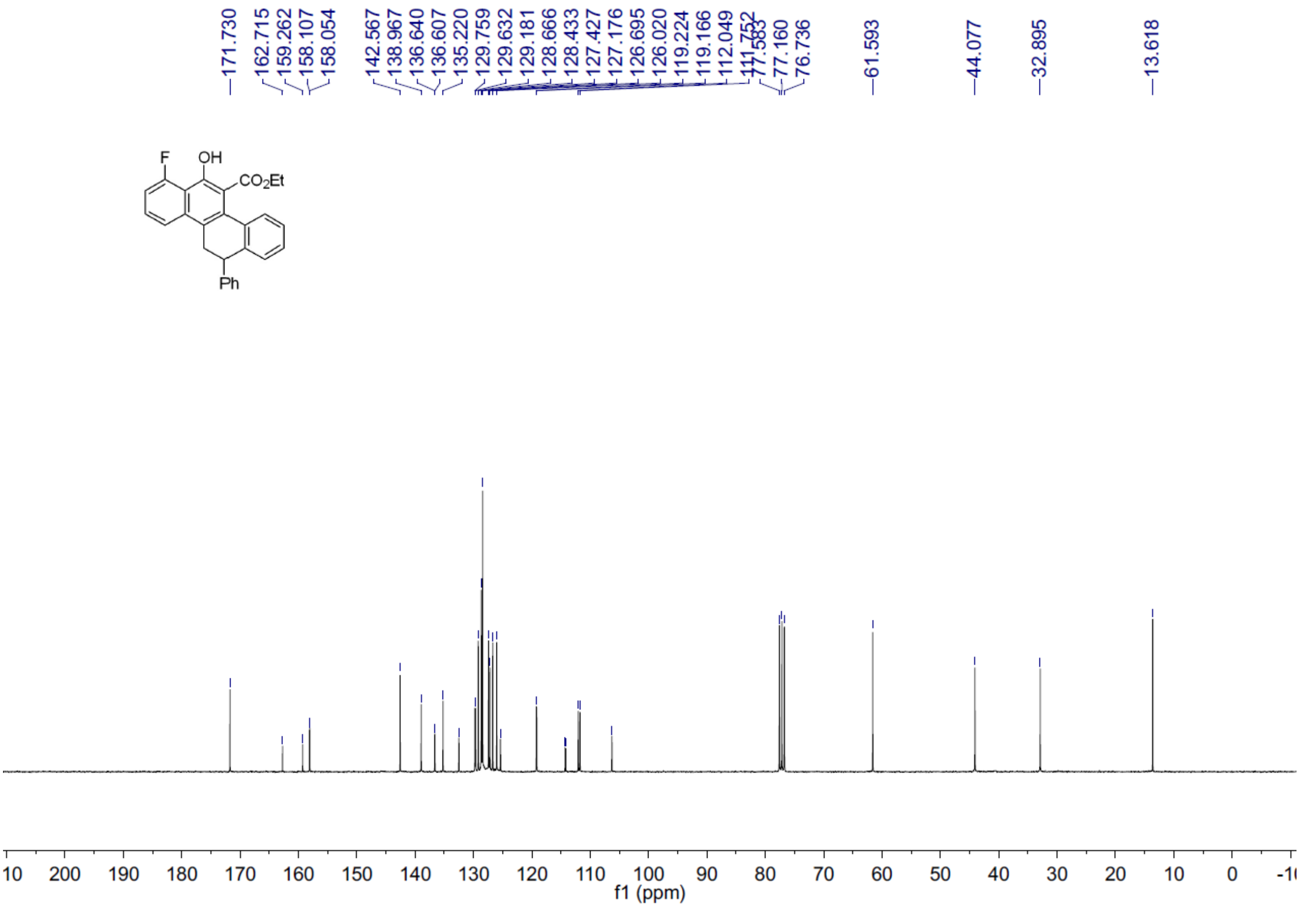

Supplementary Figure 13. ^{13}C NMR (75 MHz, $CDCl_3$) spectrum for compound 4.

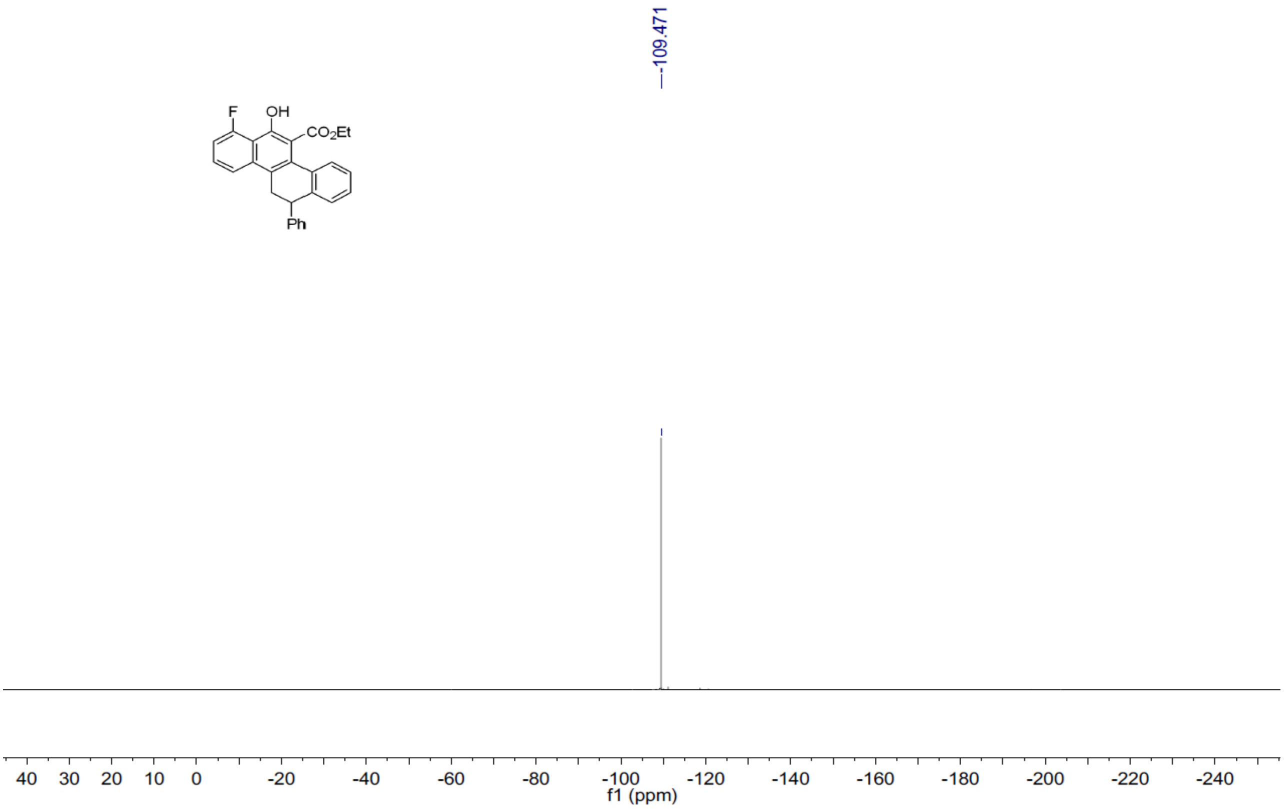

Supplementary Figure 14. ^{19}F NMR (283 MHz, $CDCl_3$) spectrum for compound 4.

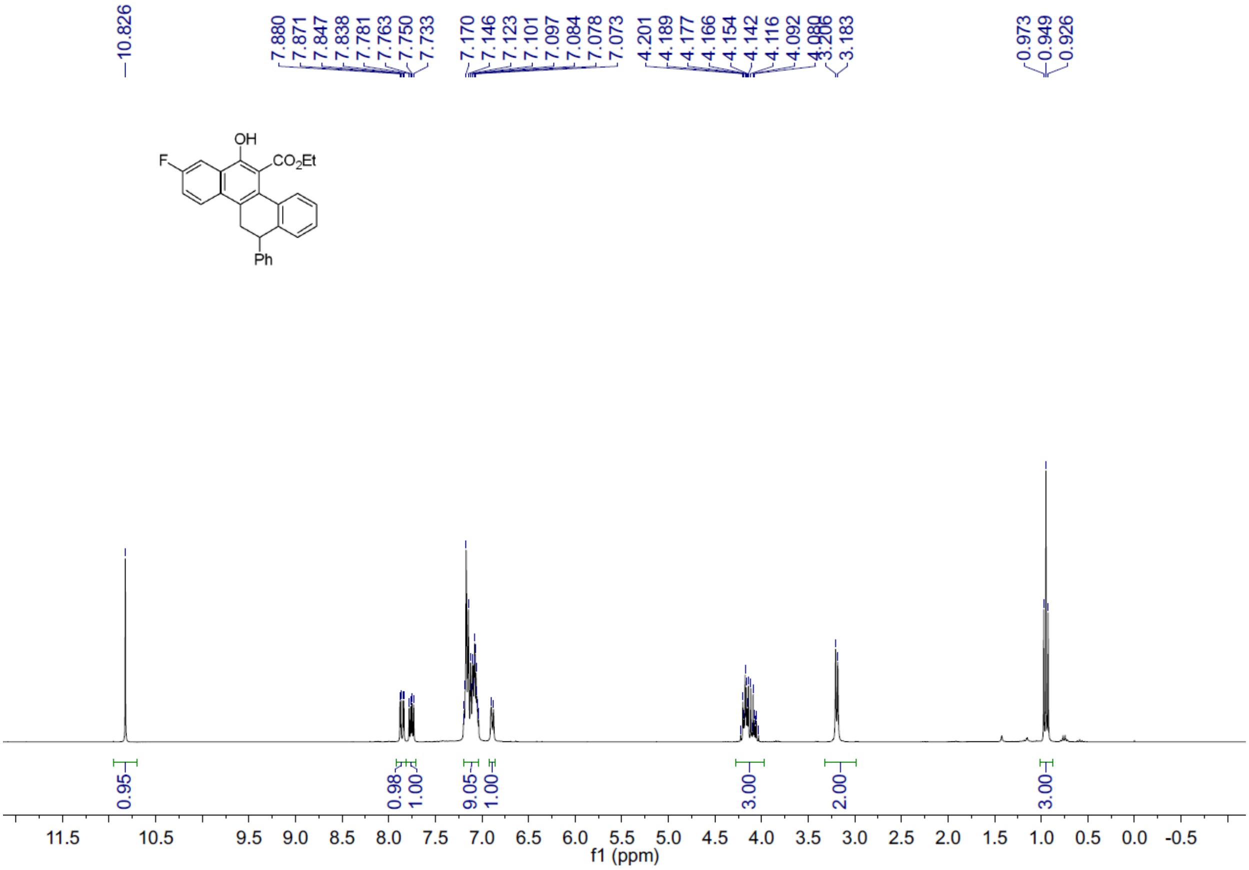

Supplementary Figure 15. ^{1}H NMR (300 MHz, $CDCl_3$) spectrum for compound 5.

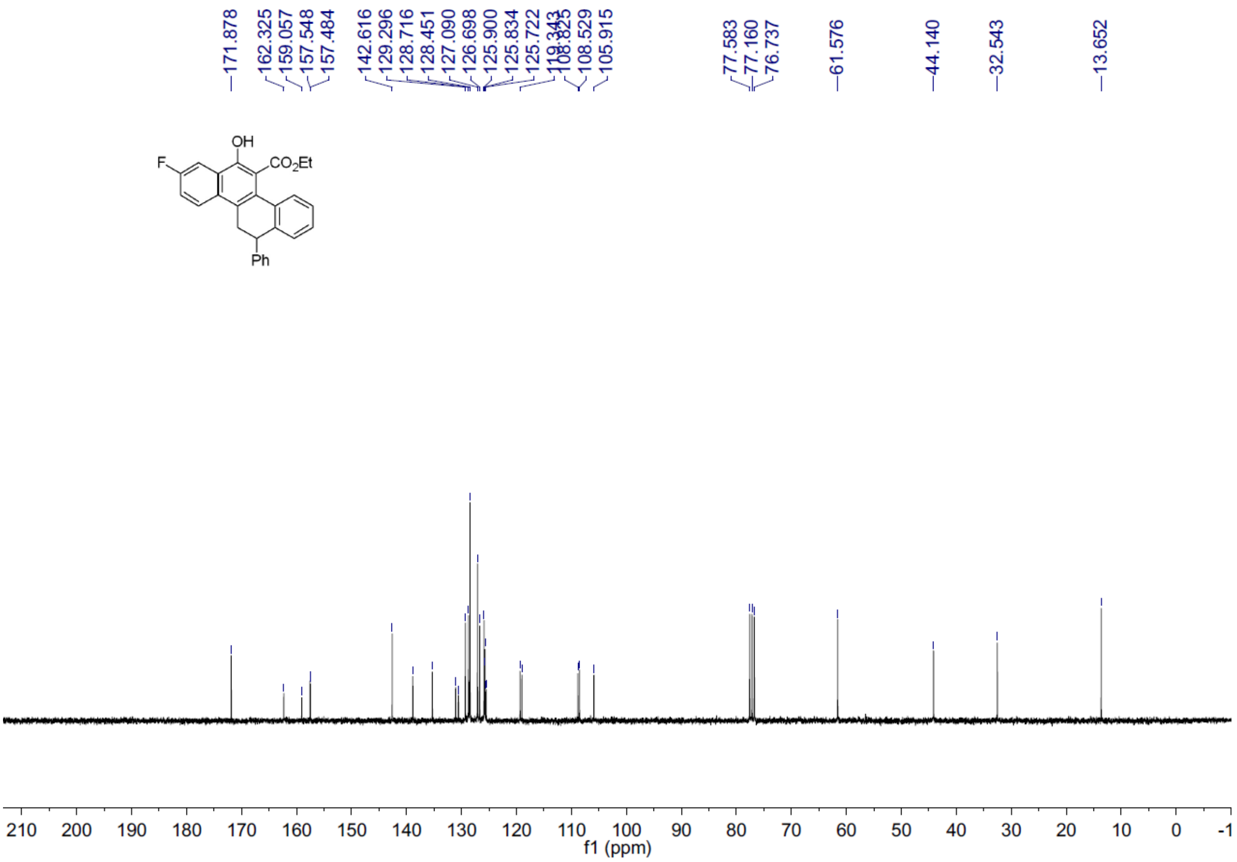

Supplementary Figure 16. ^{13}C NMR (75 MHz, $CDCl_3$) spectrum for compound 5.

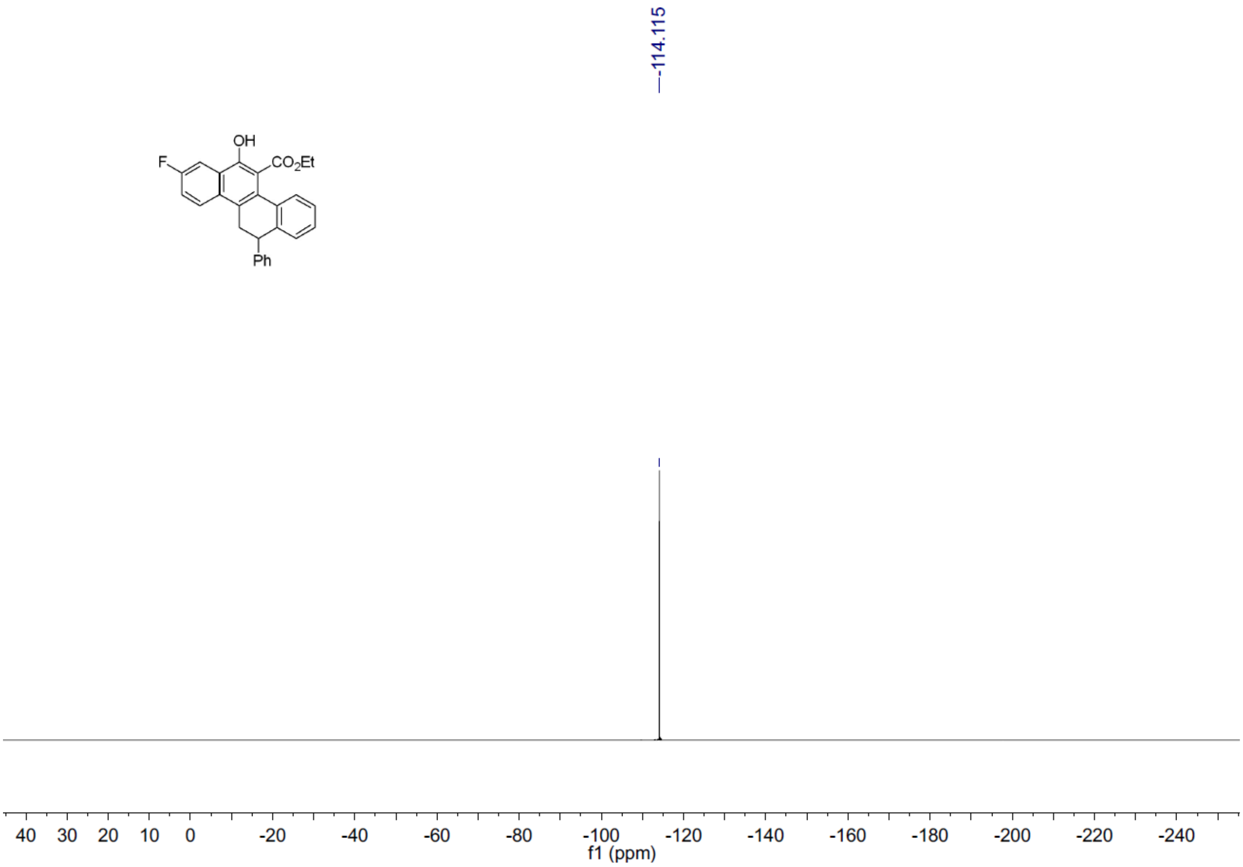

Supplementary Figure 17. ^{19}F NMR (283 MHz, $CDCl_3$) spectrum for compound 5.

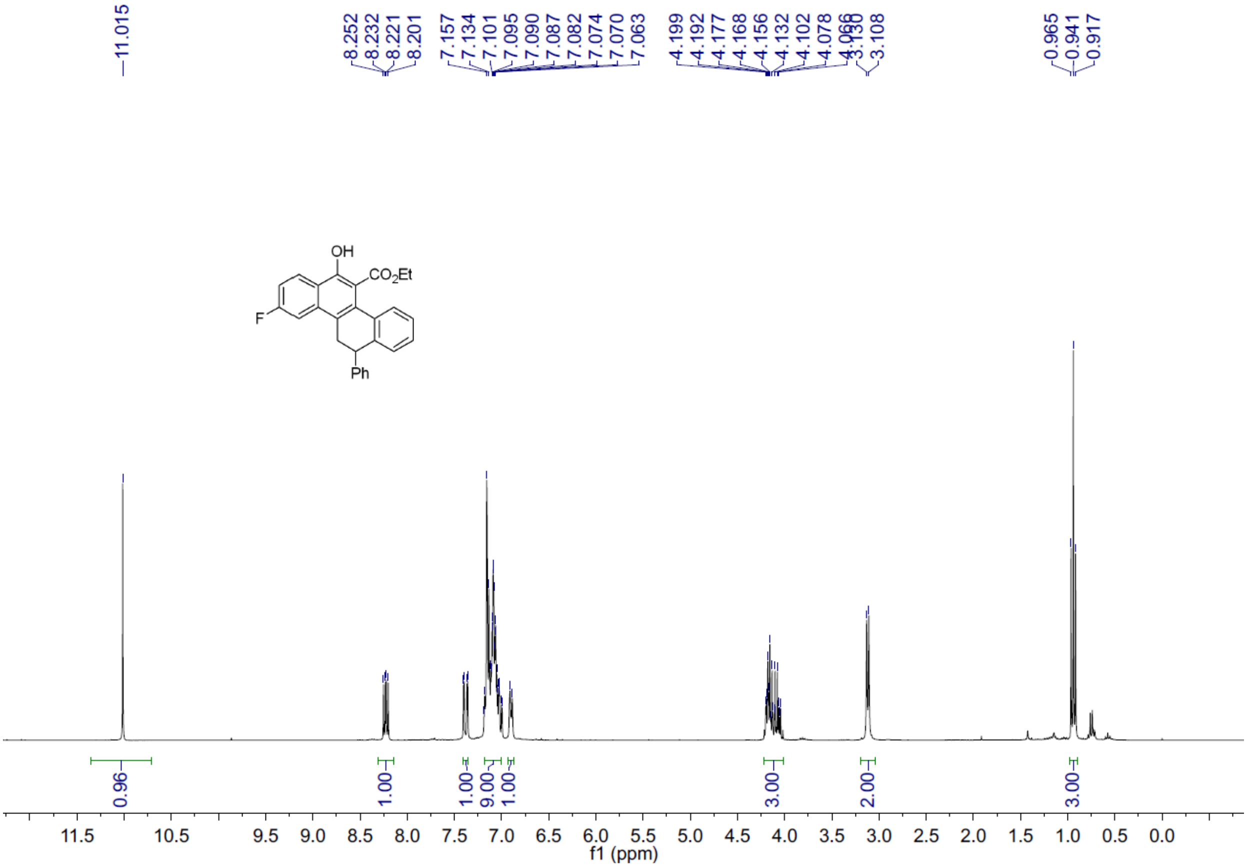

Supplementary Figure 18. ^{1}H NMR (300 MHz, $CDCl_3$) spectrum for compound 6.

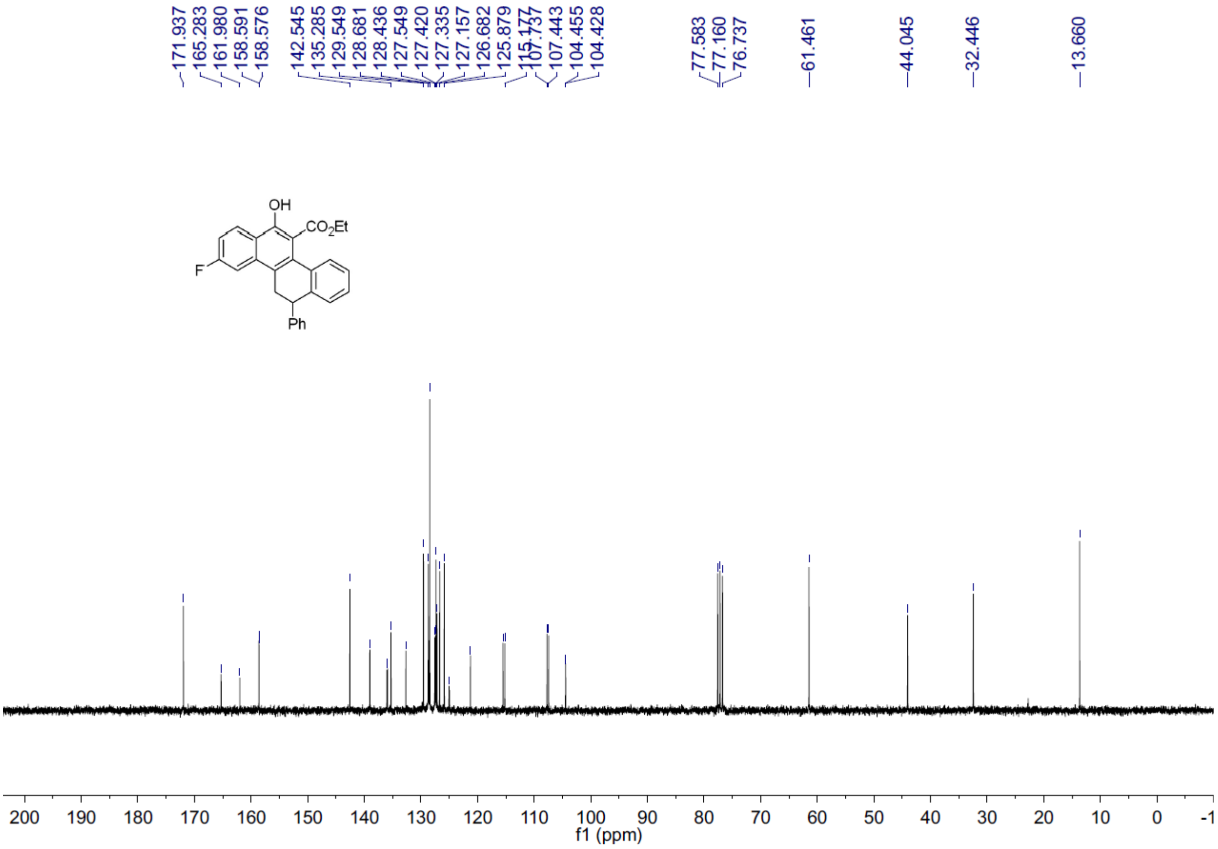

Supplementary Figure 19. ^{13}C **NMR (75 MHz,** $CDCl_3$**) spectrum for compound 6.**

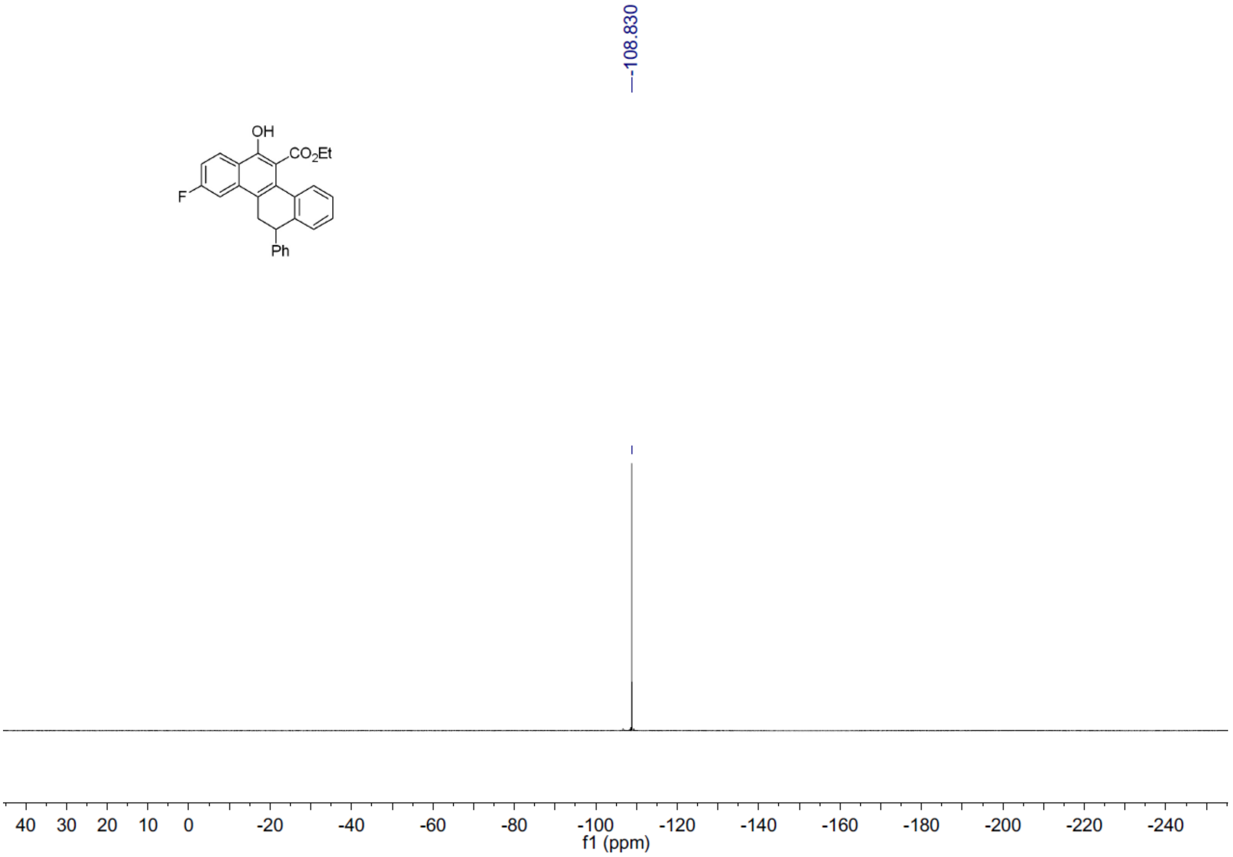

Supplementary Figure 20. ^{19}F **NMR (283 MHz,** $CDCl_3$**) spectrum for compound 6.**

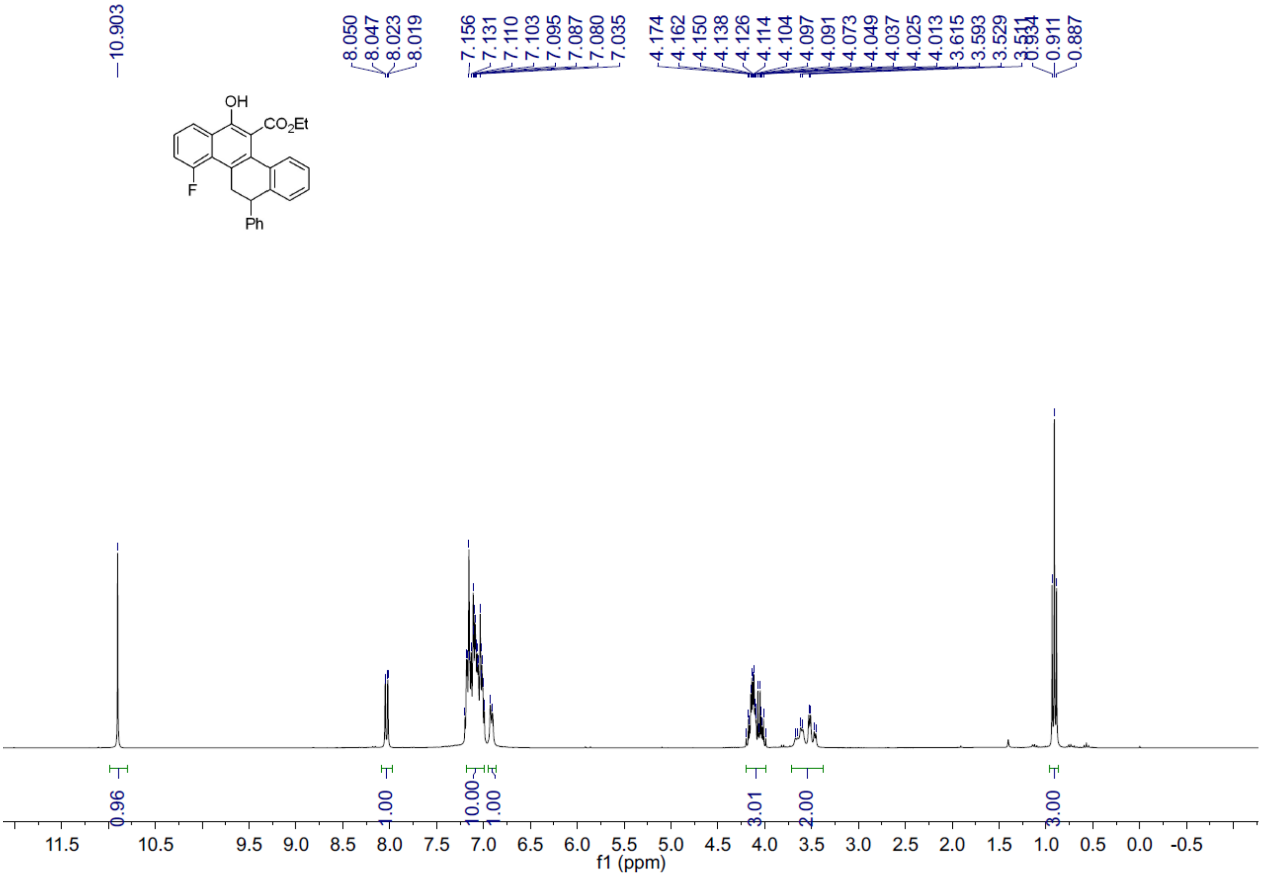

Supplementary Figure 21. ^{1}H NMR (400 MHz, $CDCl_3$) spectrum for compound 7.

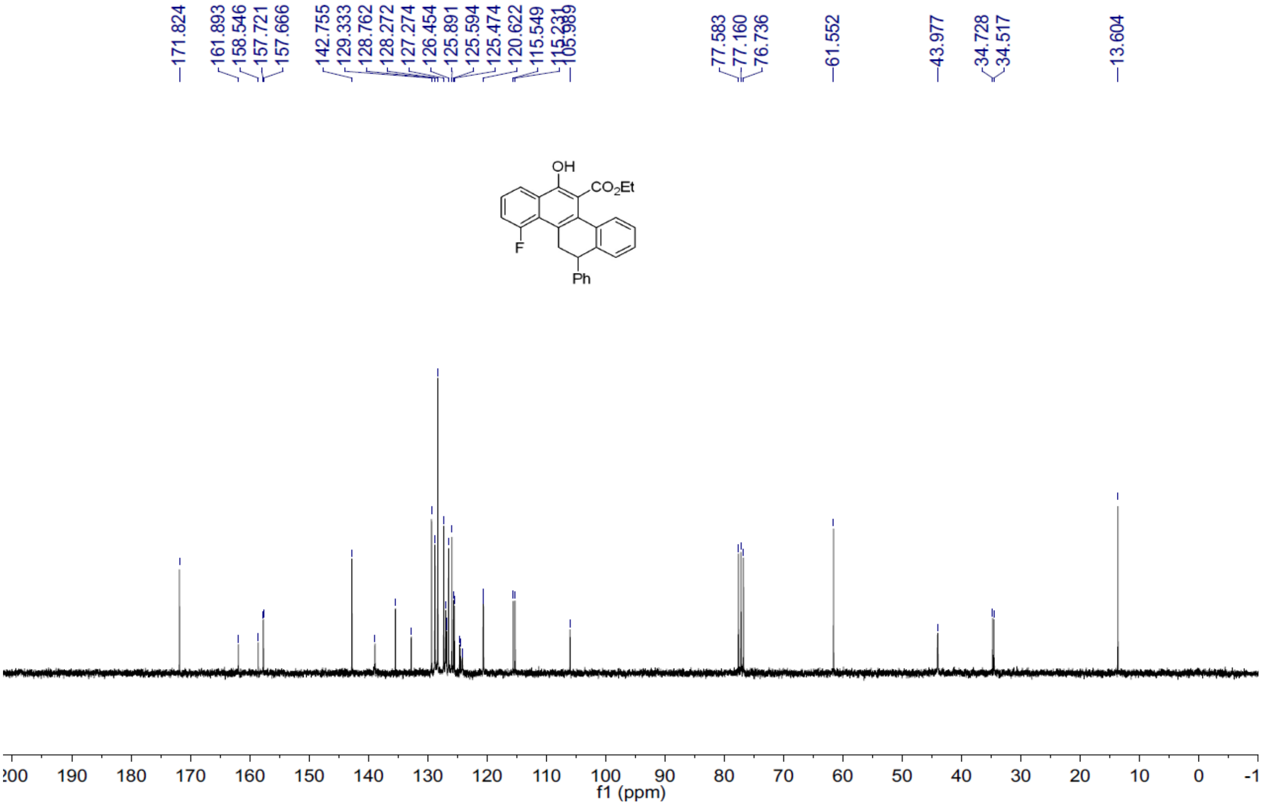

Supplementary Figure 22. ^{13}C NMR (75 MHz, $CDCl_3$) spectrum for compound 7.

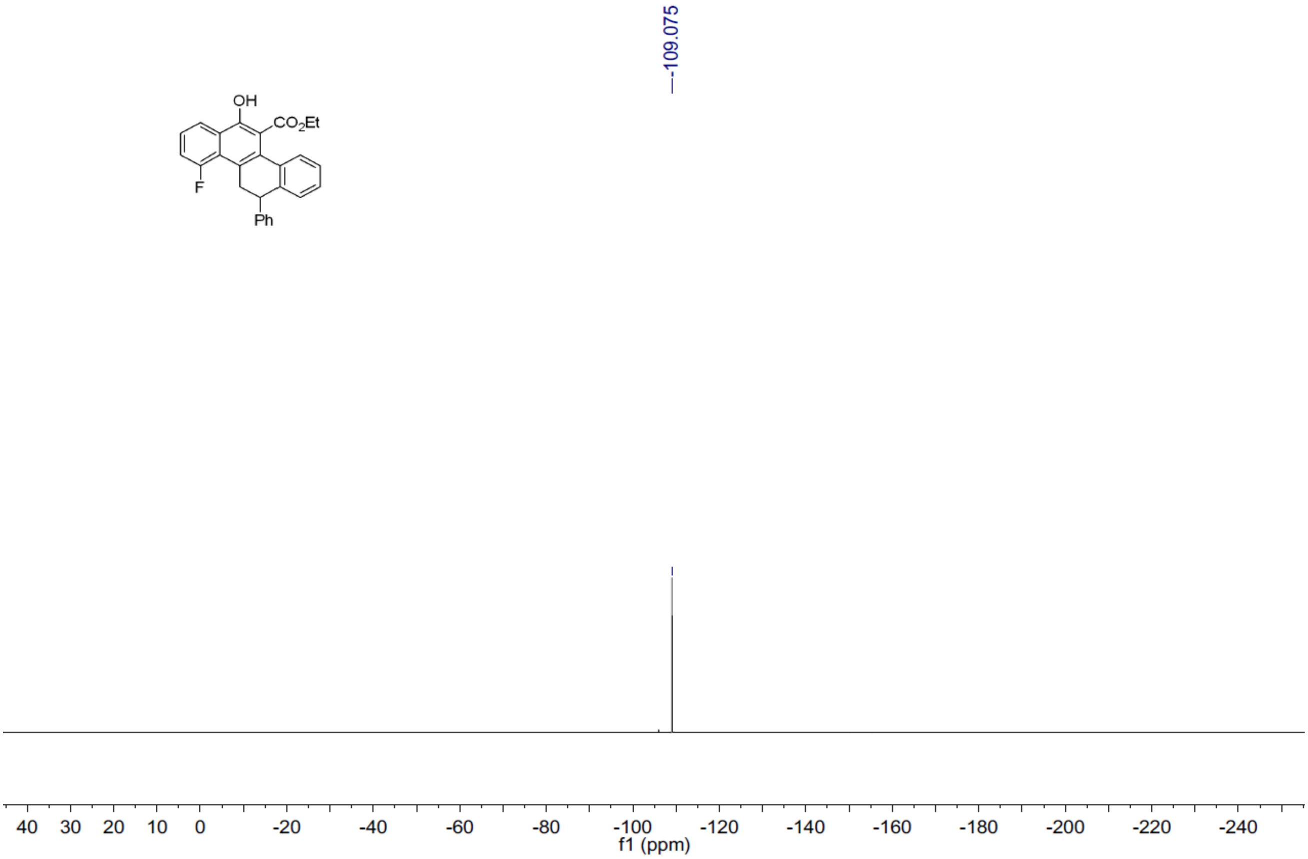

Supplementary Figure 23. ^{19}F NMR (283 MHz, $CDCl_3$) spectrum for compound 7.

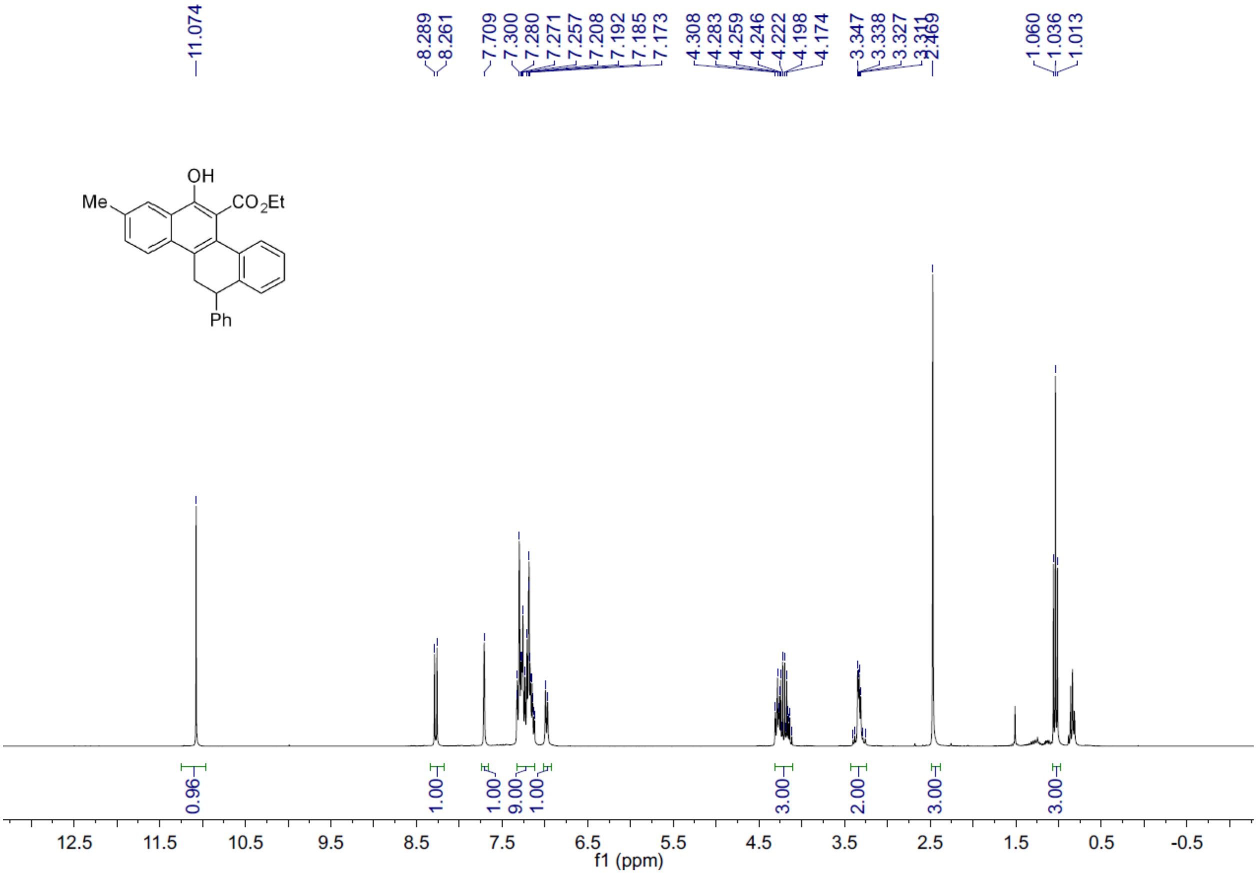

Supplementary Figure 24. ^{1}H NMR (300 MHz, $CDCl_3$) spectrum for compound 8.

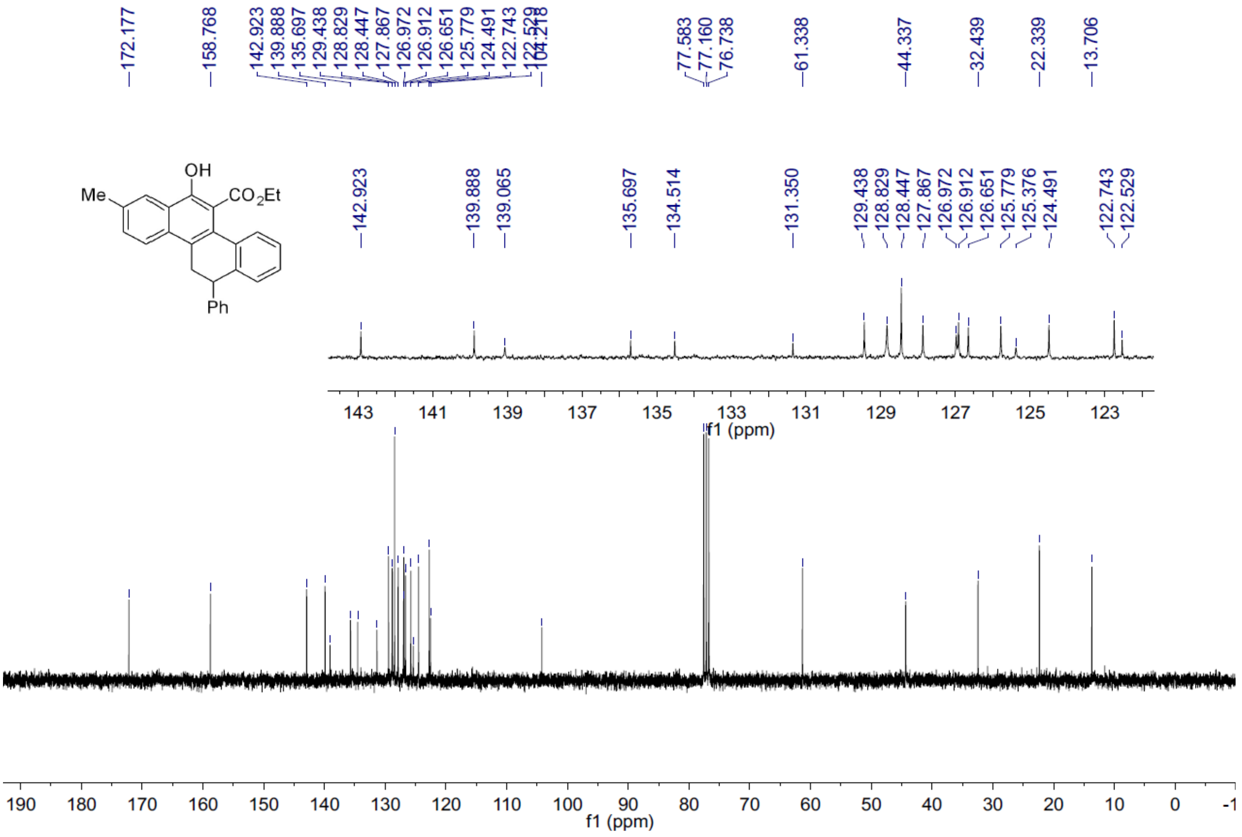

Supplementary Figure 25. **^{13}C NMR (75 MHz, $CDCl_3$) spectrum for compound 8.**

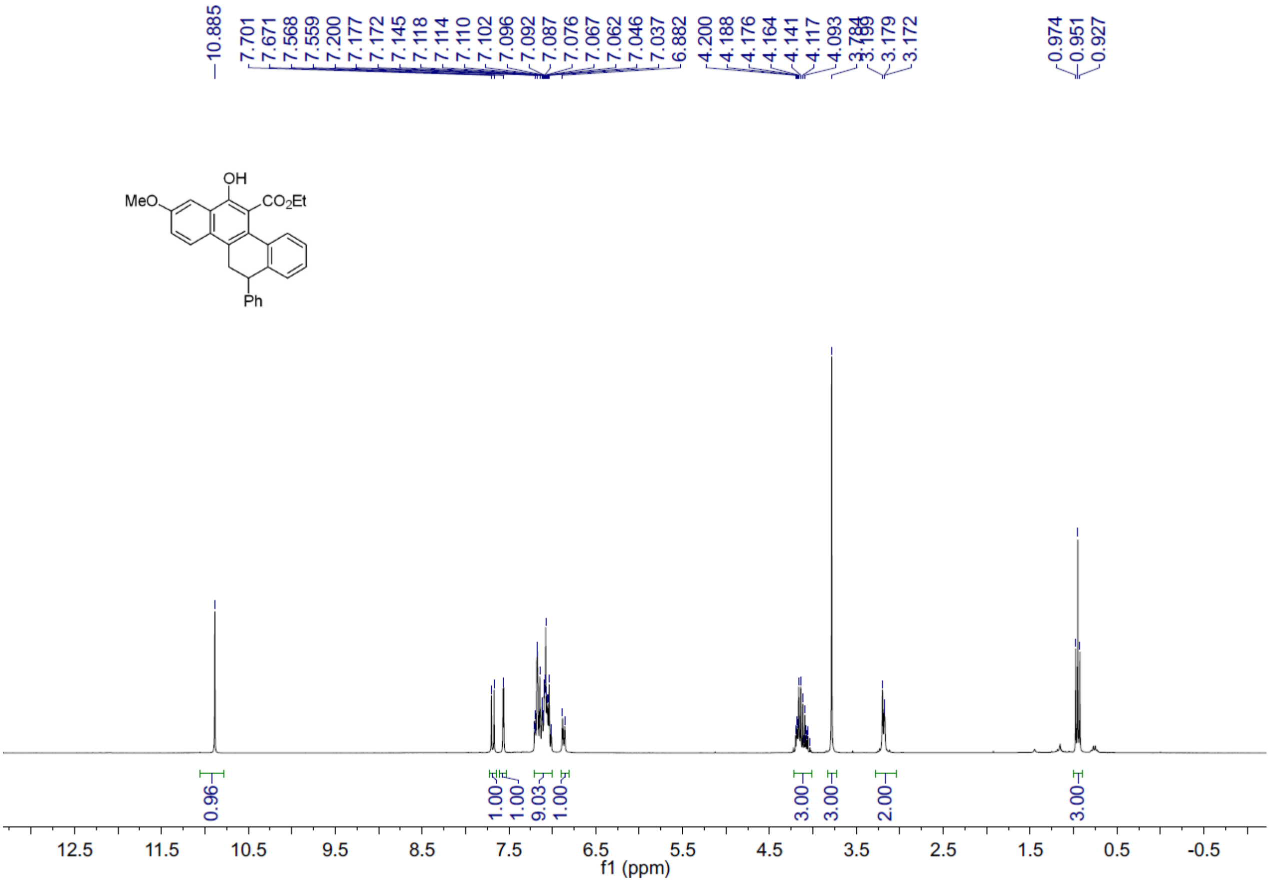

Supplementary Figure 26. **^{1}H NMR (300 MHz, $CDCl_3$) spectrum for compound 9.**

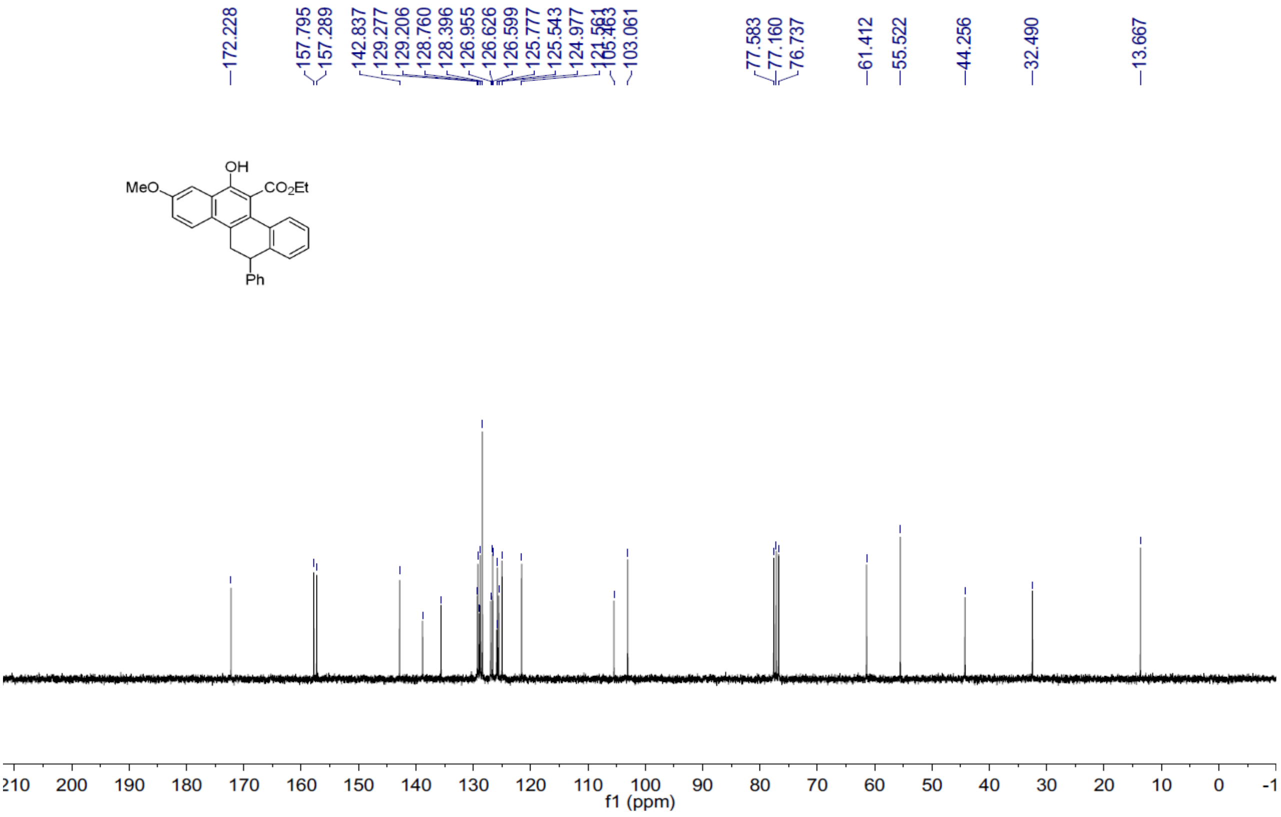

Supplementary Figure 27. ^{13}C NMR (75 MHz, $CDCl_3$) spectrum for compound 9.

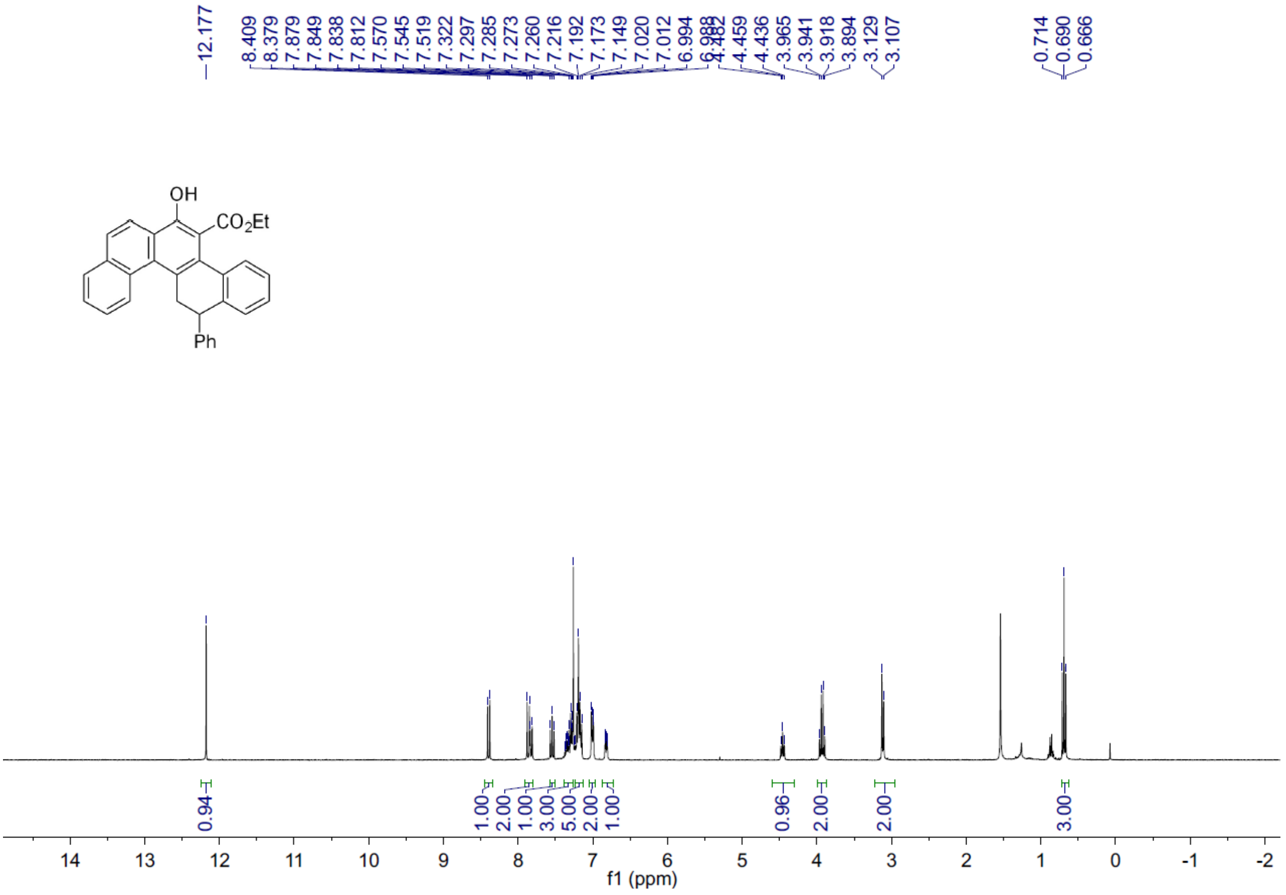

Supplementary Figure 28. ^{1}H NMR (400 MHz, $CDCl_3$) spectrum for compound 10.

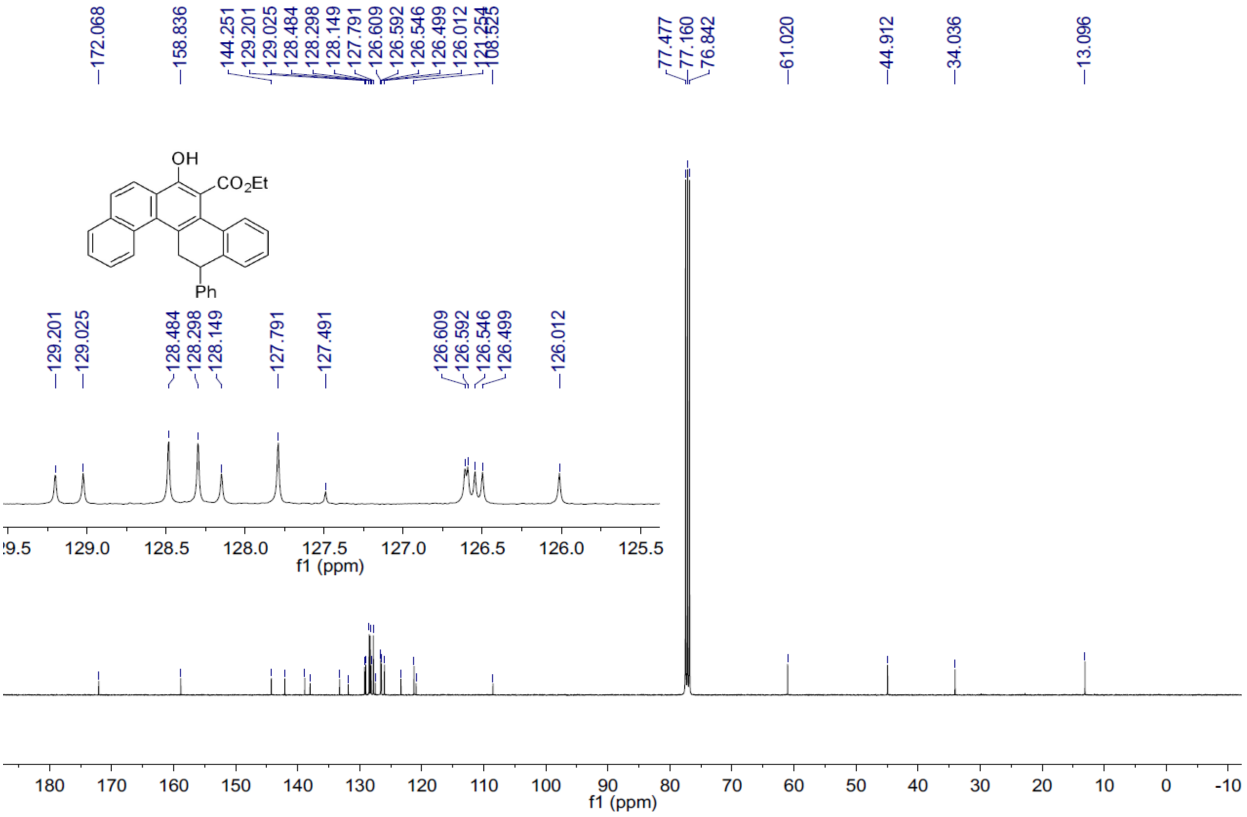

Supplementary Figure 29. ^{13}C NMR (125 MHz, $CDCl_3$) spectrum for compound 10.

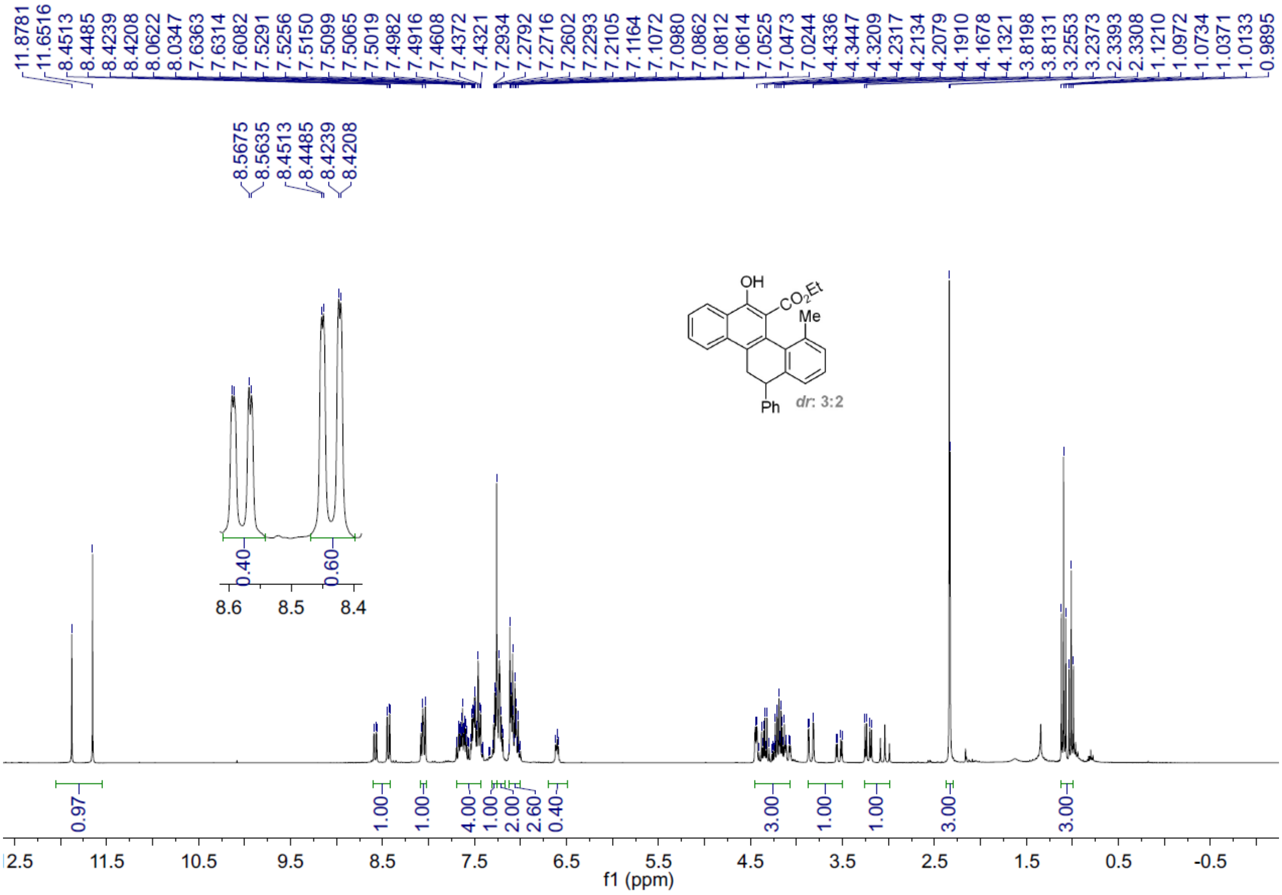

Supplementary Figure 30. ^{1}H NMR (300 MHz, $CDCl_3$) spectrum for compound 11.

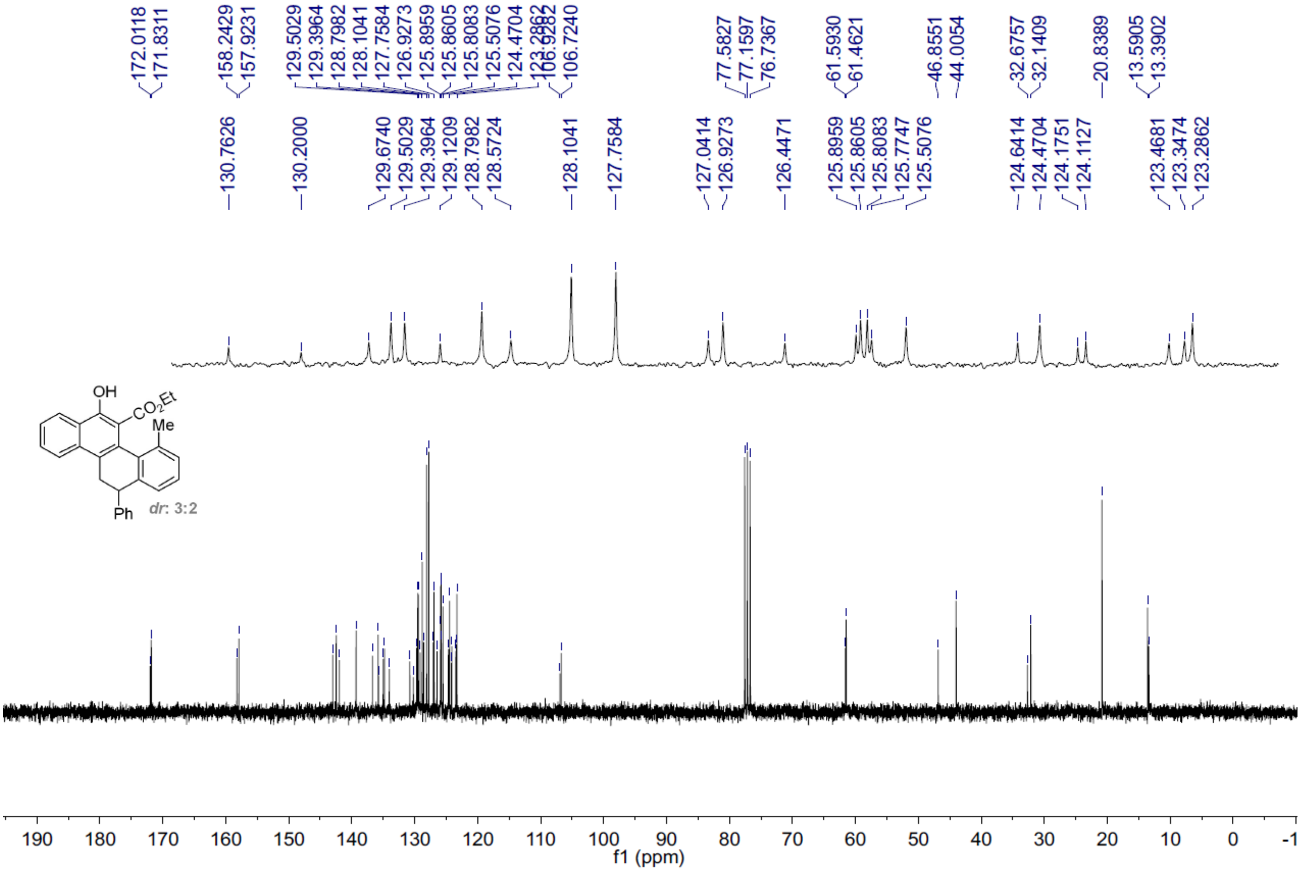

Supplementary Figure 31. ^{13}C NMR (75 MHz, $CDCl_3$) spectrum for compound 11.

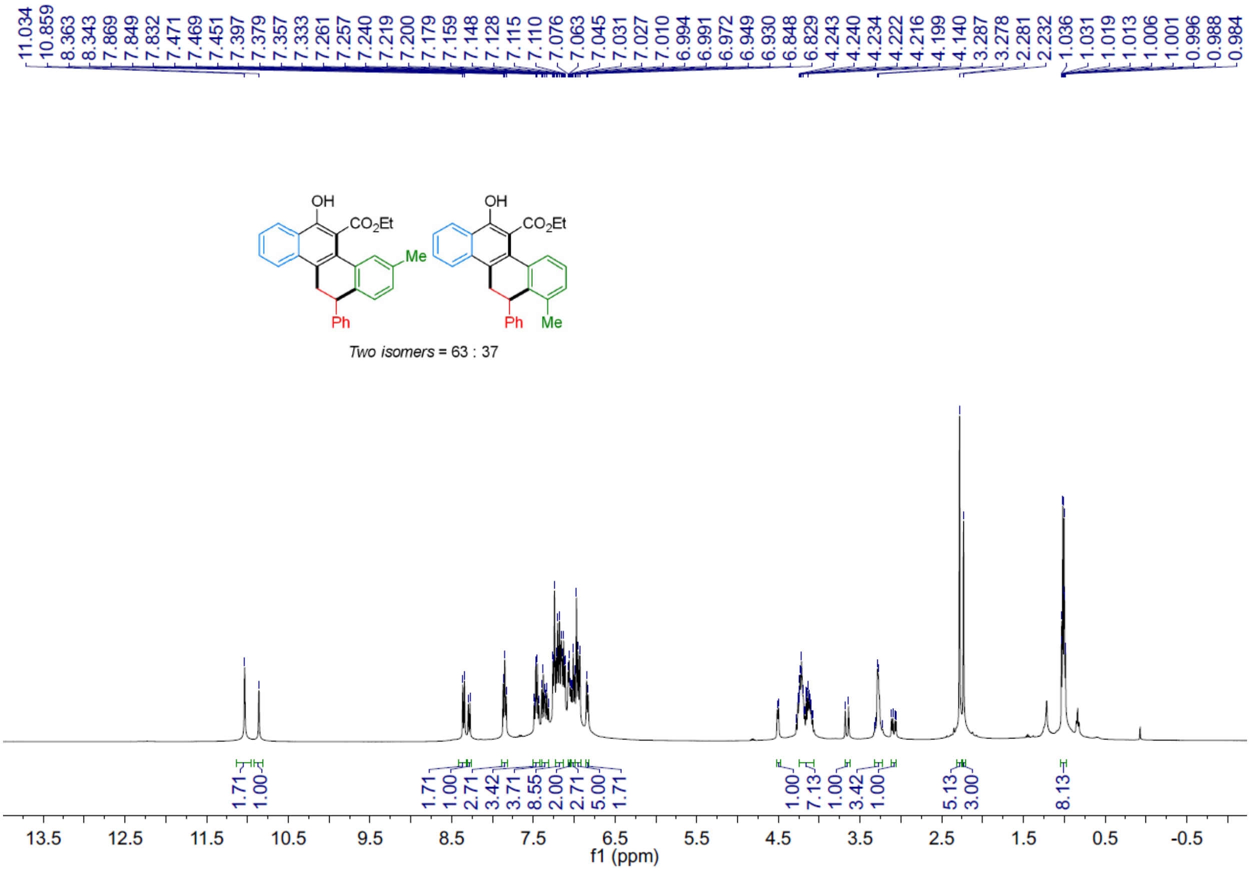

Supplementary Figure 32. ^{1}H NMR (400 MHz, $CDCl_3$) spectrum for compound 12.

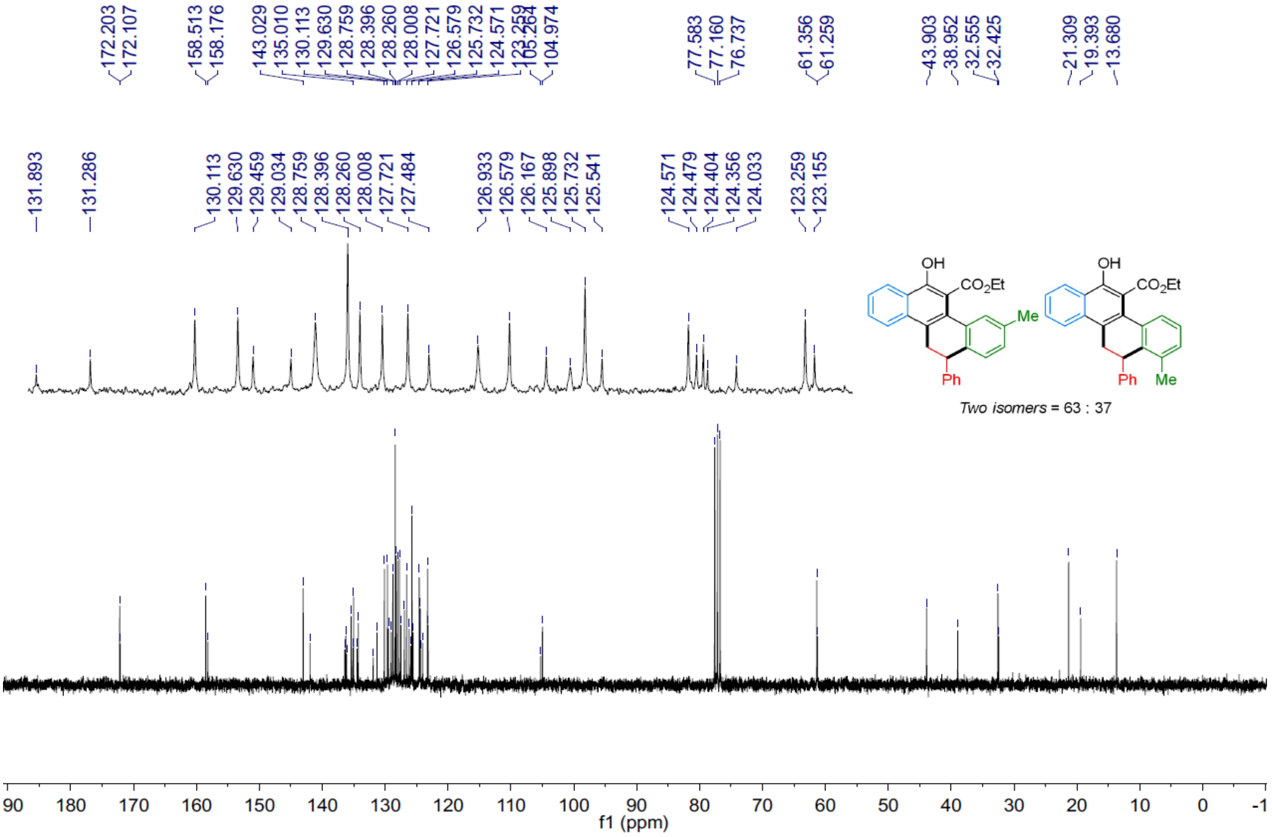

Supplementary Figure 33. ^{13}C NMR (100 MHz, $CDCl_3$) spectrum for compound 12.

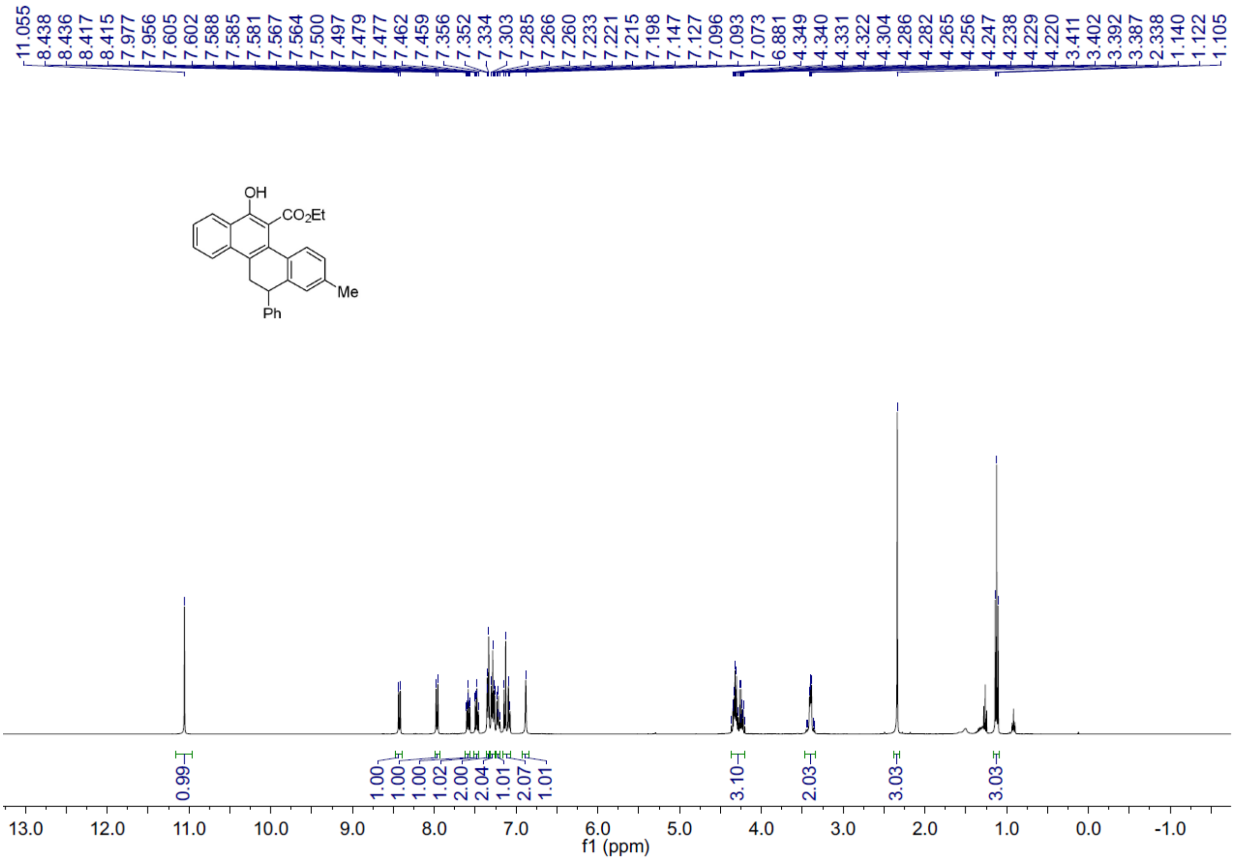

Supplementary Figure 34. ^{1}H NMR (400 MHz, $CDCl_3$) spectrum for compound 13.

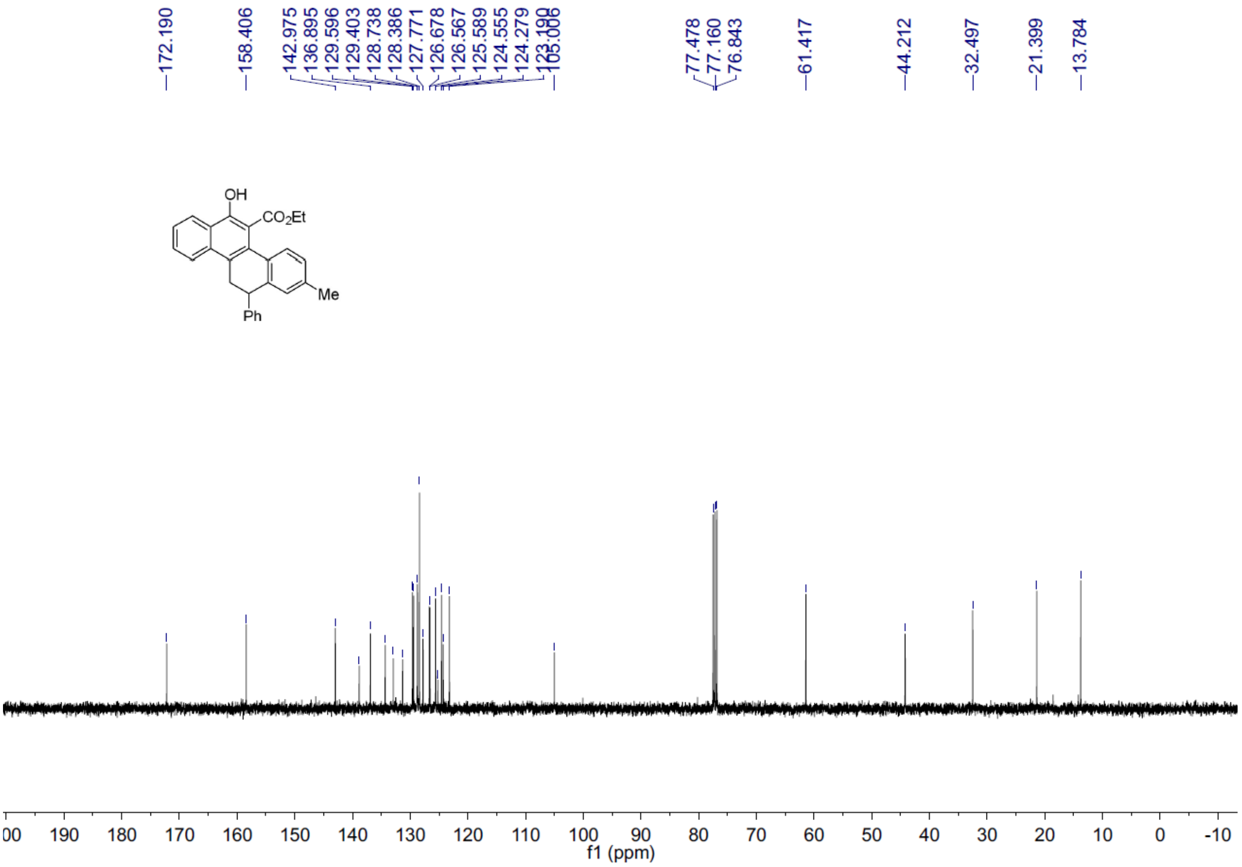

Supplementary Figure 35. ^{13}C NMR (100 MHz, CDCl$_3$) spectrum for compound 13.

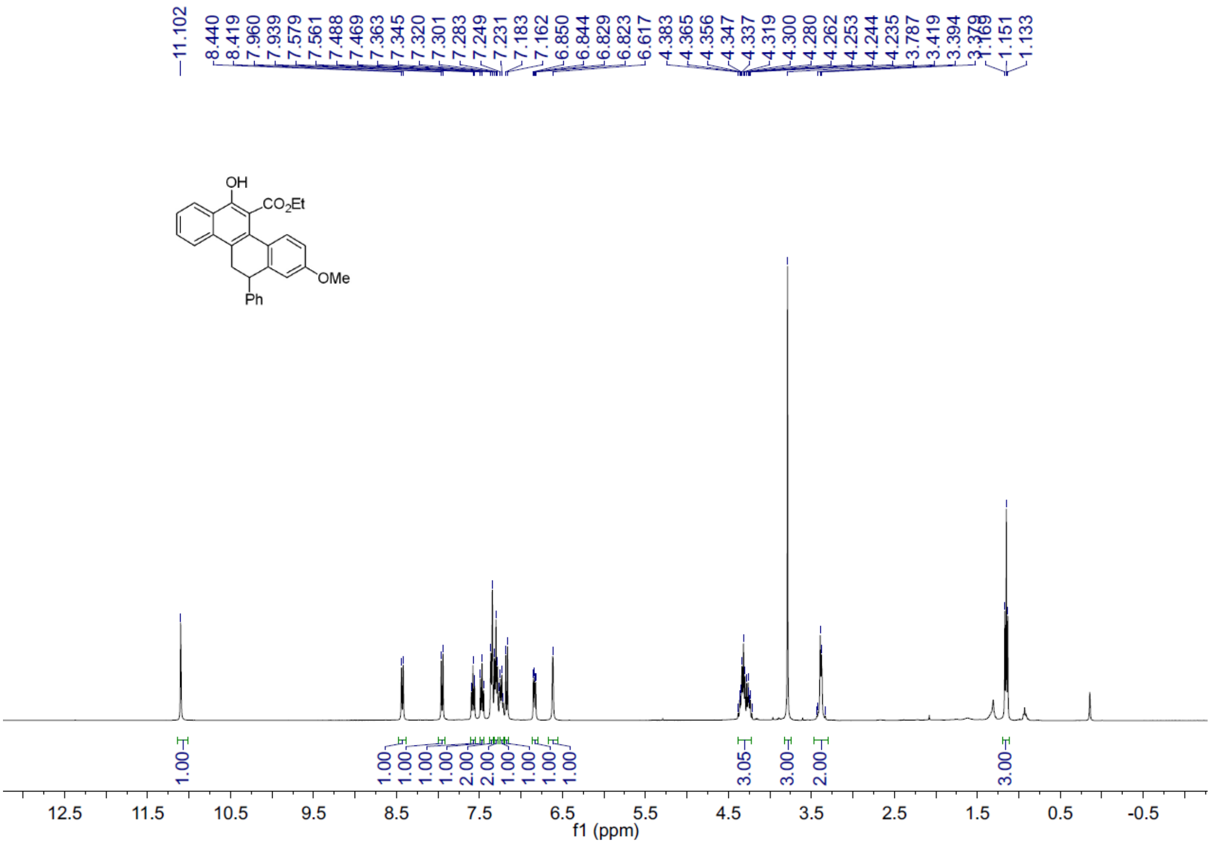

Supplementary Figure 36. ^{1}H NMR (400 MHz, CDCl$_3$) spectrum for compound 14.

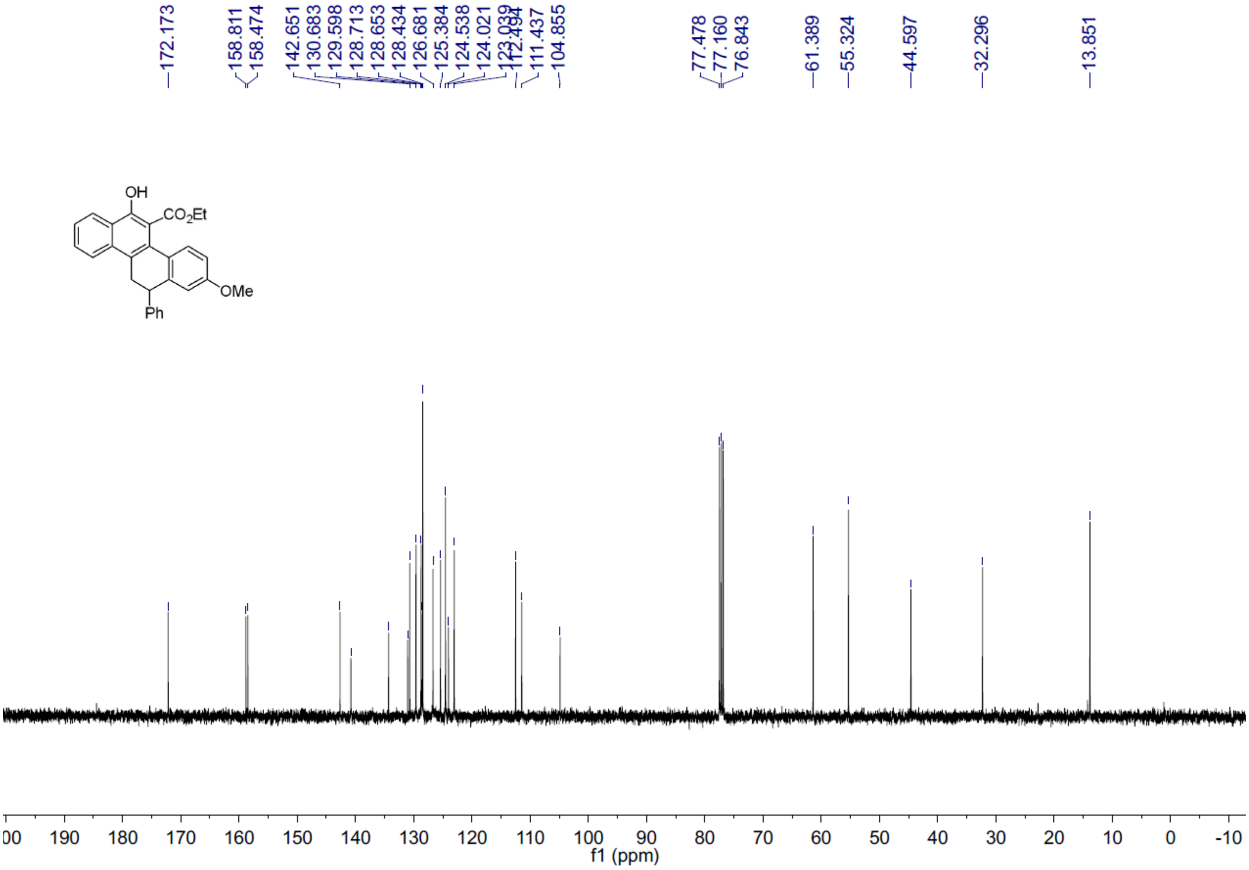

Supplementary Figure 37. ^{13}C NMR (100 MHz, $CDCl_3$) spectrum for compound 14.

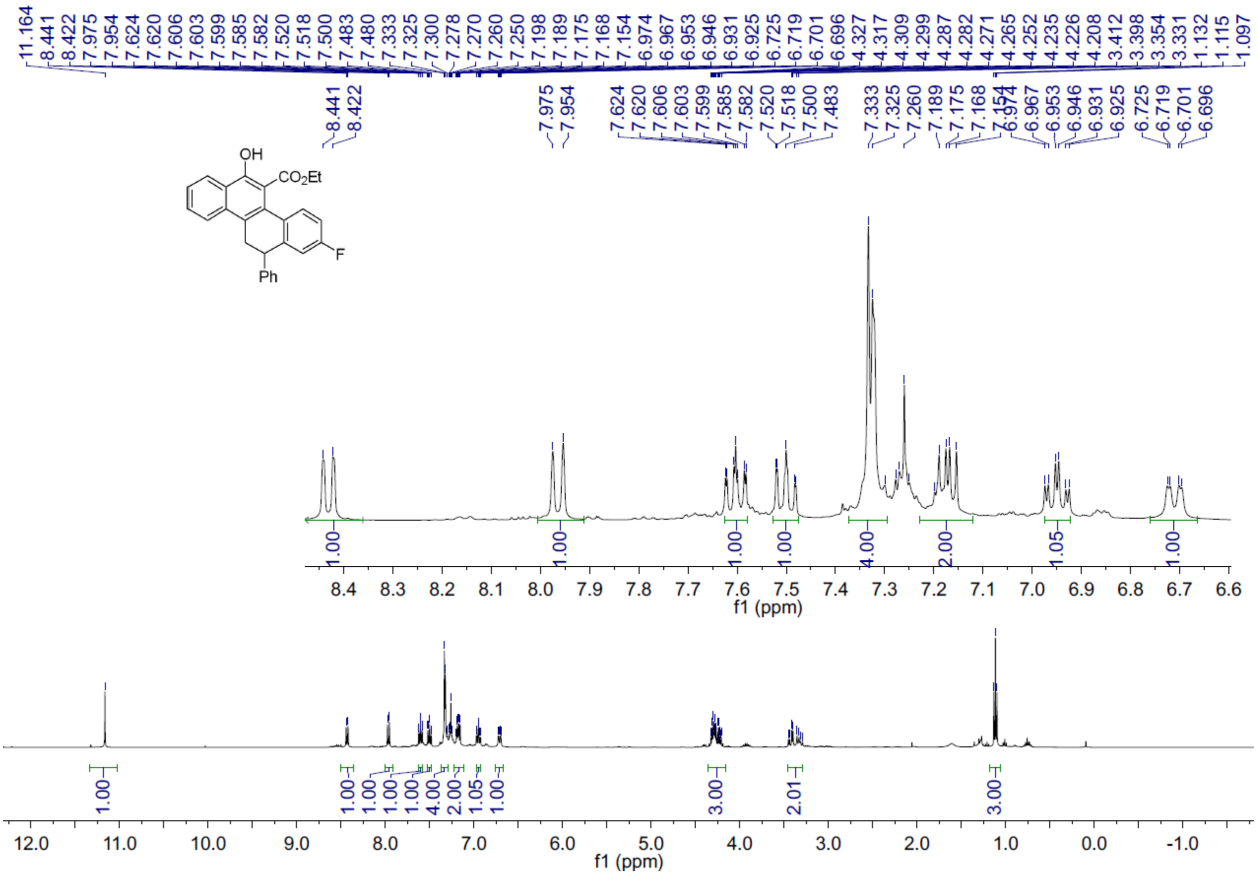

Supplementary Figure 38. ^{1}H NMR (400 MHz, $CDCl_3$) spectrum for compound 15.

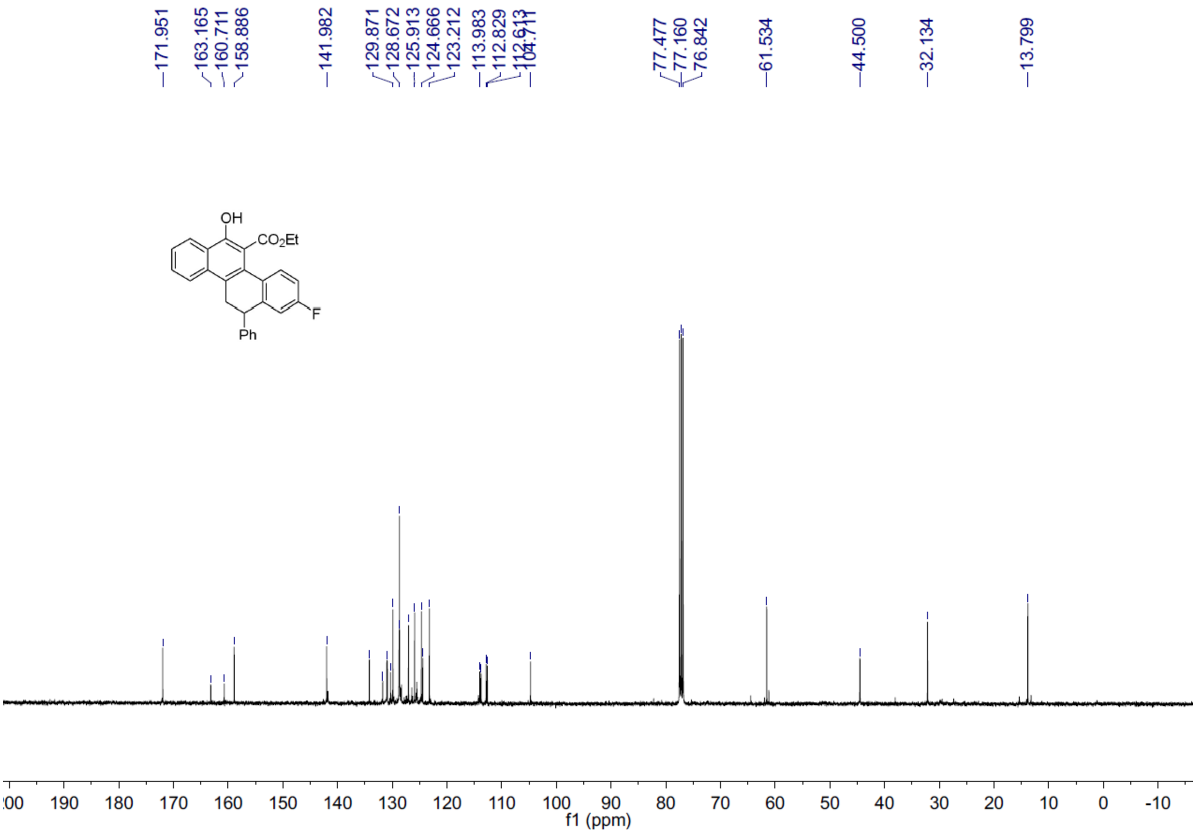

Supplementary Figure 39. ^{13}**C NMR (100 MHz,** $CDCl_3$**) spectrum for compound 15.**

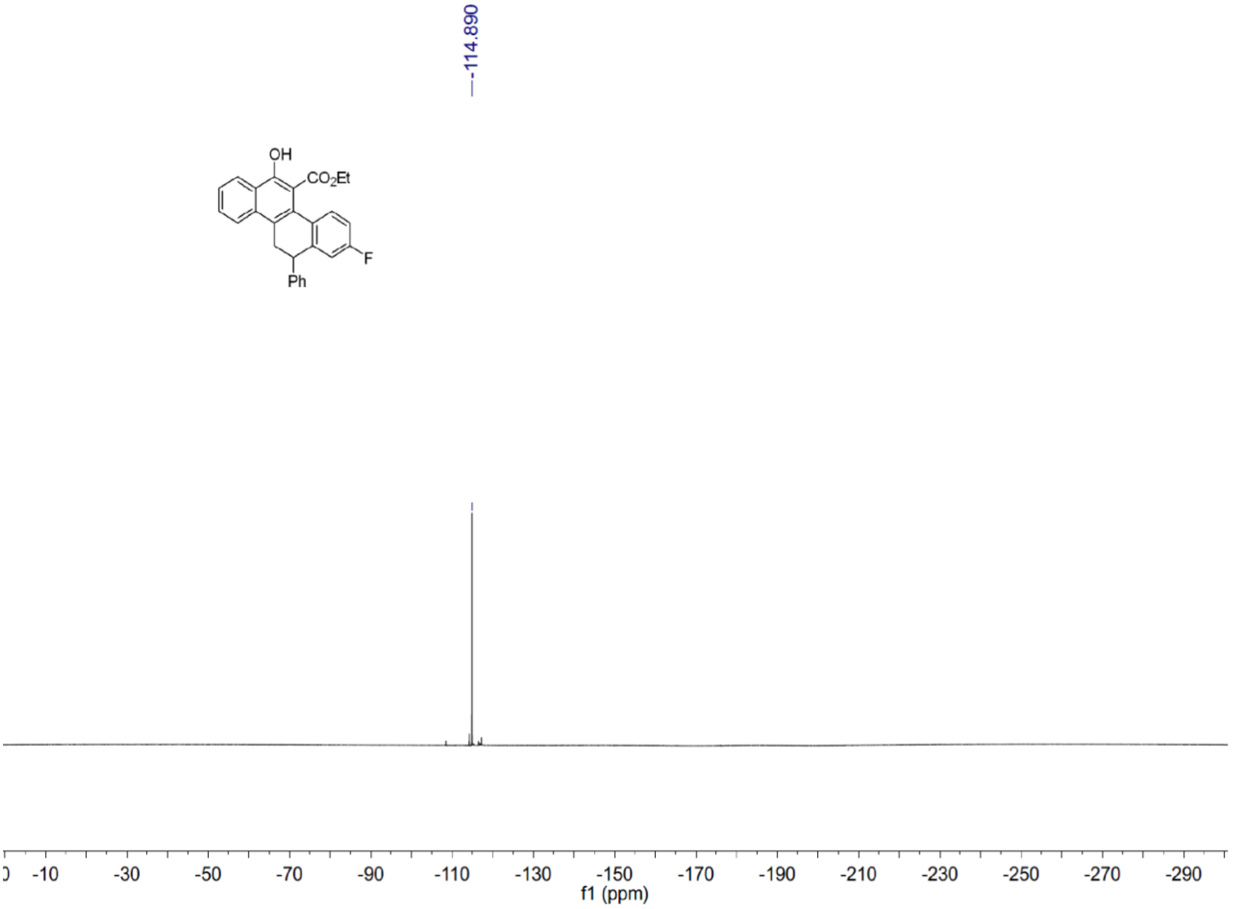

Supplementary Figure 40. ^{19}**F NMR (376 MHz,** $CDCl_3$**) spectrum for compound 15.**

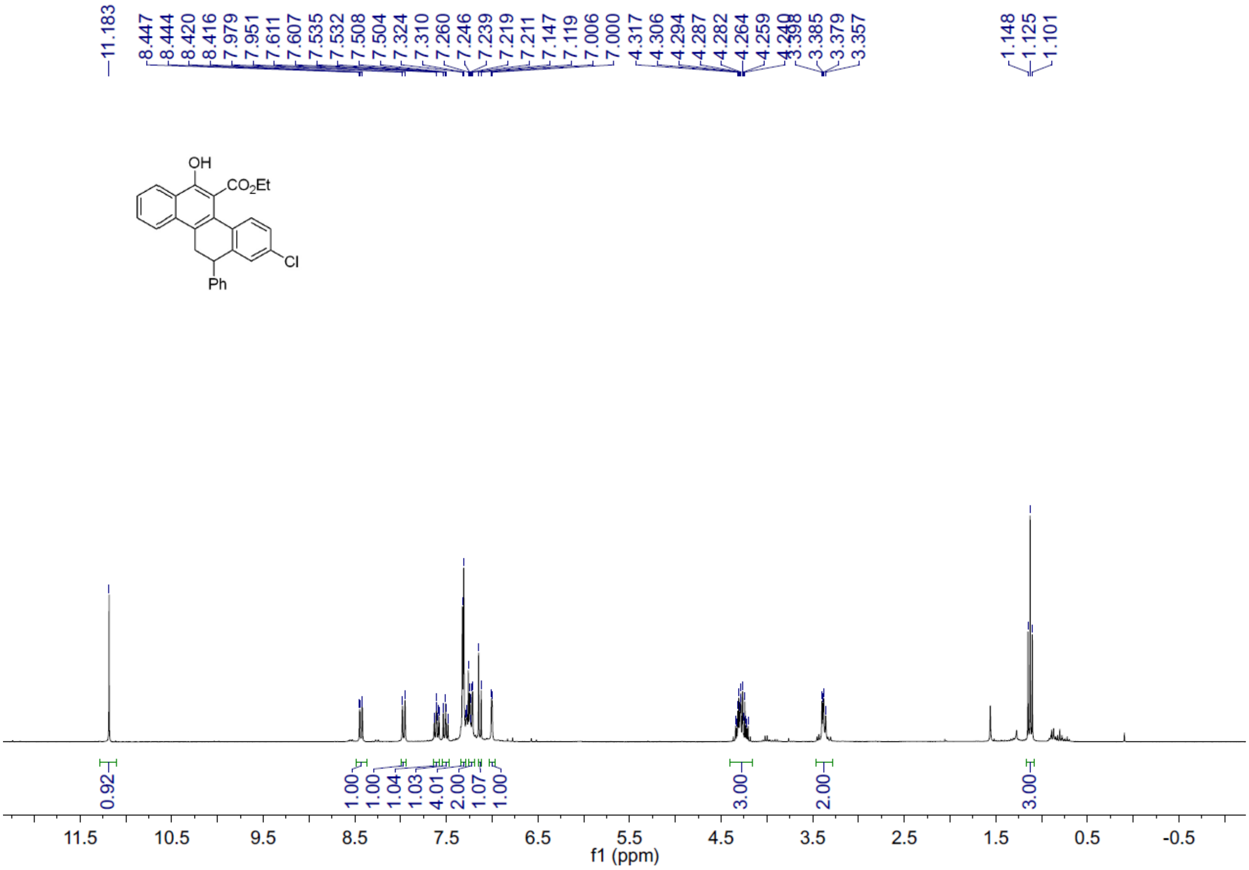

Supplementary Figure 41. ^{1}H NMR (300 MHz, CDCl$_3$) spectrum for compound 16.

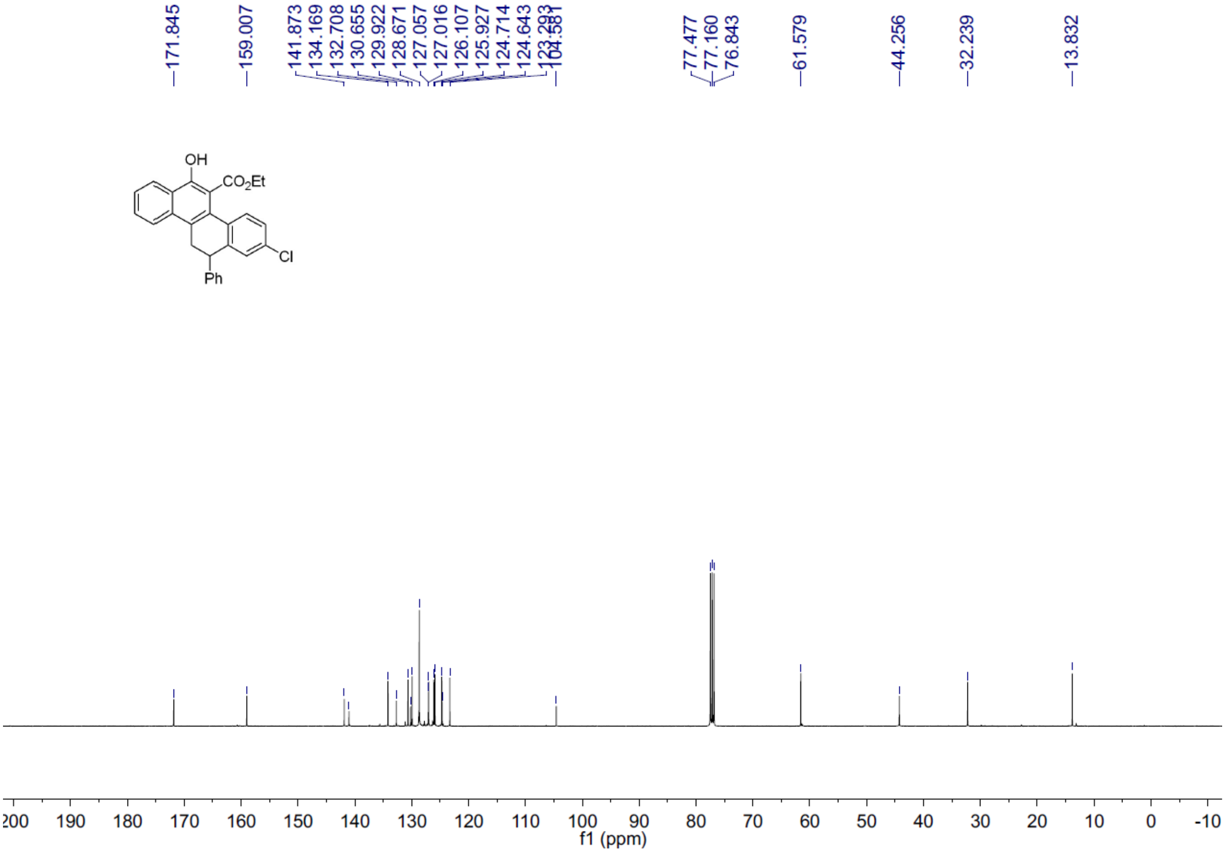

Supplementary Figure 42. ^{13}C NMR (100 MHz, CDCl$_3$) spectrum for compound 16.

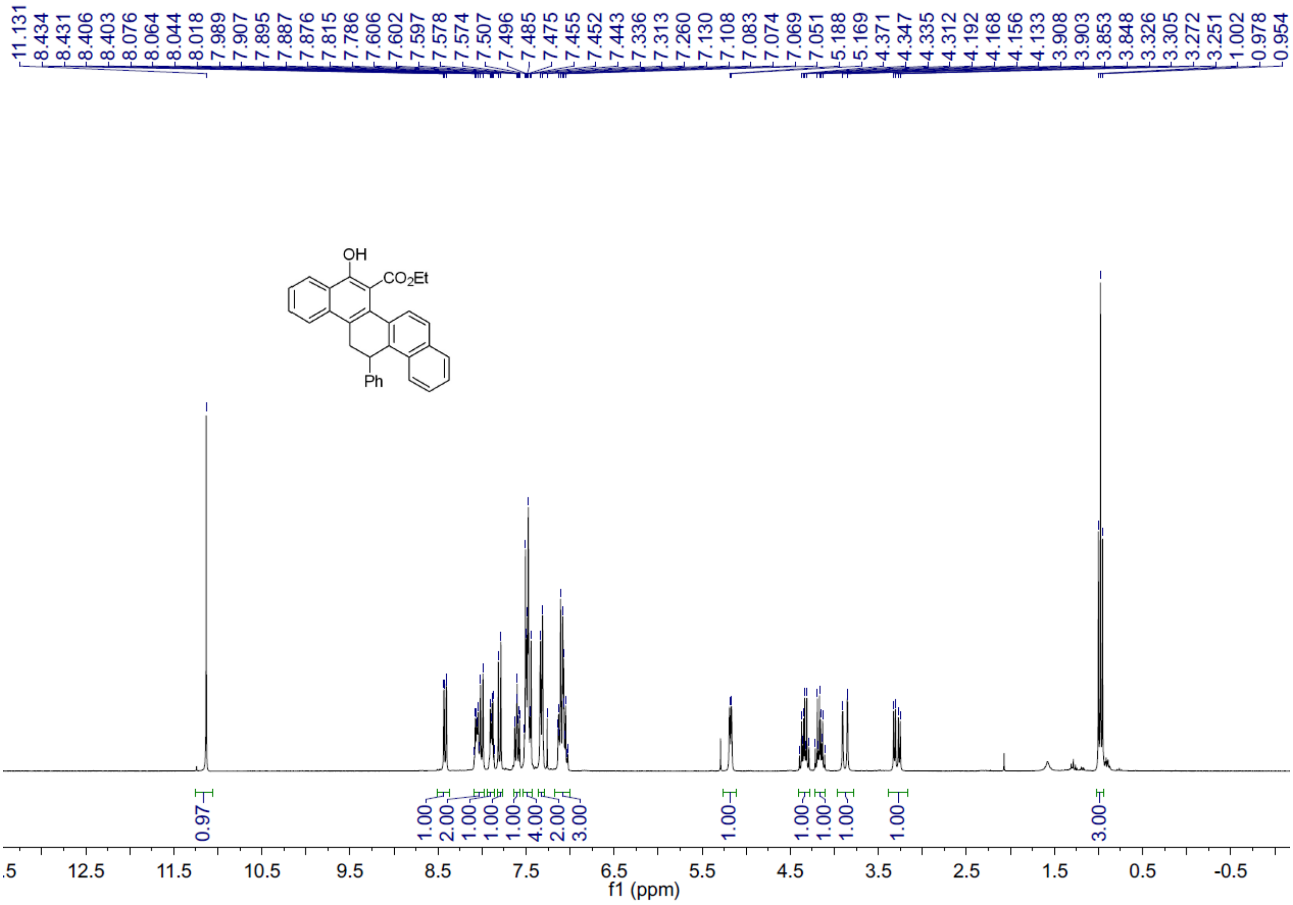

Supplementary Figure 43. ^{1}H NMR (400 MHz, $CDCl_3$) spectrum for compound 17.

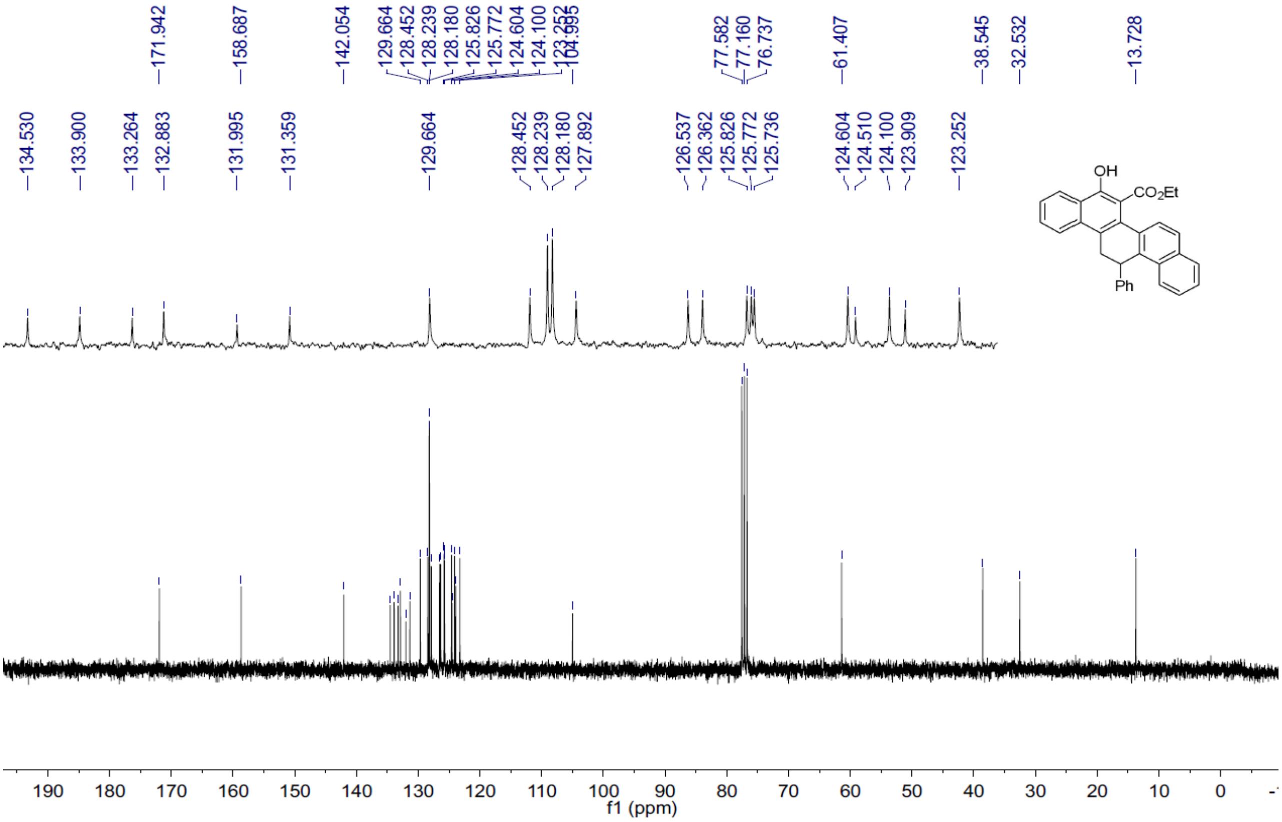

Supplementary Figure 44. ^{13}C NMR (100 MHz, $CDCl_3$) spectrum for compound 17.

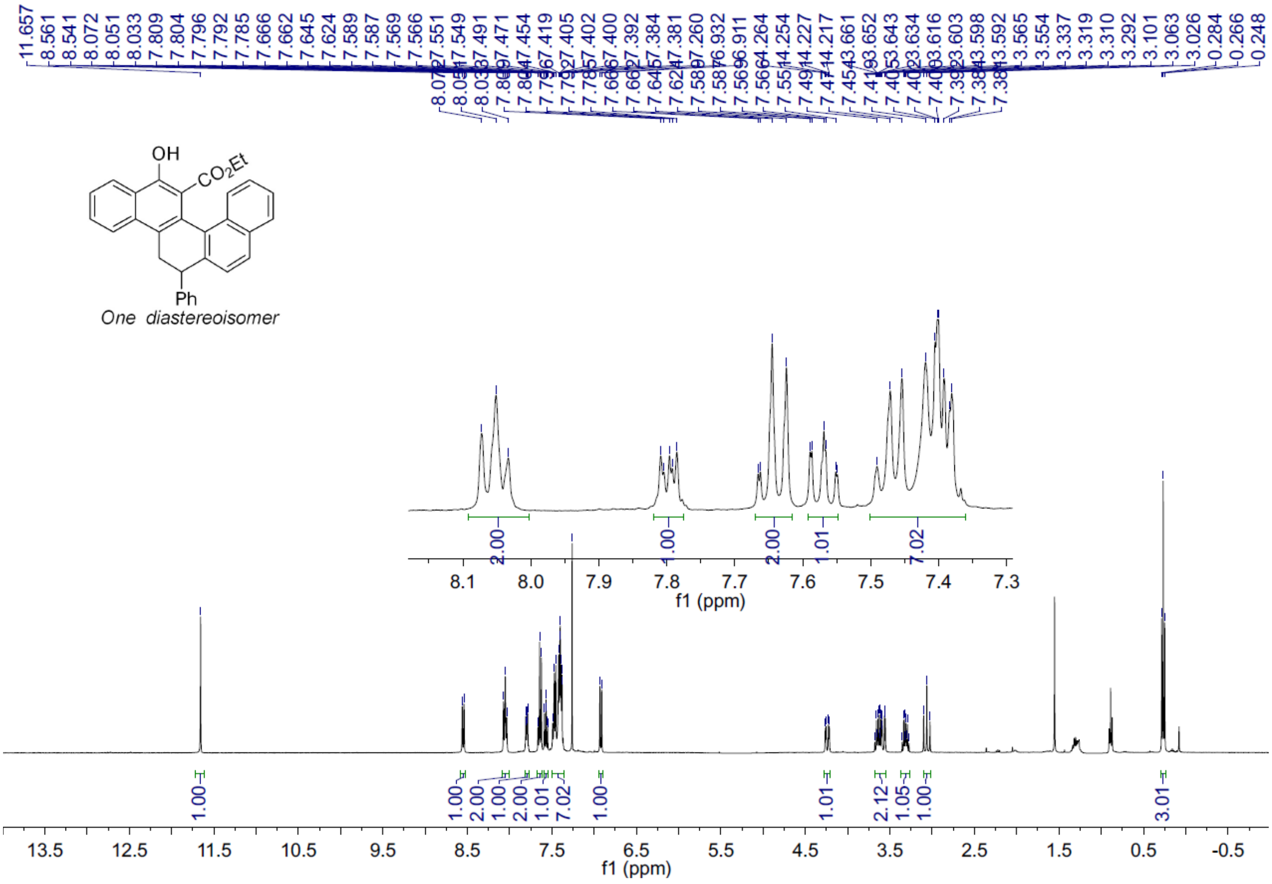

Supplementary Figure 45. ^{1}**H NMR (400 MHz,** $CDCl_3$**) spectrum for compound 18.**

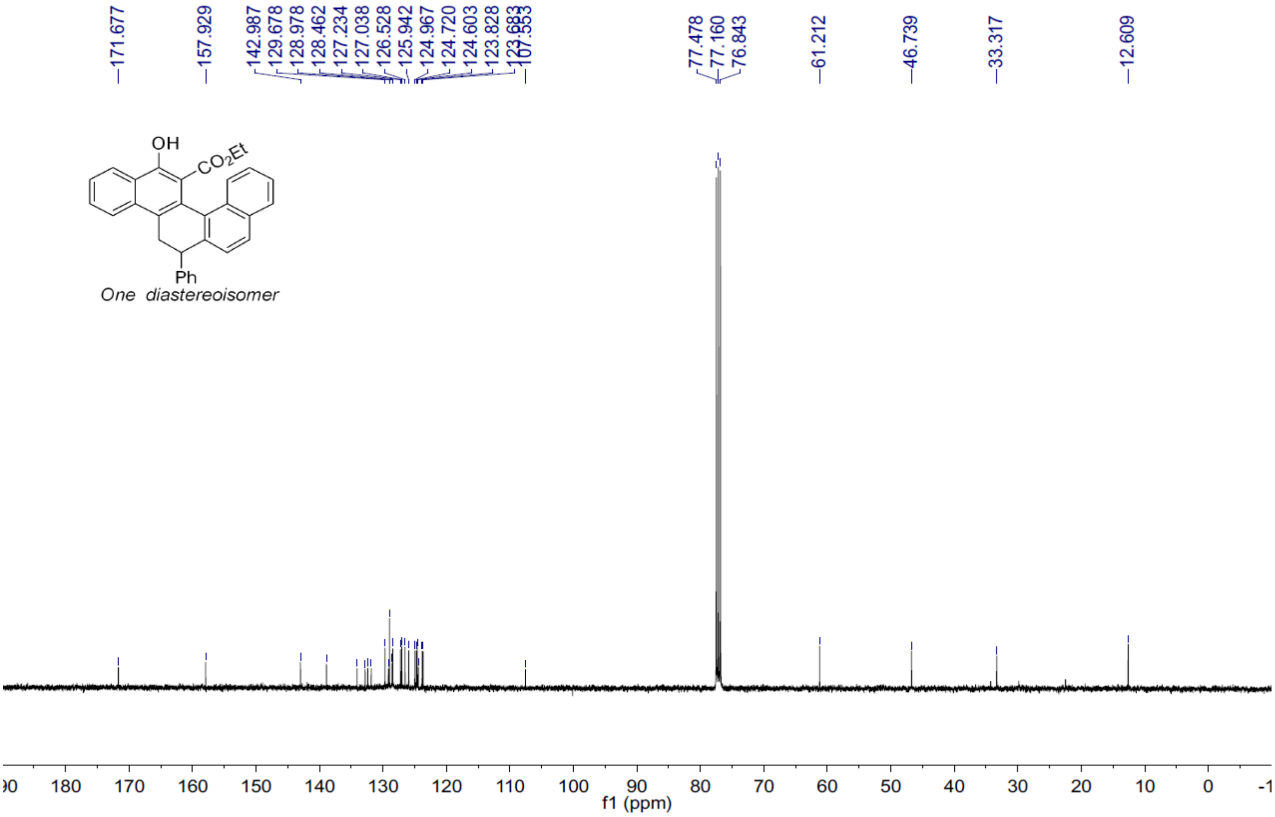

Supplementary Figure 46. ^{13}**C NMR (100 MHz,** $CDCl_3$**) spectrum for compound 18.**

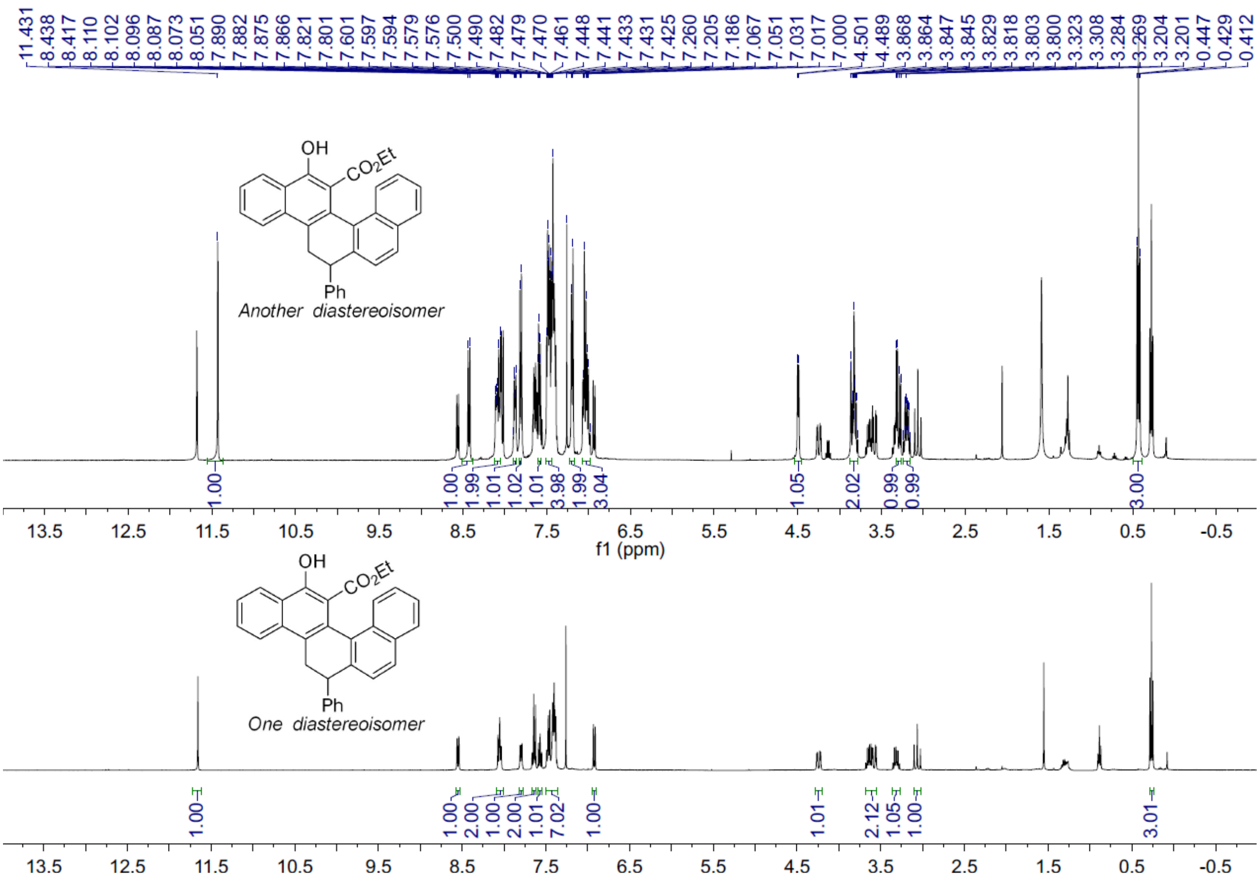

Supplementary Figure 47. ^{1}H NMR (400 MHz, $CDCl_3$) spectrum for compound 18.

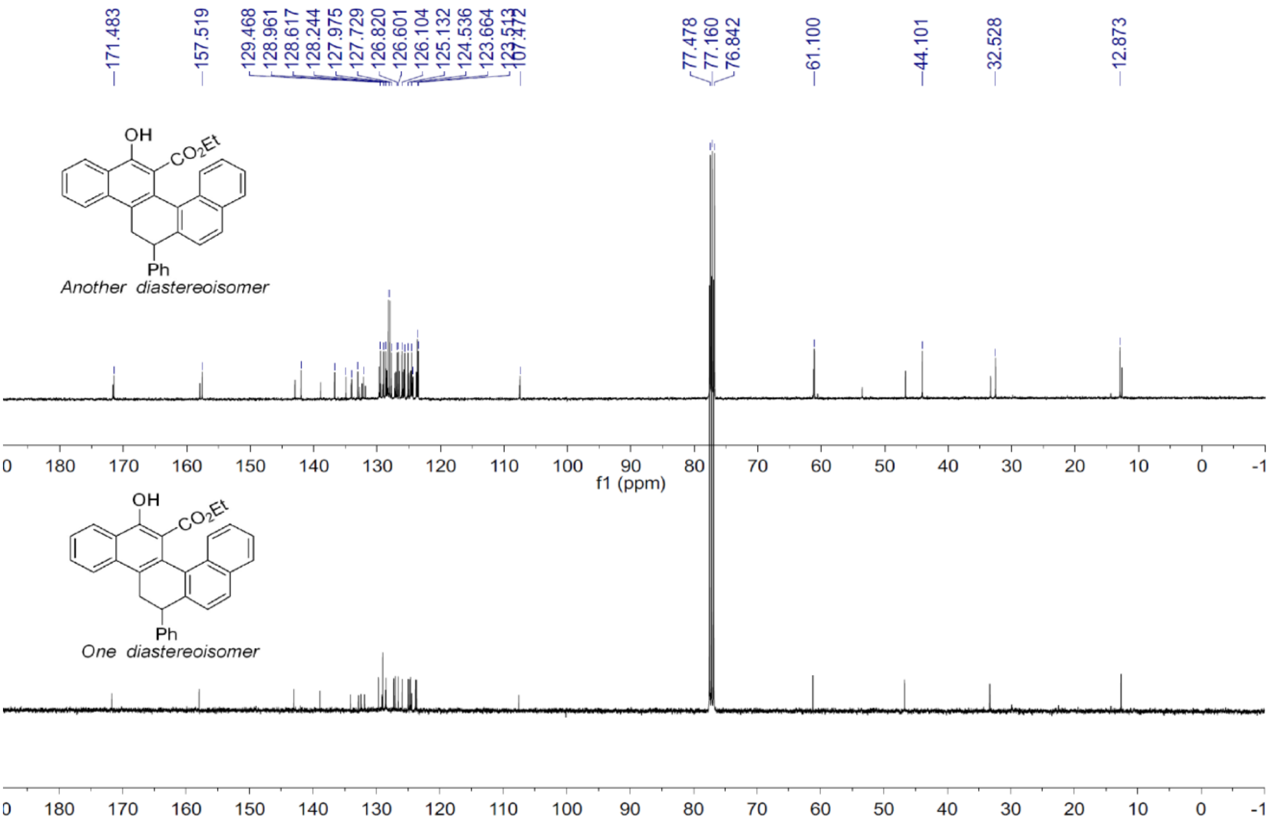

Supplementary Figure 48. ^{13}C NMR (100 MHz, $CDCl_3$) spectrum for compound 18.

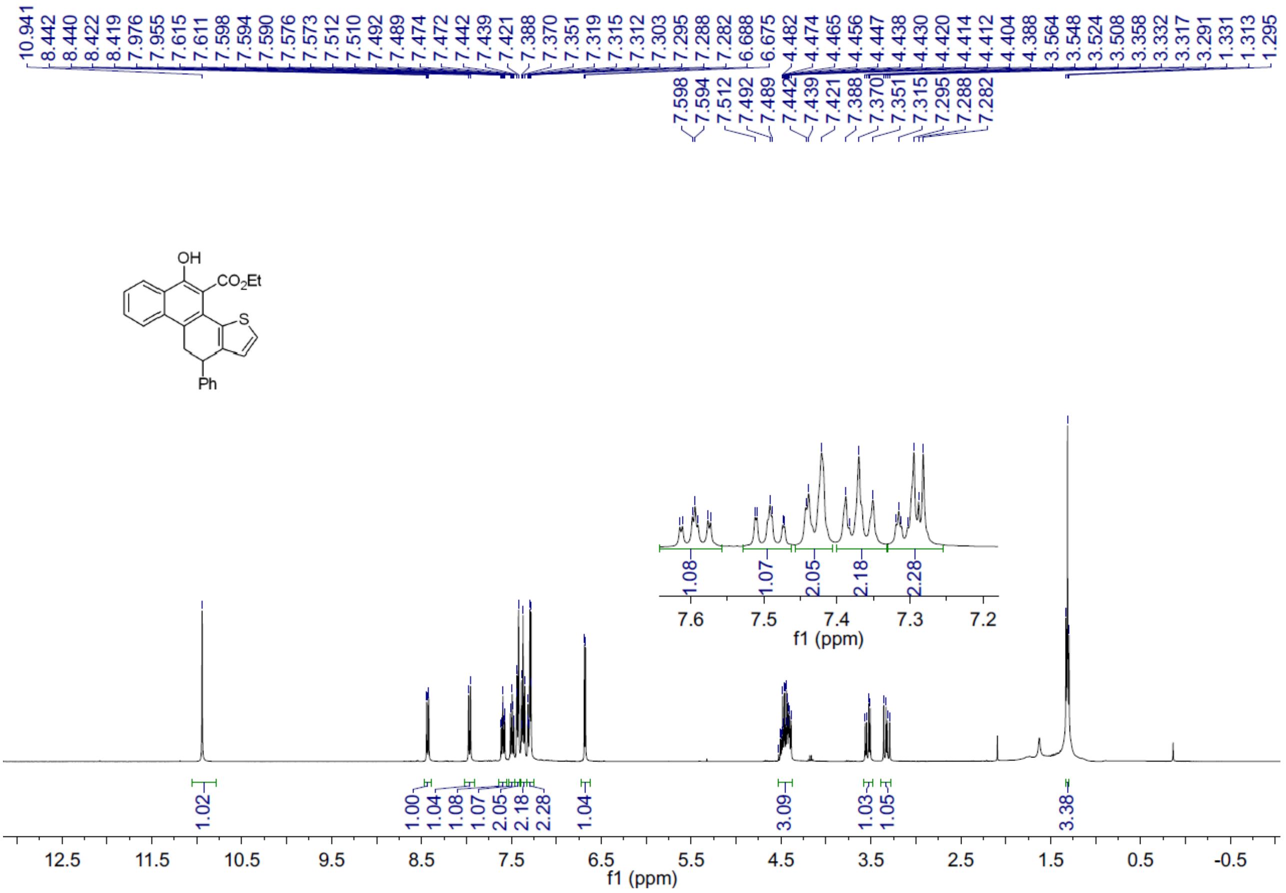

Supplementary Figure 49. **^{1}H NMR (400 MHz, $CDCl_3$) spectrum for compound 19.**

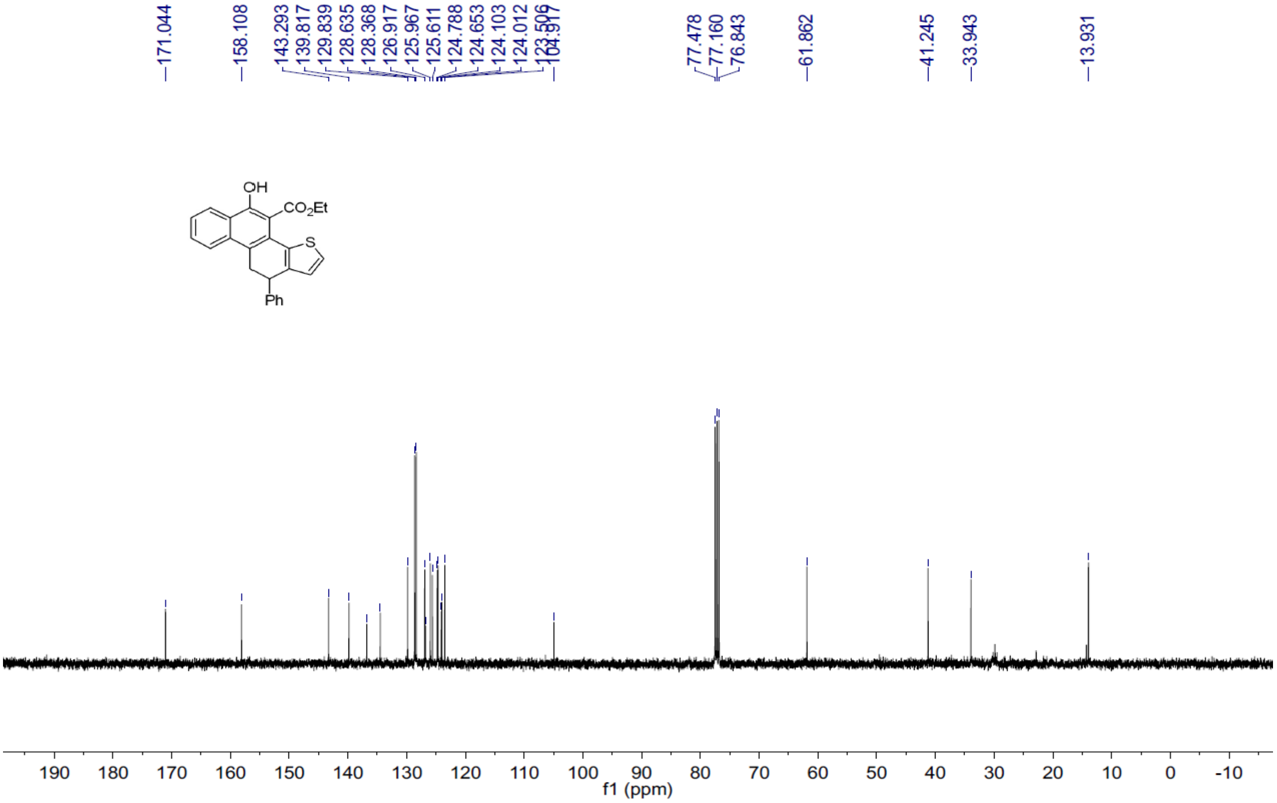

Supplementary Figure 50. **^{13}C NMR (100 MHz, $CDCl_3$) spectrum for compound 19.**

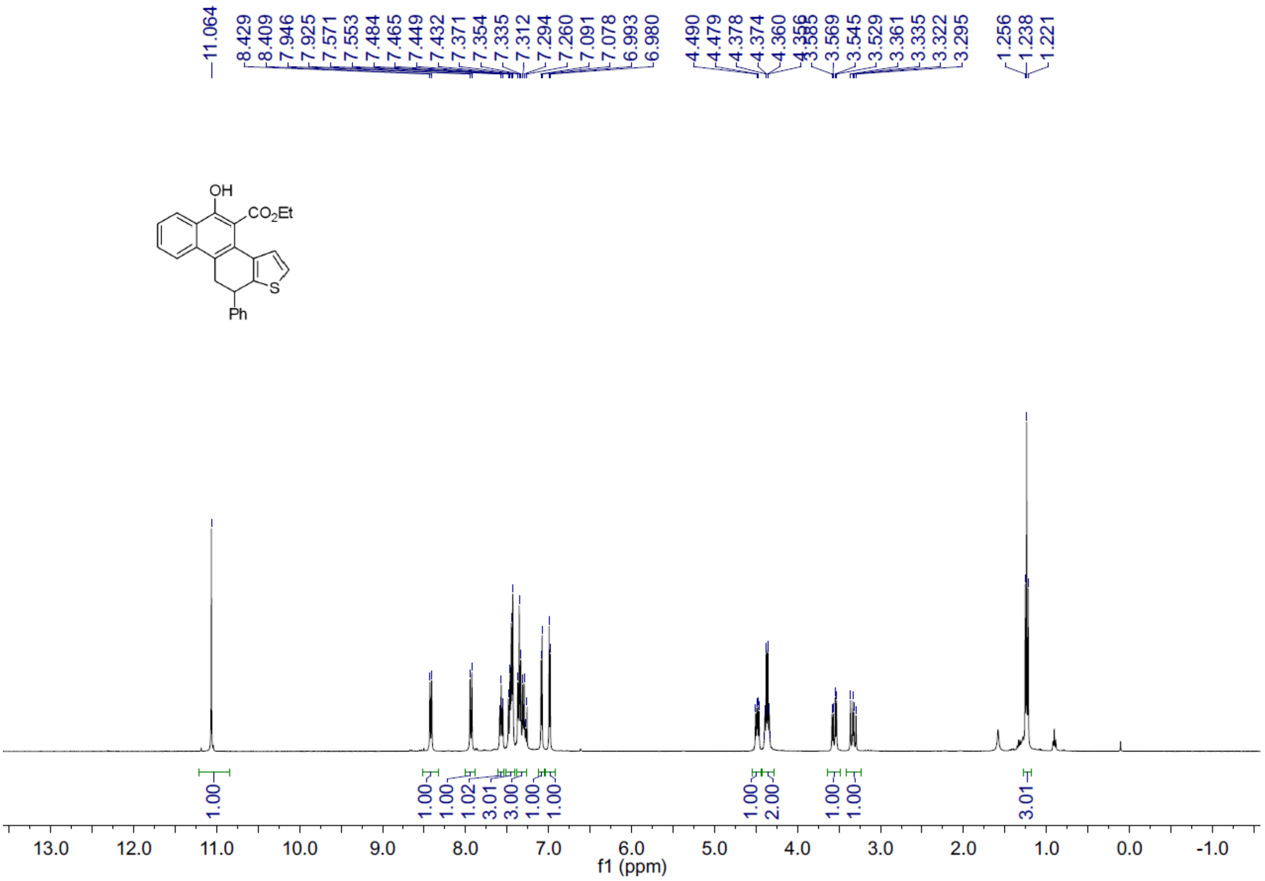

Supplementary Figure 51. ^{1}H NMR (400 MHz, $CDCl_3$) spectrum for compound 20.

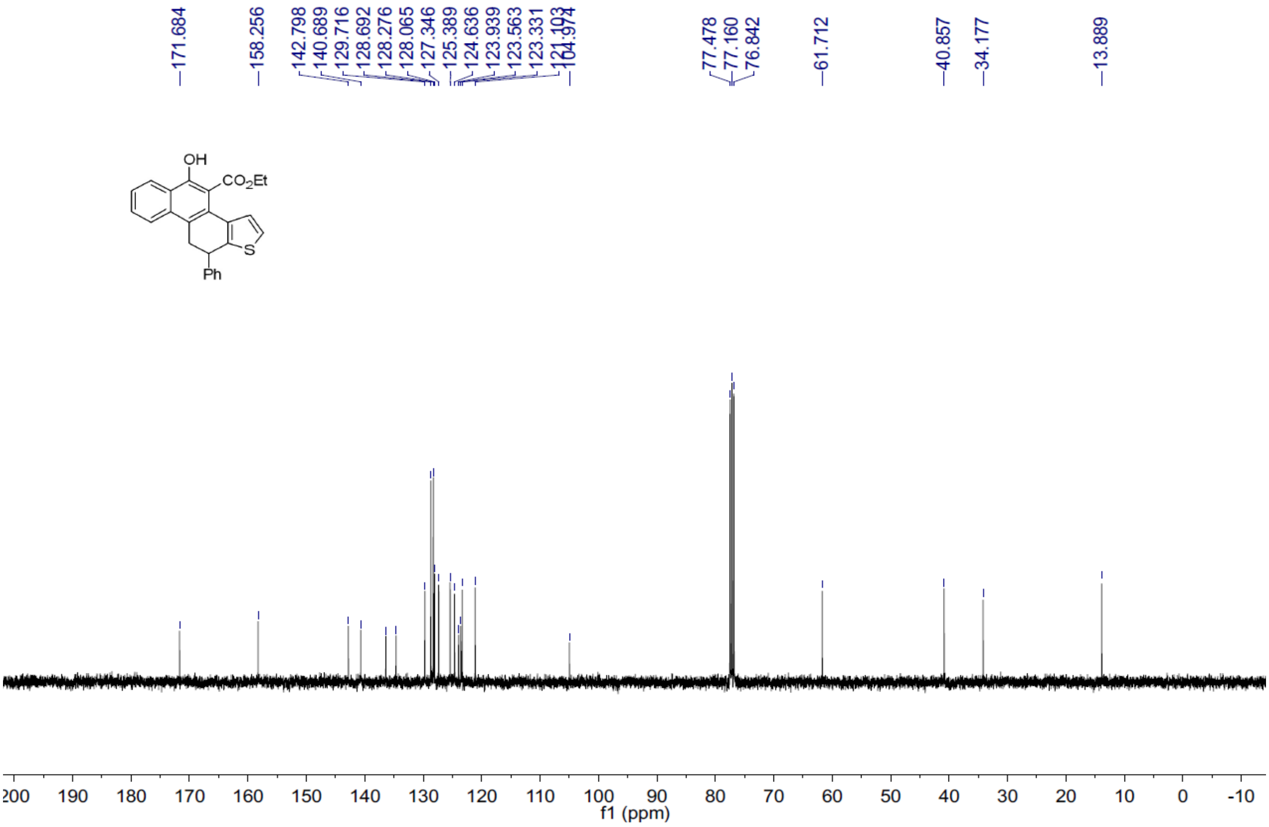

Supplementary Figure 52. ^{13}C NMR (100 MHz, $CDCl_3$) spectrum for compound 20.

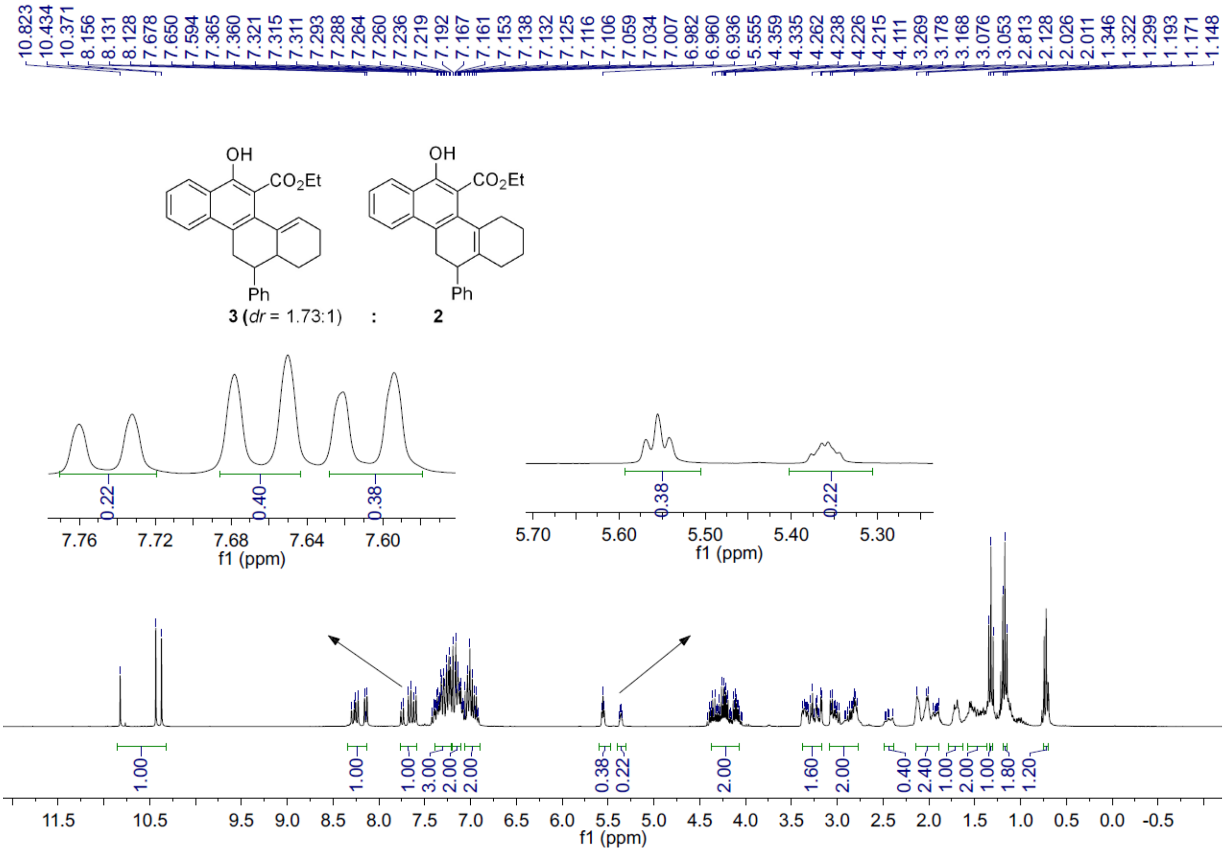

Supplementary Figure 53. ^{1}H NMR (300 MHz, $CDCl_3$) spectrum for compound 21.

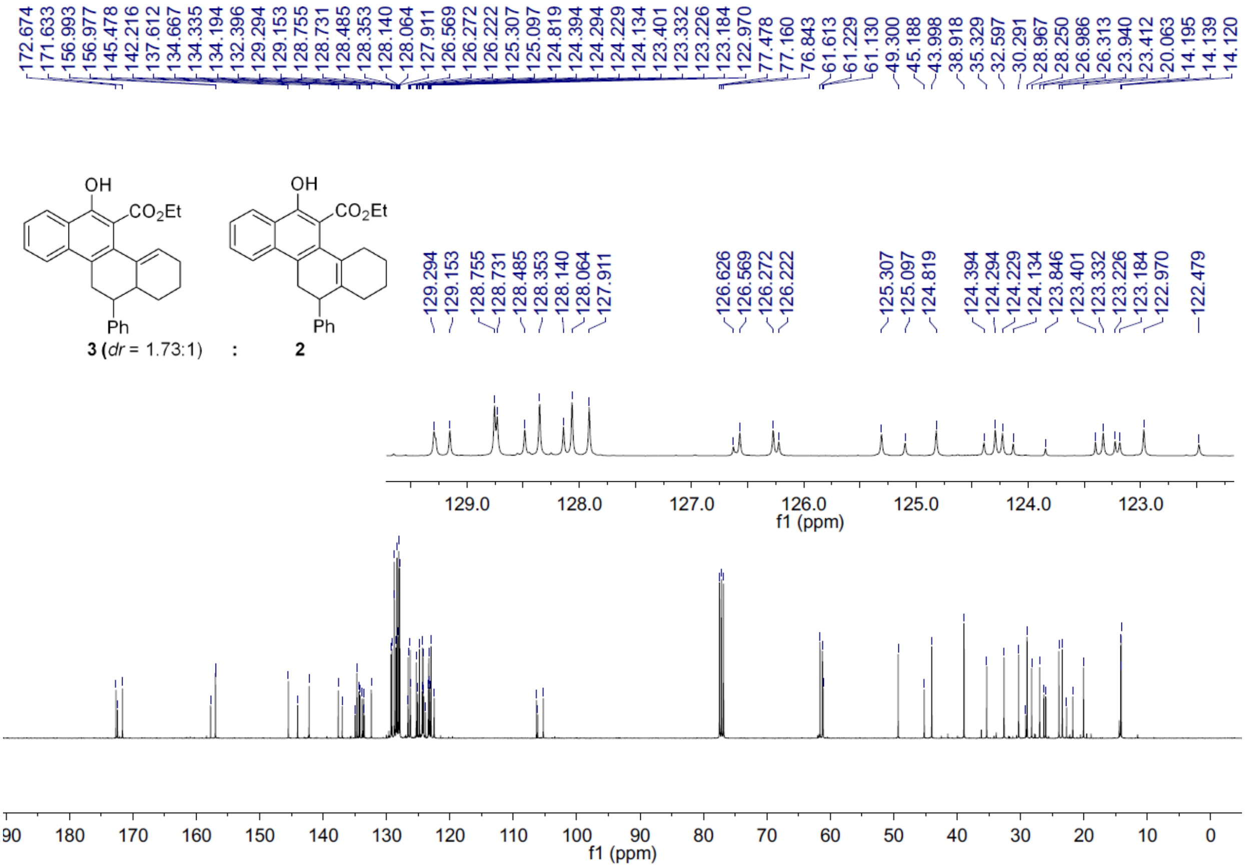

Supplementary Figure 54. ^{13}C NMR (75 MHz, $CDCl_3$) spectrum for compound 21.

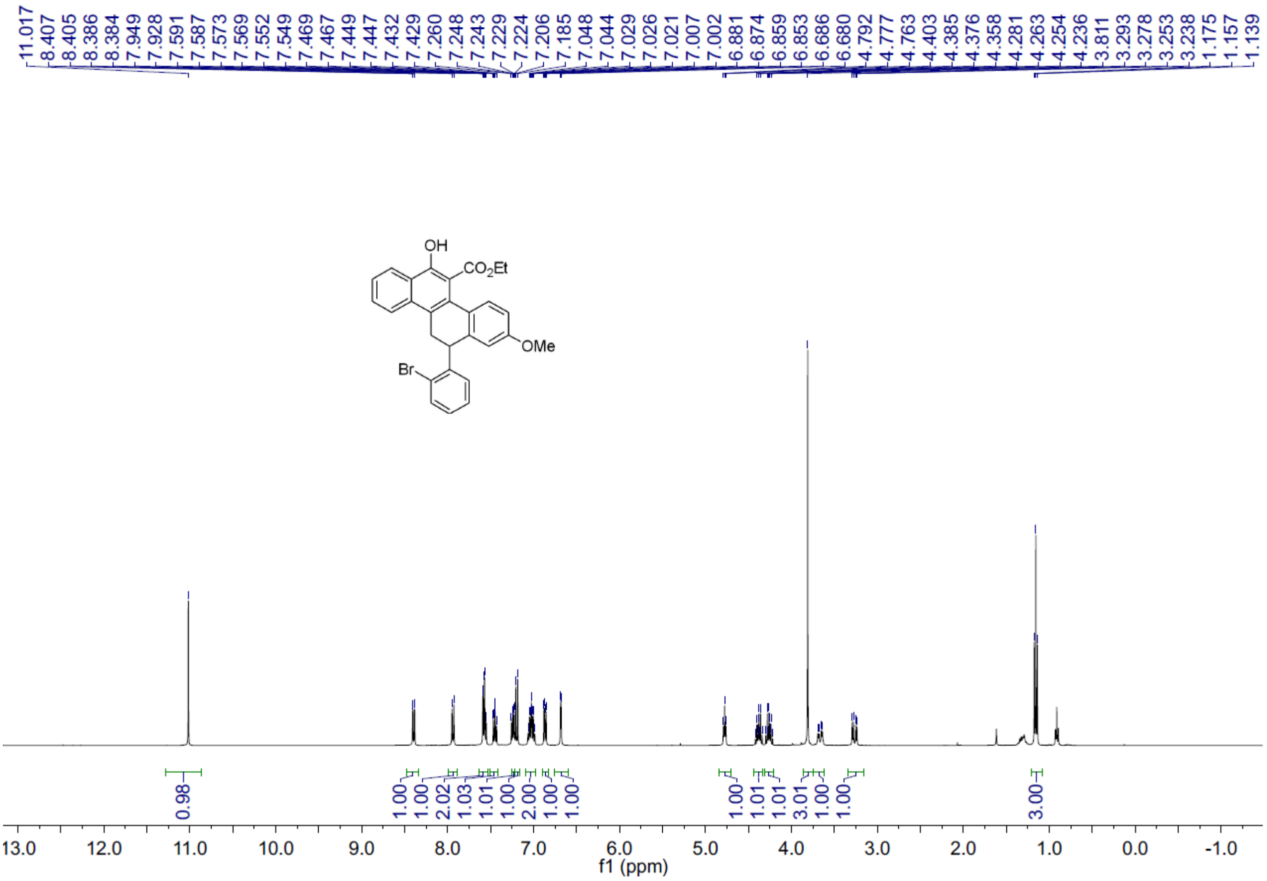

Supplementary Figure 55. ^{1}H NMR (400 MHz, $CDCl_3$) spectrum for compound 22.

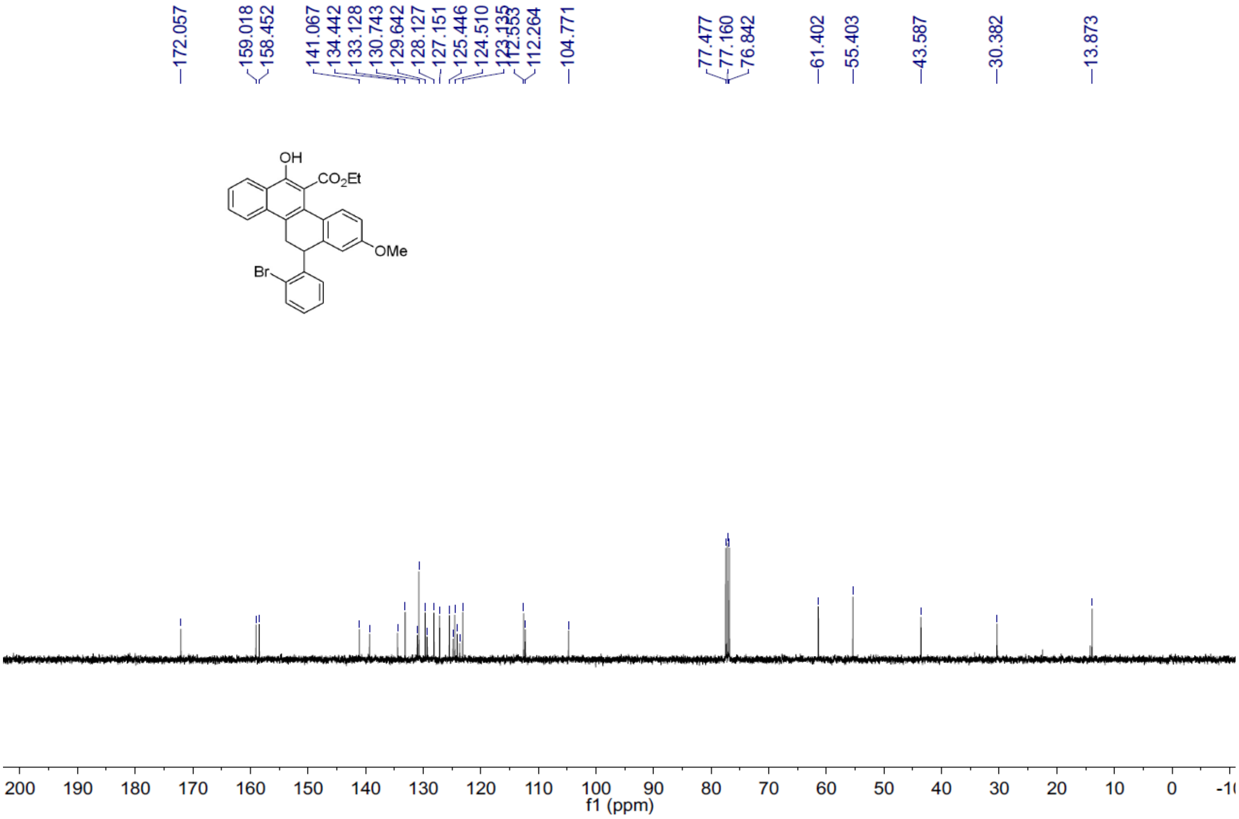

Supplementary Figure 56. ^{13}C NMR (100 MHz, $CDCl_3$) spectrum for compound 22.

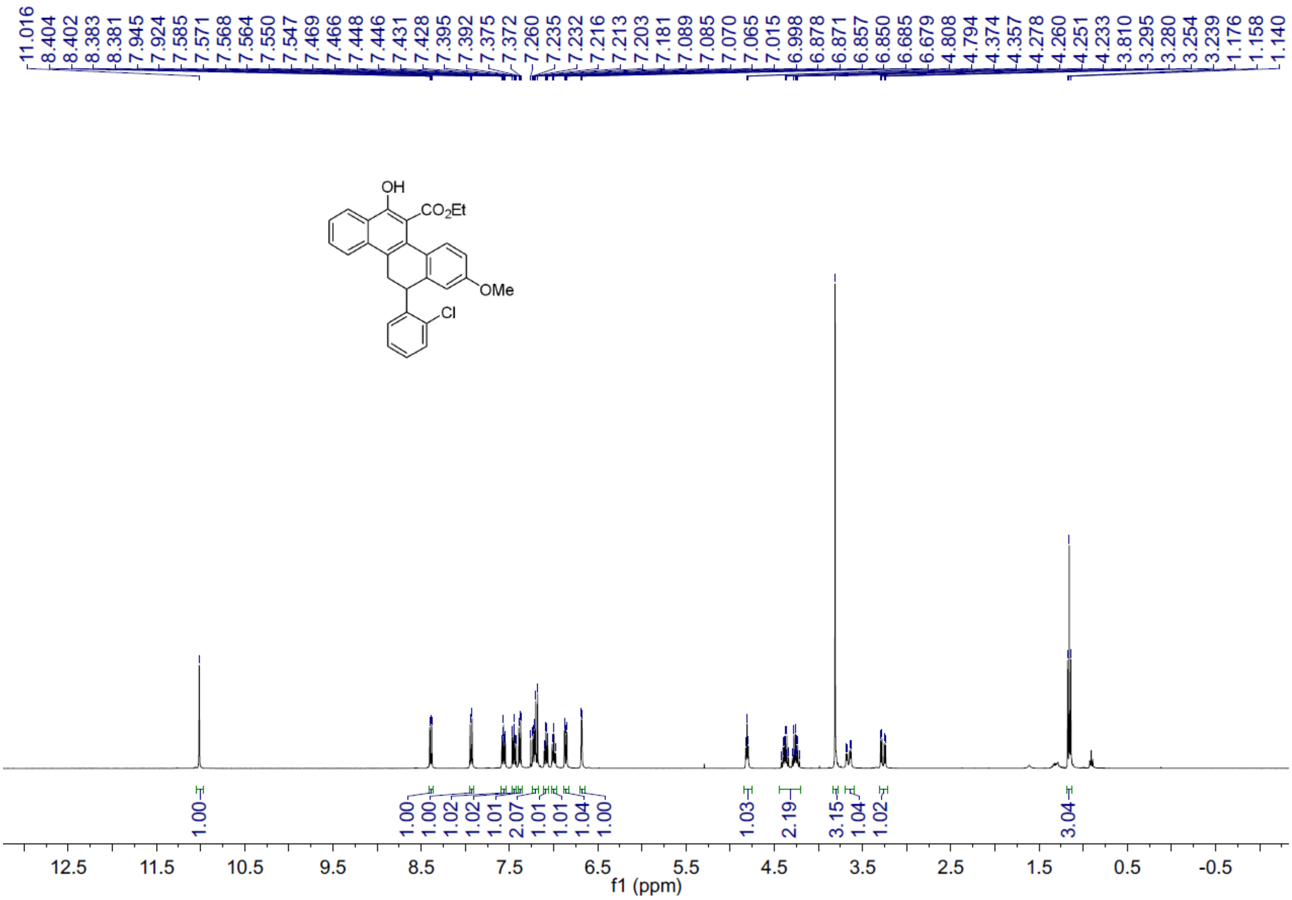

Supplementary Figure 57. **^{1}H NMR (400 MHz, $CDCl_3$) spectrum for compound 23.**

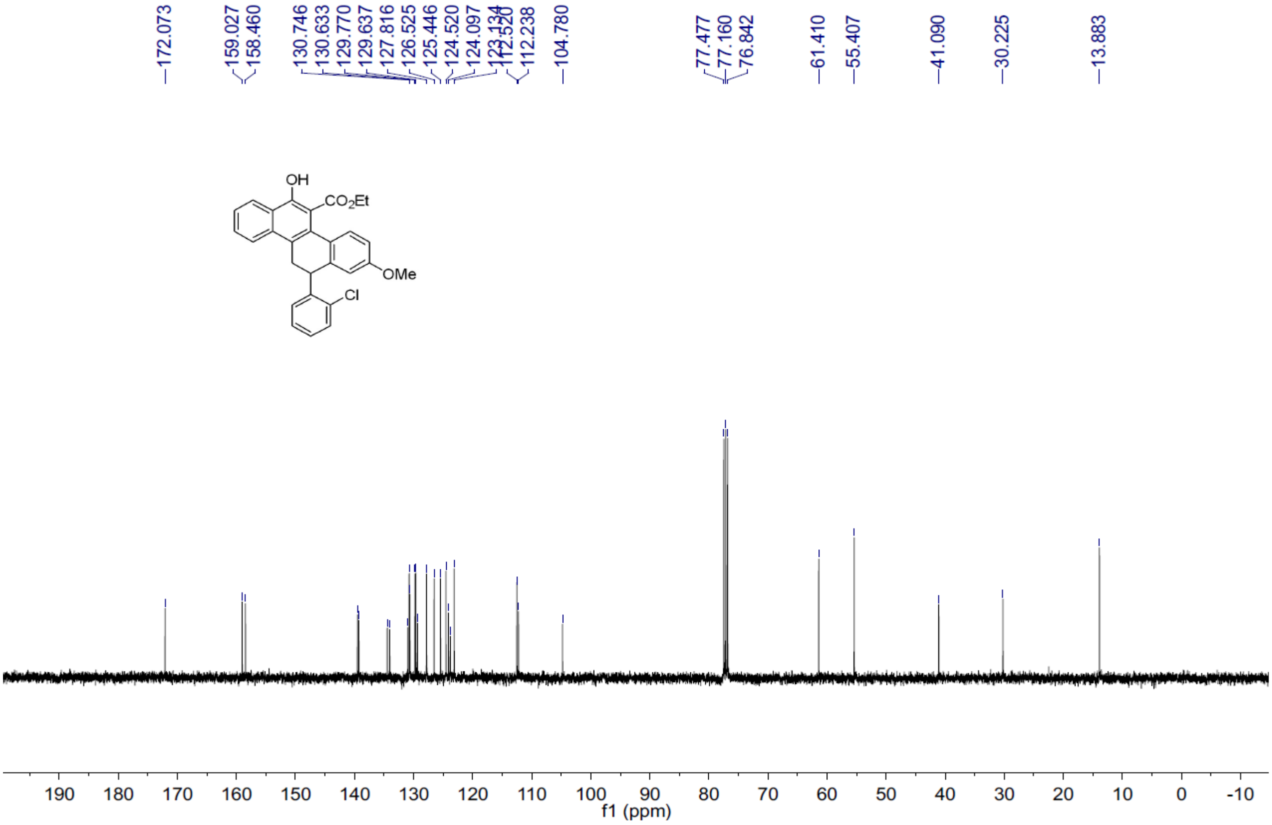

Supplementary Figure 58. **^{13}C NMR (100 MHz, $CDCl_3$) spectrum for compound 23.**

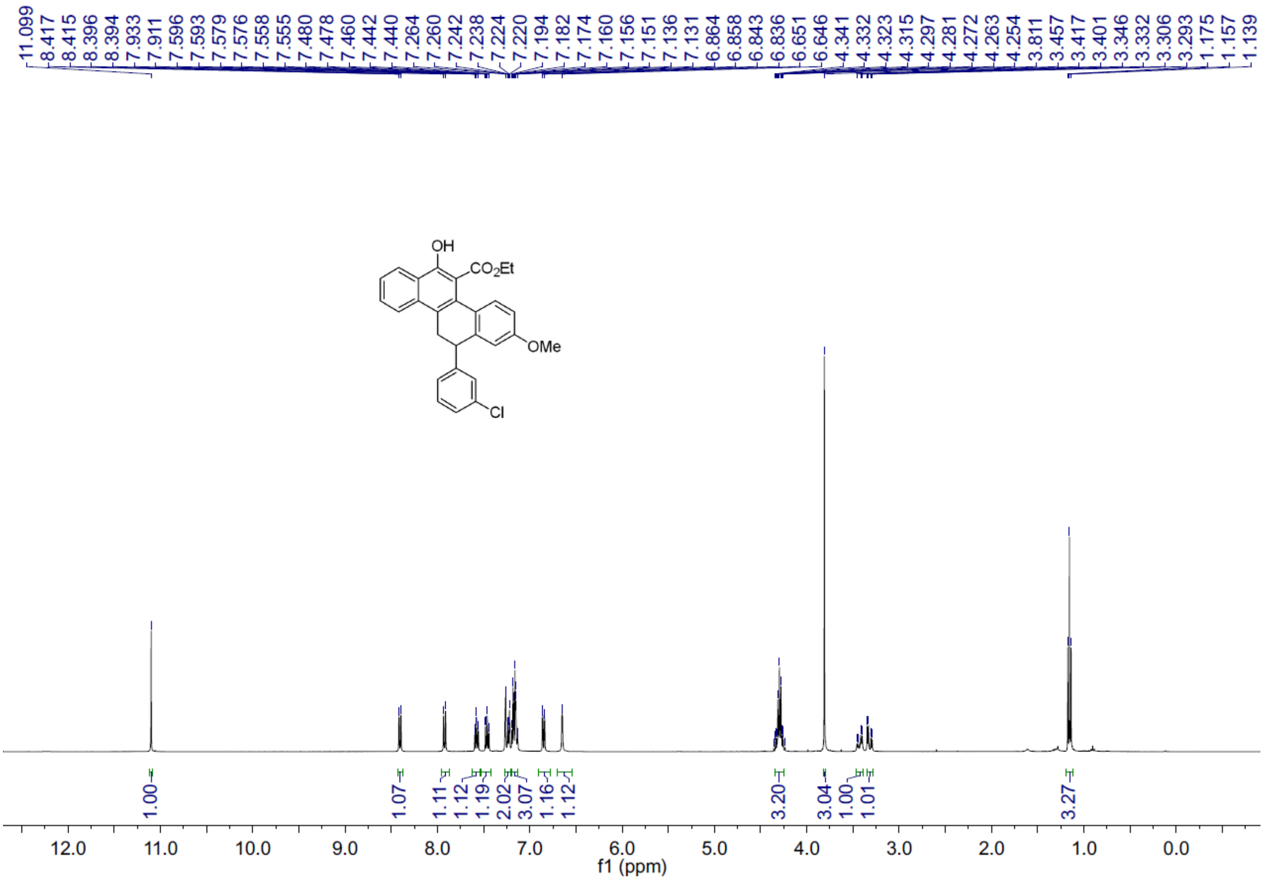

Supplementary Figure 59. ^{1}H **NMR (400 MHz,** $CDCl_3$**) spectrum for compound 24.**

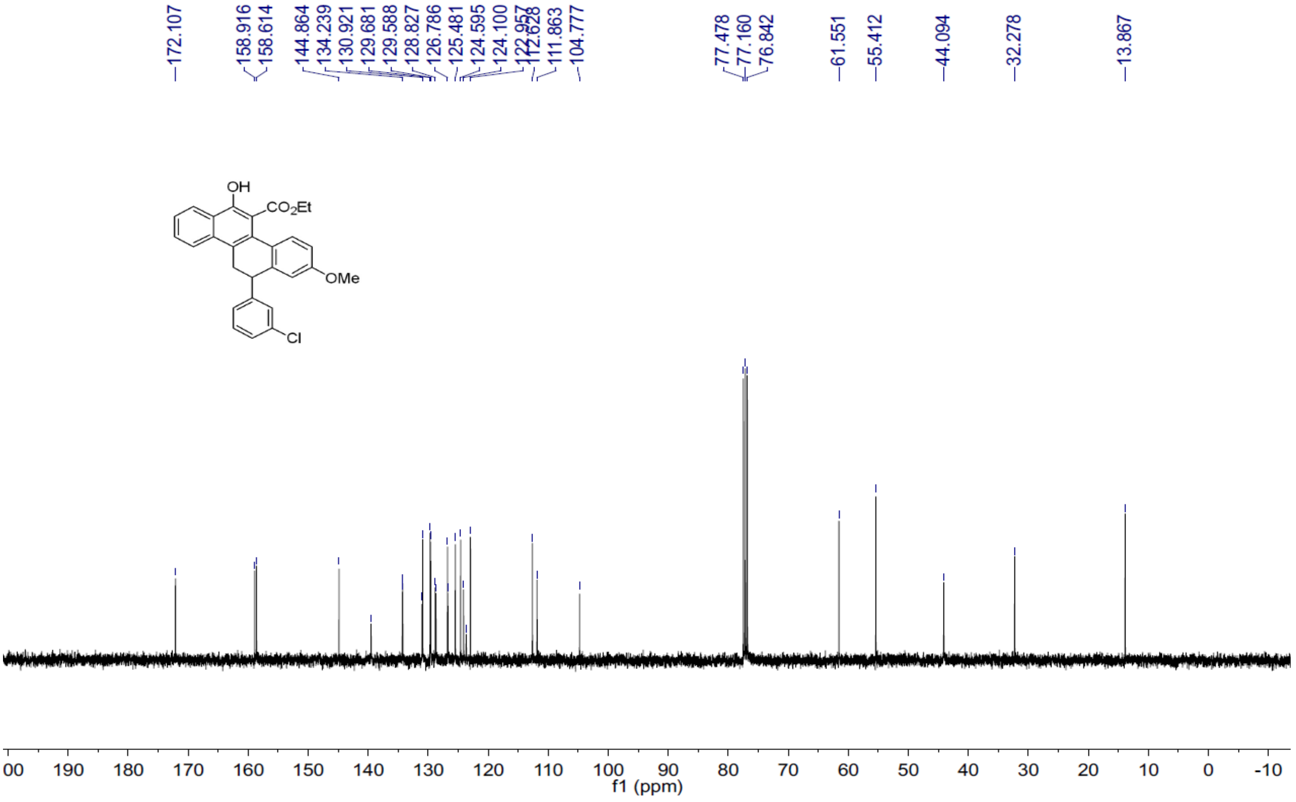

Supplementary Figure 60. ^{13}C **NMR (100 MHz,** $CDCl_3$**) spectrum for compound 24.**

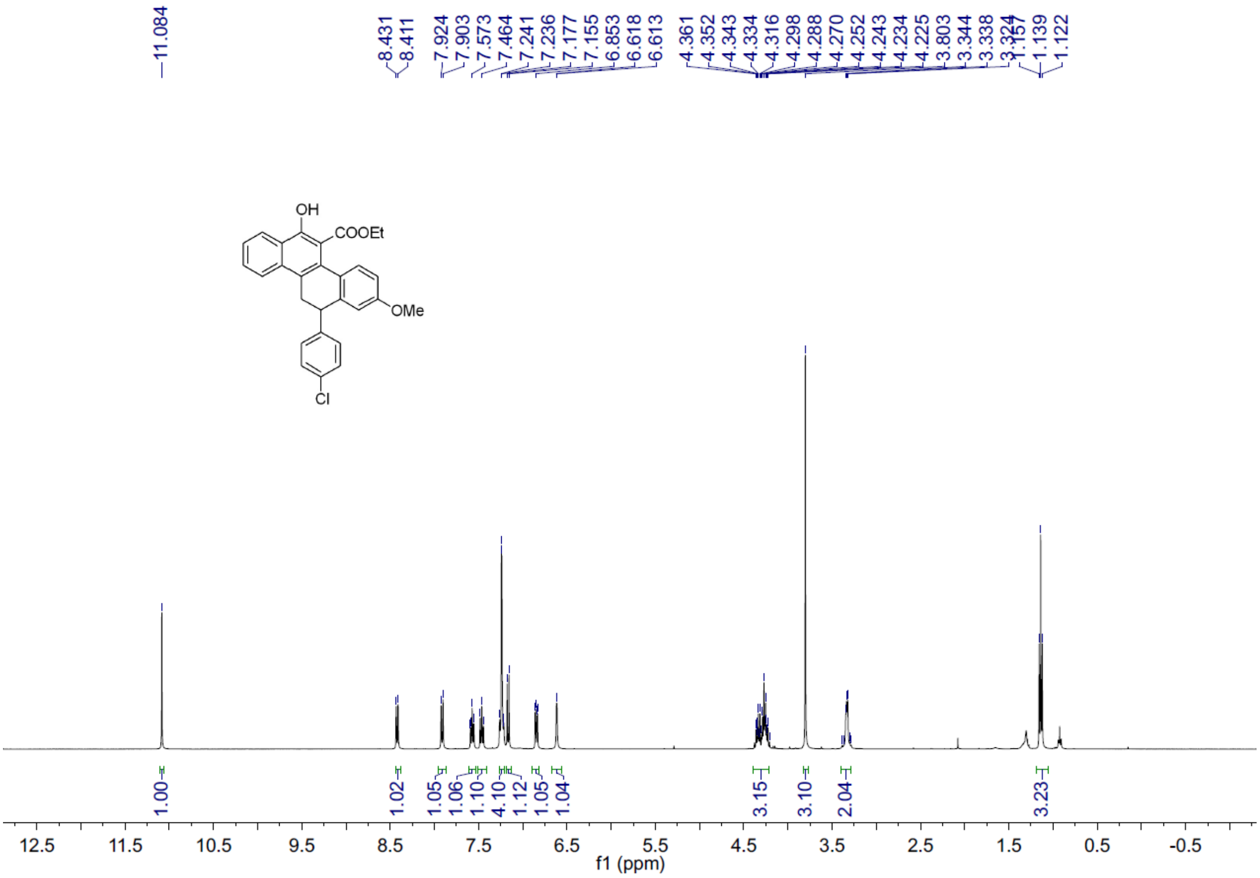

Supplementary Figure 61. ^{1}H NMR (400 MHz, $CDCl_3$) spectrum for compound 25.

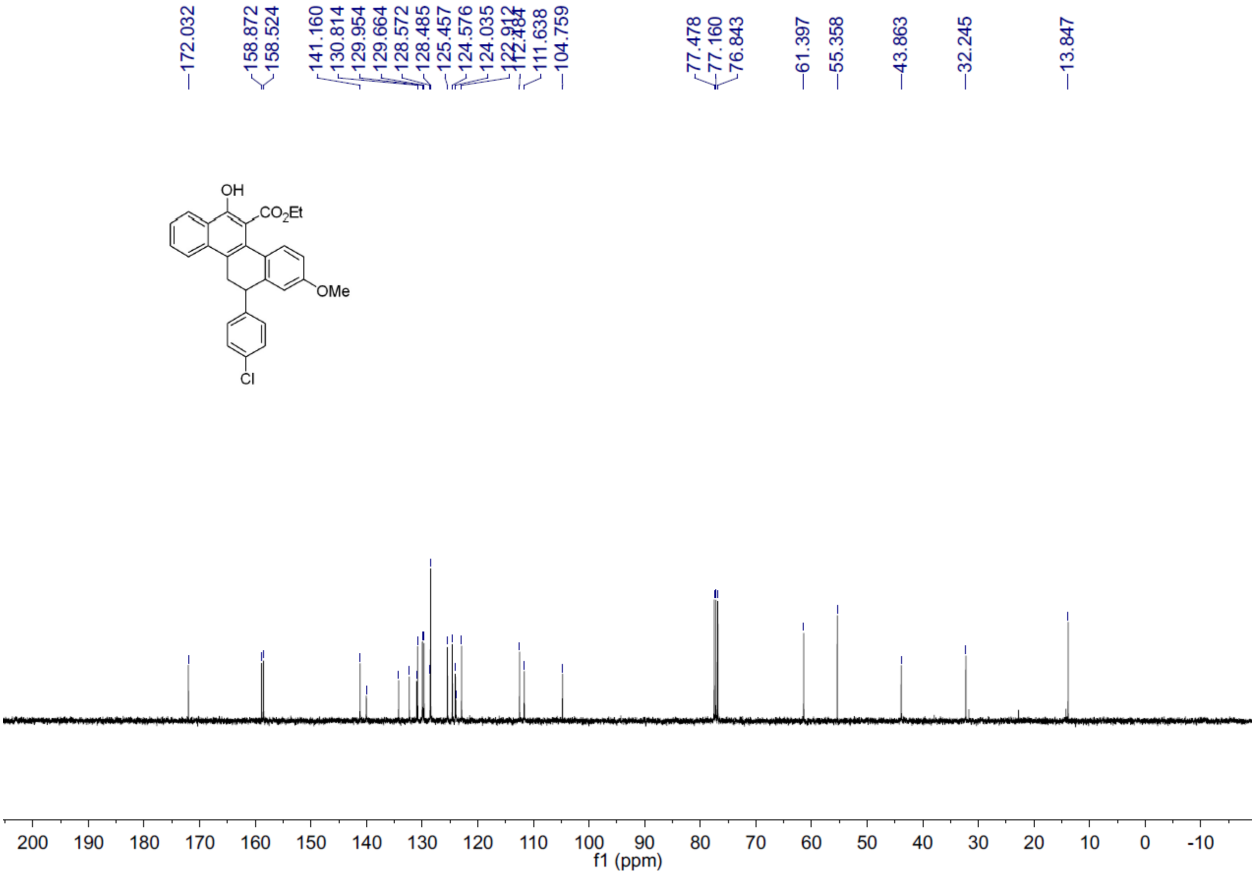

Supplementary Figure 62. ^{13}C NMR (100 MHz, $CDCl_3$) spectrum for compound 25.

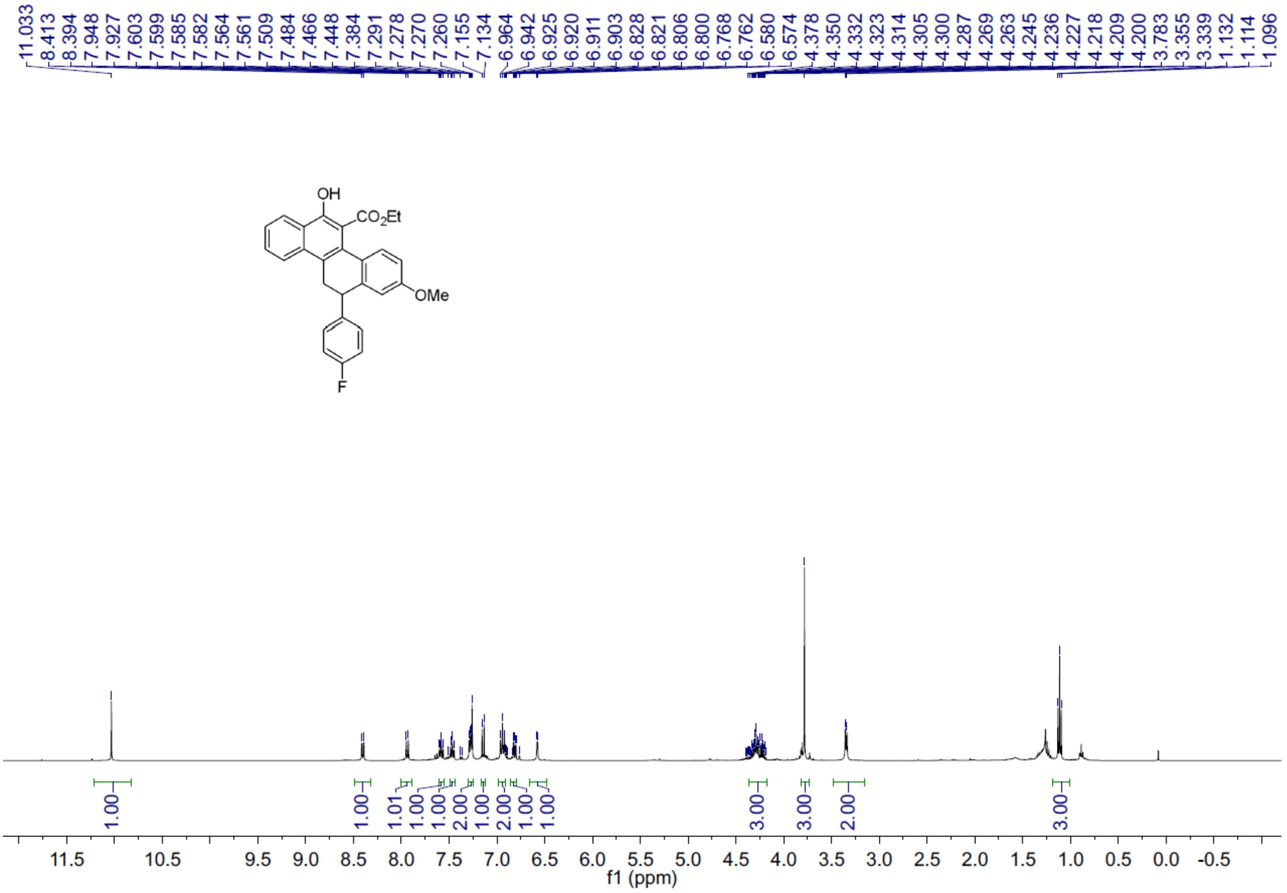

Supplementary Figure 63. ^{1}H NMR (400 MHz, $CDCl_3$) spectrum for compound 26.

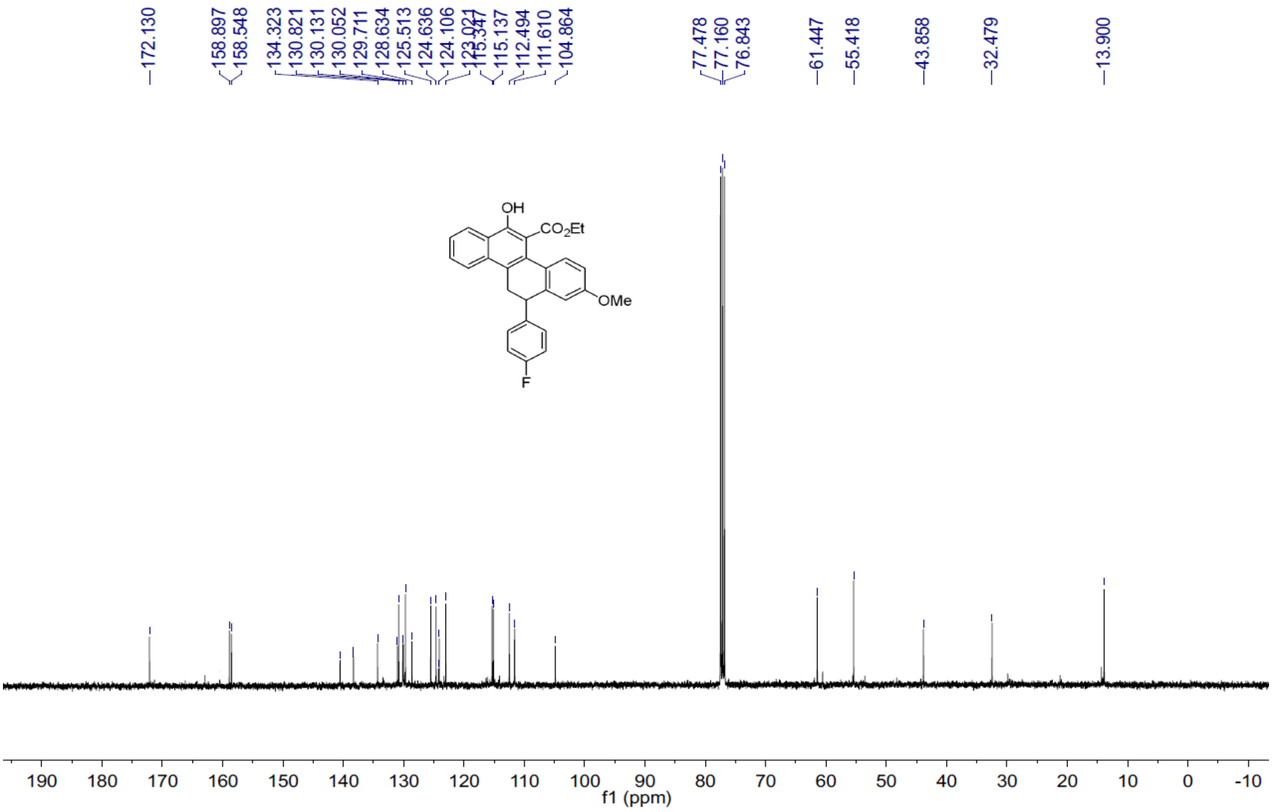

Supplementary Figure 64. ^{13}C NMR (100 MHz, $CDCl_3$) spectrum for compound 26.

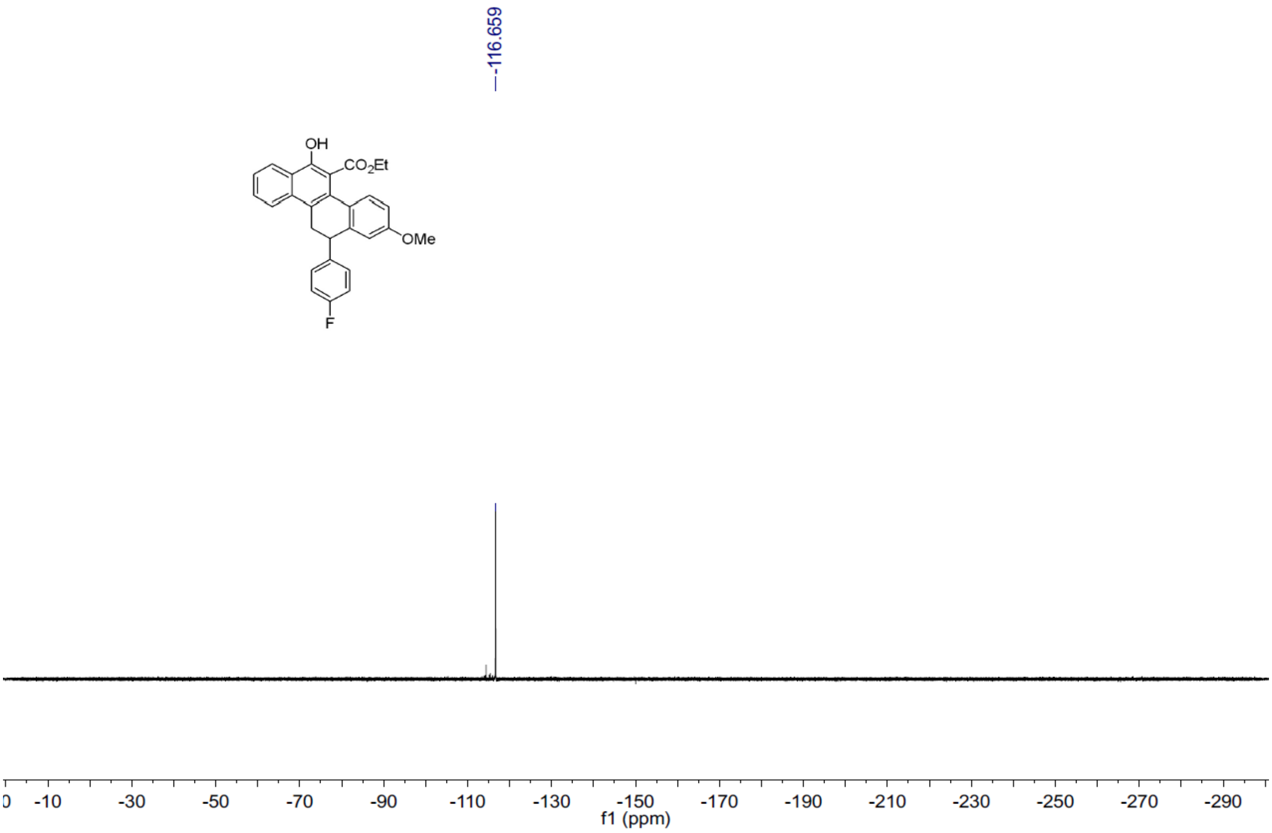

Supplementary Figure 65. ^{19}F NMR (376 MHz, CDCl$_3$) spectrum for compound 26.

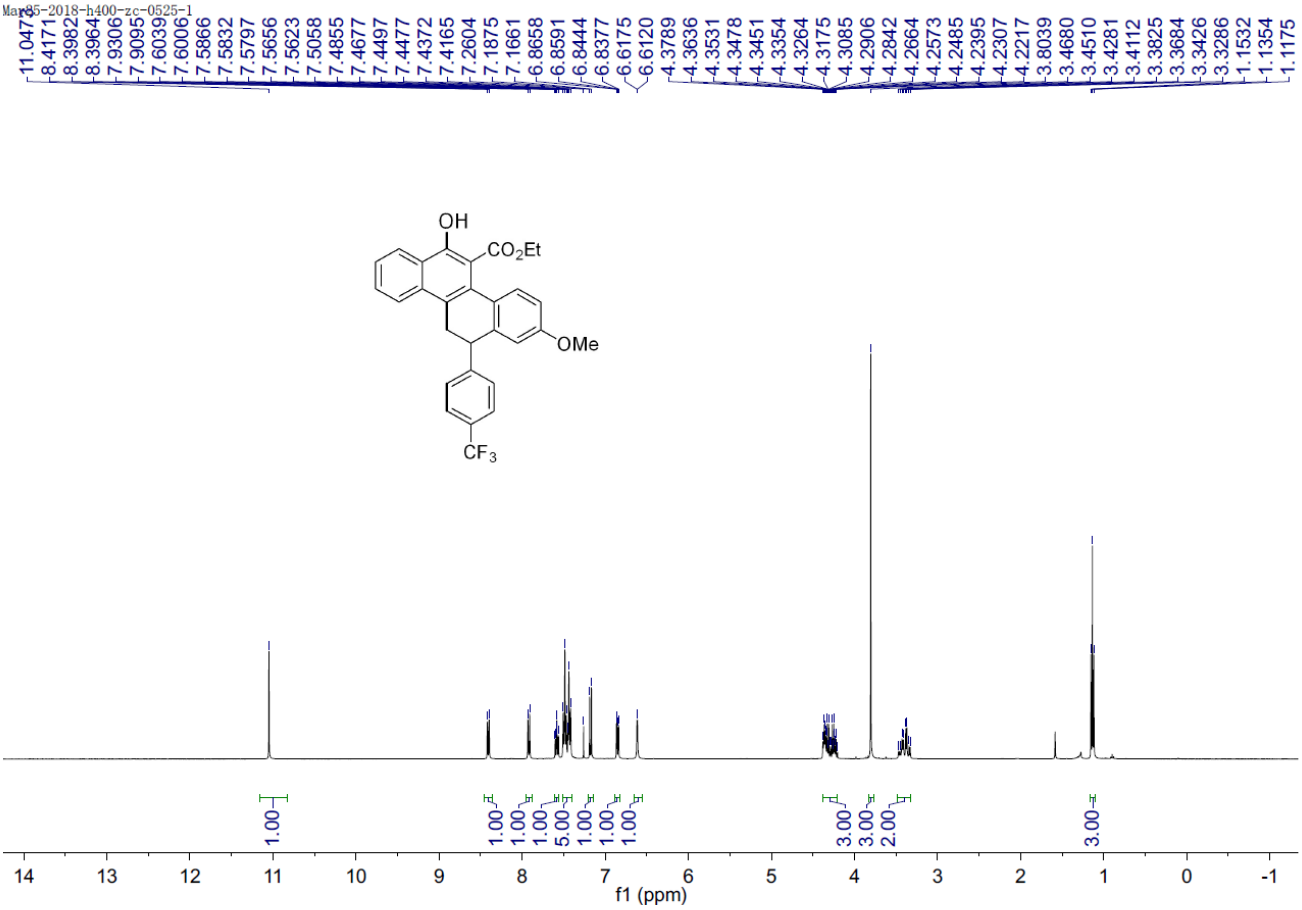

Supplementary Figure 66. ^{1}H NMR (400 MHz, CDCl$_3$) spectrum for compound 27.

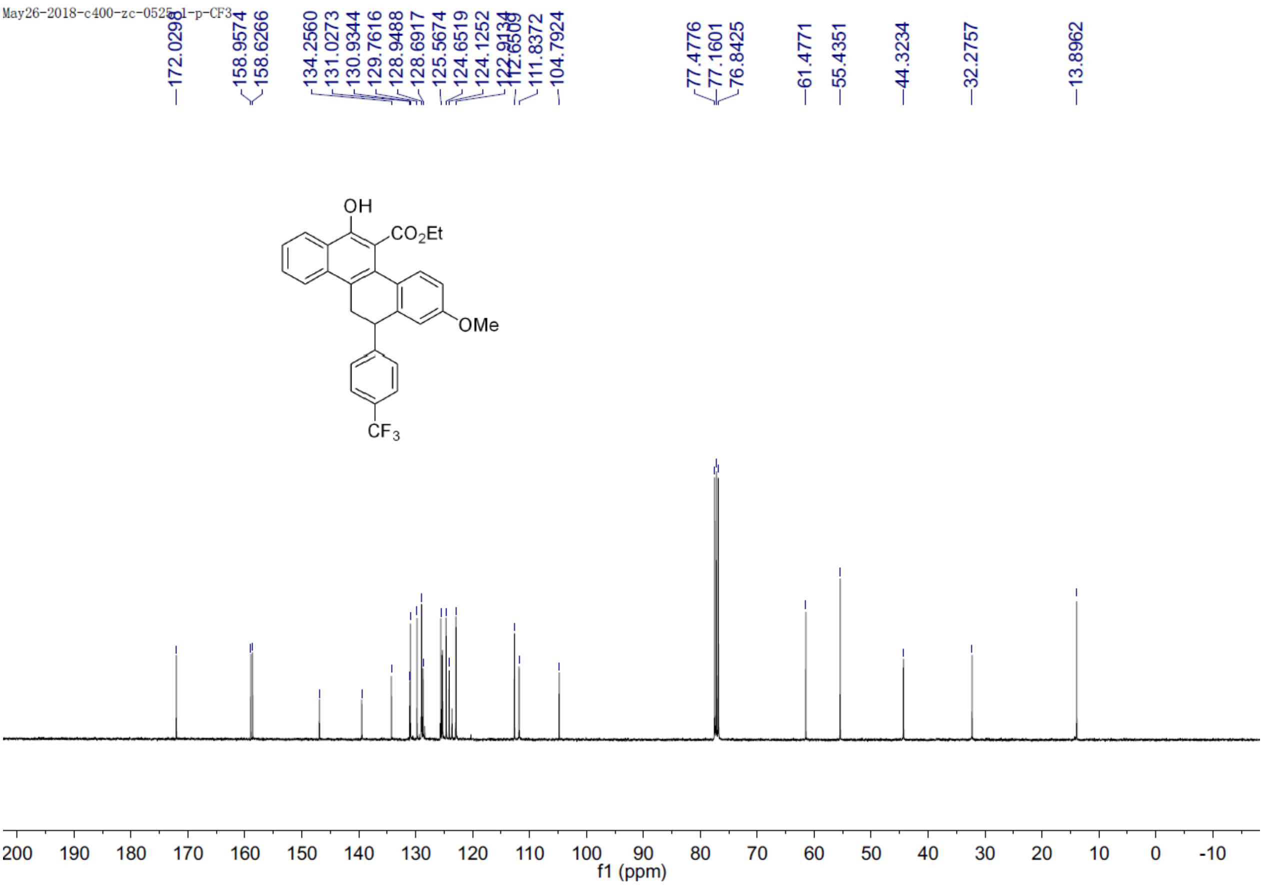

Supplementary Figure 67. ^{13}C NMR (100 MHz, $CDCl_3$) spectrum for compound 27.

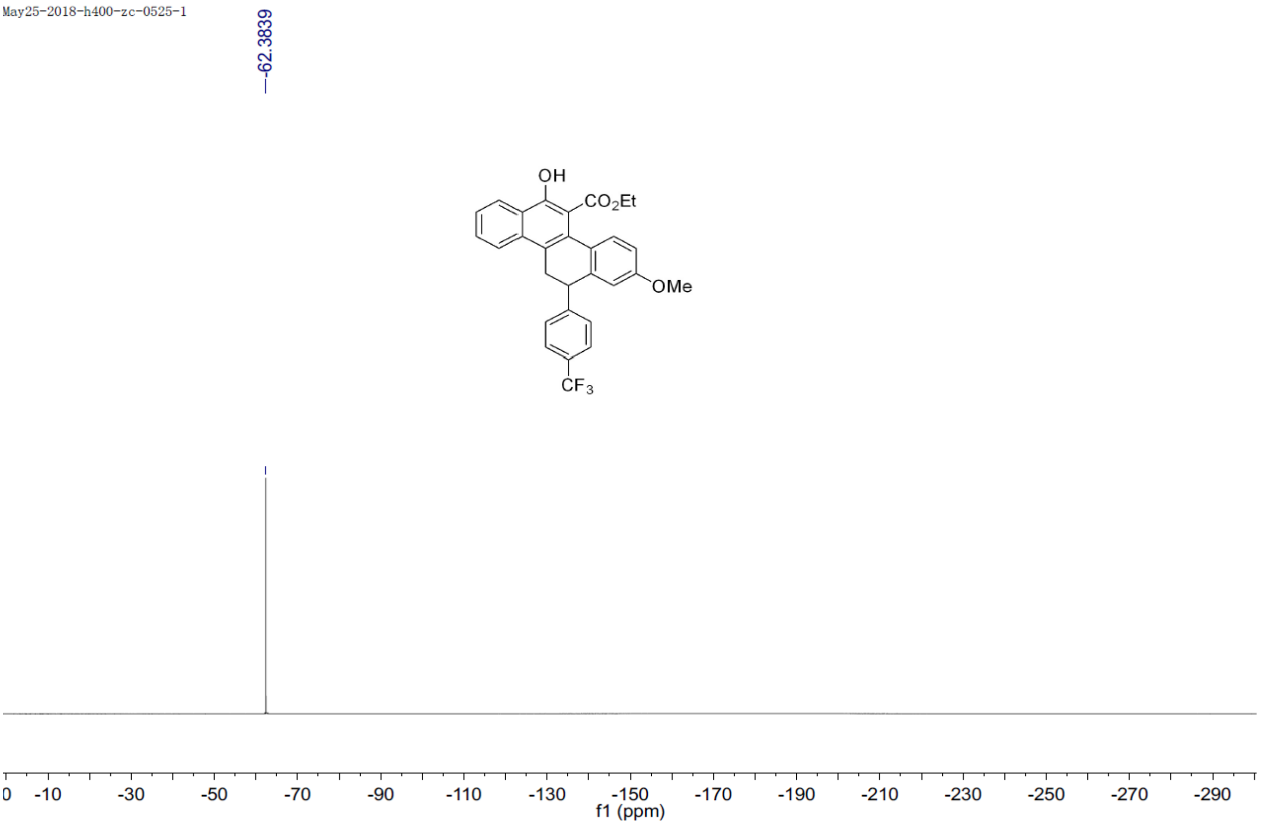

Supplementary Figure 68. ^{19}F NMR (376 MHz, $CDCl_3$) spectrum for compound 27.

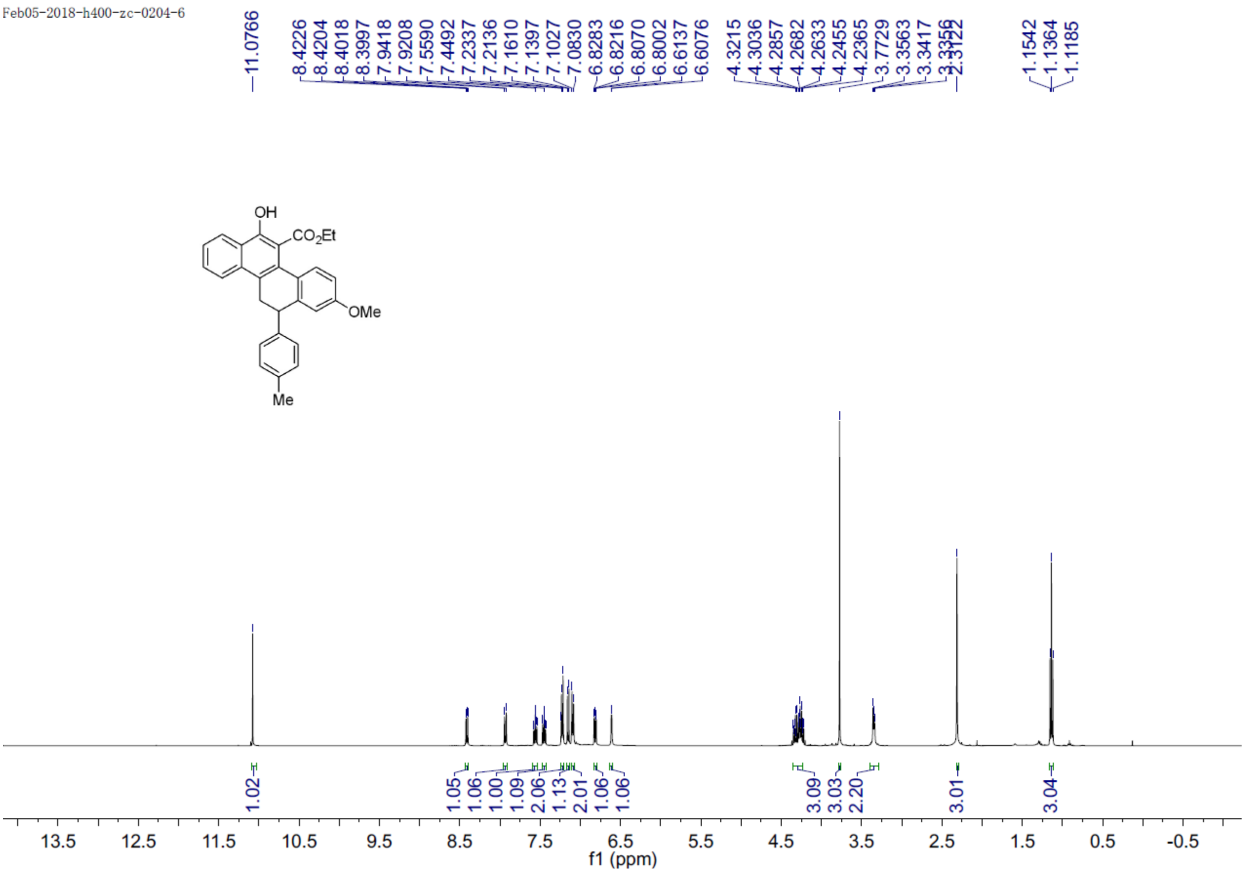

Supplementary Figure 69. ^{1}H NMR (400 MHz, $CDCl_3$) spectrum for compound 28.

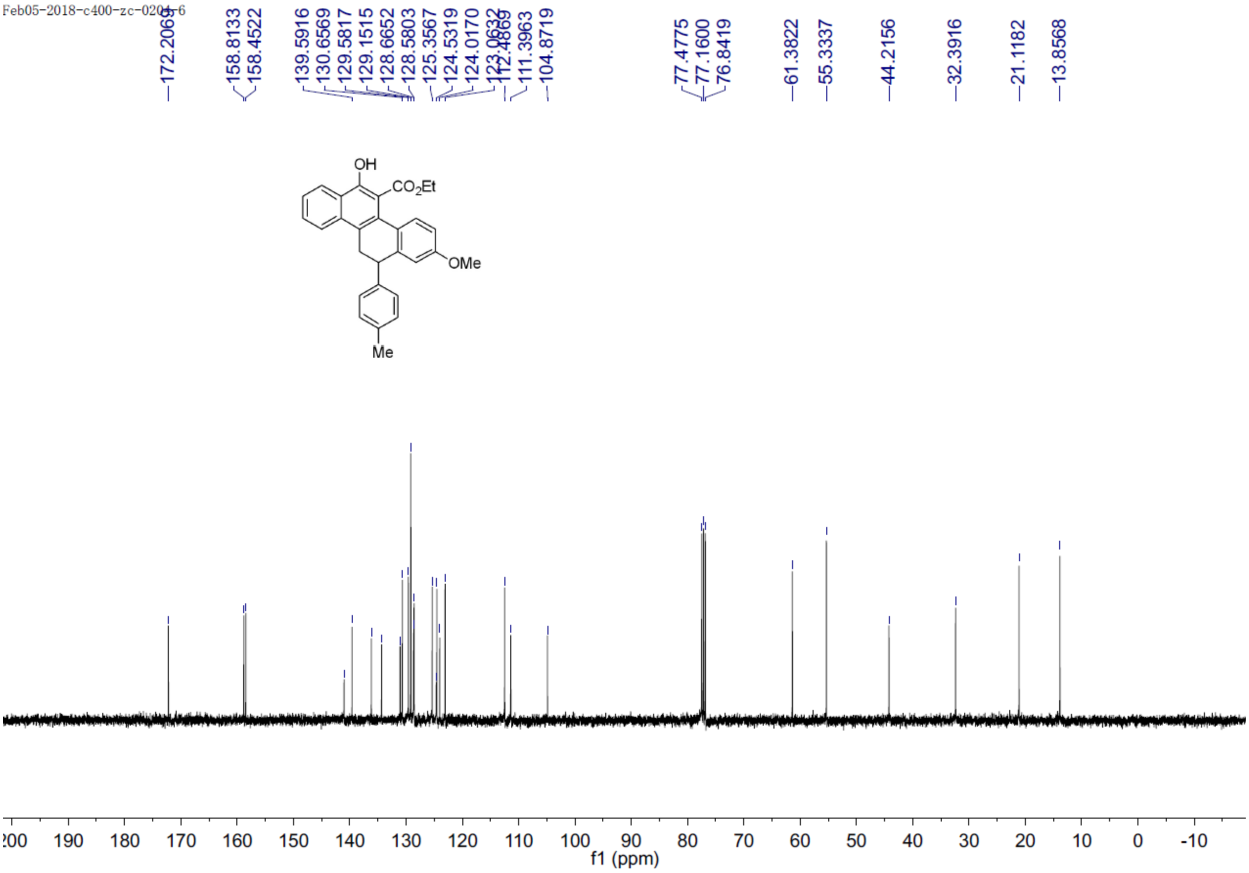

Supplementary Figure 70. ^{13}C NMR (100 MHz, $CDCl_3$) spectrum for compound 28.

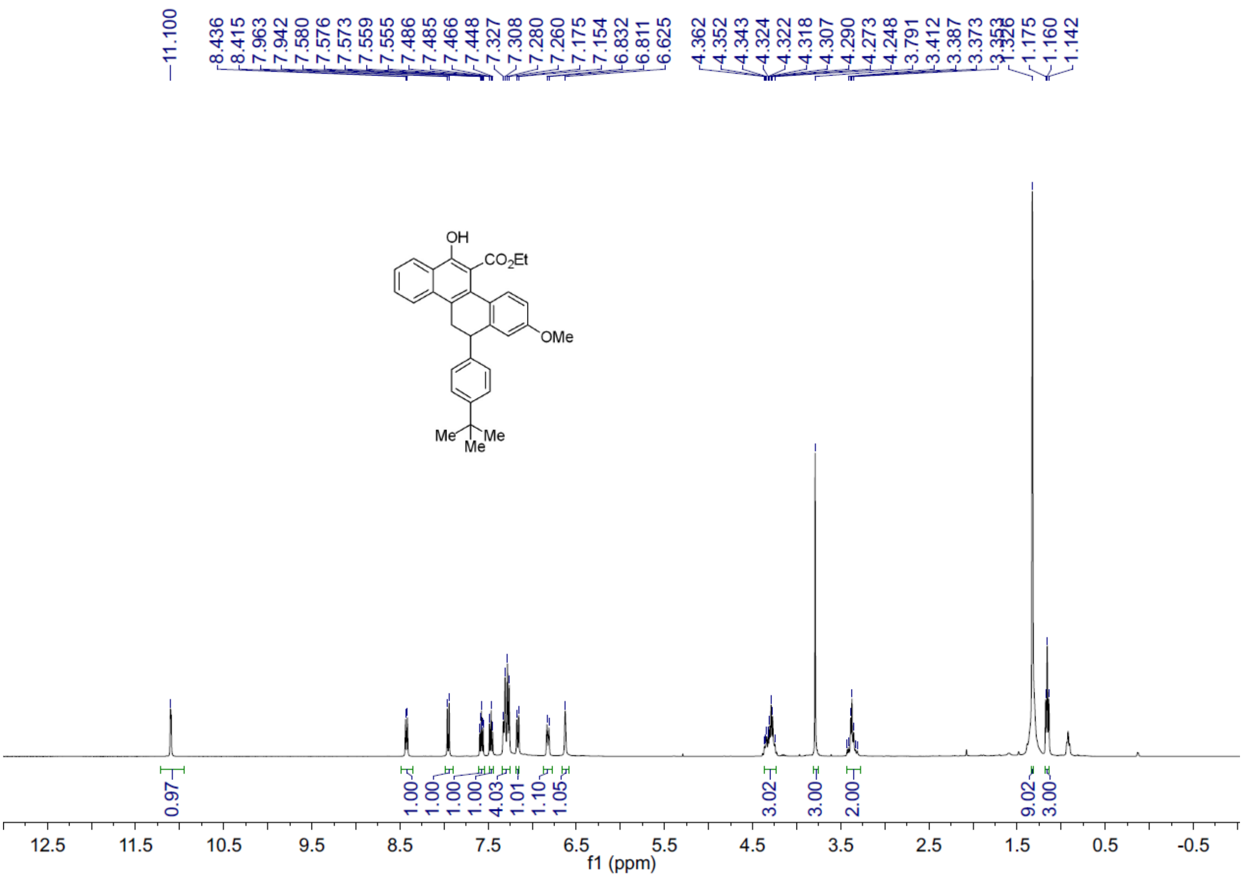

Supplementary Figure 71. ^{1}H NMR (400 MHz, $CDCl_3$) spectrum for compound 29.

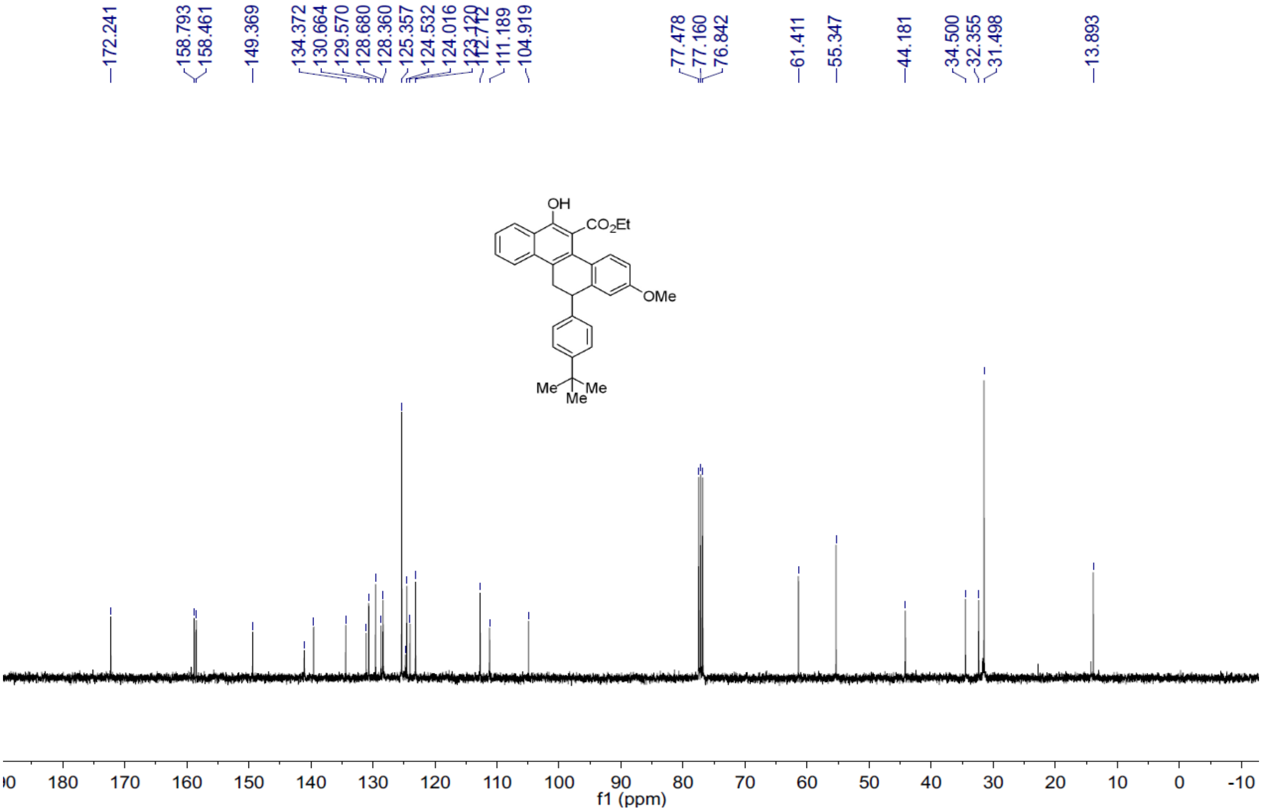

Supplementary Figure 72. ^{13}C NMR (100 MHz, $CDCl_3$) spectrum for compound 29.

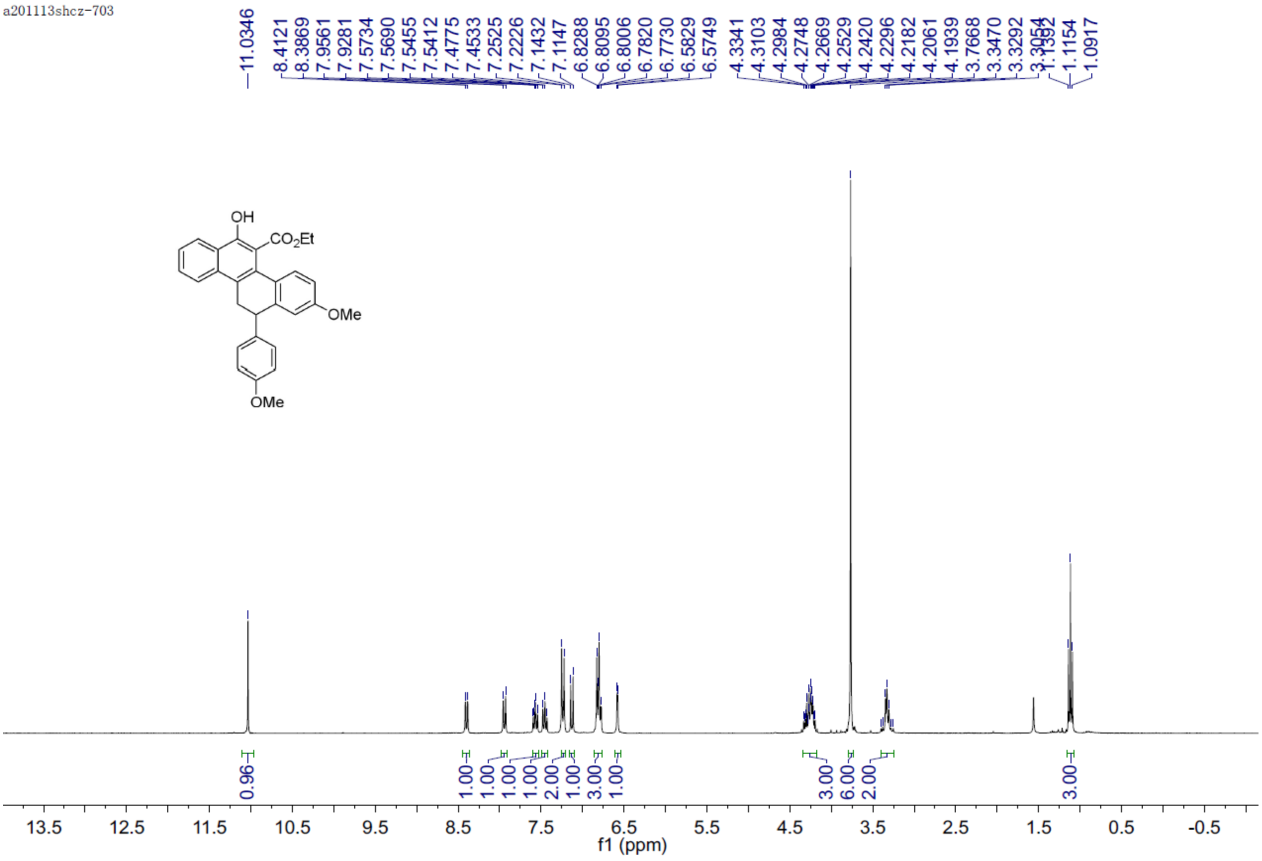

Supplementary Figure 73. ^{1}**H NMR (400 MHz, CDCl**$_3$**) spectrum for compound 30.**

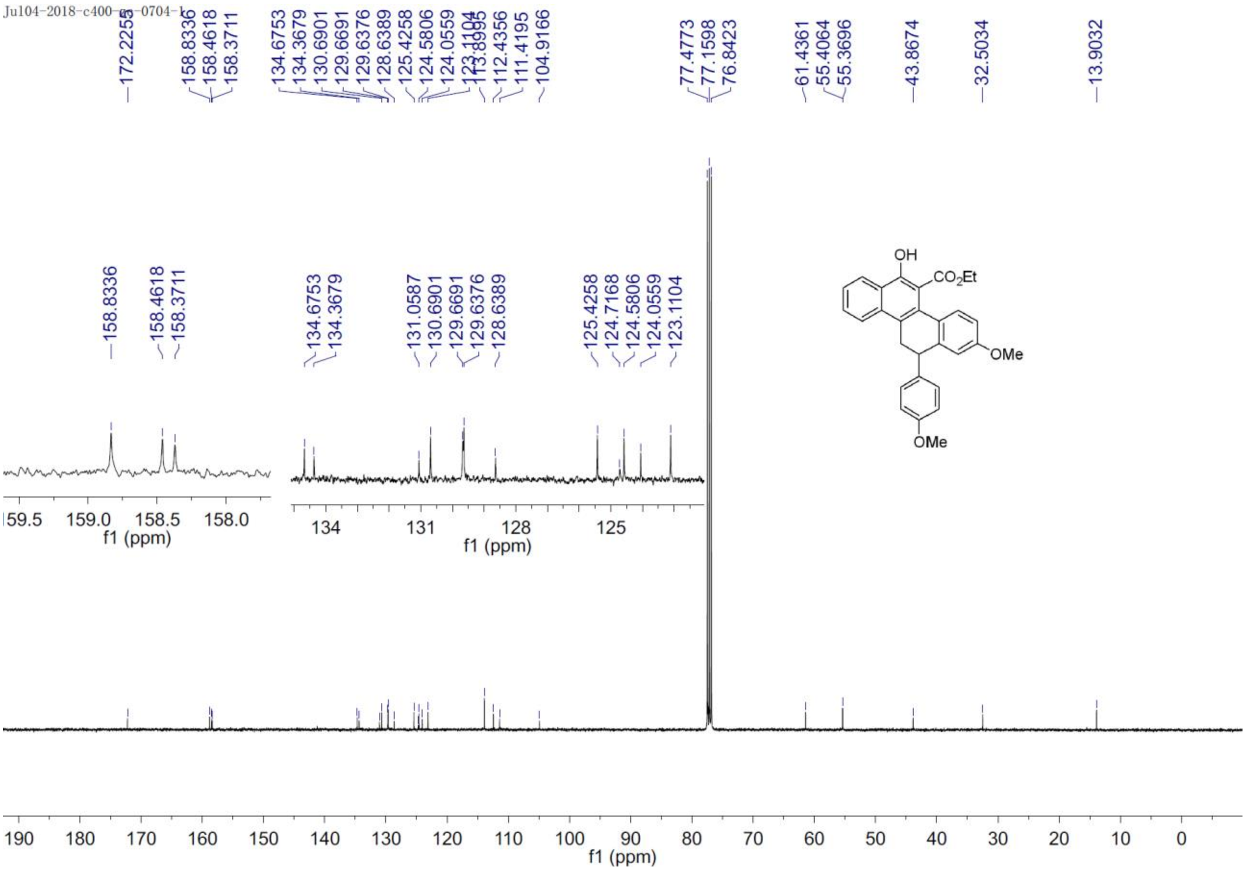

Supplementary Figure 74. ^{13}**C NMR (100 MHz, CDCl**$_3$**) spectrum for compound 30.**

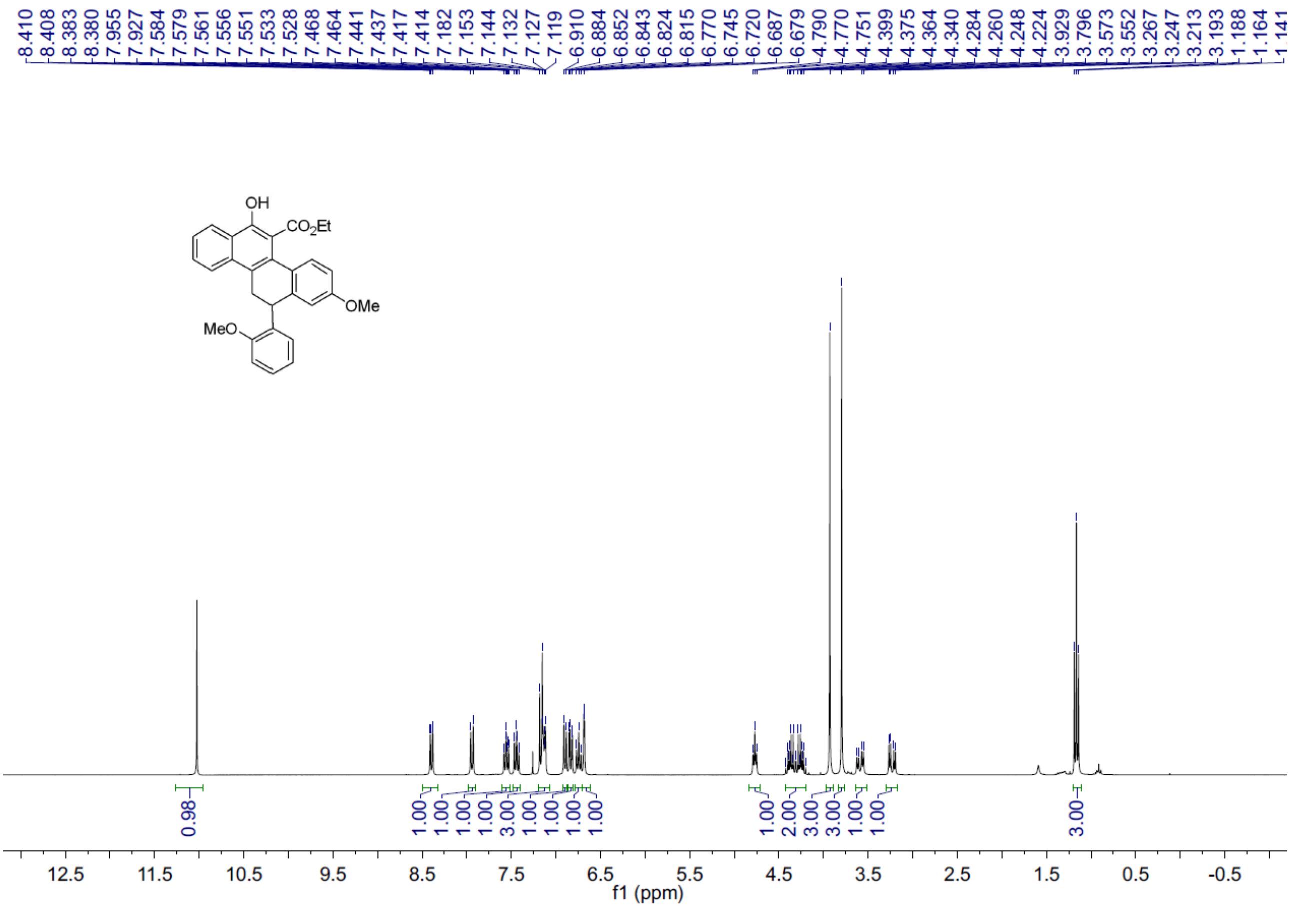

Supplementary Figure 75. ^{1}H NMR (300 MHz, $CDCl_3$) spectrum for compound 31.

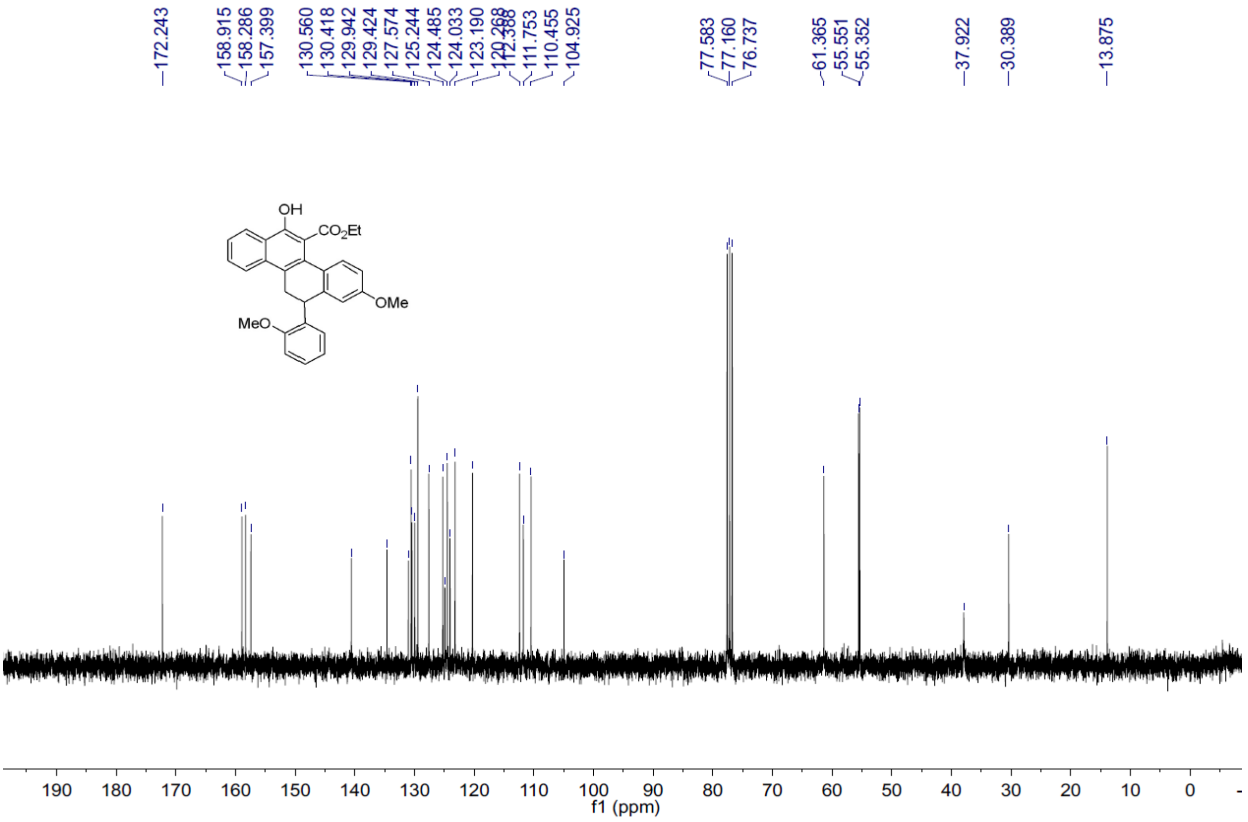

Supplementary Figure 76. ^{13}C NMR (75 MHz, $CDCl_3$) spectrum for compound 31.

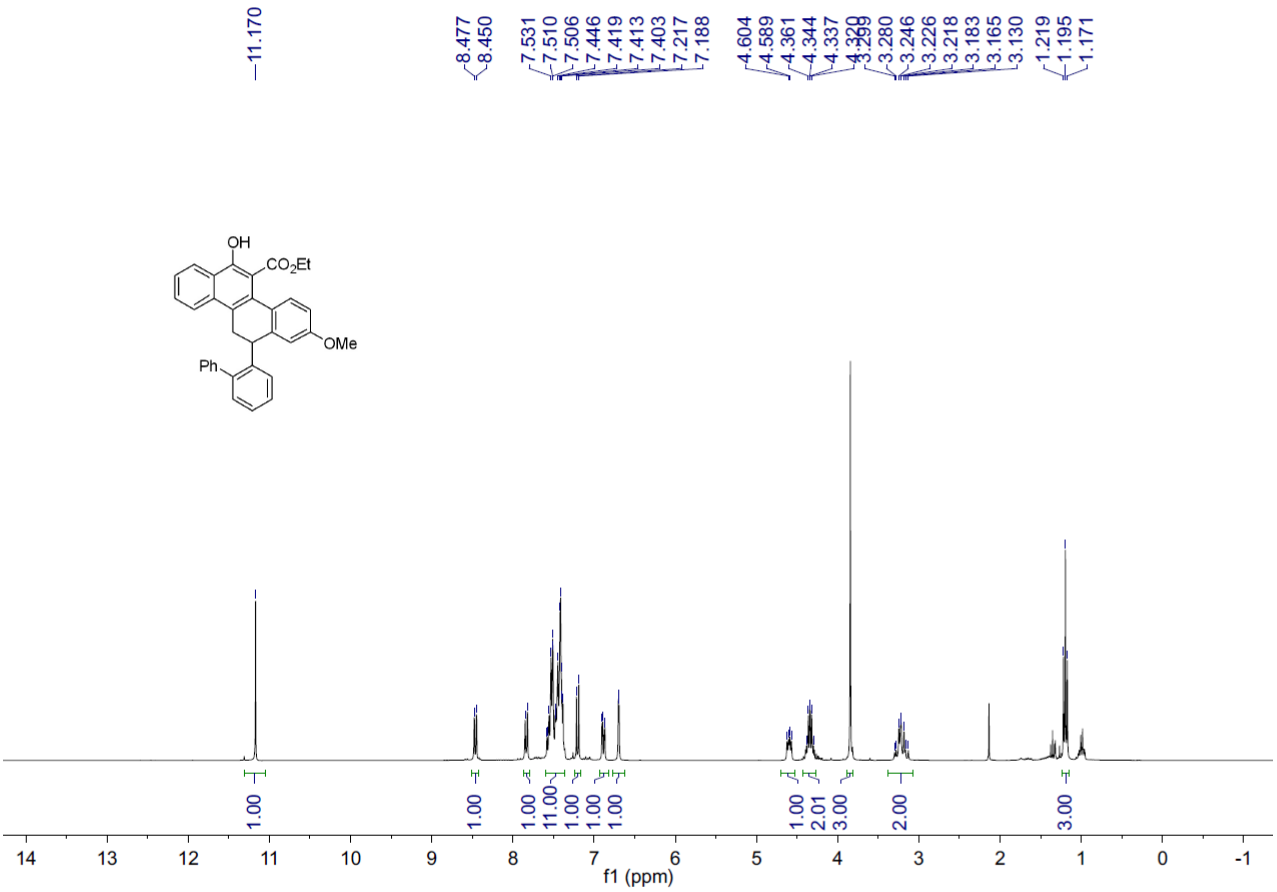

Supplementary Figure 77. ^{1}H NMR (300 MHz, $CDCl_3$) spectrum for compound 32.

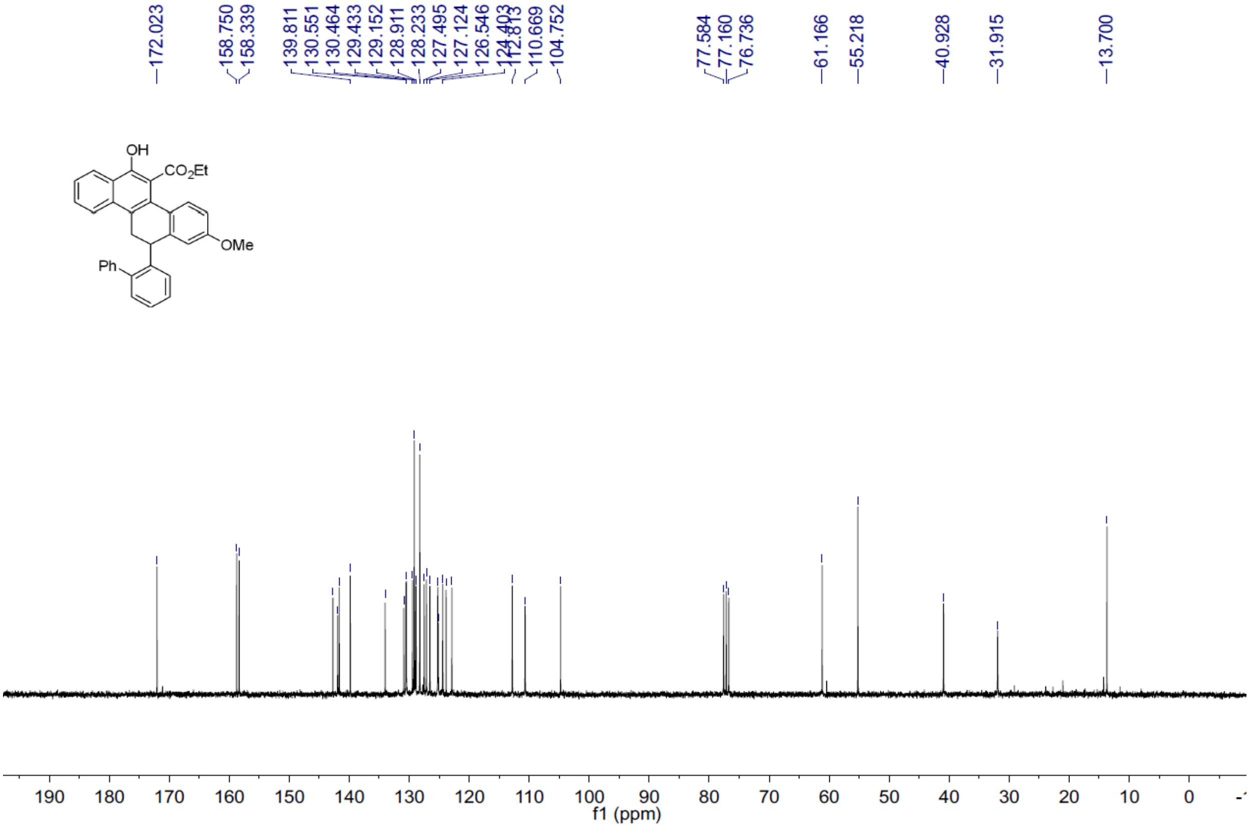

Supplementary Figure 78. ^{13}C NMR (75 MHz, $CDCl_3$) spectrum for compound 32.

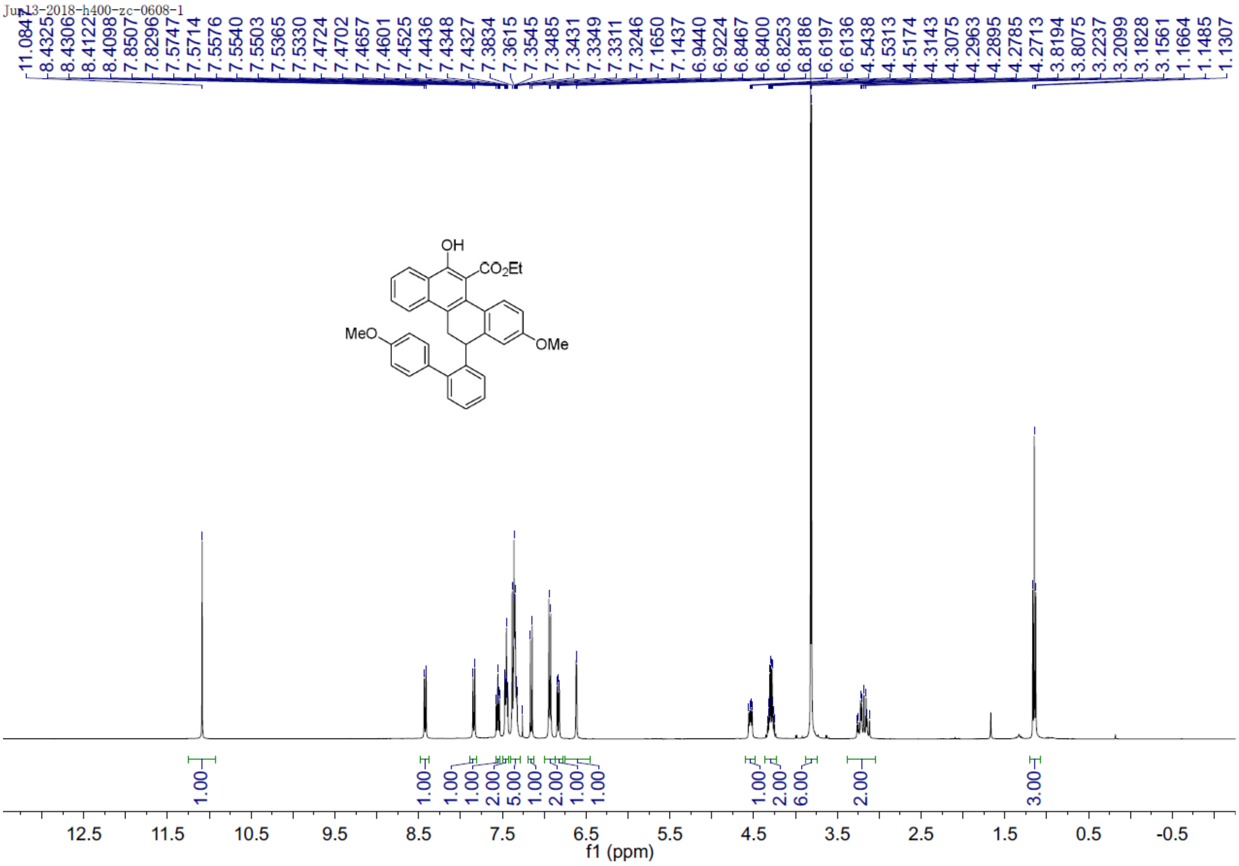

Supplementary Figure 79. ^{1}H NMR (400 MHz, $CDCl_3$) spectrum for compound 33.

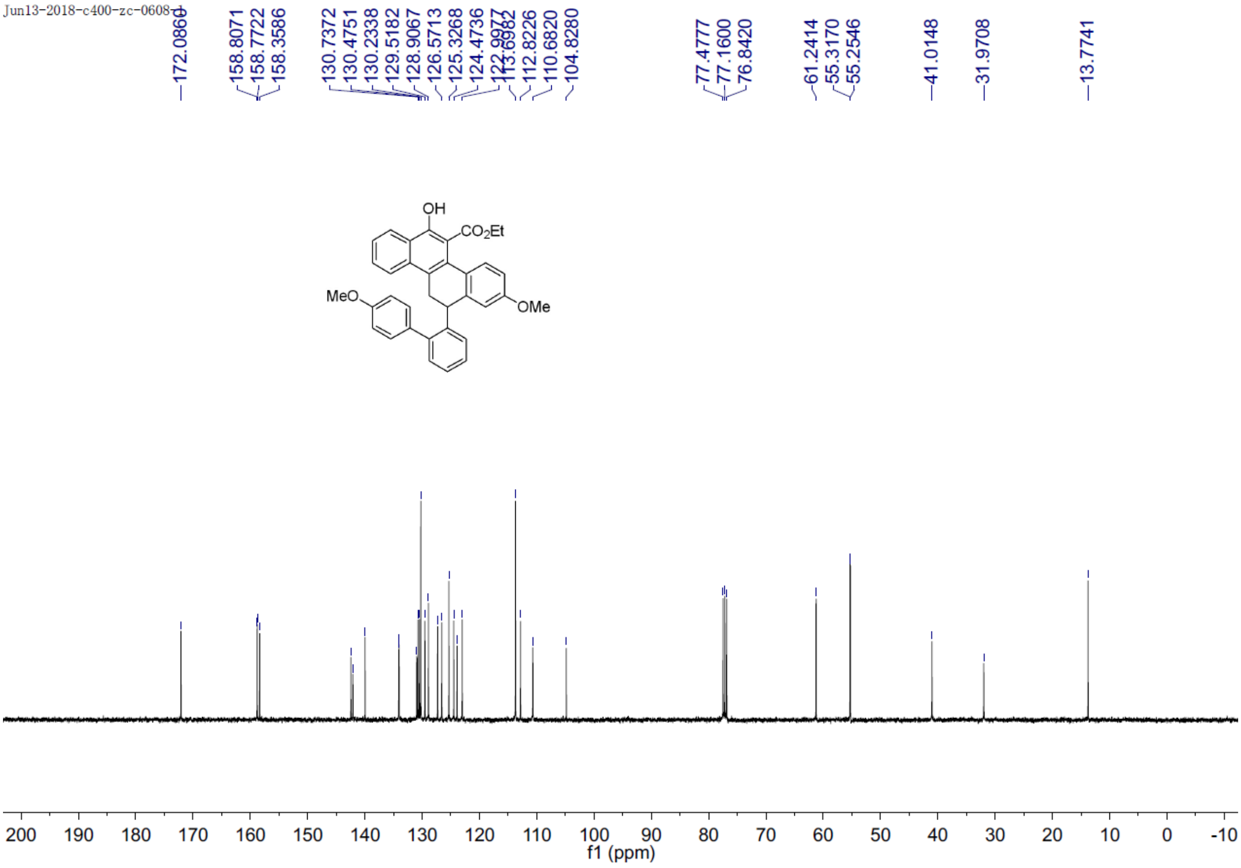

Supplementary Figure 80. ^{13}C NMR (100 MHz, $CDCl_3$) spectrum for compound 33.

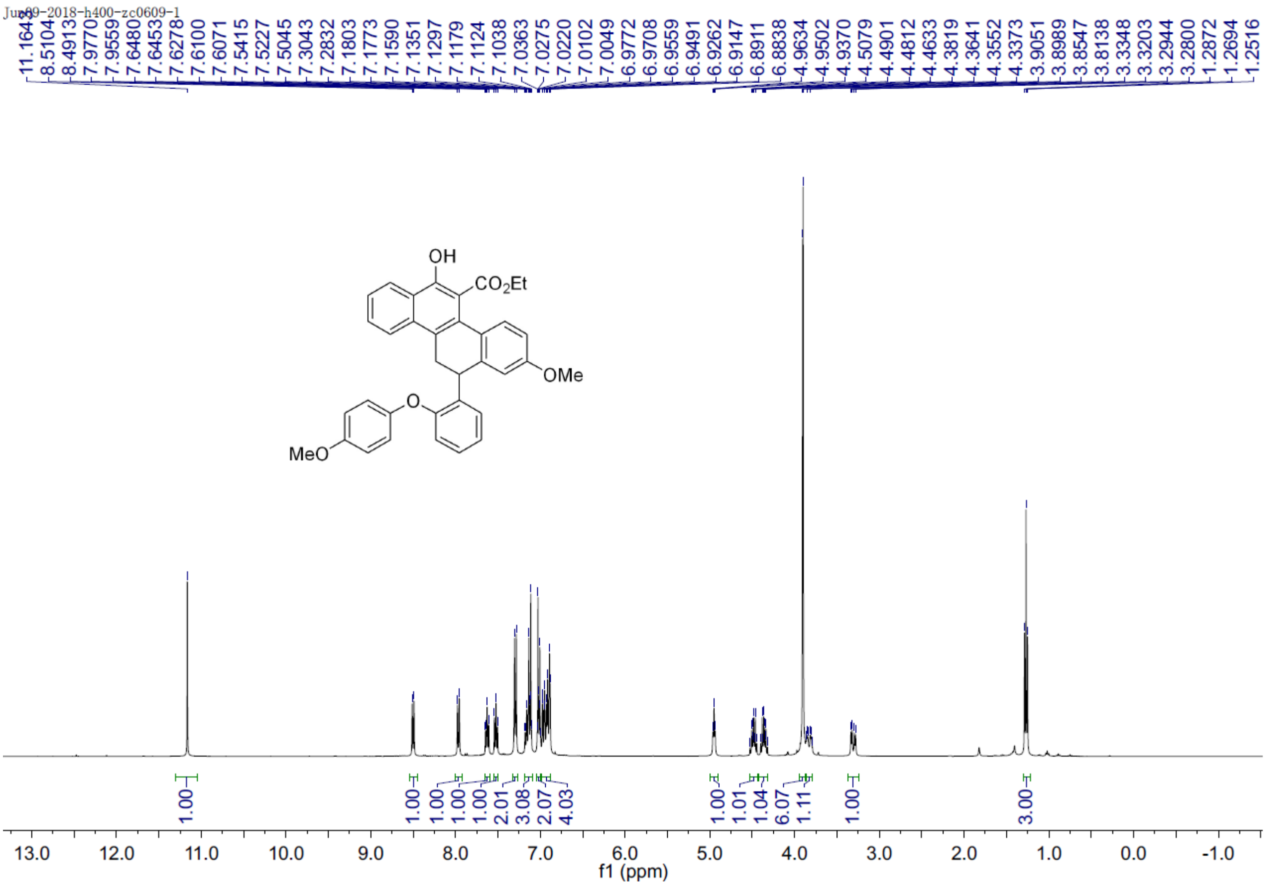

Supplementary Figure 81. ^{1}H **NMR (400 MHz,** $CDCl_3$**) spectrum for compound 34.**

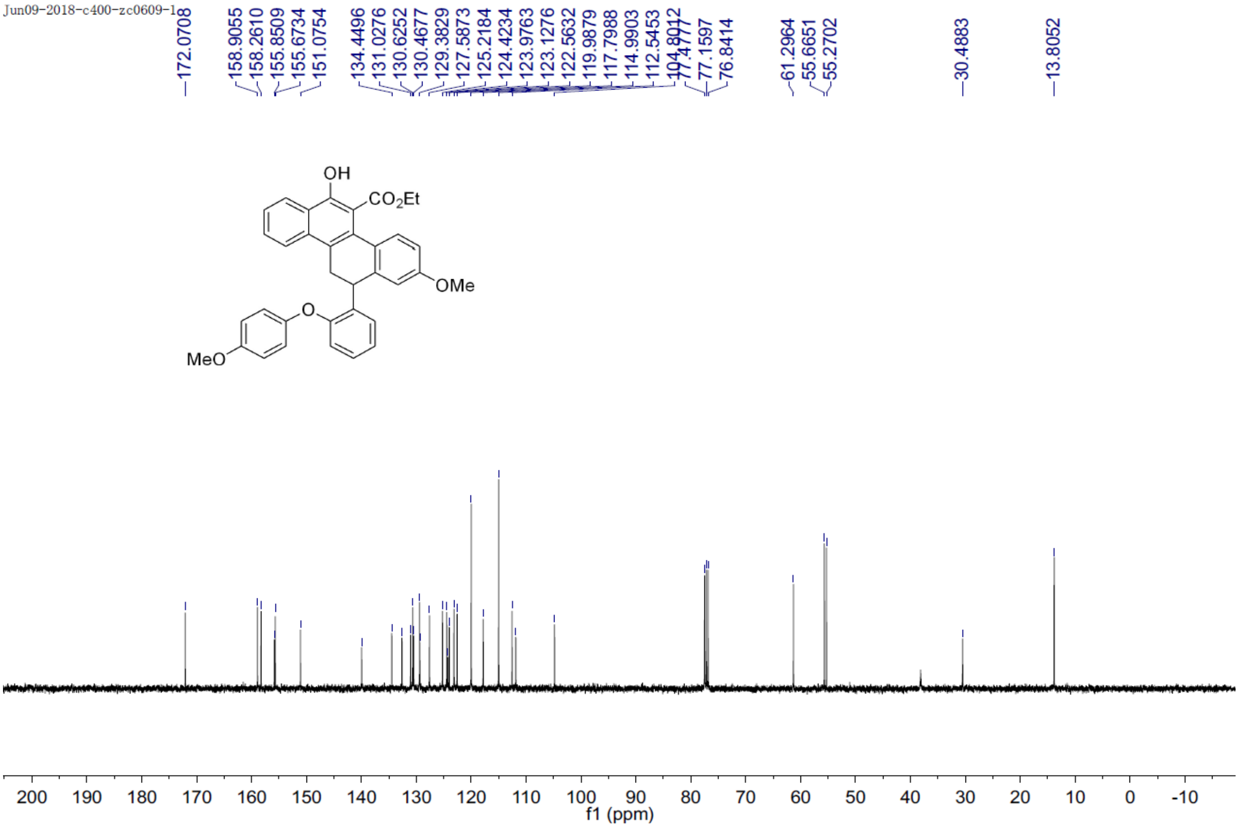

Supplementary Figure 82. ^{13}C **NMR (100 MHz,** $CDCl_3$**) spectrum for compound 34.**

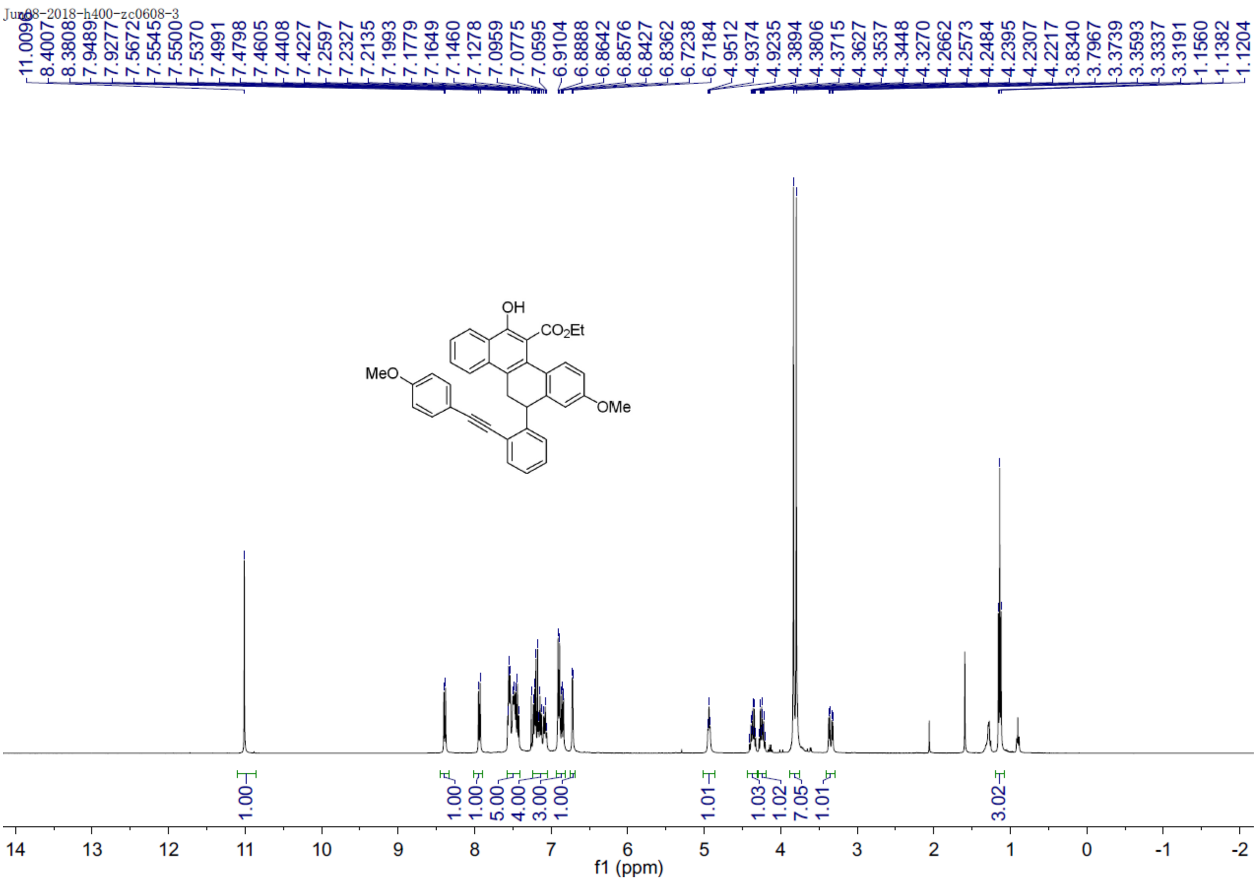

Supplementary Figure 83. ^{1}H NMR (400 MHz, $CDCl_3$) spectrum for compound 35.

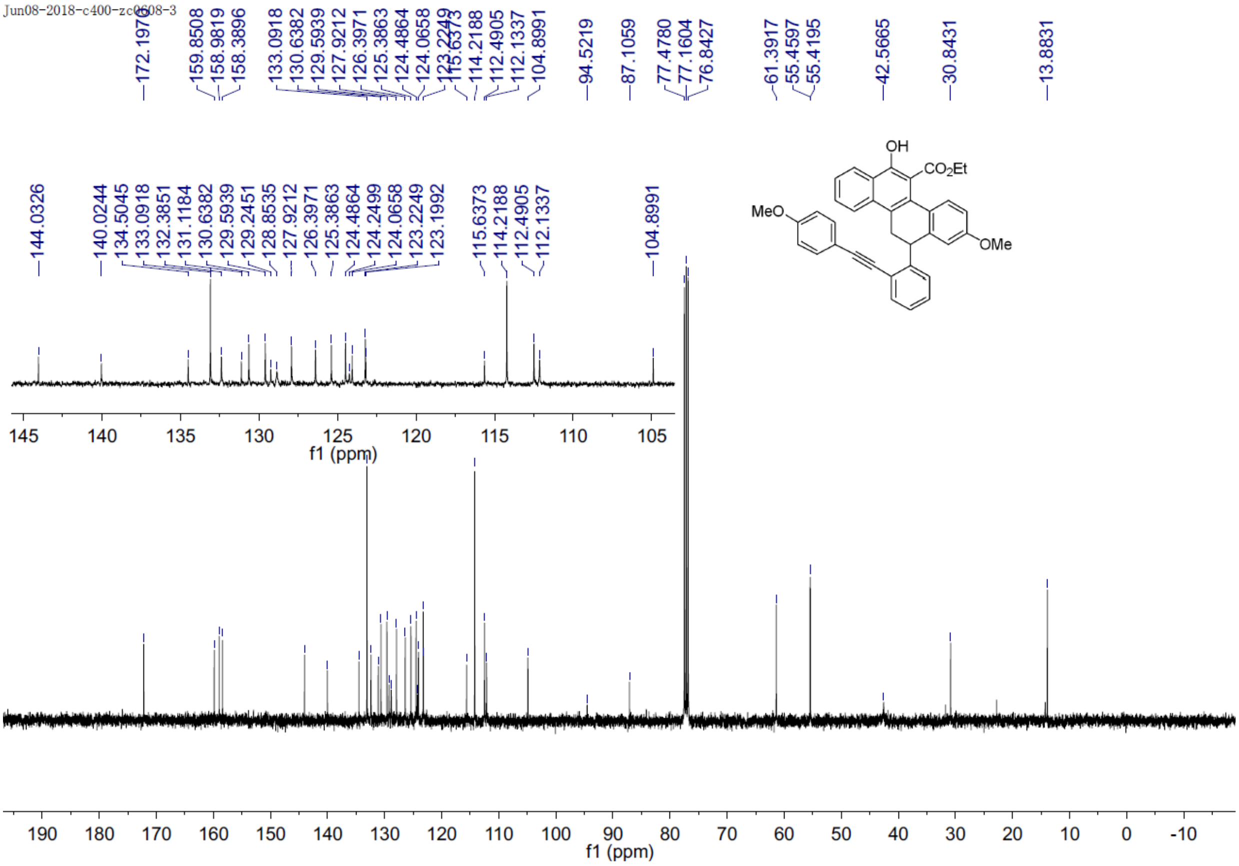

Supplementary Figure 84. ^{13}C NMR (100 MHz, $CDCl_3$) spectrum for compound 35.

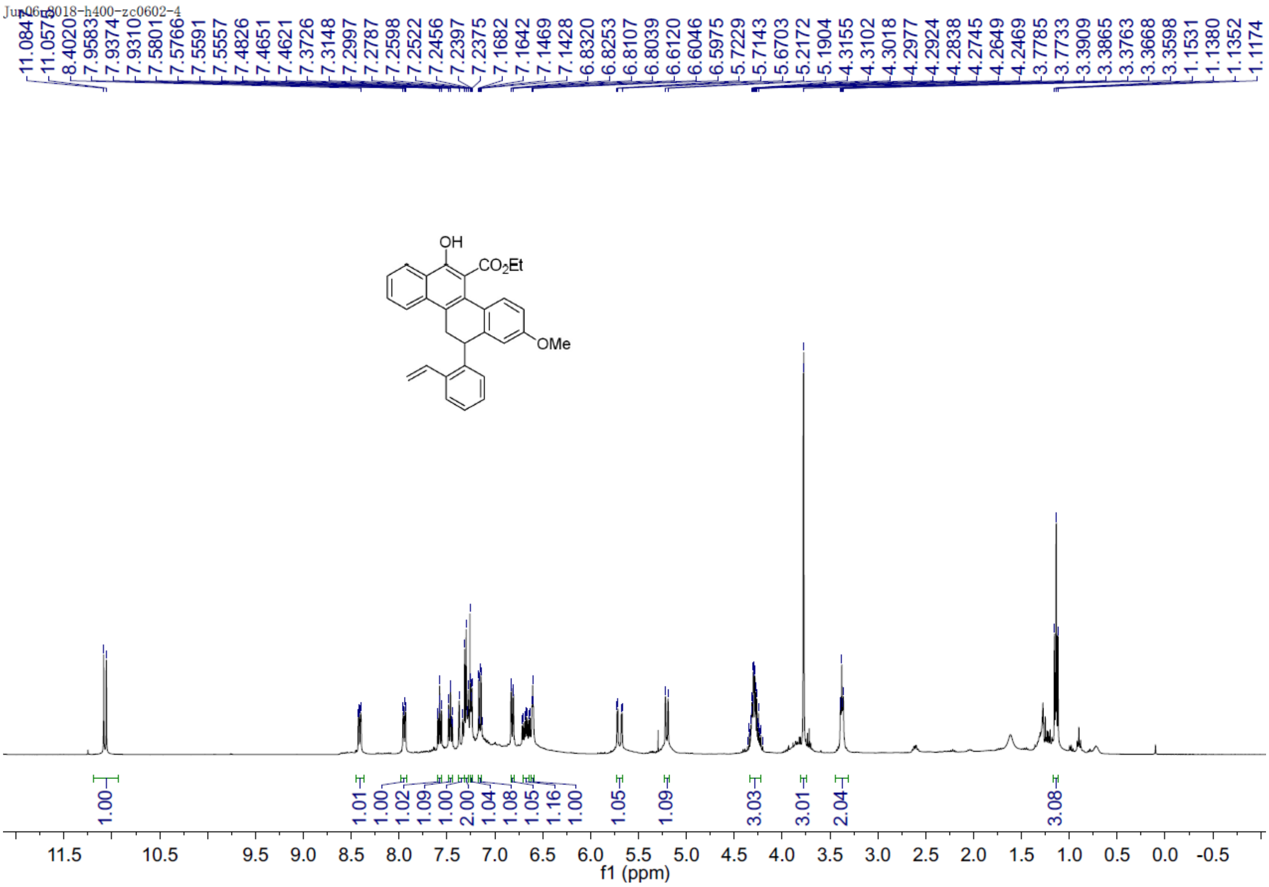

Supplementary Figure 85. ^{1}H **NMR (400 MHz,** $CDCl_3$**) spectrum for compound 36.**

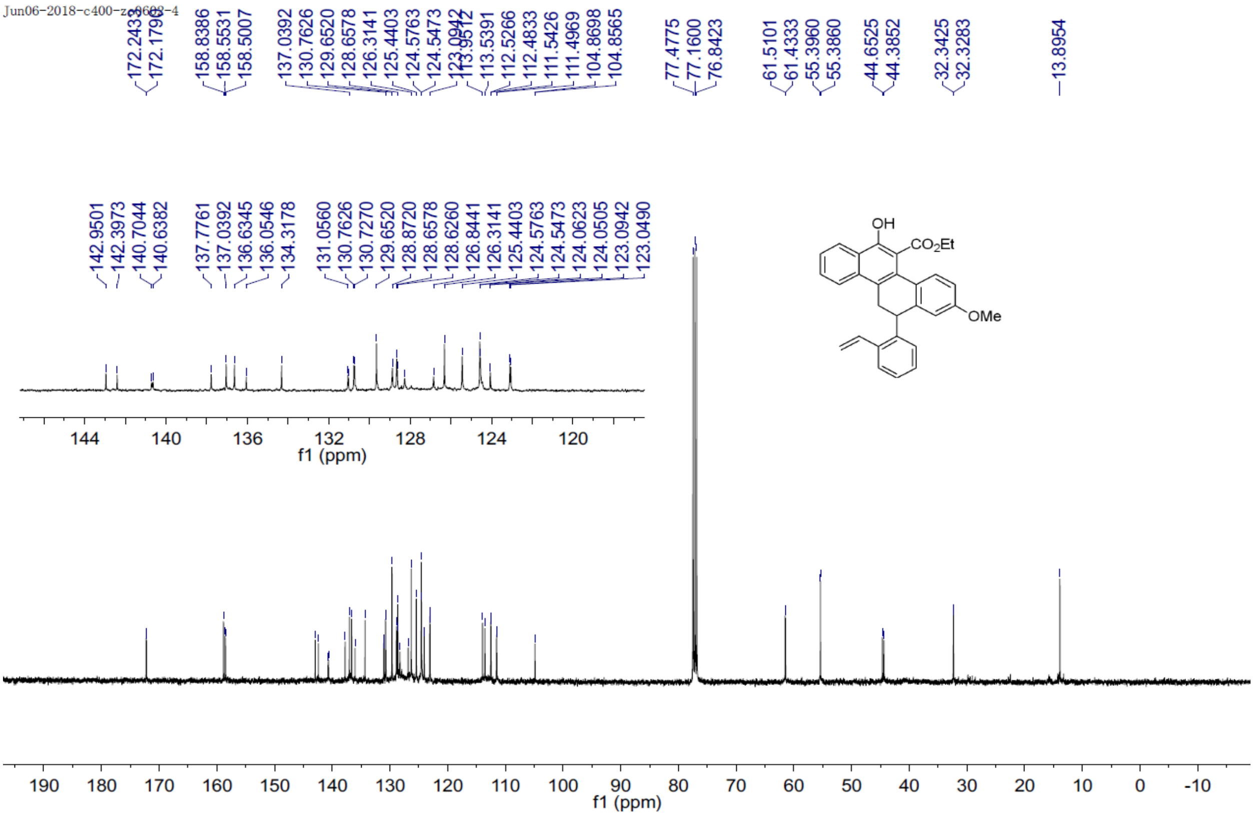

Supplementary Figure 86. ^{13}C **NMR (100 MHz,** $CDCl_3$**) spectrum for compound 36.**

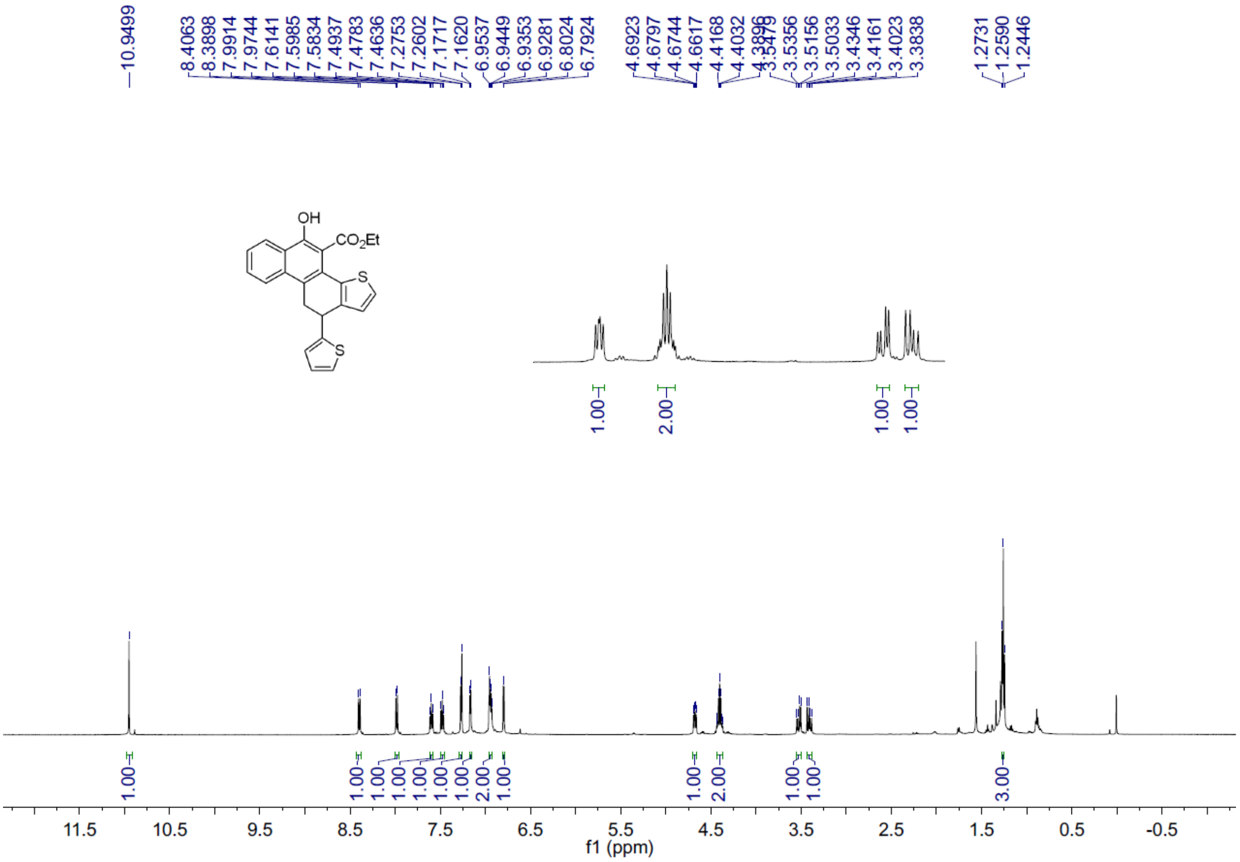

Supplementary Figure 87. ^{1}H NMR (500 MHz, $CDCl_3$) spectrum for compound 37.

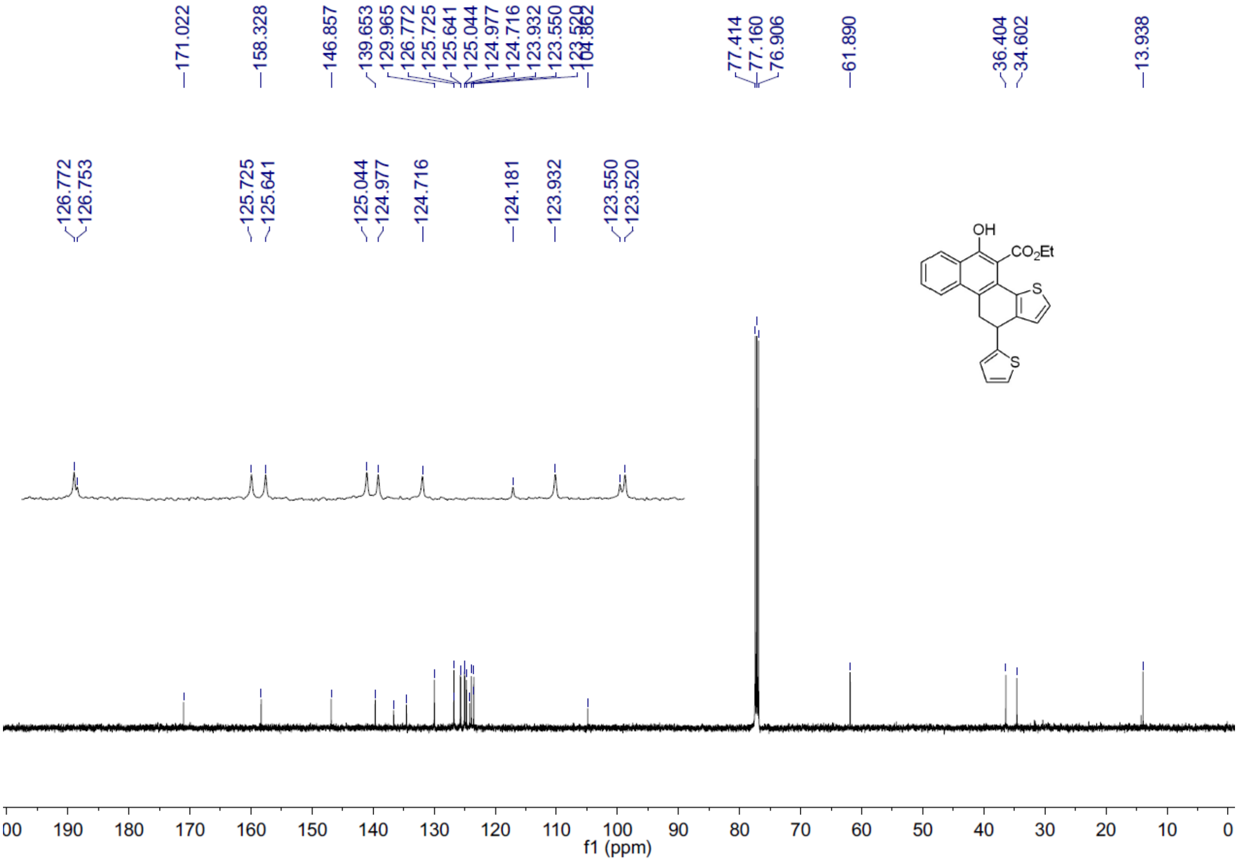

Supplementary Figure 88. ^{13}C NMR (125 MHz, $CDCl_3$) spectrum for compound 37.

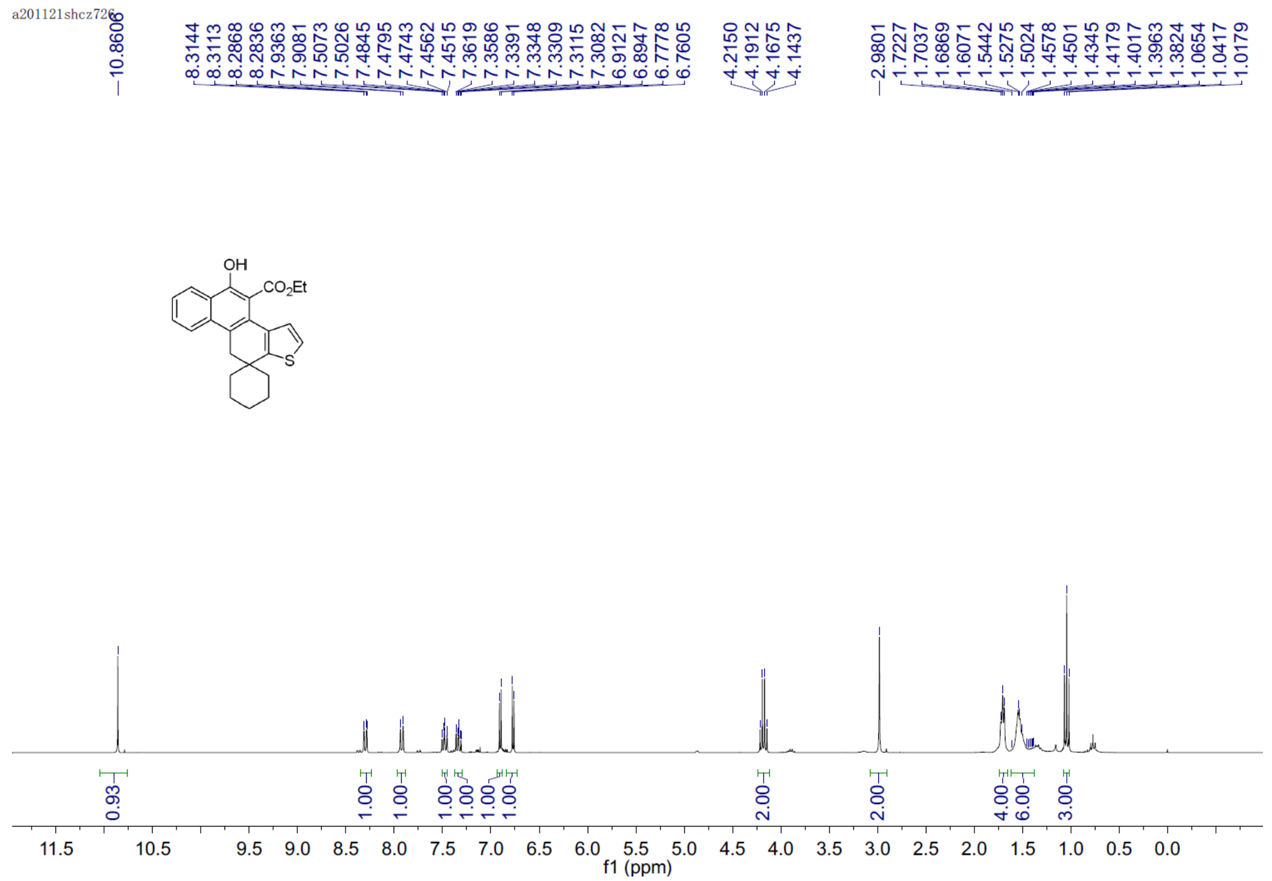

Supplementary Figure 89. ^{1}H NMR (300 MHz, $CDCl_3$) spectrum for compound 38.

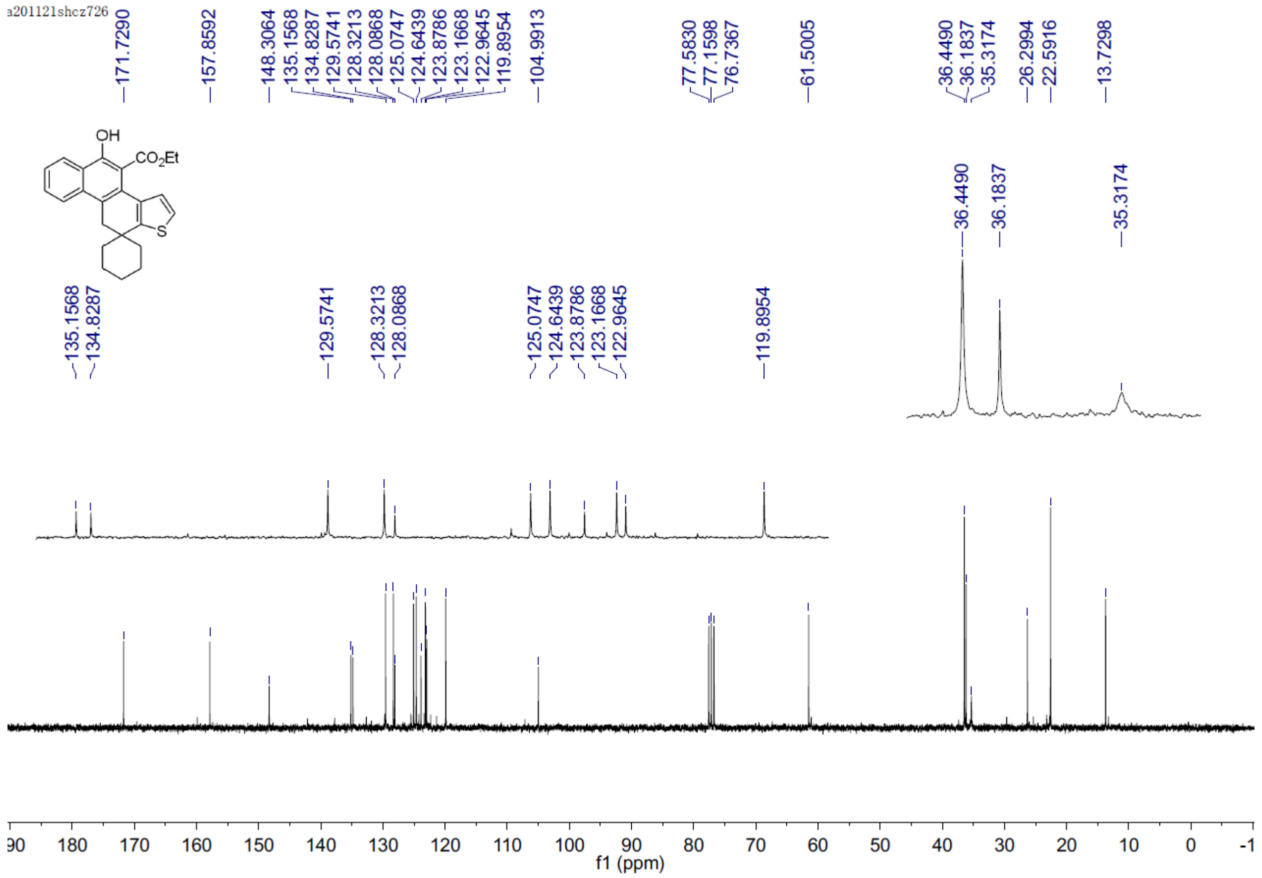

Supplementary Figure 90. ^{13}C NMR (75 MHz, $CDCl_3$) spectrum for compound 38.

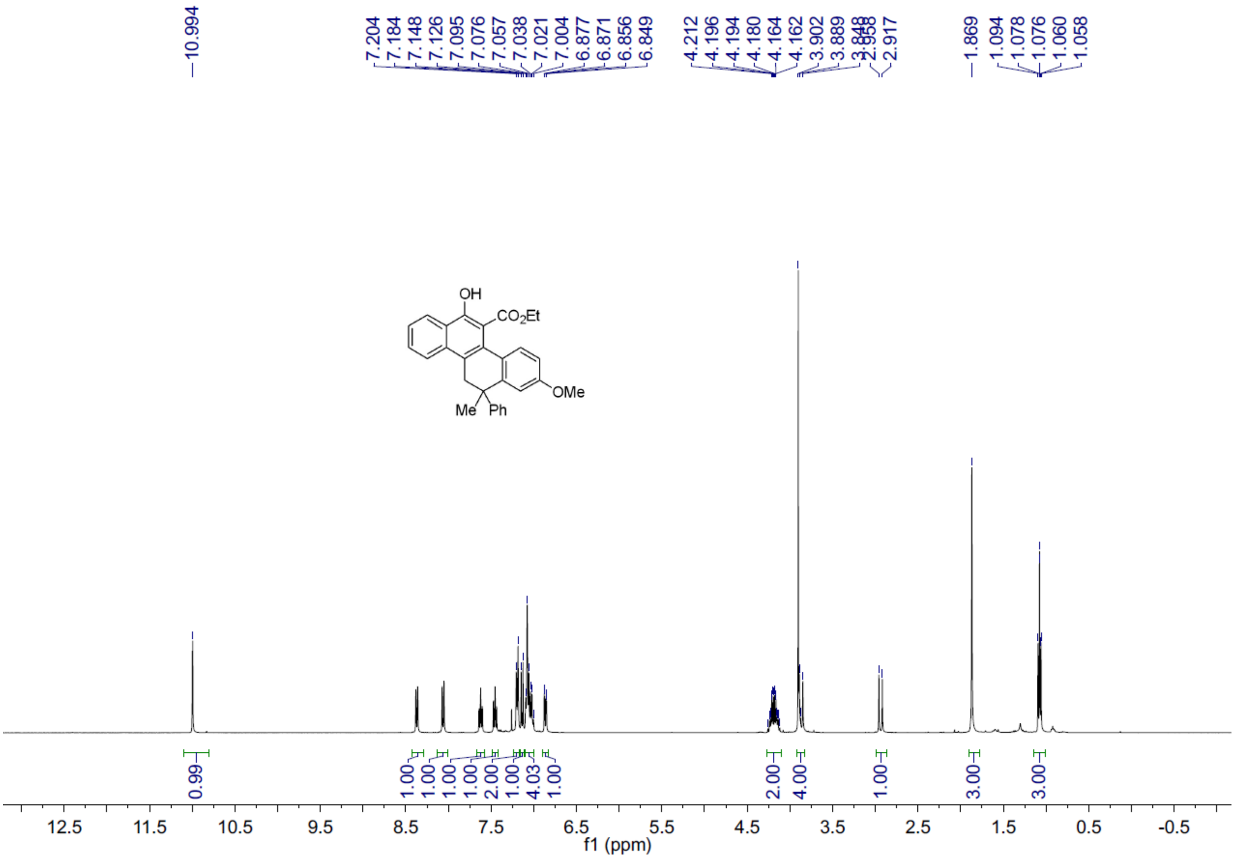

Supplementary Figure 91. ^{1}H NMR (400 MHz, $CDCl_3$) spectrum for compound 39.

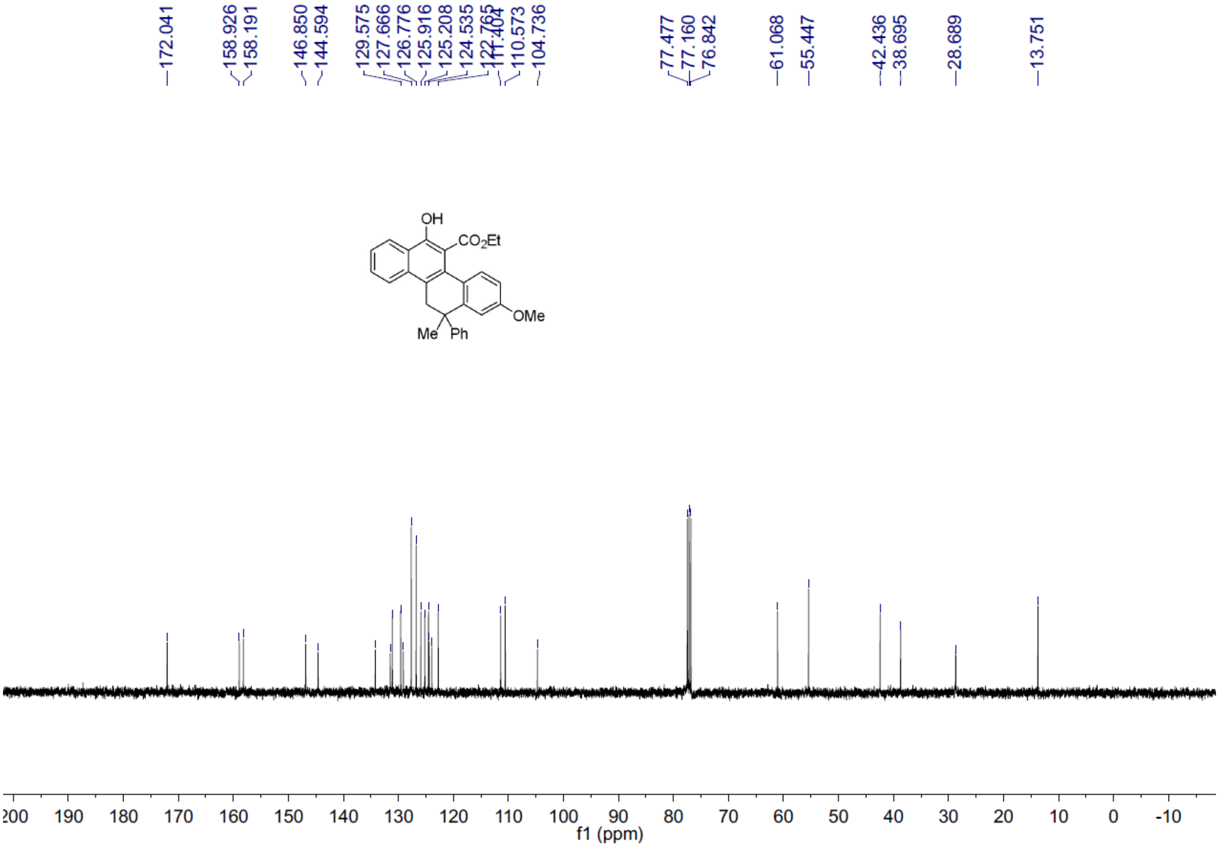

Supplementary Figure 92. ^{13}C NMR (100 MHz, $CDCl_3$) spectrum for compound 39.

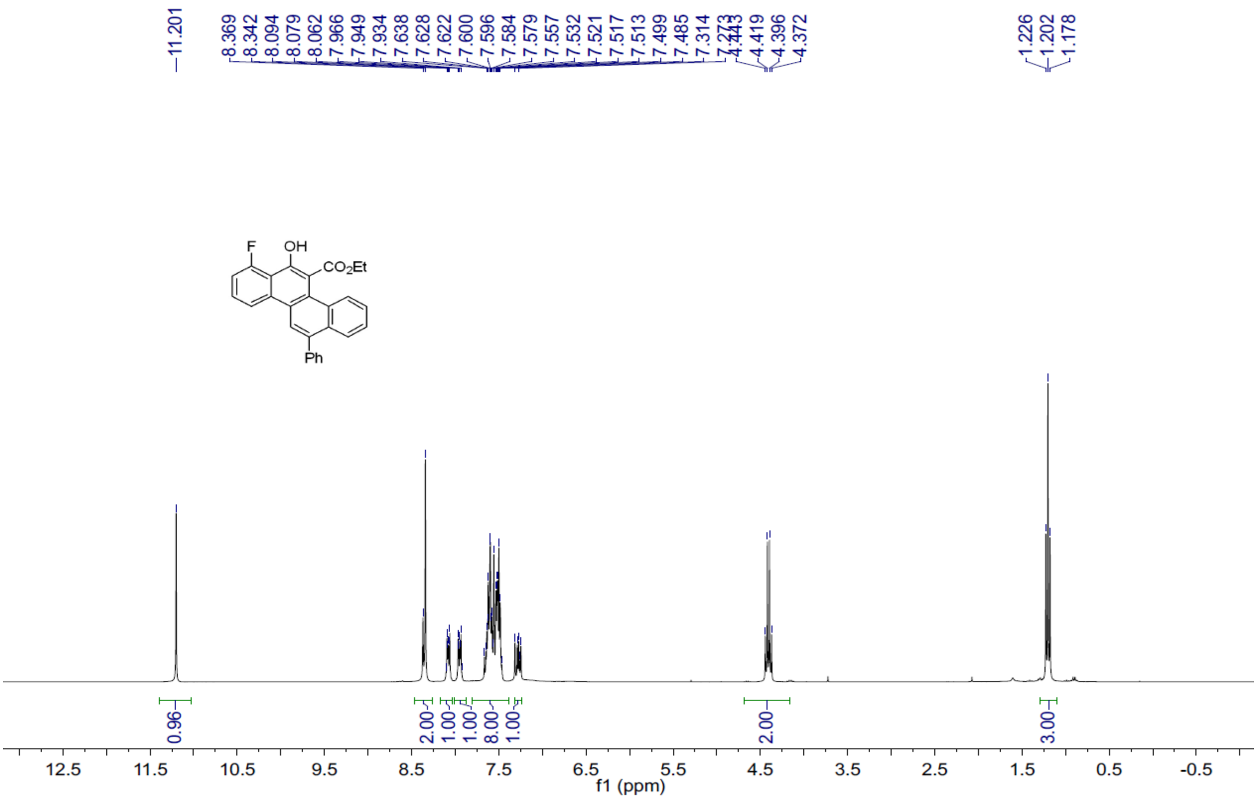

Supplementary Figure 93. ^{1}H NMR (300 MHz, $CDCl_3$) spectrum for compound 40.

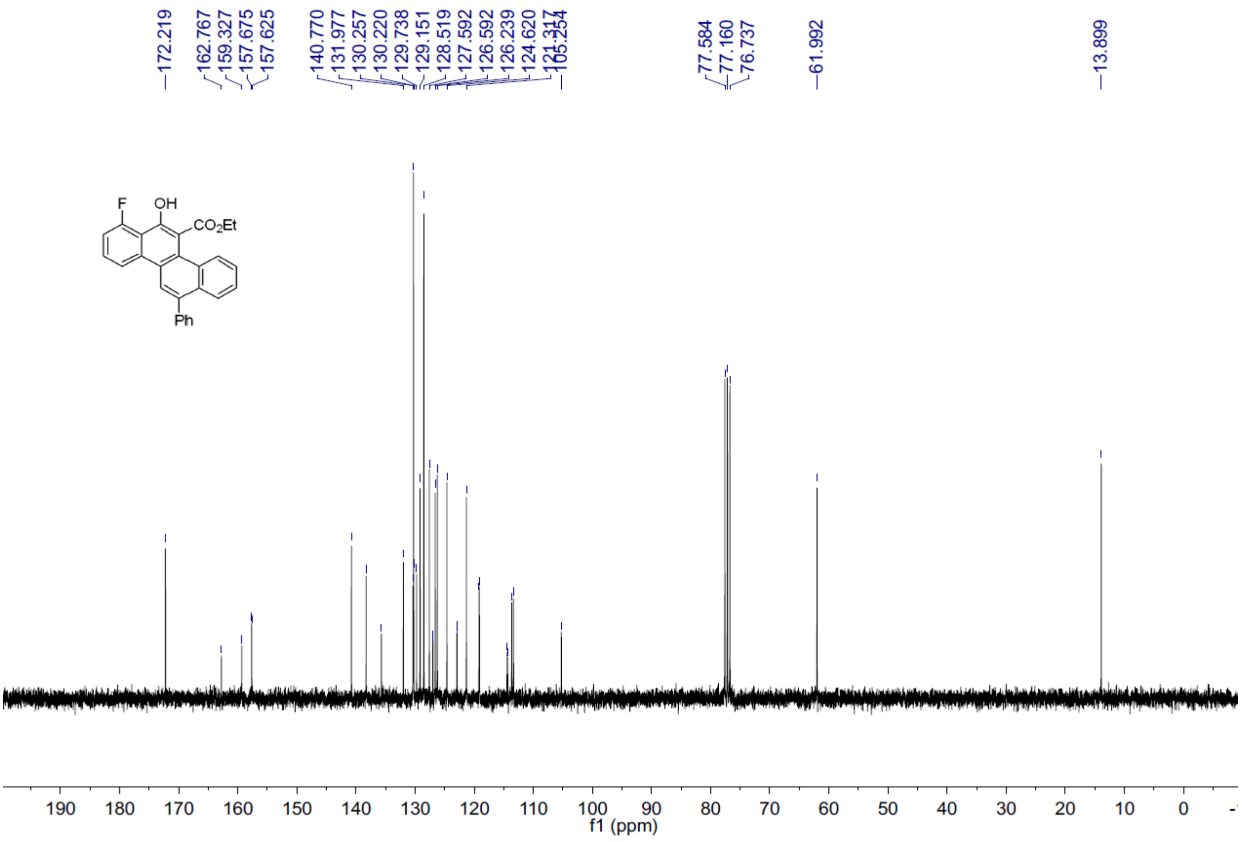

Supplementary Figure 94. ^{13}C NMR (75 MHz, $CDCl_3$) spectrum for compound 40.

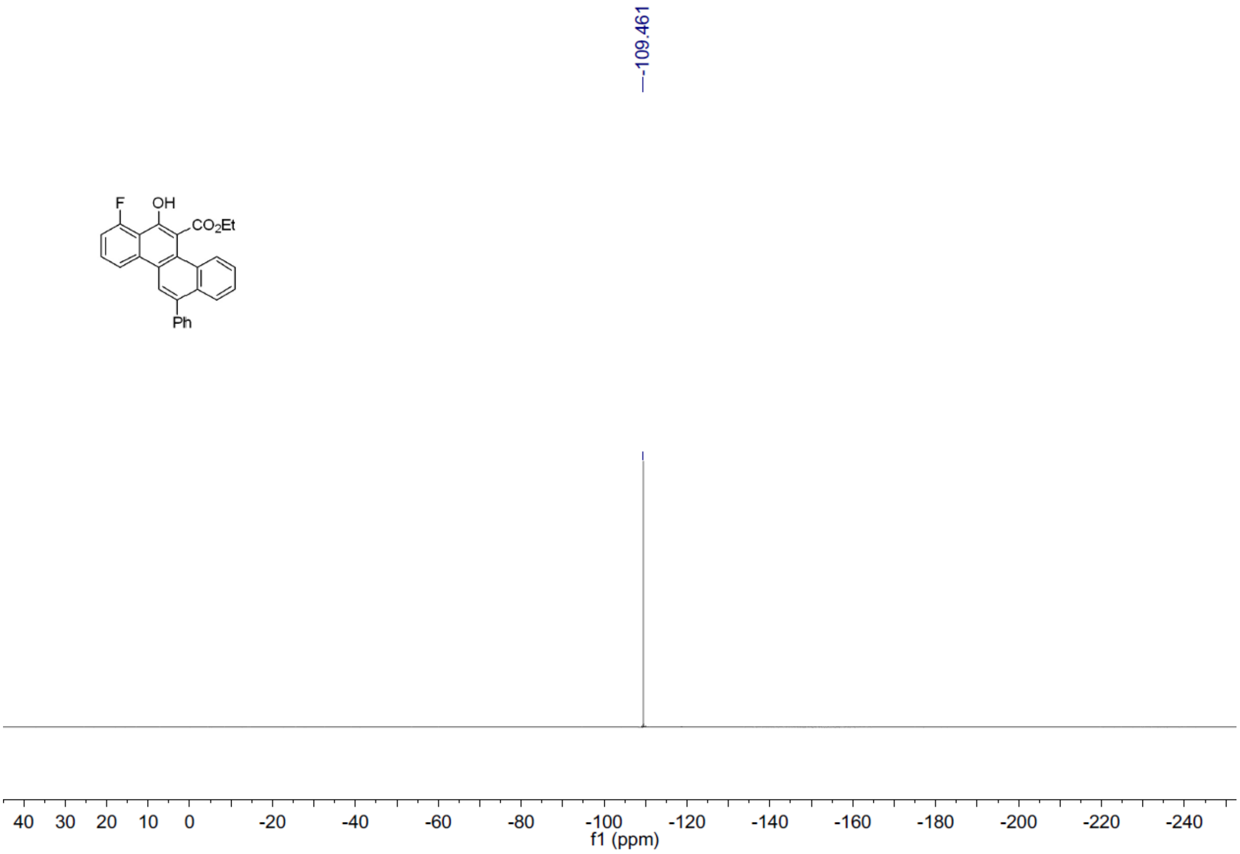

Supplementary Figure 95. ^{19}F NMR (283 MHz, $CDCl_3$) spectrum for compound 40.

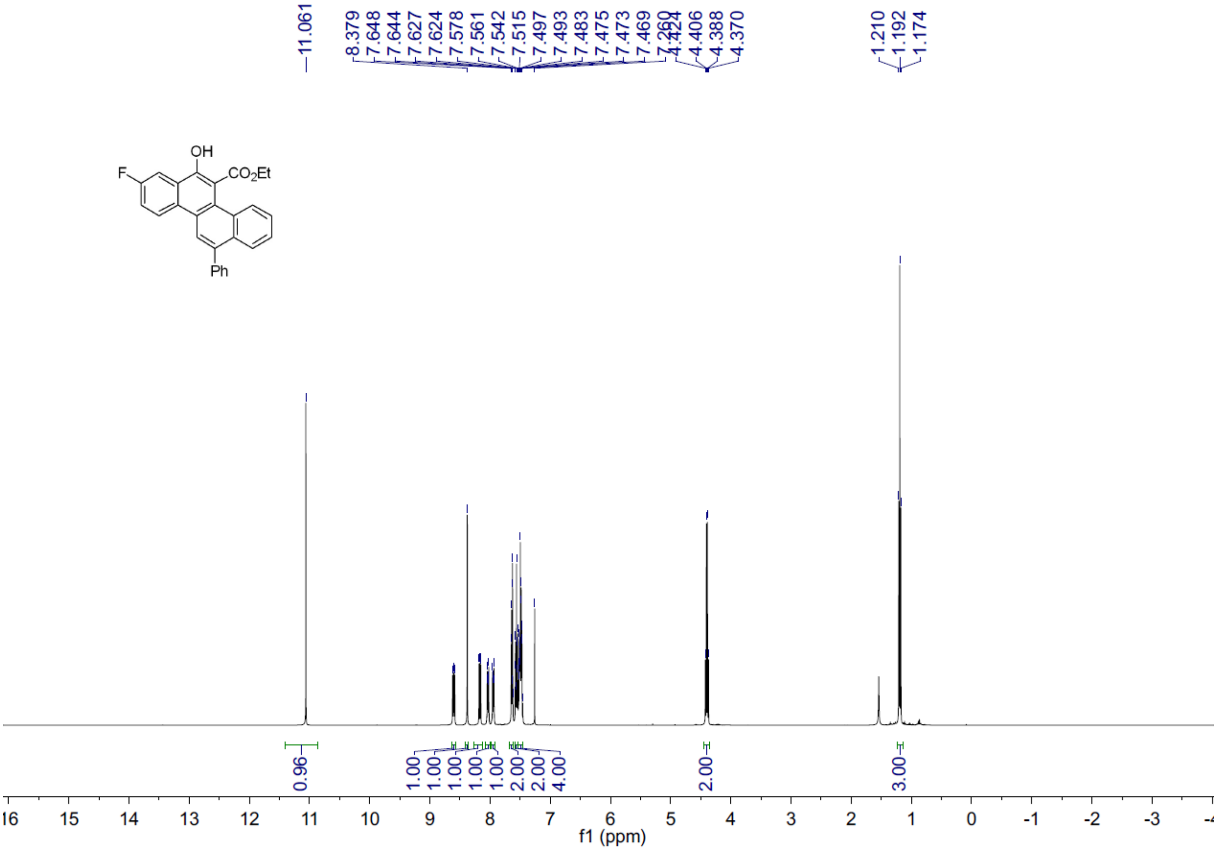

Supplementary Figure 96. ^{1}H NMR (400 MHz, $CDCl_3$) spectrum for compound 41.

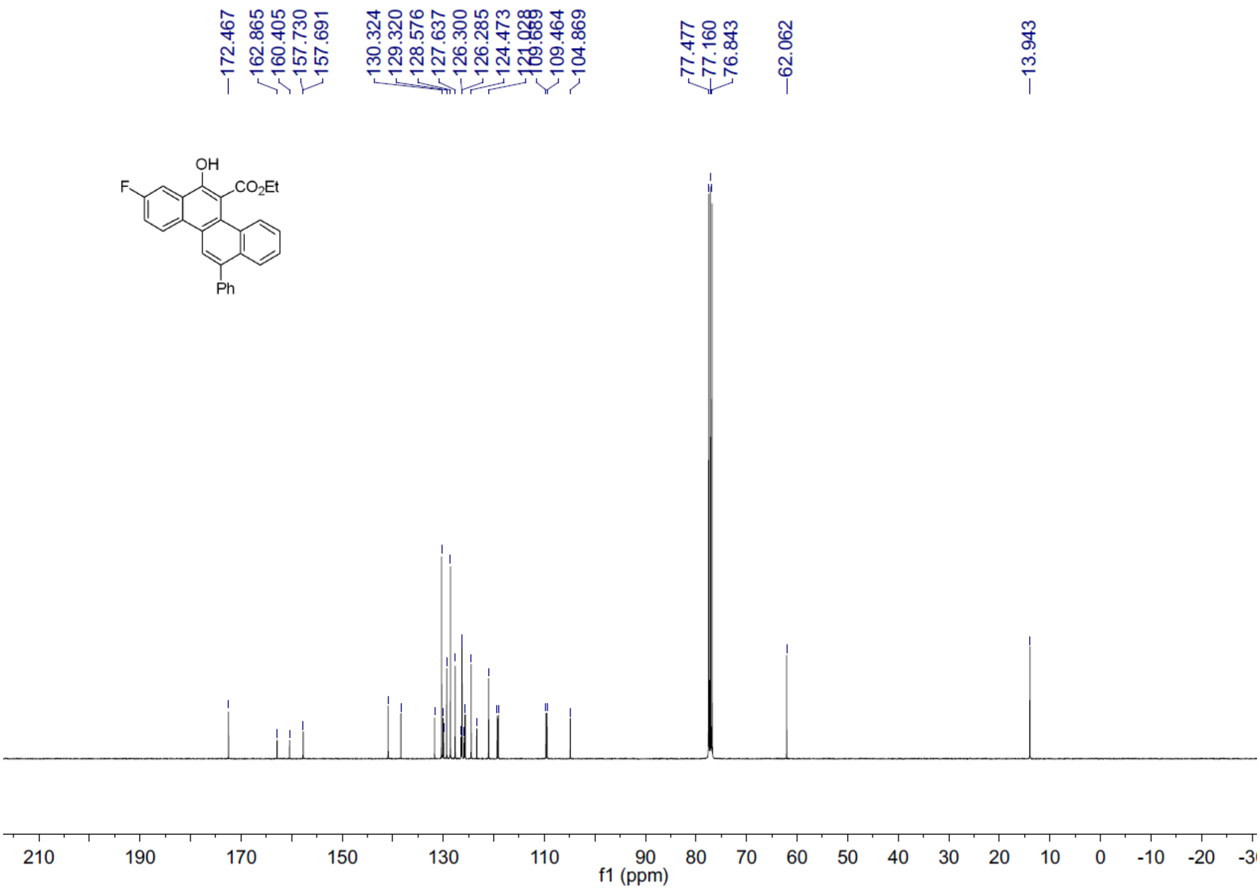

Supplementary Figure 97. ^{13}**C NMR (100 MHz, $CDCl_3$) spectrum for compound 41.**

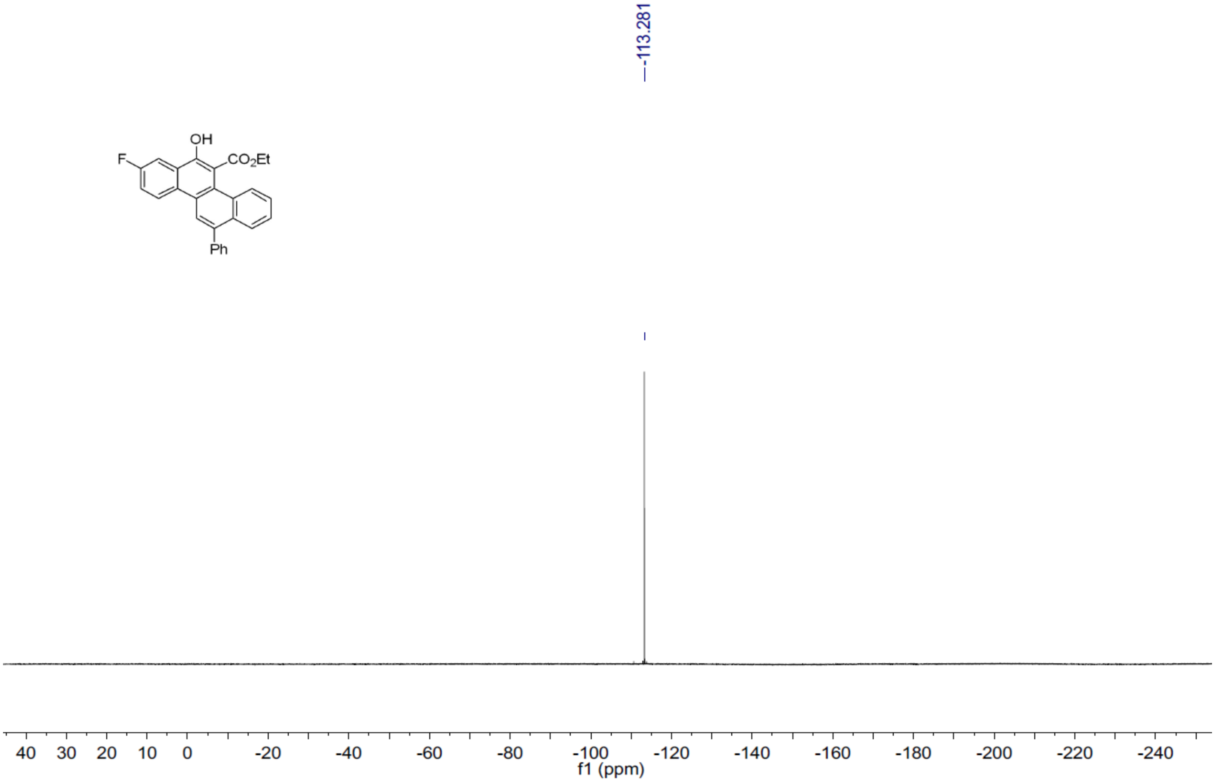

Supplementary Figure 98. ^{19}**F NMR (283 MHz, $CDCl_3$) spectrum for compound 41.**

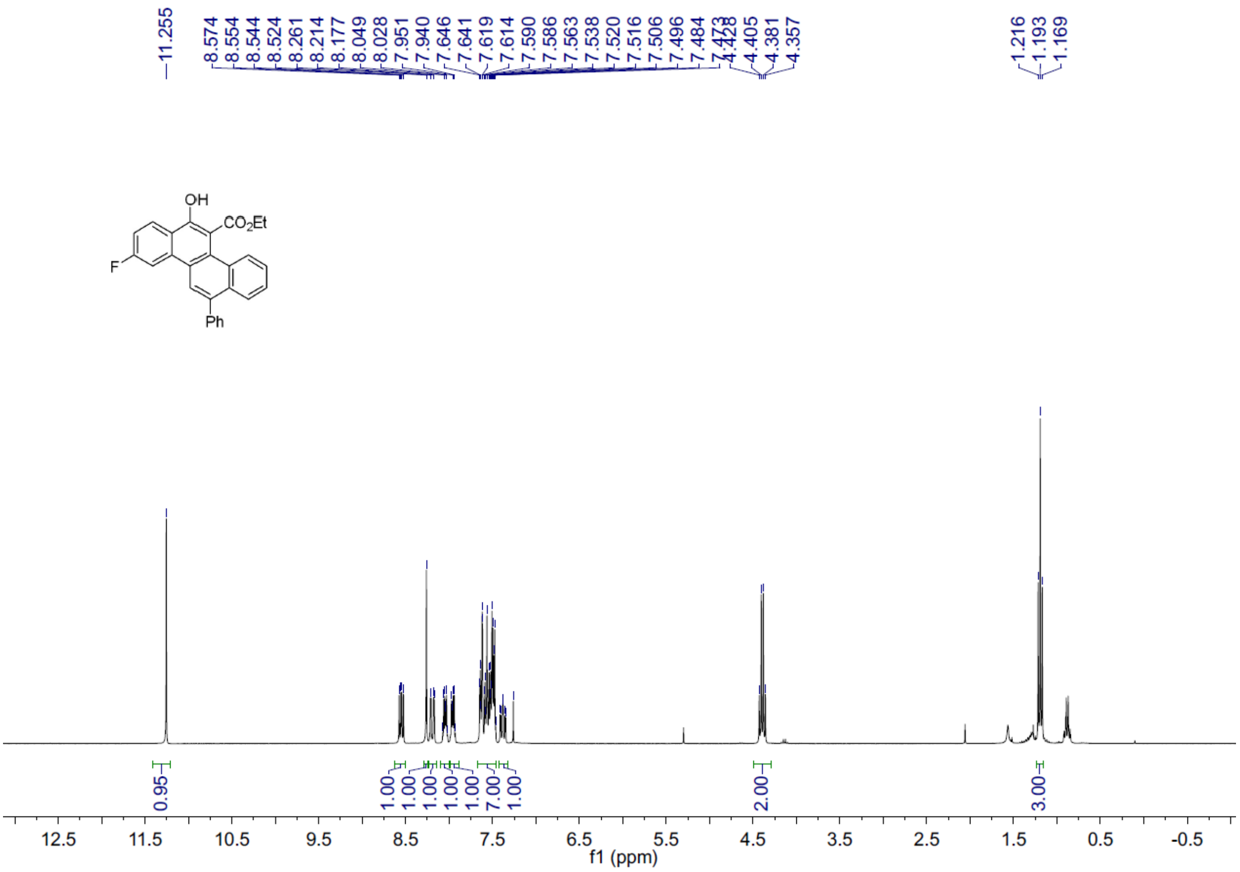

Supplementary Figure 99. ^{1}H NMR (300 MHz, $CDCl_3$) spectrum for compound 42.

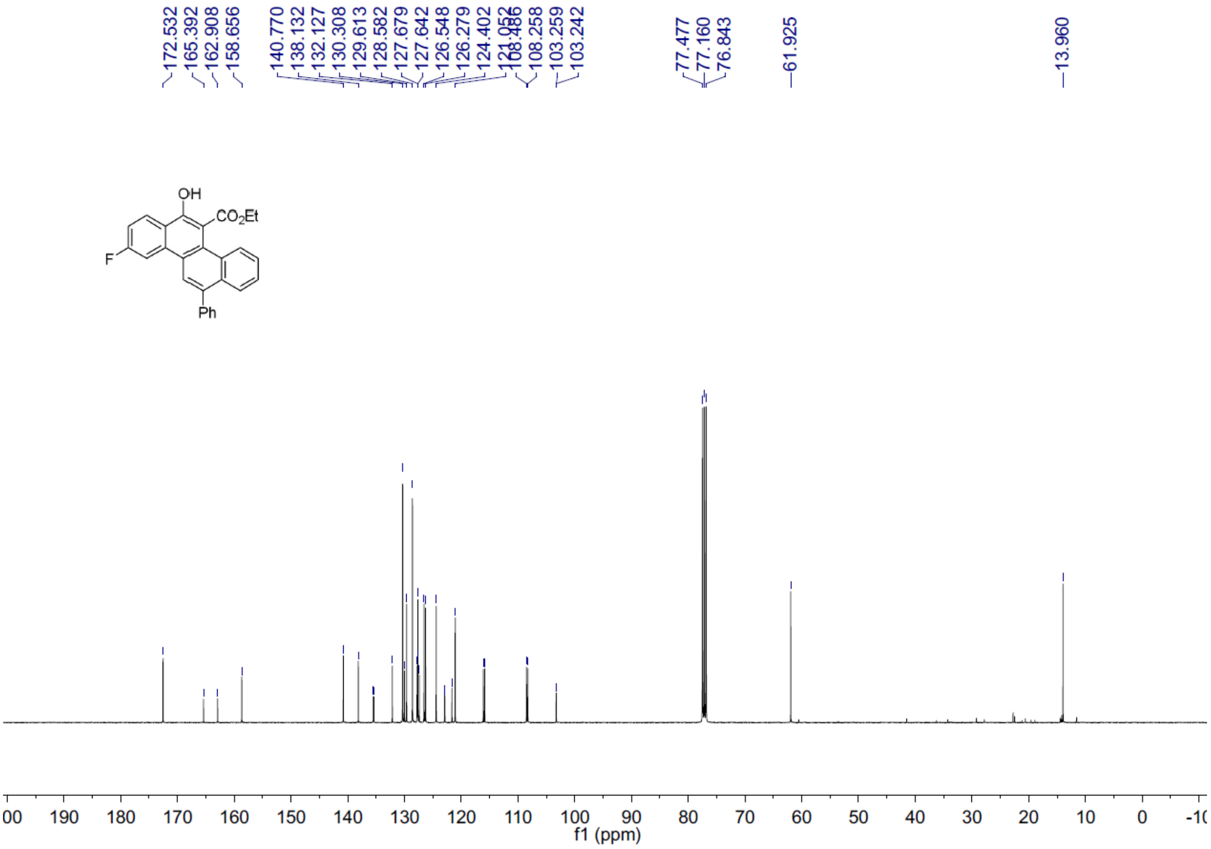

Supplementary Figure 100. ^{13}C NMR (100 MHz, $CDCl_3$) spectrum for compound 42.

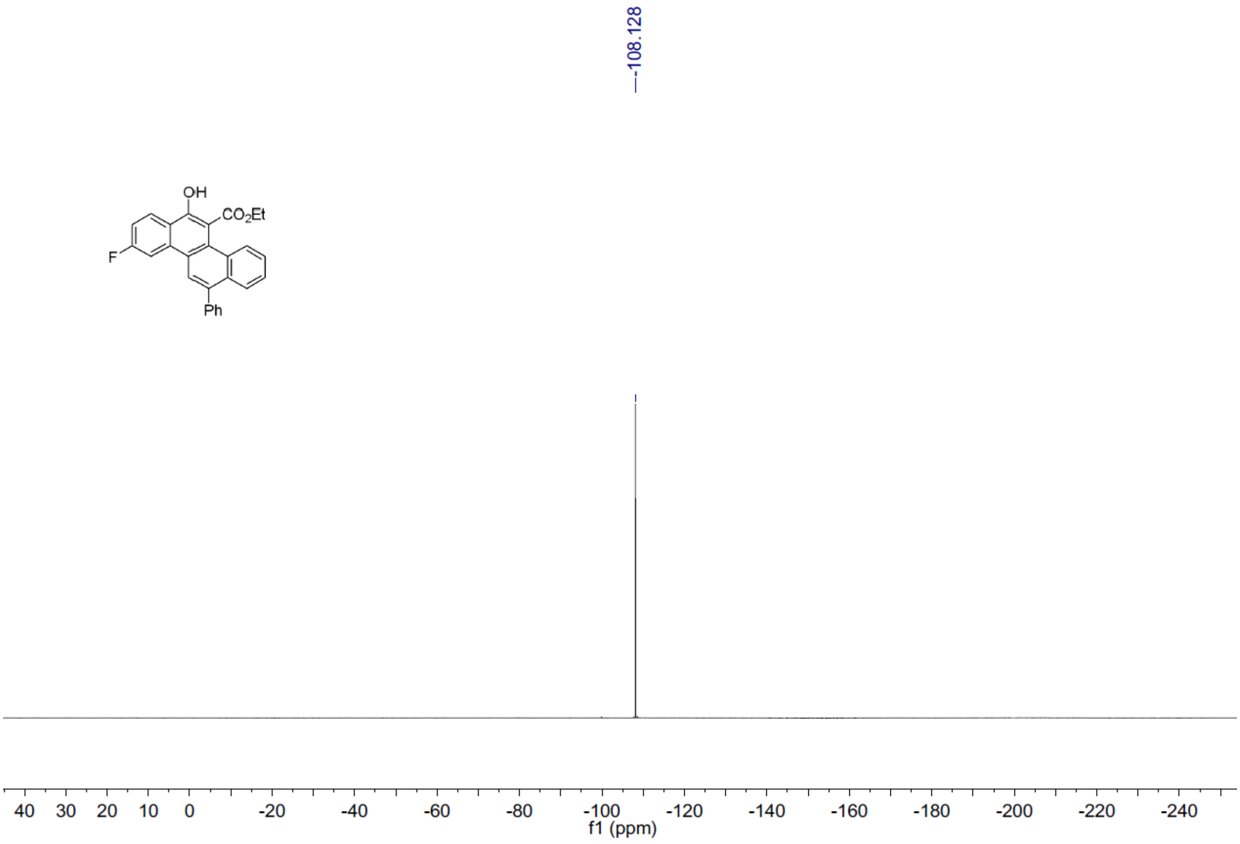

Supplementary Figure 101. ^{19}F NMR (283 MHz, $CDCl_3$) spectrum for compound 42.

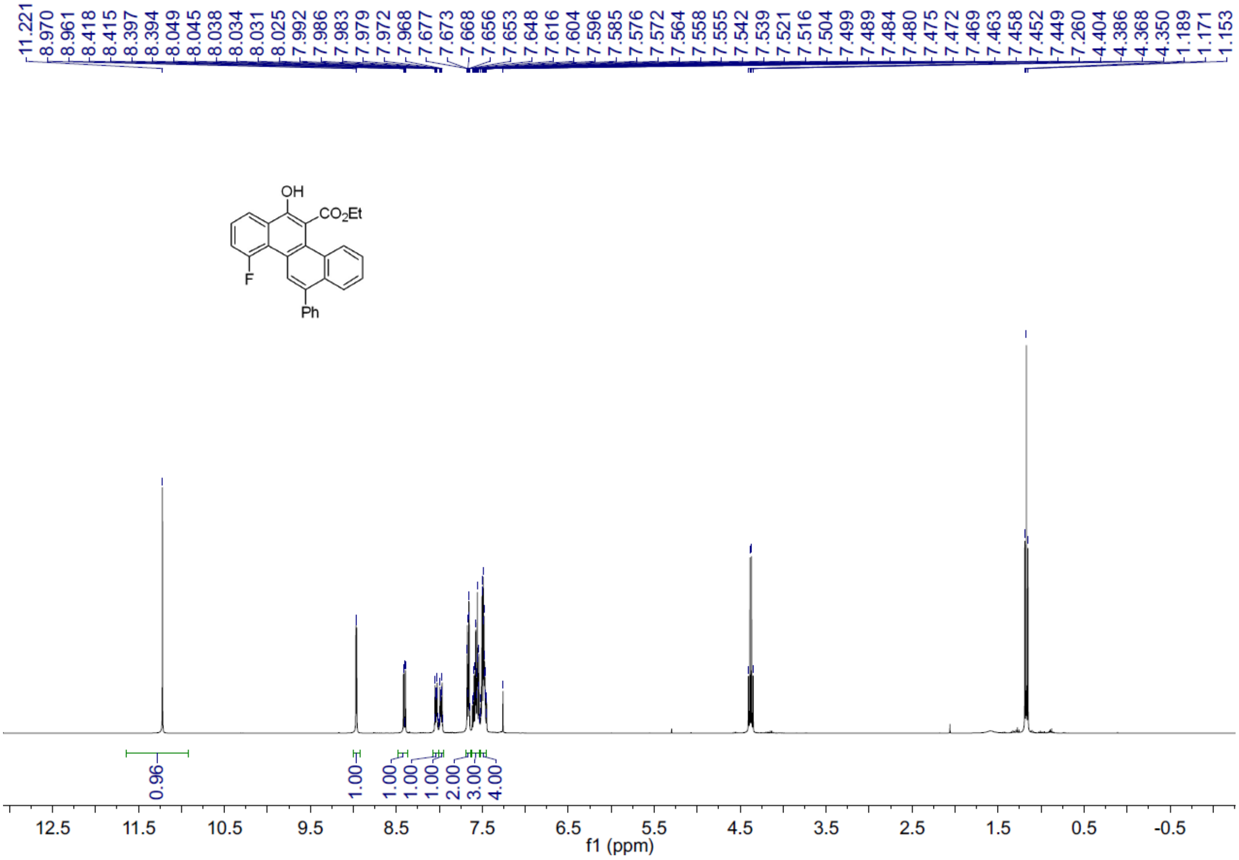

Supplementary Figure 102. ^{1}H NMR (400 MHz, $CDCl_3$) spectrum for compound 43.

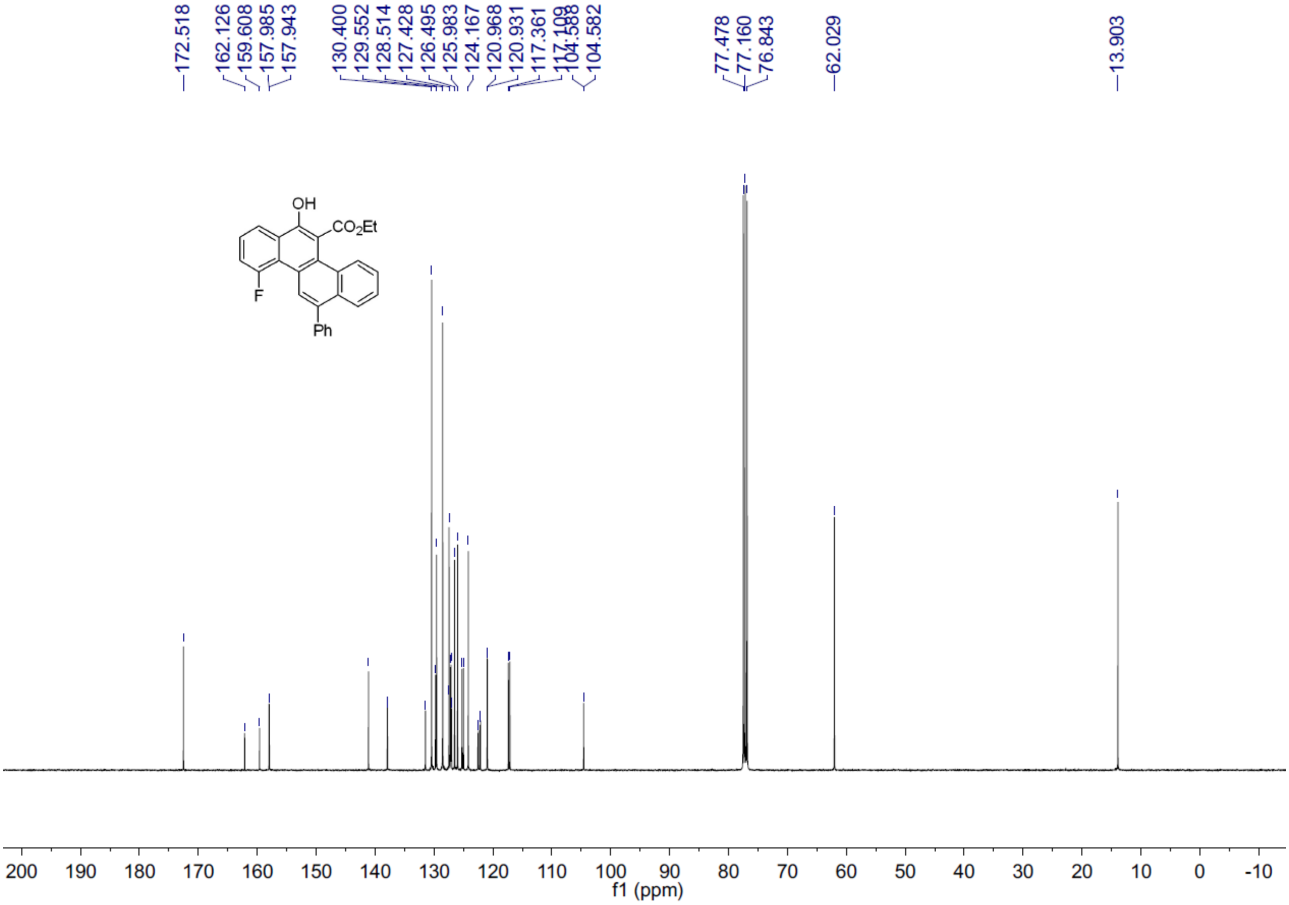

Supplementary Figure 103. ^{13}**C NMR (100 MHz, CDCl$_3$) spectrum for compound 43.**

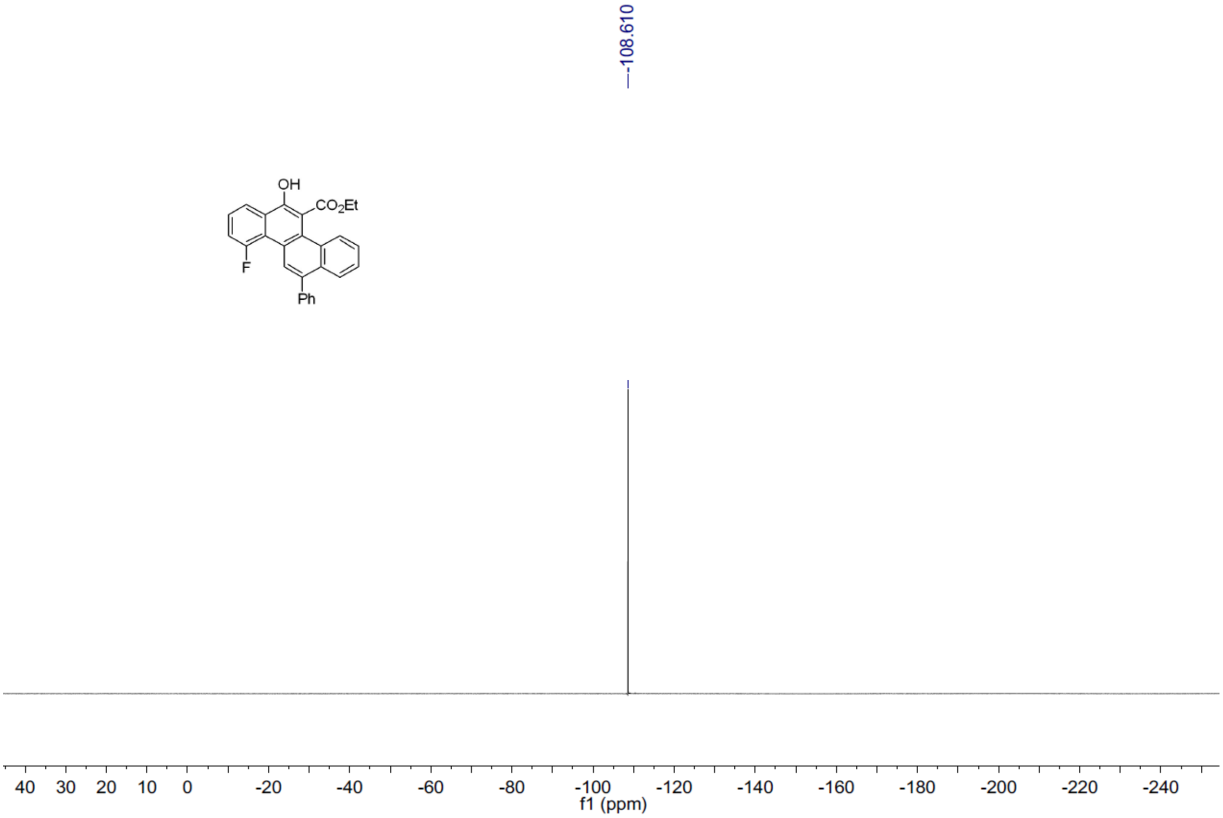

Supplementary Figure 104. ^{19}**F NMR (283 MHz, CDCl$_3$) spectrum for compound 43.**

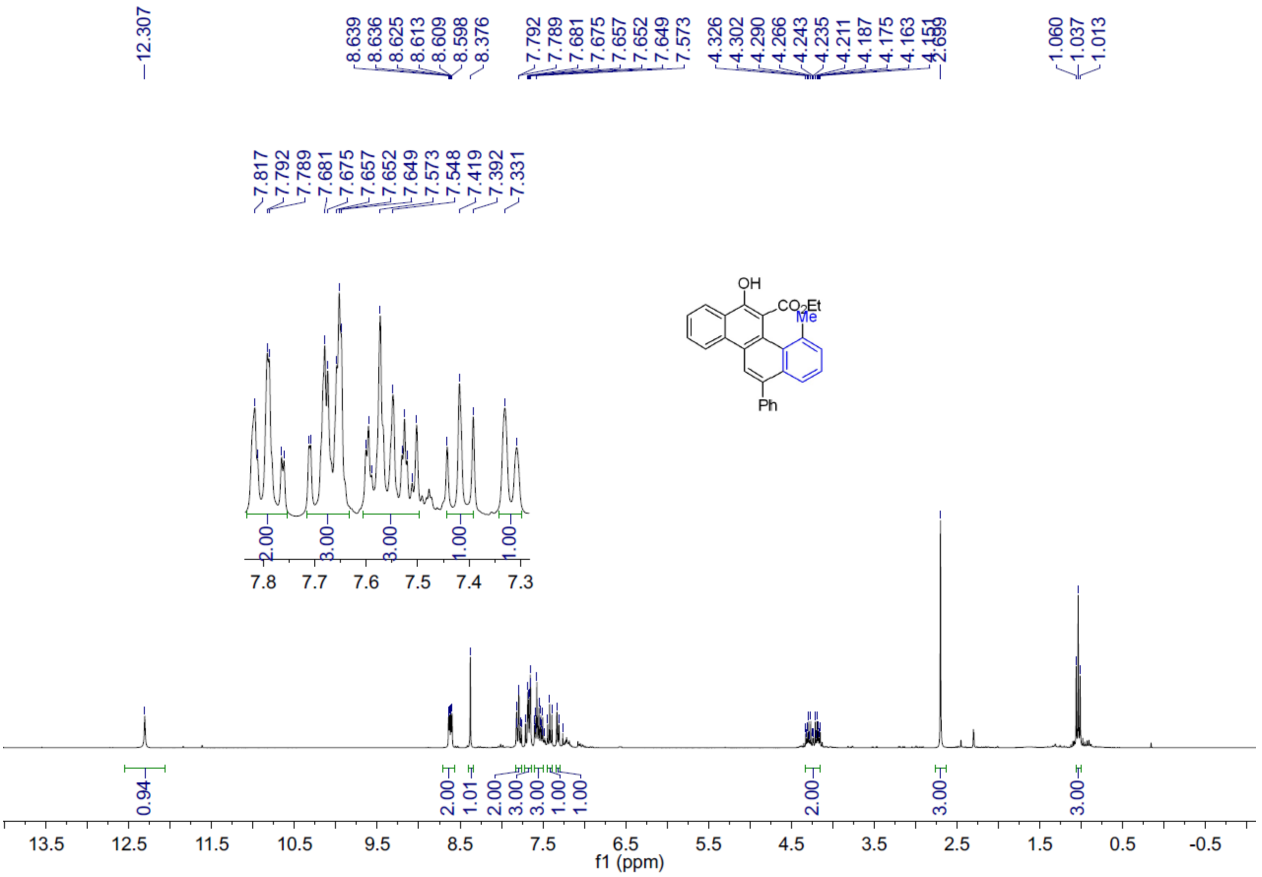

Supplementary Figure 105. **^{1}H NMR (300 MHz, $CDCl_3$) spectrum for compound 44.**

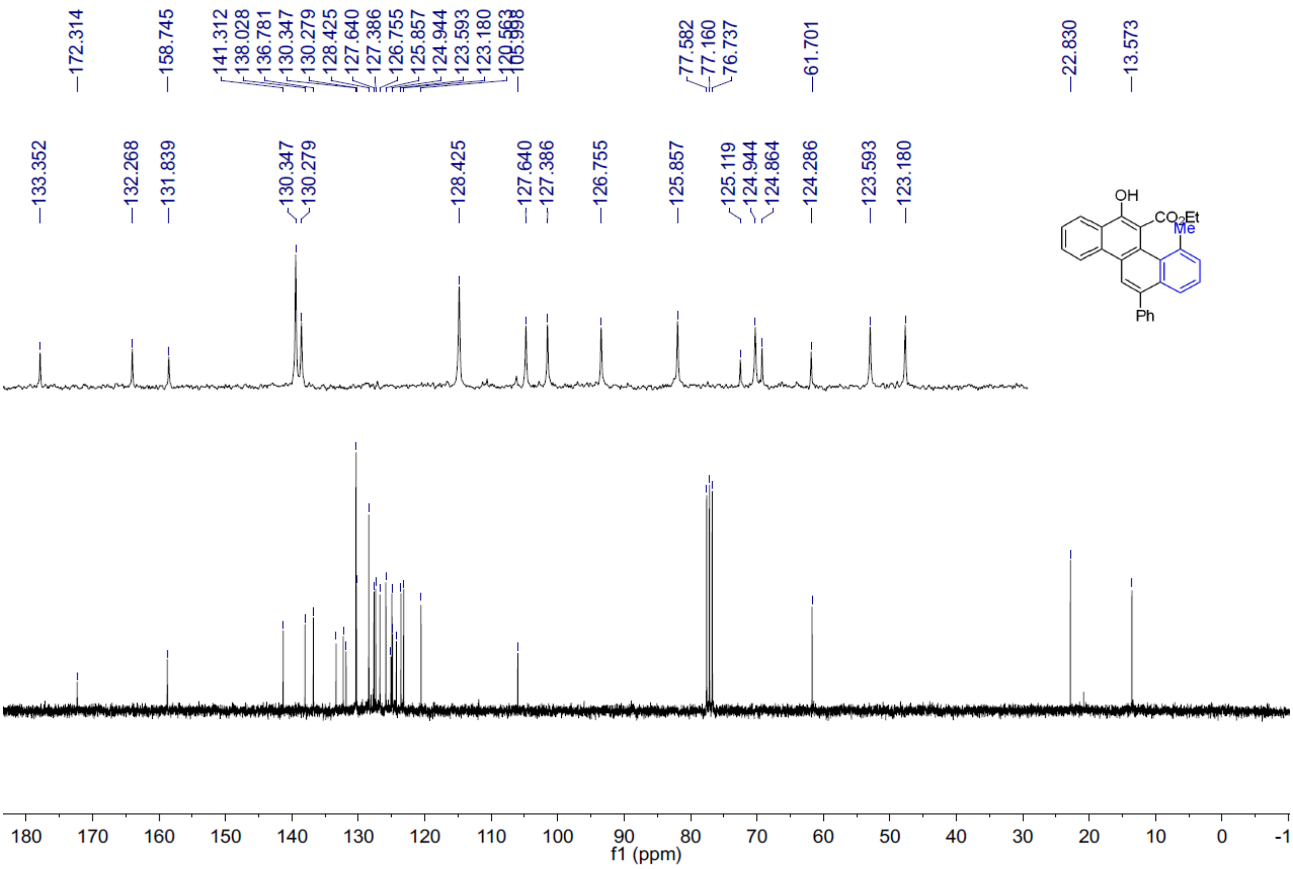

Supplementary Figure 106. **^{13}C NMR (75 MHz, $CDCl_3$) spectrum for compound 44.**

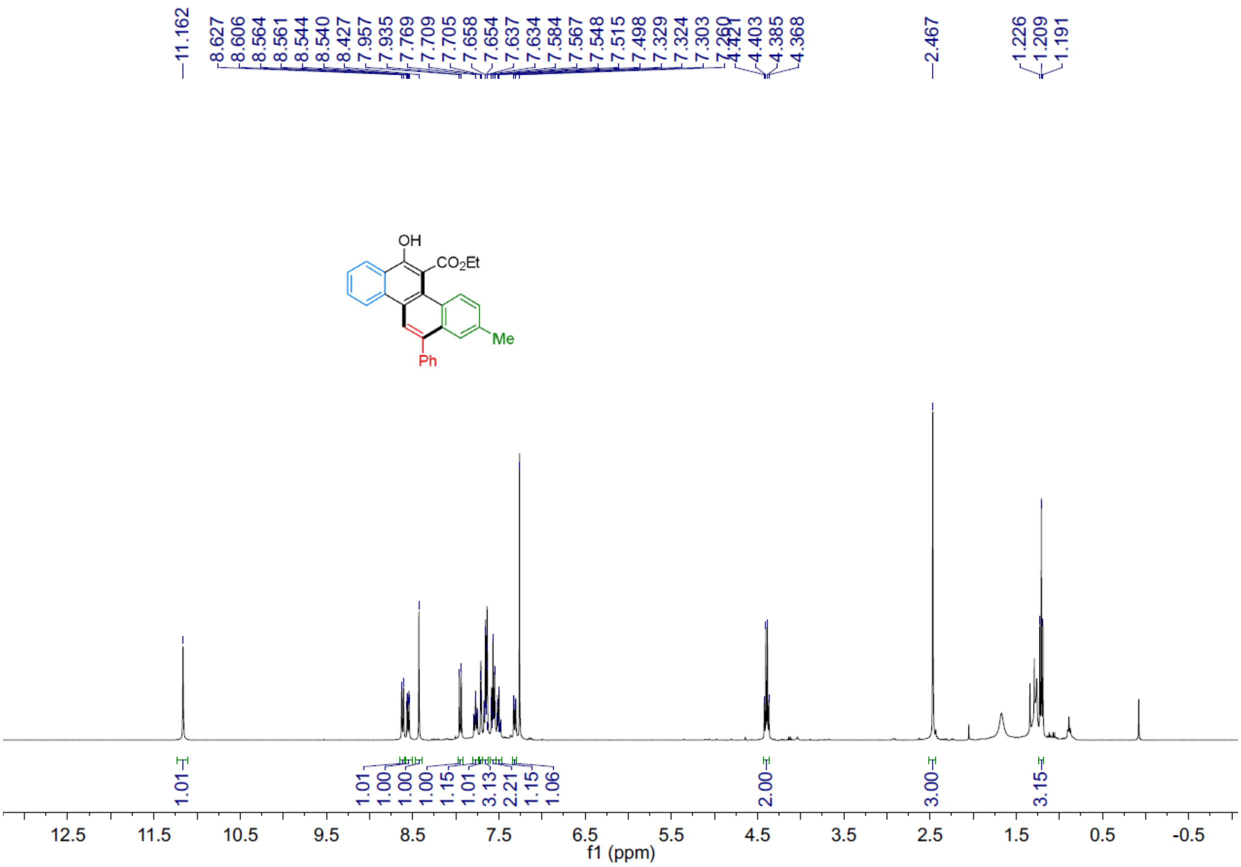

Supplementary Figure 107. ^{1}H NMR (400 MHz, $CDCl_3$) spectrum for compound 45.

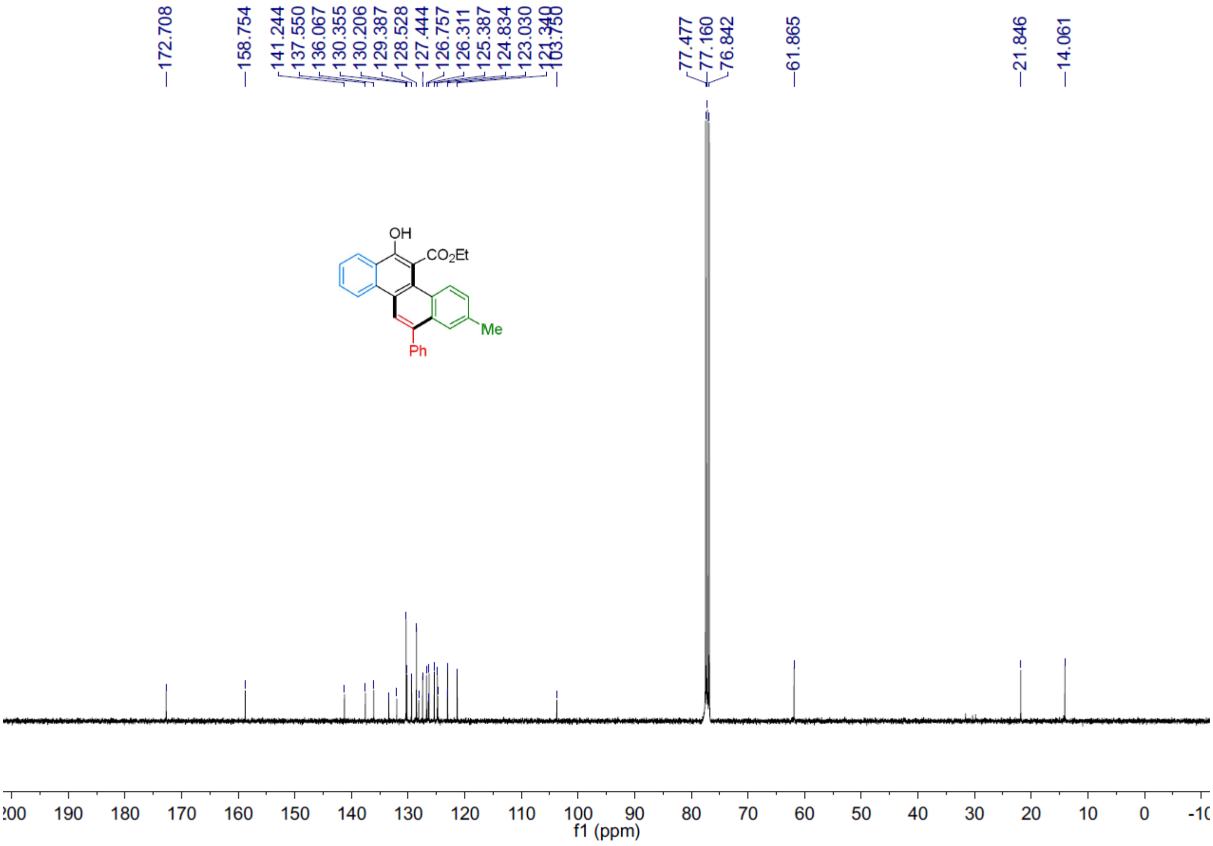

Supplementary Figure 108. ^{13}C NMR (100 MHz, $CDCl_3$) spectrum for compound 45.

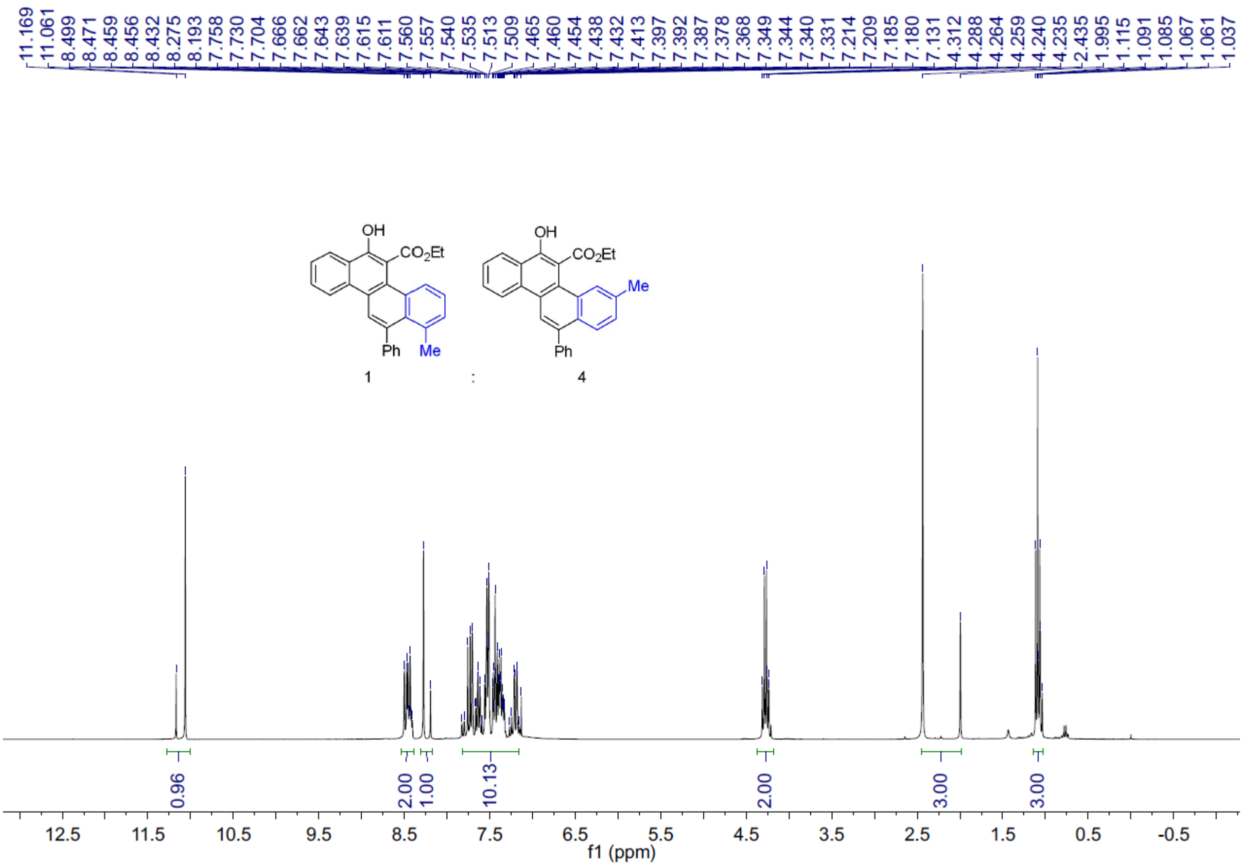

Supplementary Figure 109. ^{1}H NMR (300 MHz, $CDCl_3$) spectrum for compound 46.

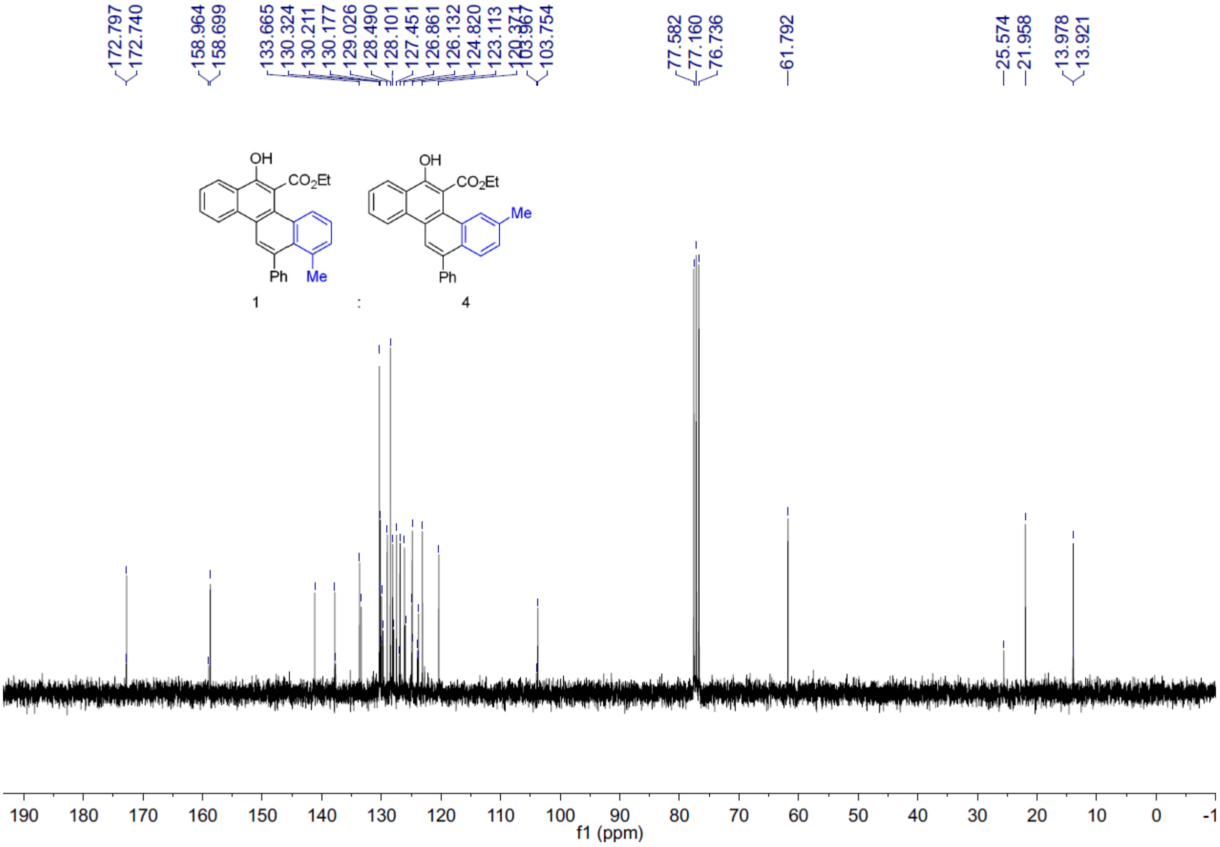

Supplementary Figure 110. ^{13}C NMR (75 MHz, $CDCl_3$) spectrum for compound 46.

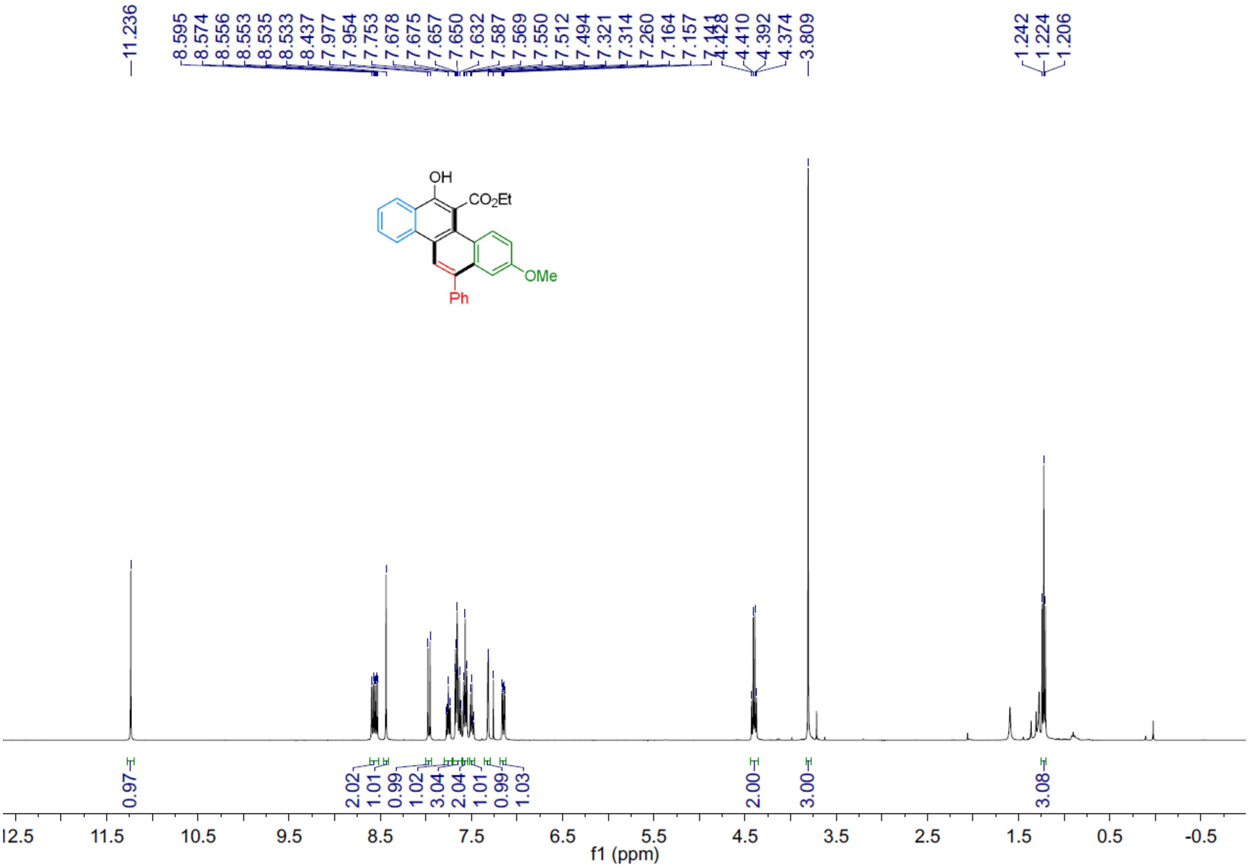

Supplementary Figure 111. ^{1}H NMR (400 MHz, $CDCl_3$) spectrum for compound 47.

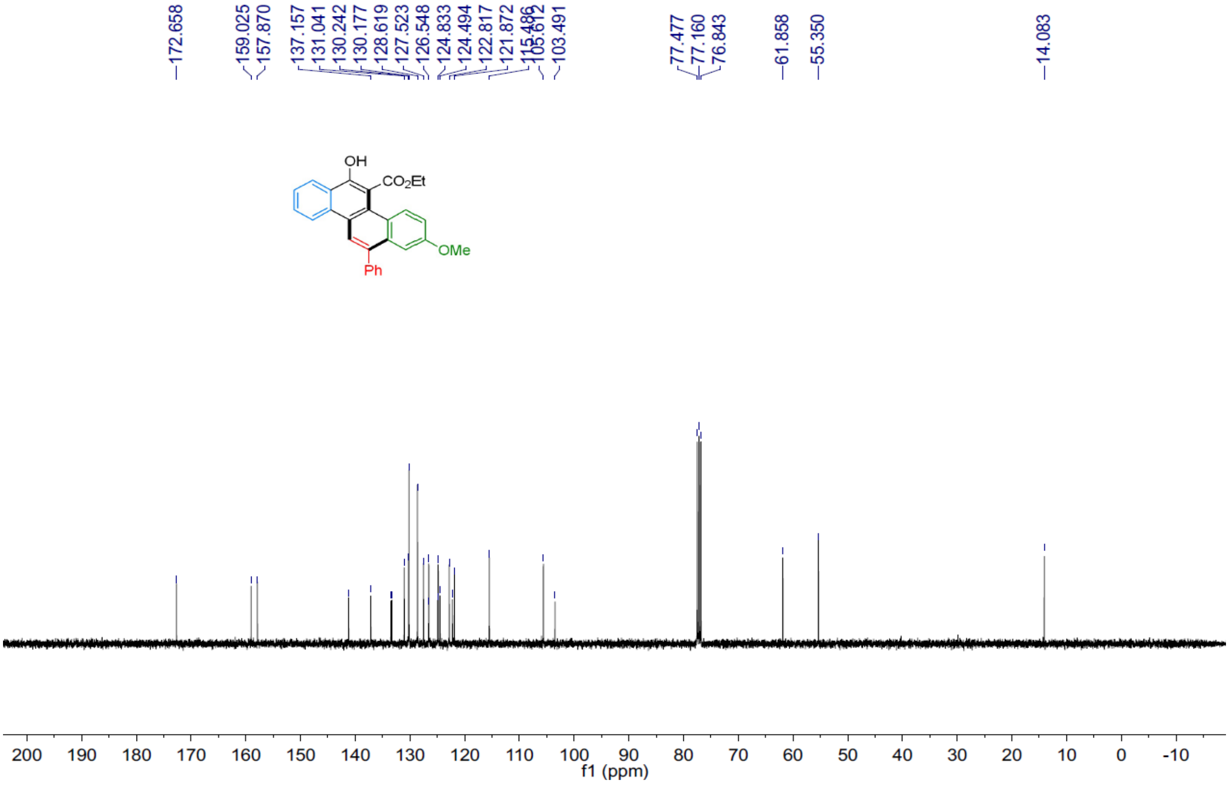

Supplementary Figure 112. ^{13}C NMR (100 MHz, $CDCl_3$) spectrum for compound 47.

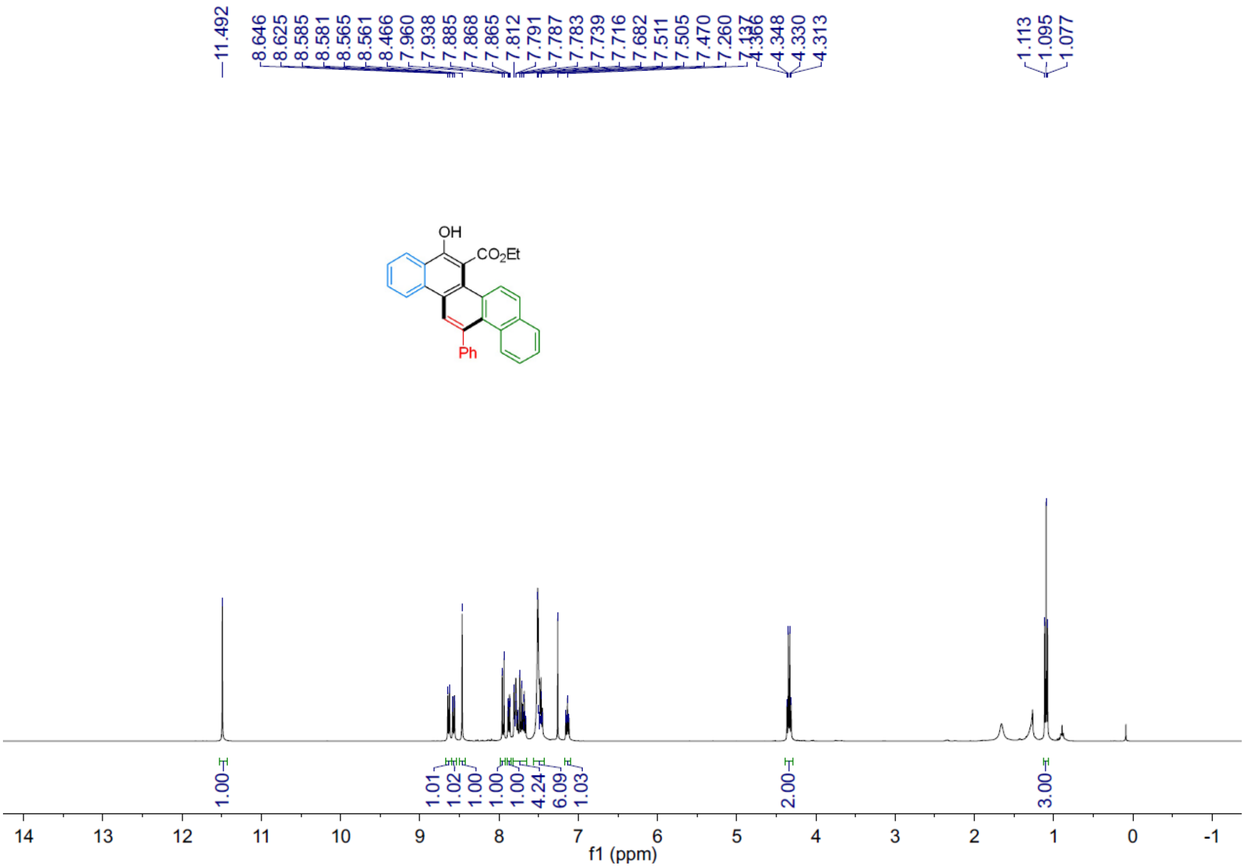

Supplementary Figure 113. ^{1}**H NMR (400 MHz, CDCl$_3$) spectrum for compound 48.**

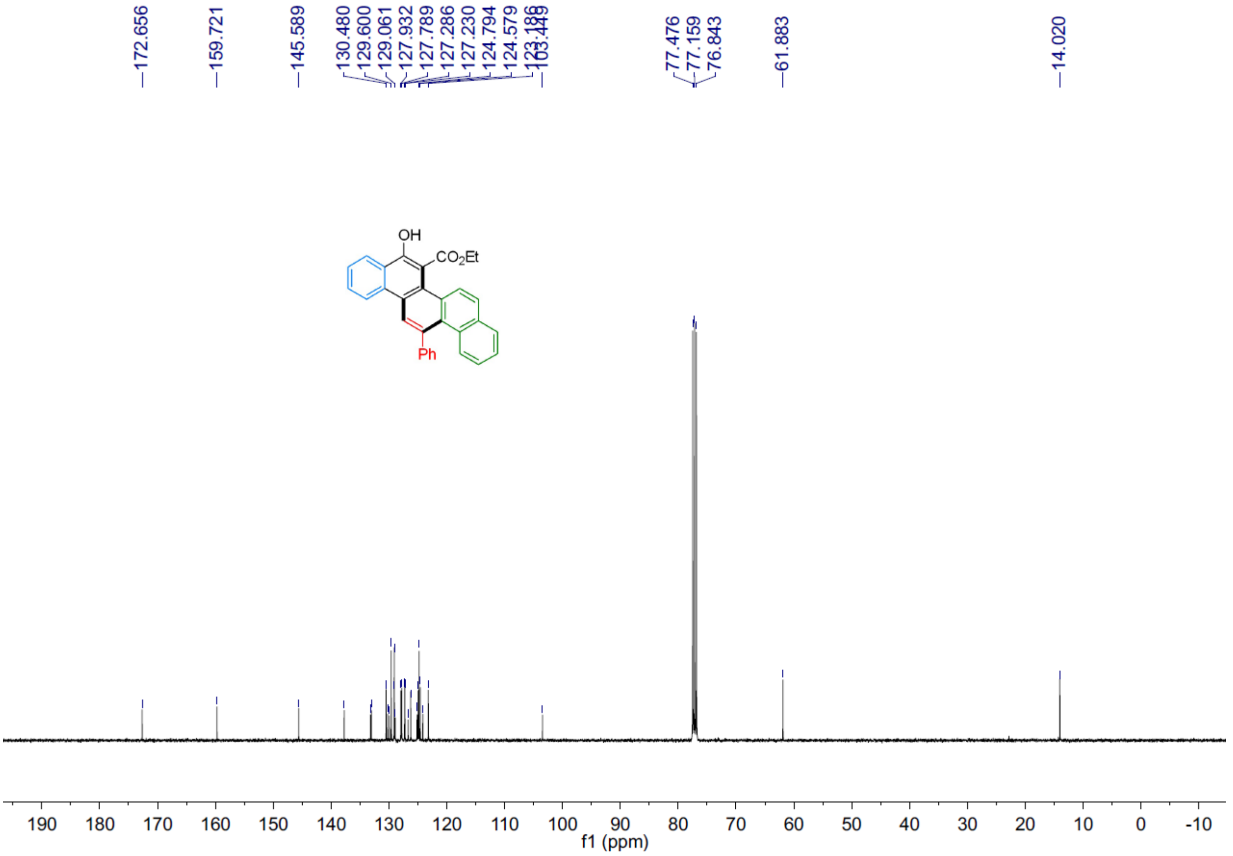

Supplementary Figure 114. ^{13}**C NMR (100 MHz, CDCl$_3$) spectrum for compound 48.**

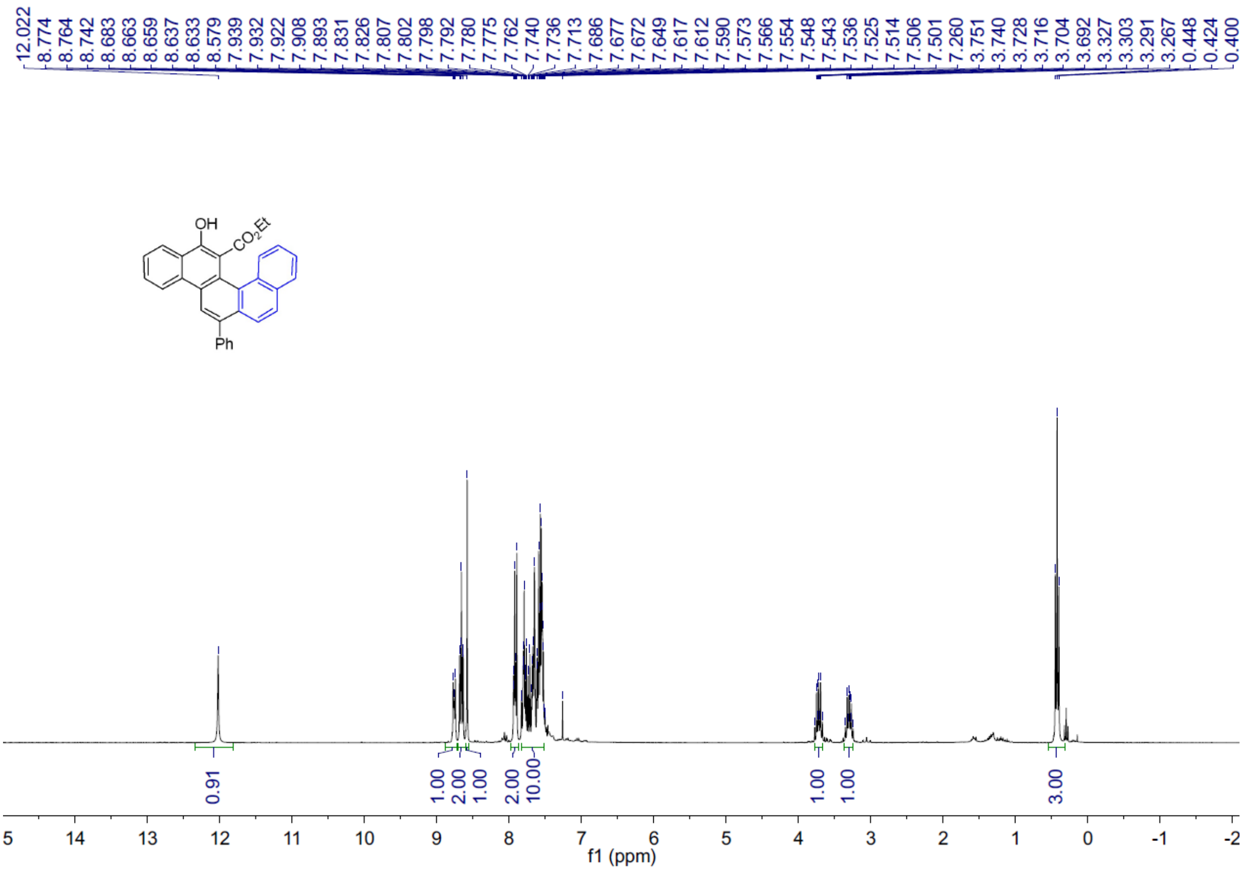

Supplementary Figure 115. ^{1}H NMR (300 MHz, $CDCl_3$) spectrum for compound 49.

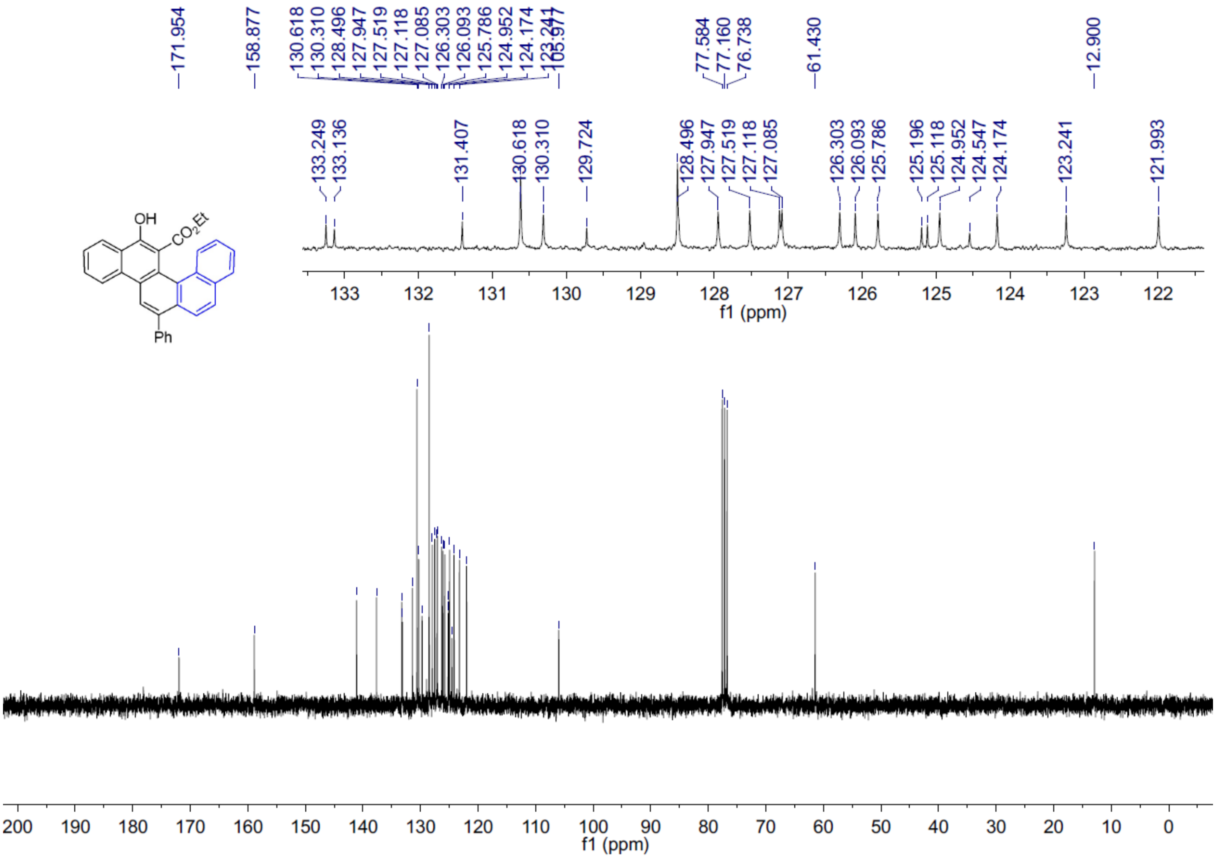

Supplementary Figure 116. ^{13}C NMR (75 MHz, $CDCl_3$) spectrum for compound 49.

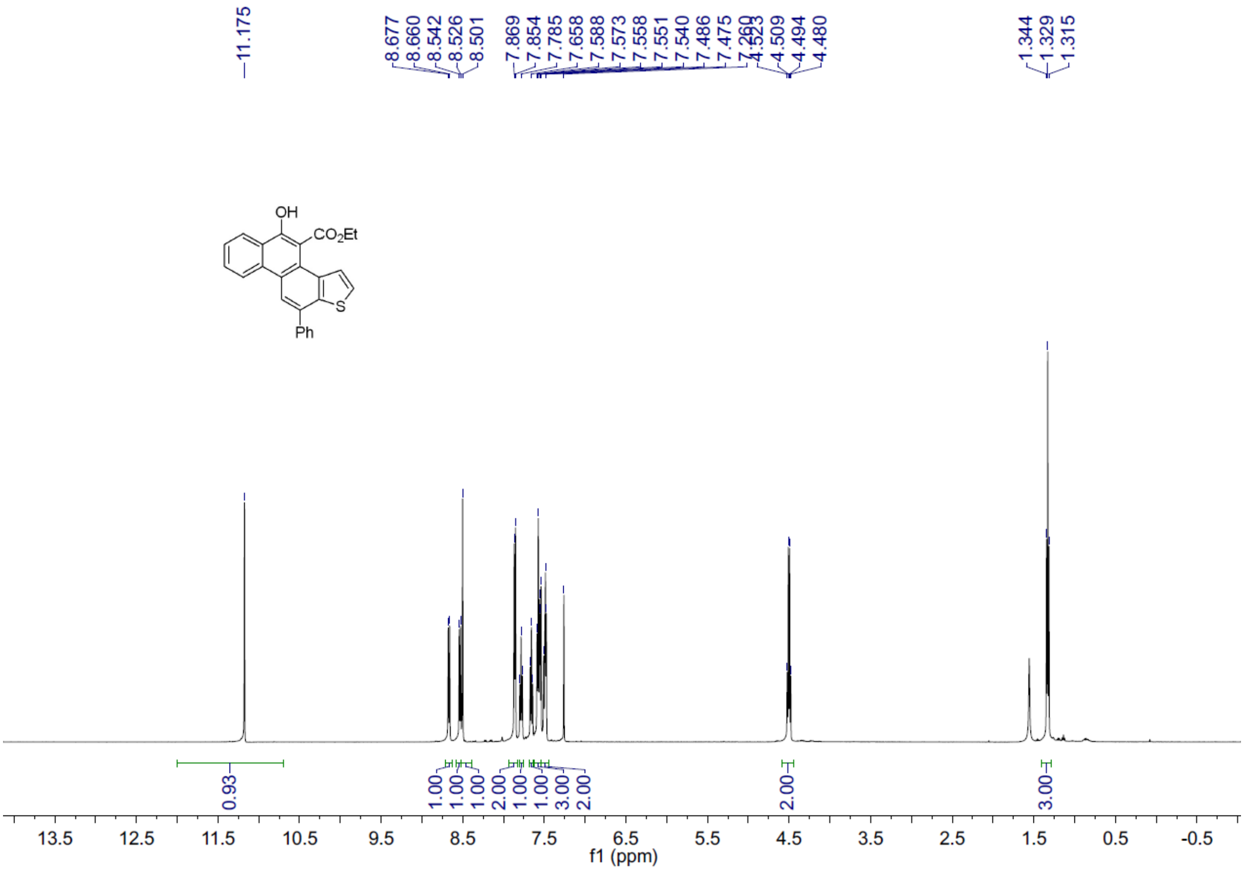

Supplementary Figure 117. **^{1}H NMR (500 MHz, $CDCl_3$) spectrum for compound 50.**

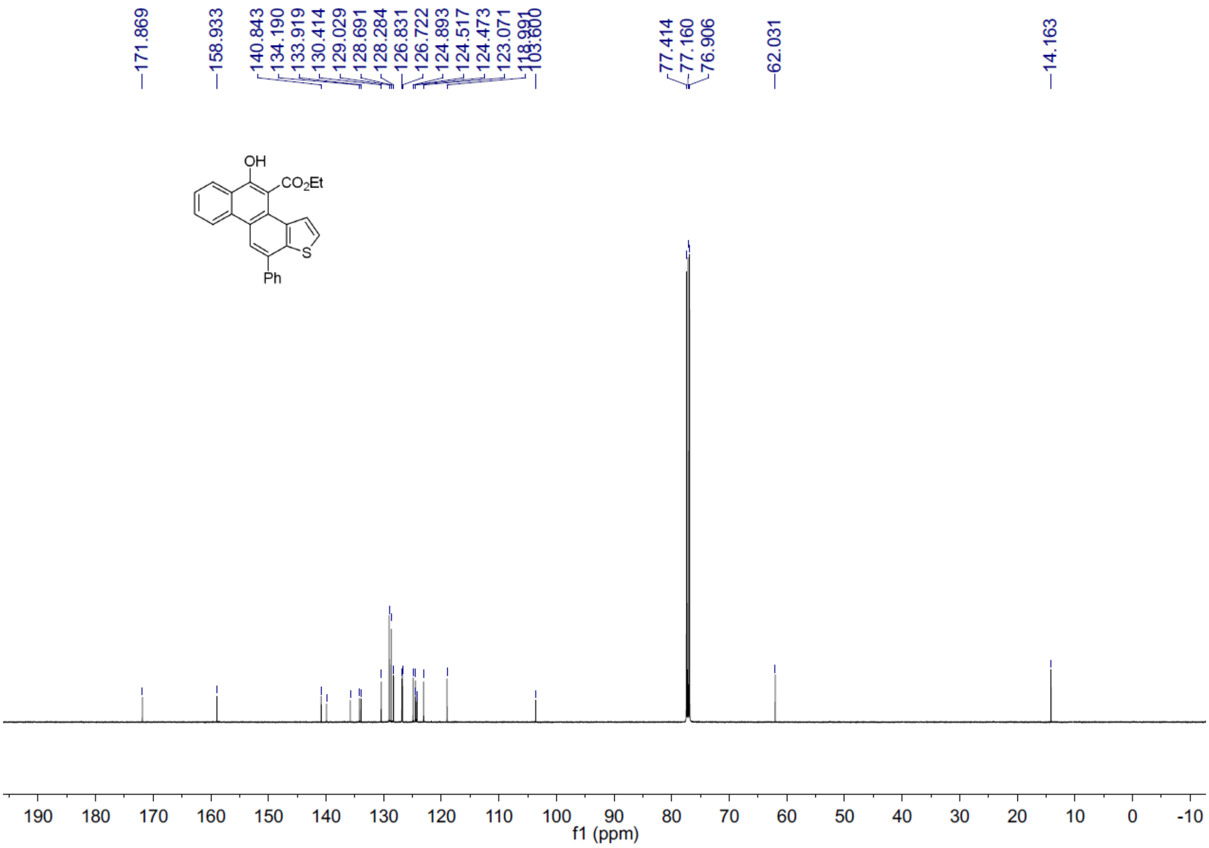

Supplementary Figure 118. **^{13}C NMR (125 MHz, $CDCl_3$) spectrum for compound 50.**

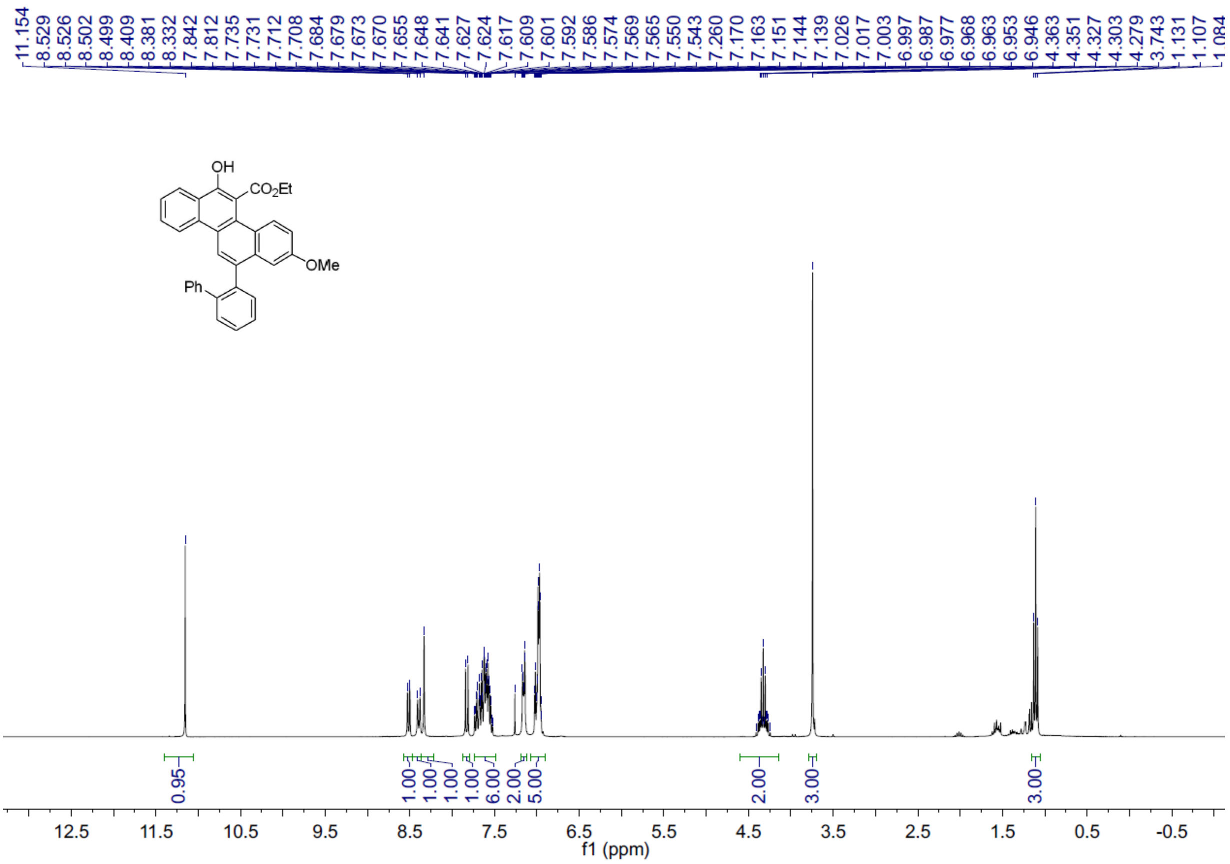

Supplementary Figure 119. ^{1}H NMR (400 MHz, $CDCl_3$) spectrum for compound 51.

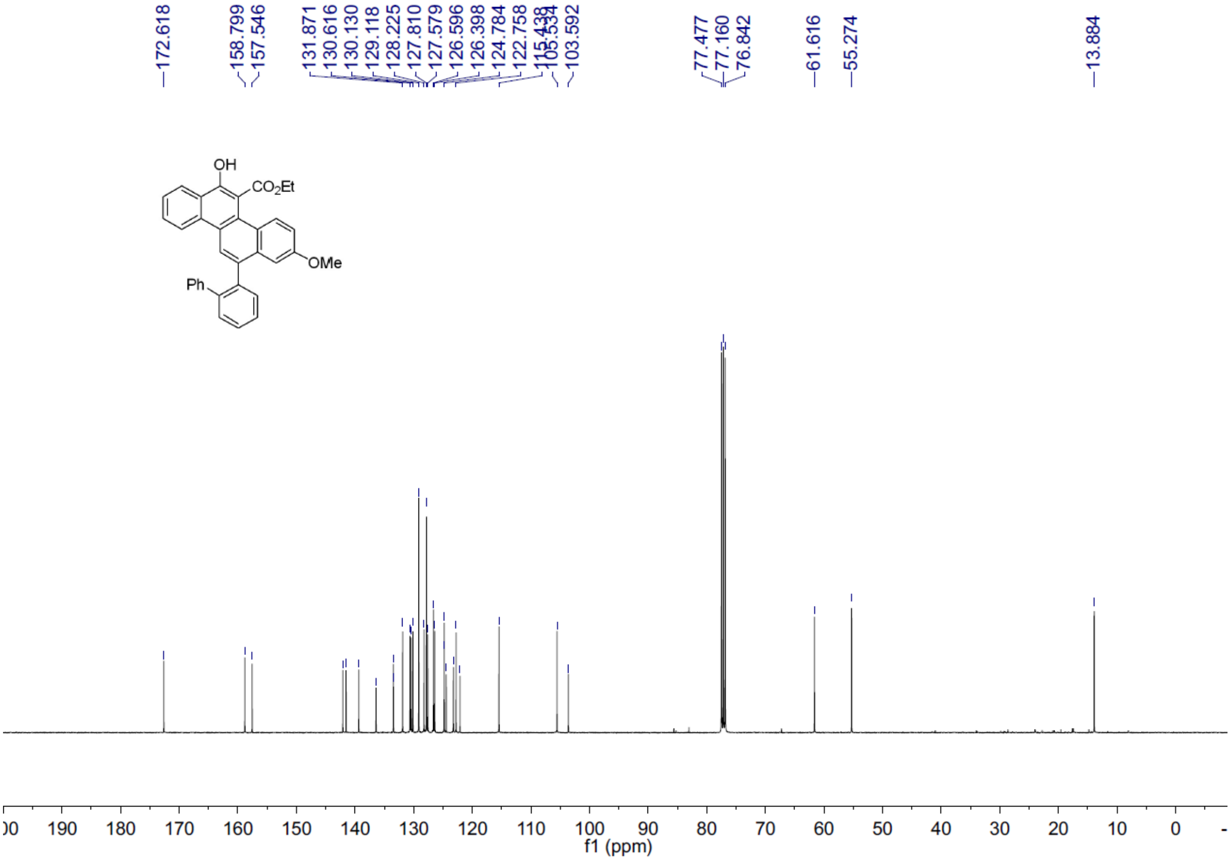

Supplementary Figure 120. ^{13}C NMR (100 MHz, $CDCl_3$) spectrum for compound 51.

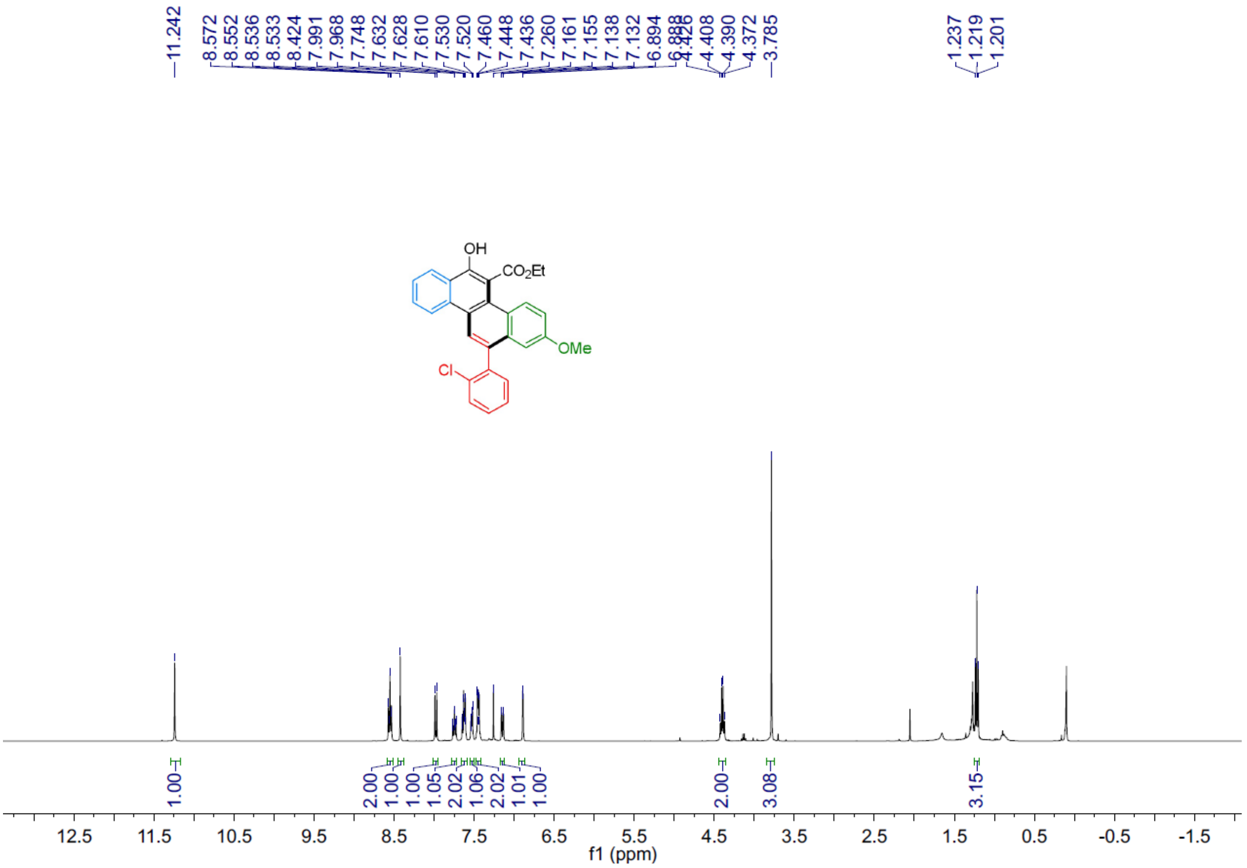

Supplementary Figure 121. ^{1}H NMR (400 MHz, $CDCl_3$) spectrum for compound 52.

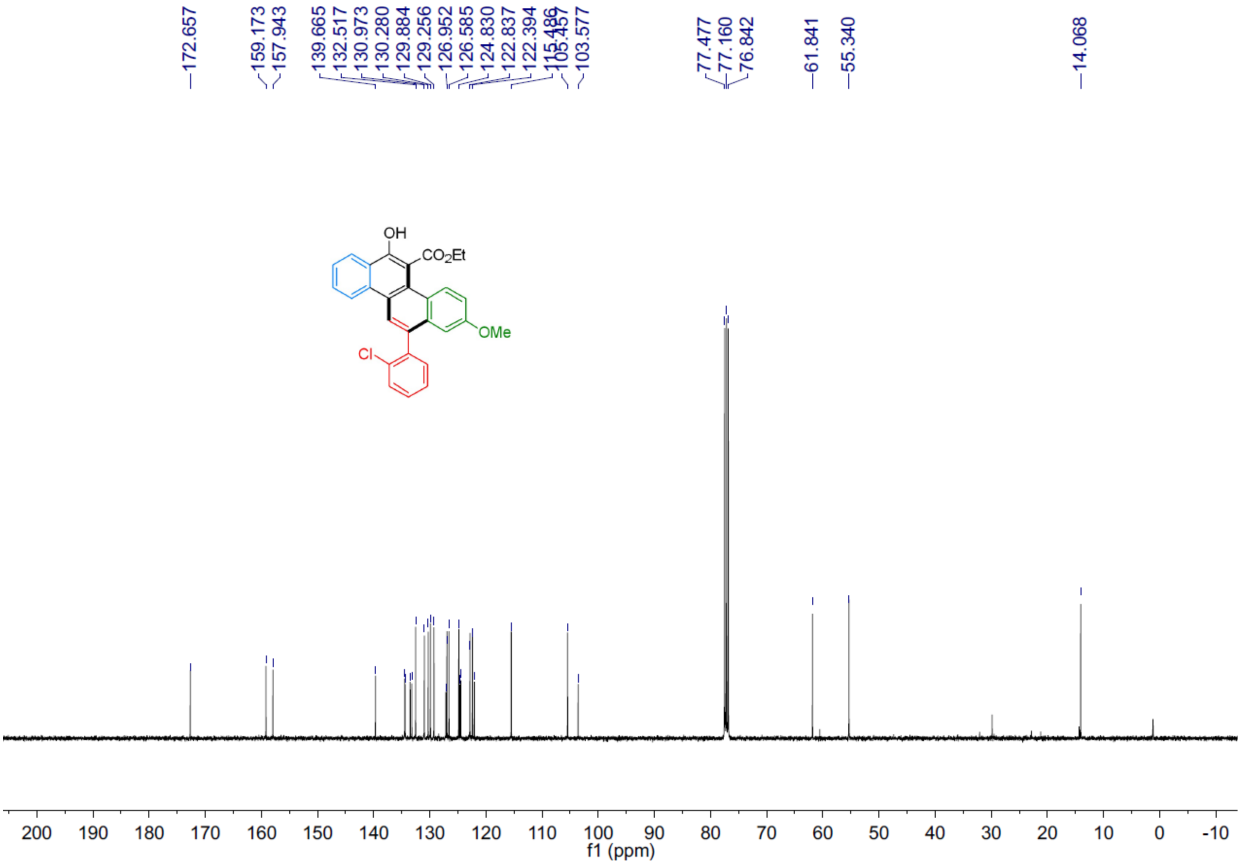

Supplementary Figure 122. ^{13}C NMR (100 MHz, $CDCl_3$) spectrum for compound 52.

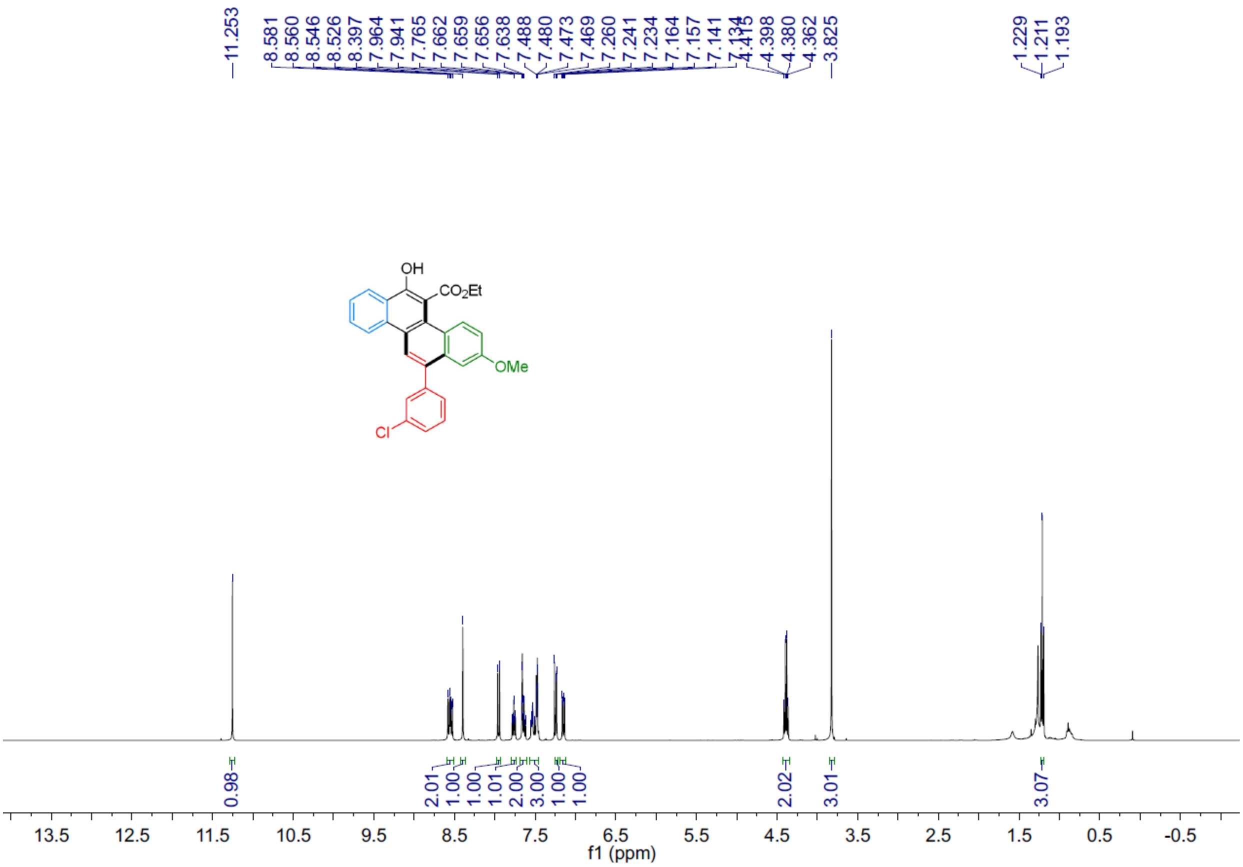

Supplementary Figure 123. ^{1}H NMR (400 MHz, $CDCl_3$) spectrum for compound 53.

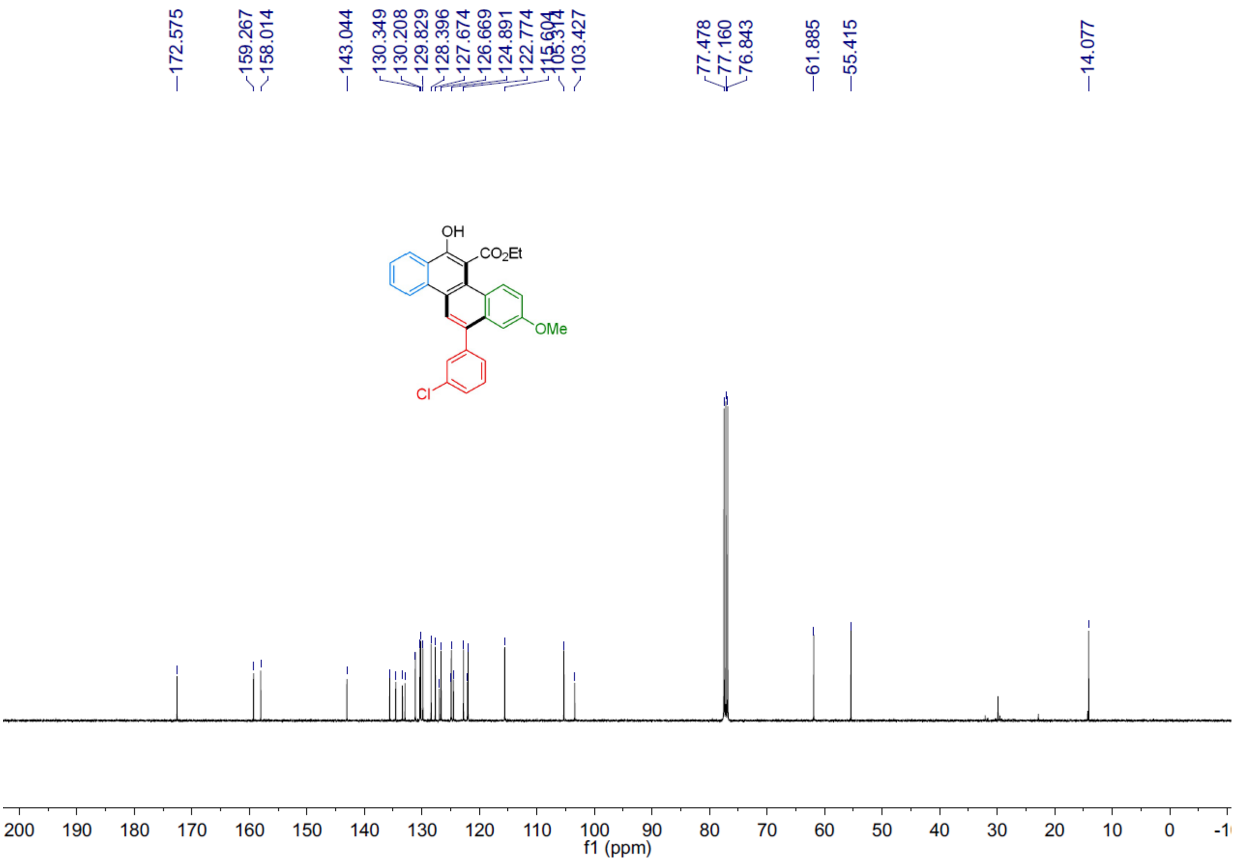

Supplementary Figure 124. ^{13}C NMR (100 MHz, $CDCl_3$) spectrum for compound 53.

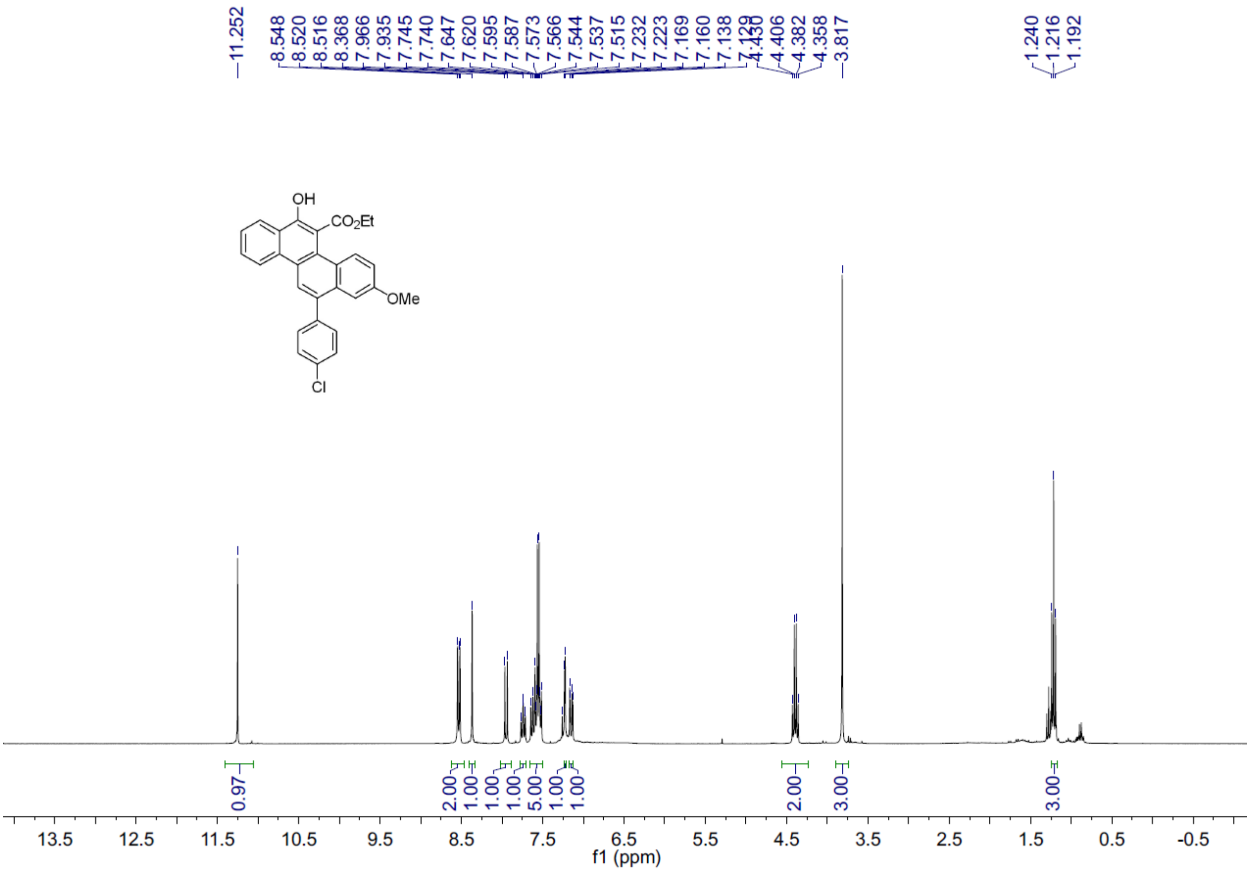

Supplementary Figure 125. ^{1}H NMR (300 MHz, $CDCl_3$) spectrum for compound 54.

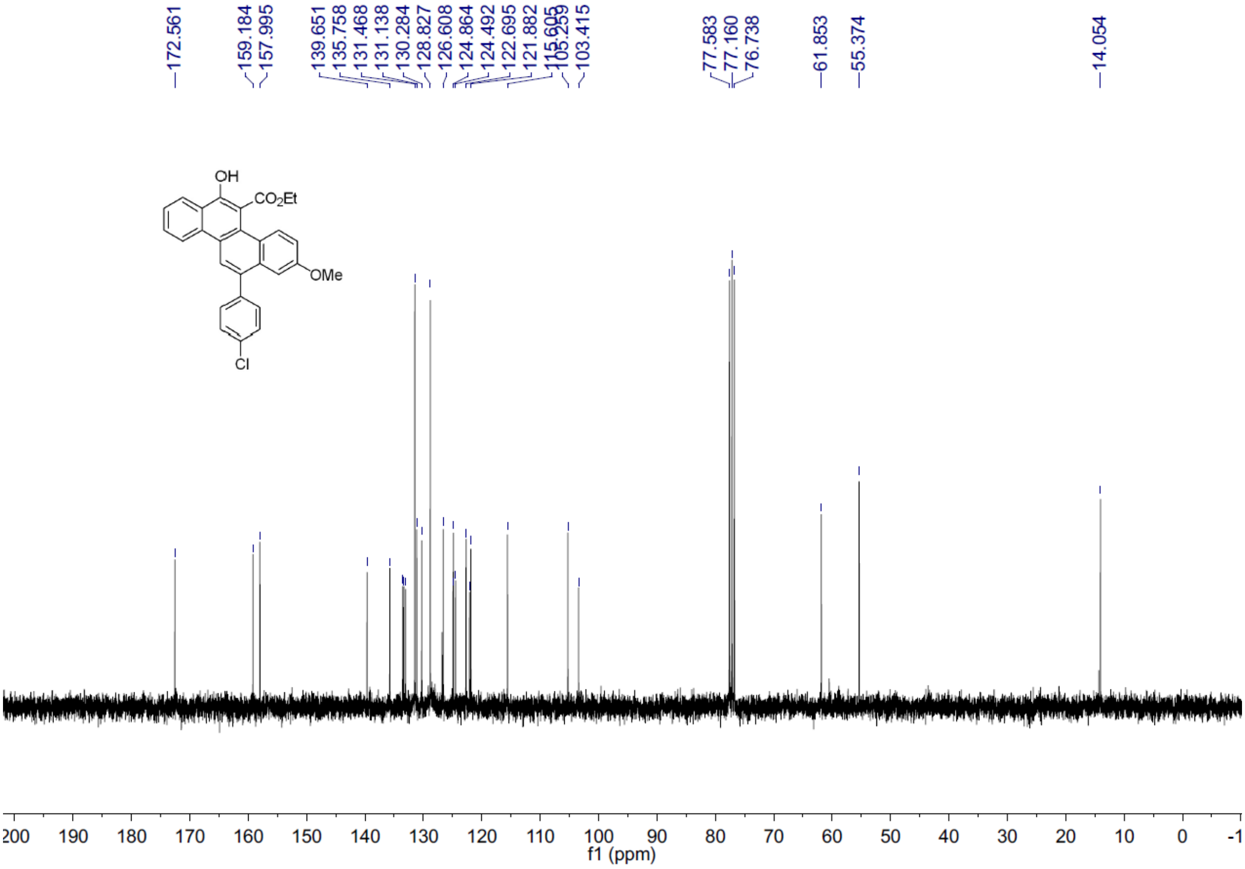

Supplementary Figure 126. ^{13}C NMR (75 MHz, $CDCl_3$) spectrum for compound 54.

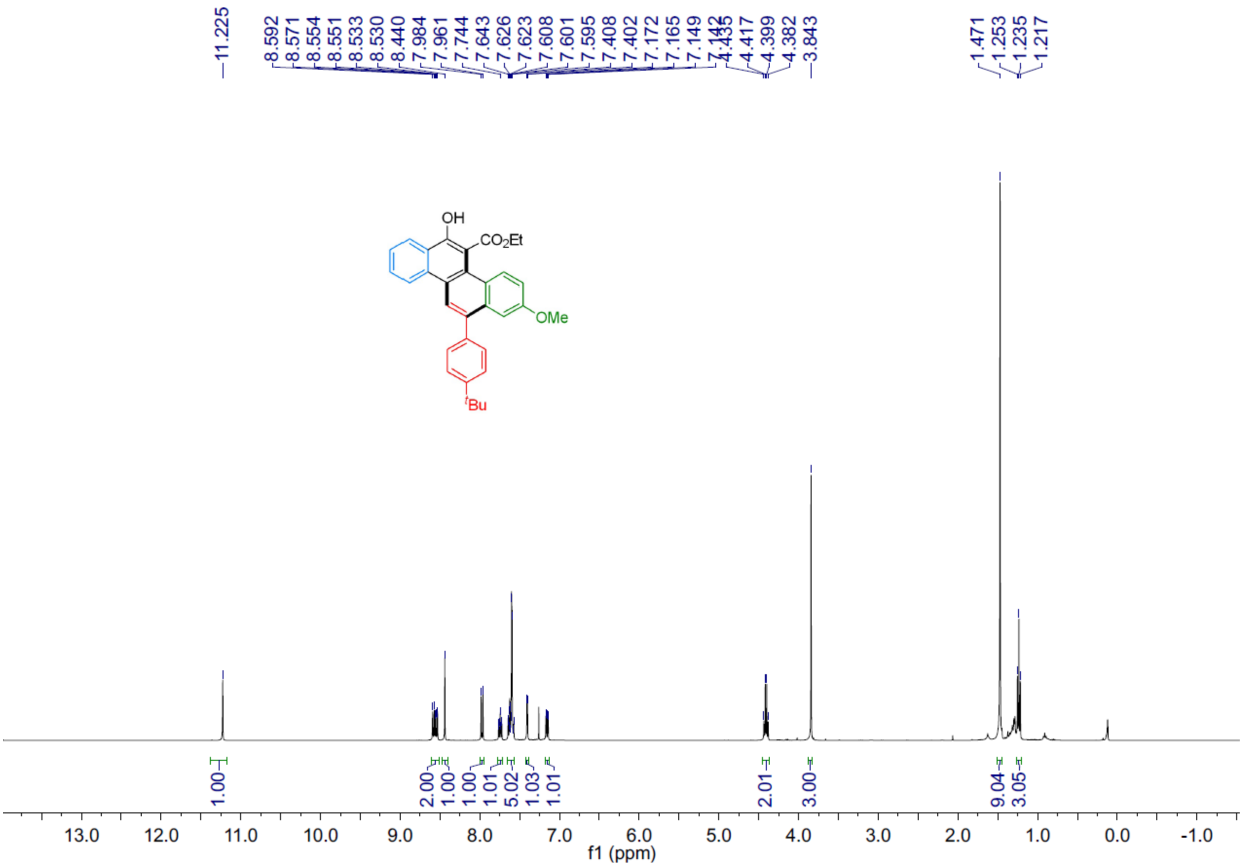

Supplementary Figure 127. ^{1}H NMR (400 MHz, $CDCl_3$) spectrum for compound 55.

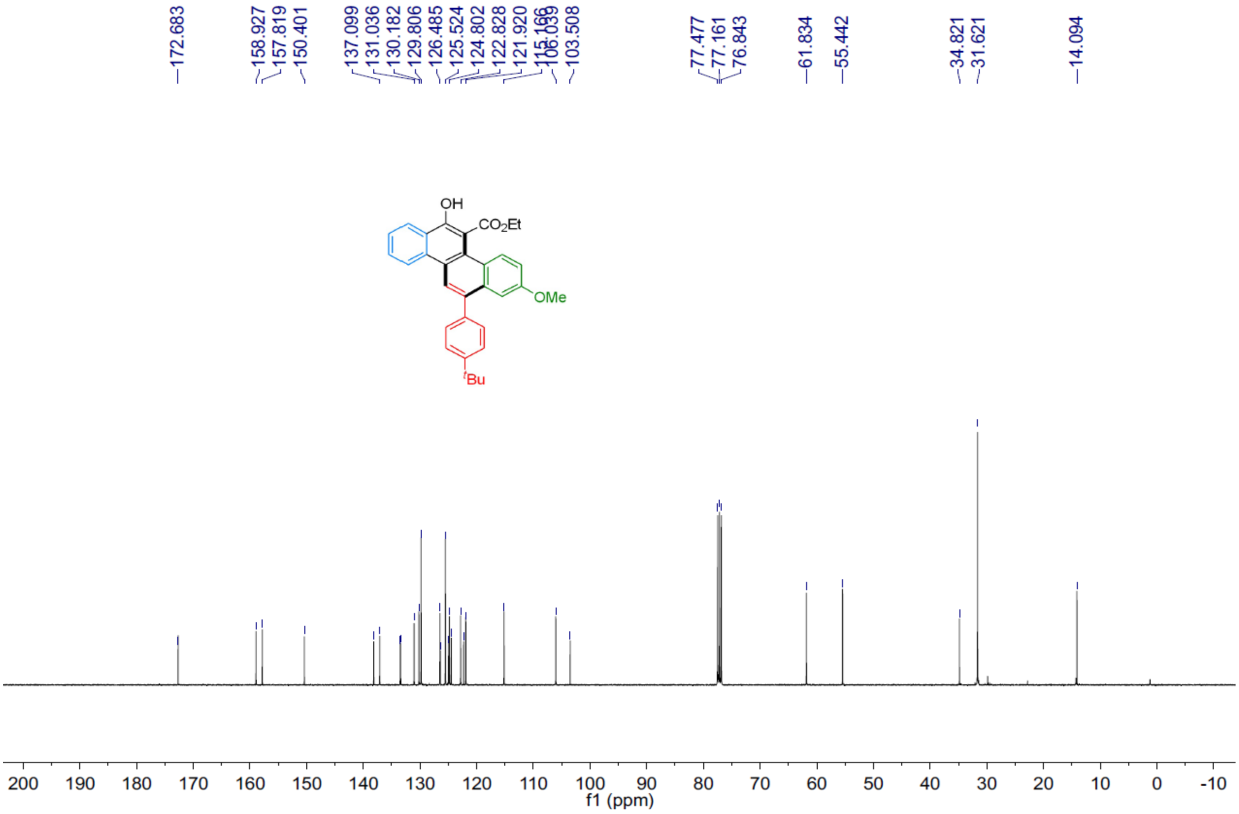

Supplementary Figure 128. ^{13}C NMR (100 MHz, $CDCl_3$) spectrum for compound 55.

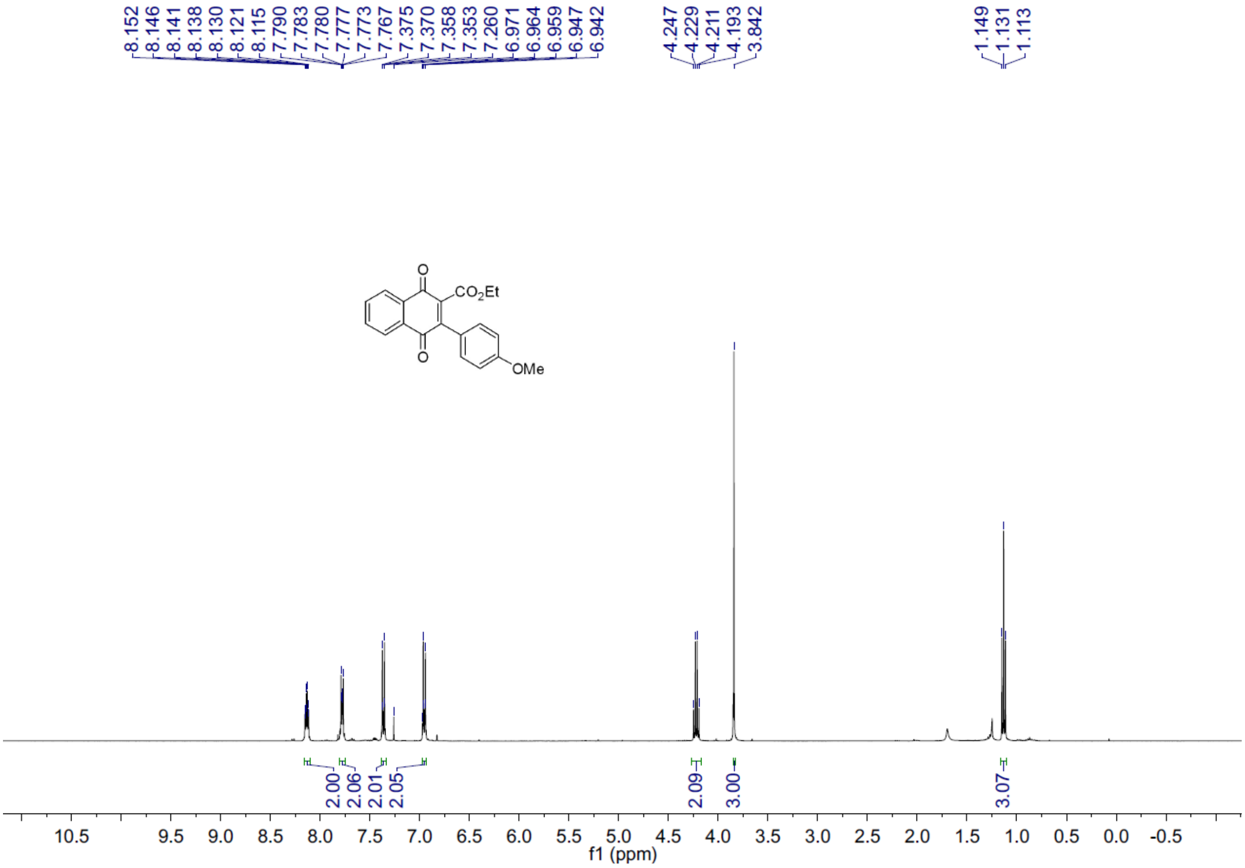

Supplementary Figure 129. ^{1}H NMR (400 MHz, $CDCl_3$) spectrum for compound 56.

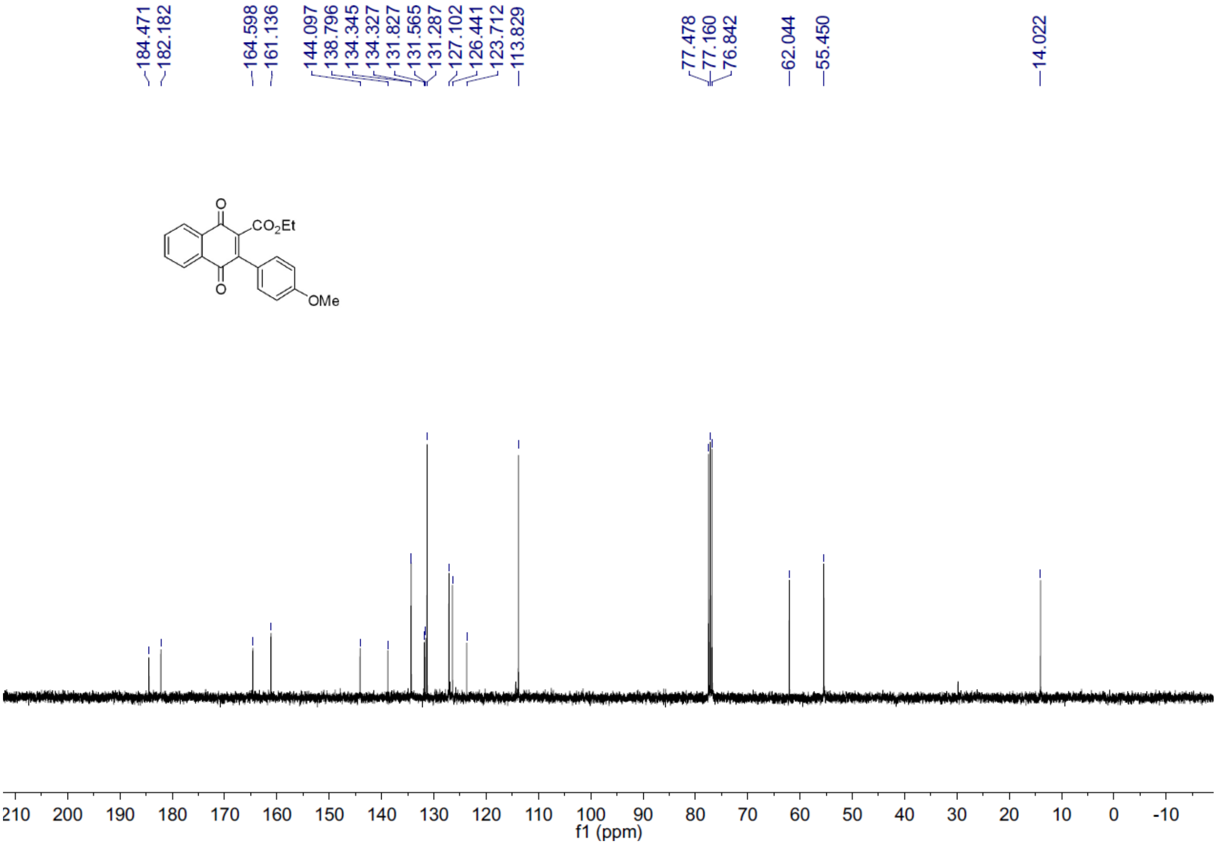

Supplementary Figure 130. ^{13}C NMR (100 MHz, $CDCl_3$) spectrum for compound 56.

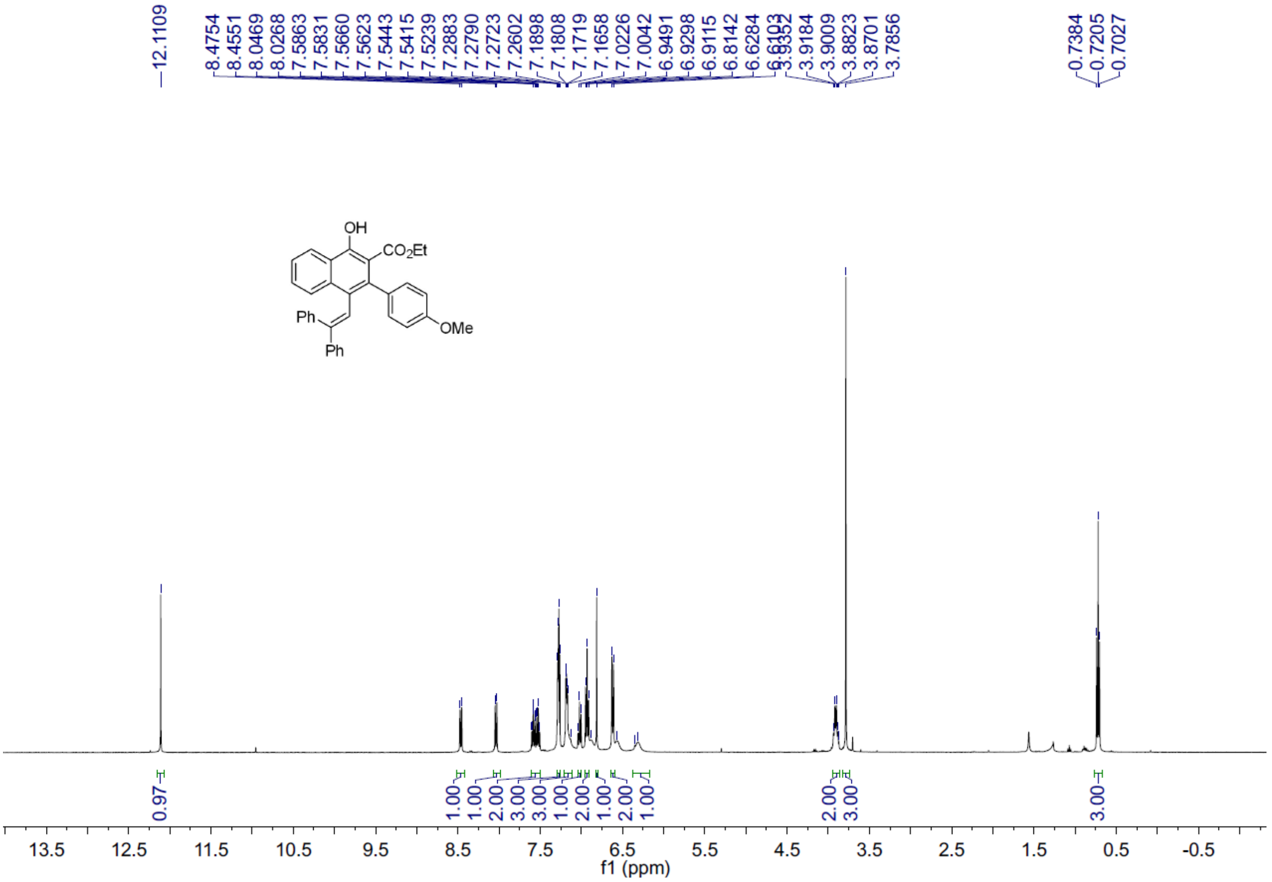

Supplementary Figure 131. ^{1}H NMR (400 MHz, $CDCl_3$) spectrum for compound 57.

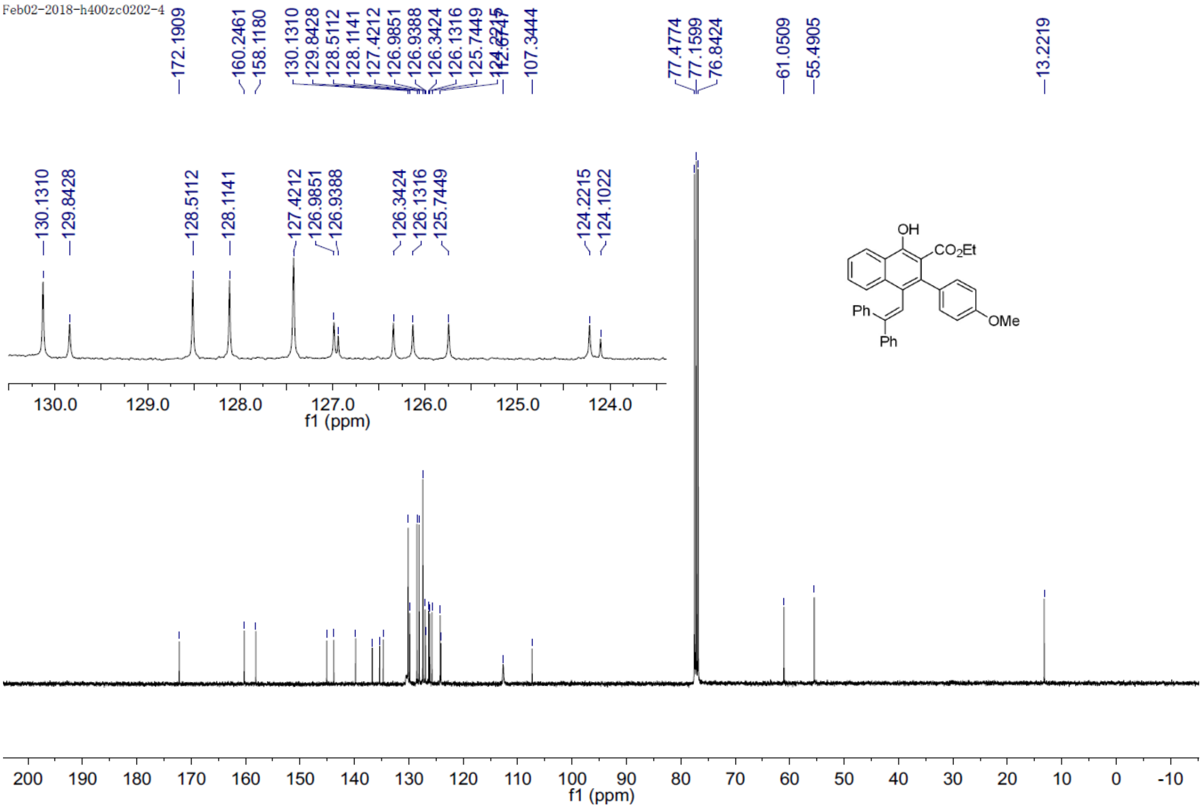

Supplementary Figure 132. ^{13}C NMR (100 MHz, $CDCl_3$) spectrum for compound 57.

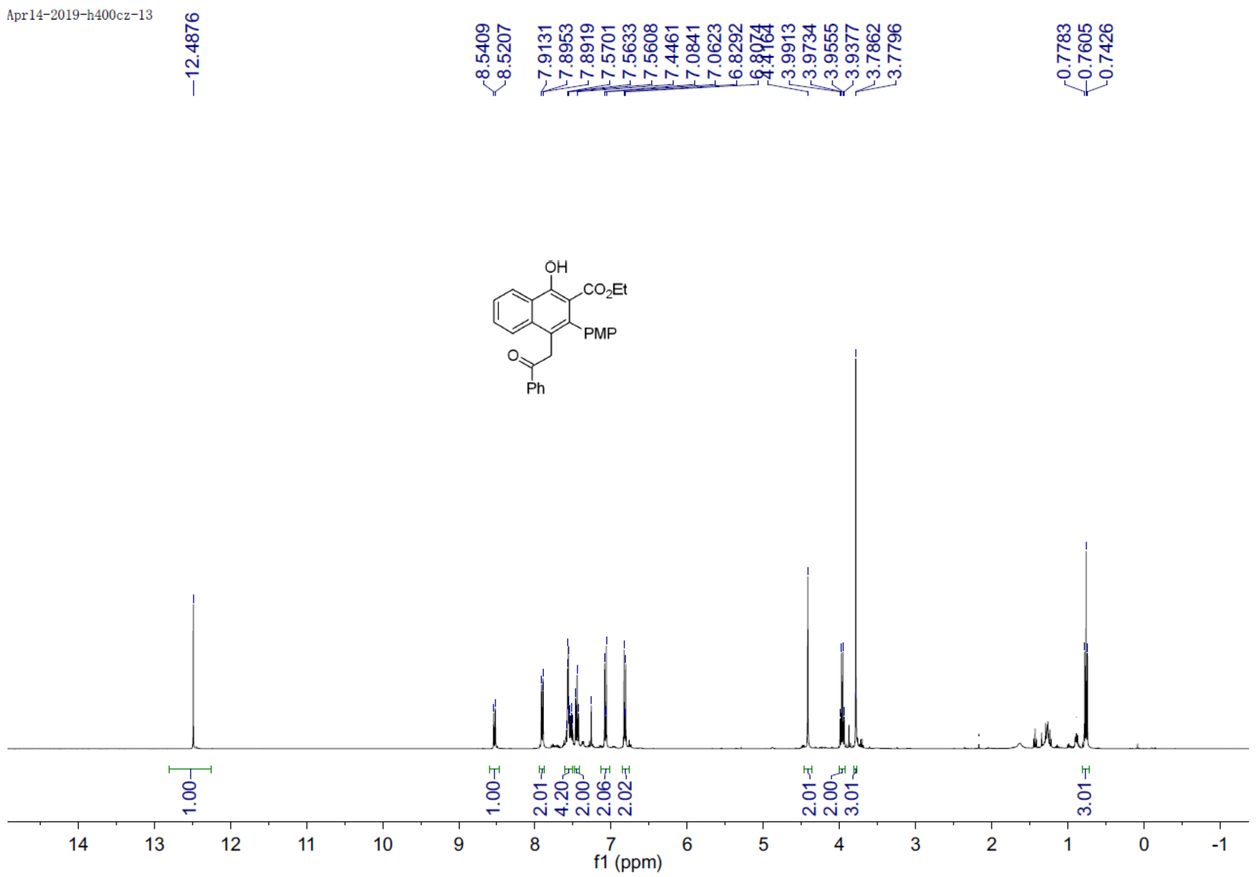

Supplementary Figure 133. ^{1}**H NMR (400 MHz, CDCl$_3$) spectrum for compound 58.**

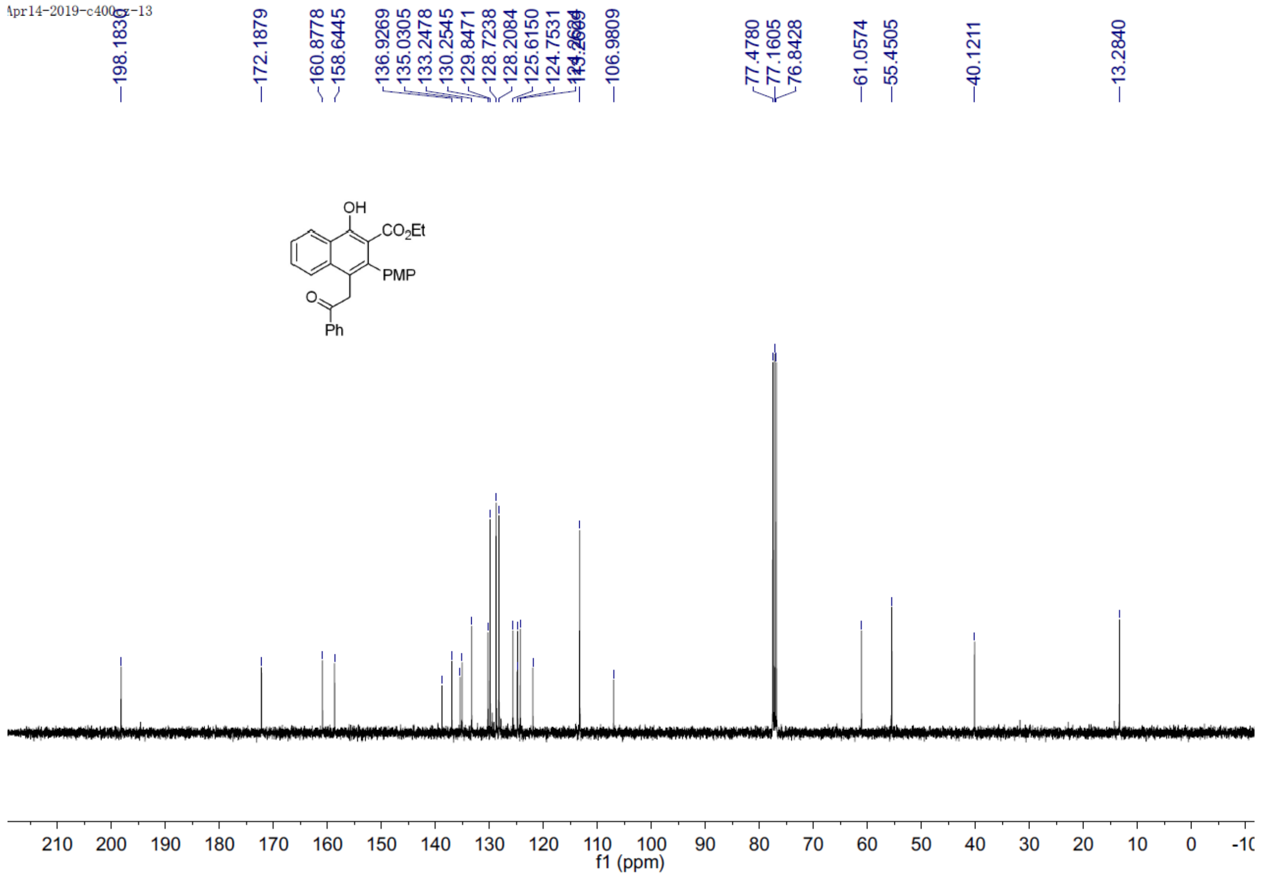

Supplementary Figure 134. ^{13}**C NMR (100 MHz, CDCl$_3$) spectrum for compound 58.**

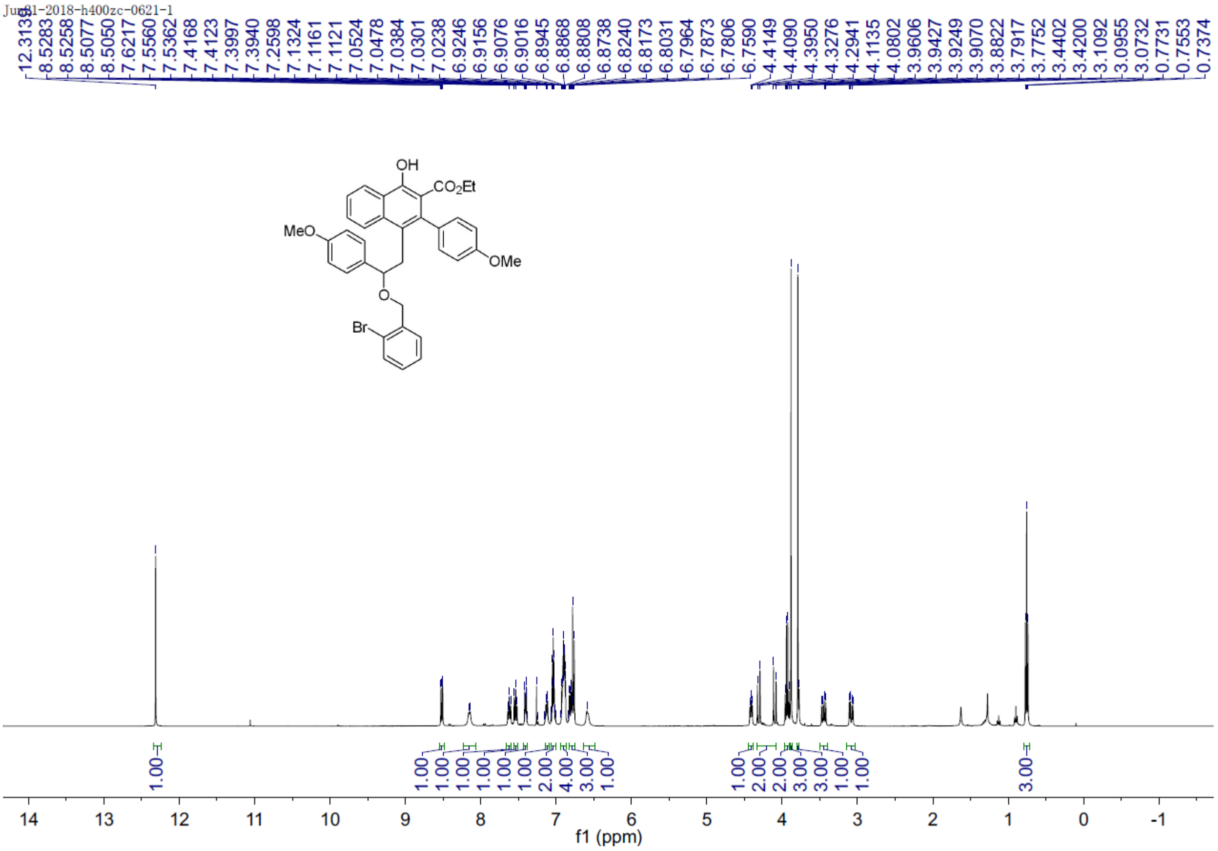

Supplementary Figure 135. ^{1}H NMR (400 MHz, $CDCl_3$) spectrum for compound 59.

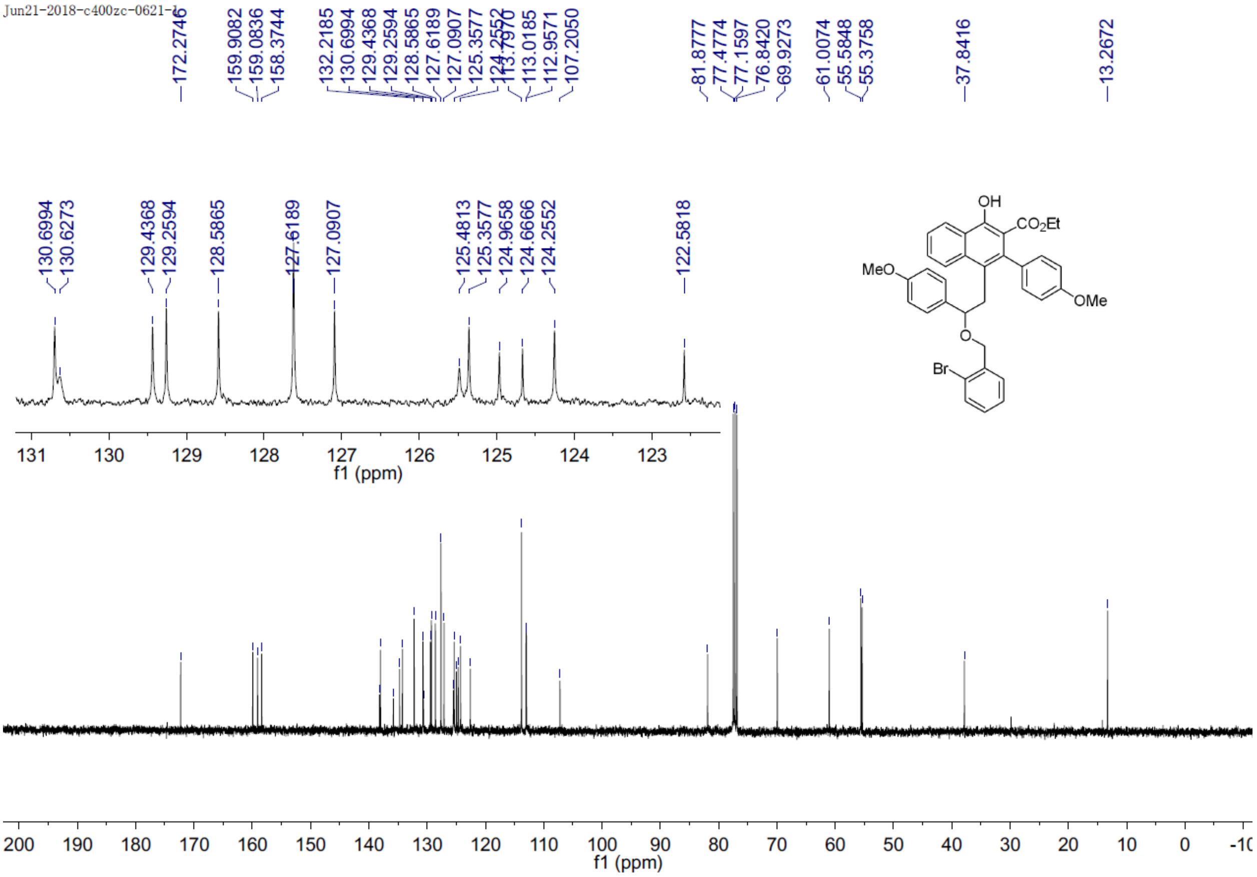

Supplementary Figure 136. ^{13}C NMR (100 MHz, $CDCl_3$) spectrum for compound 59.

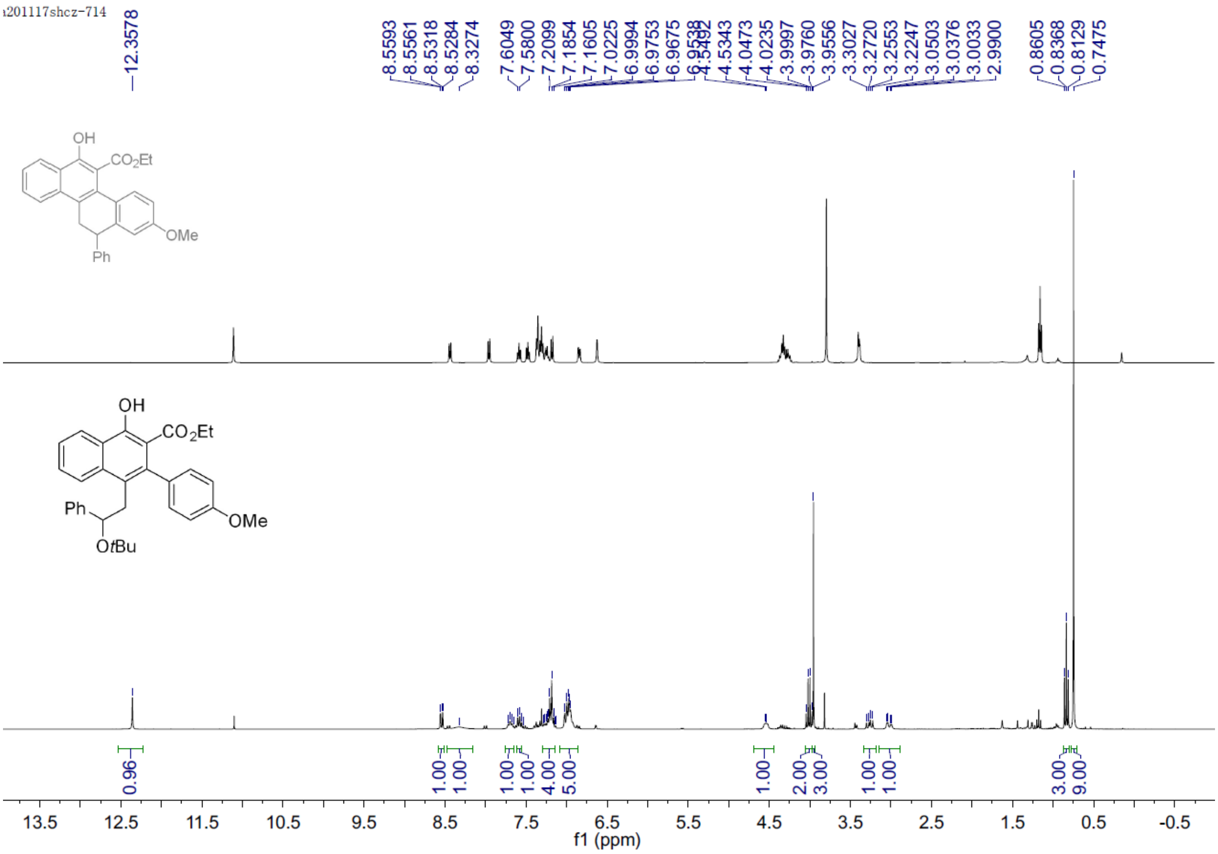

Supplementary Figure 137. ^{1}H NMR (300 MHz, $CDCl_3$) spectrum for compound 60.

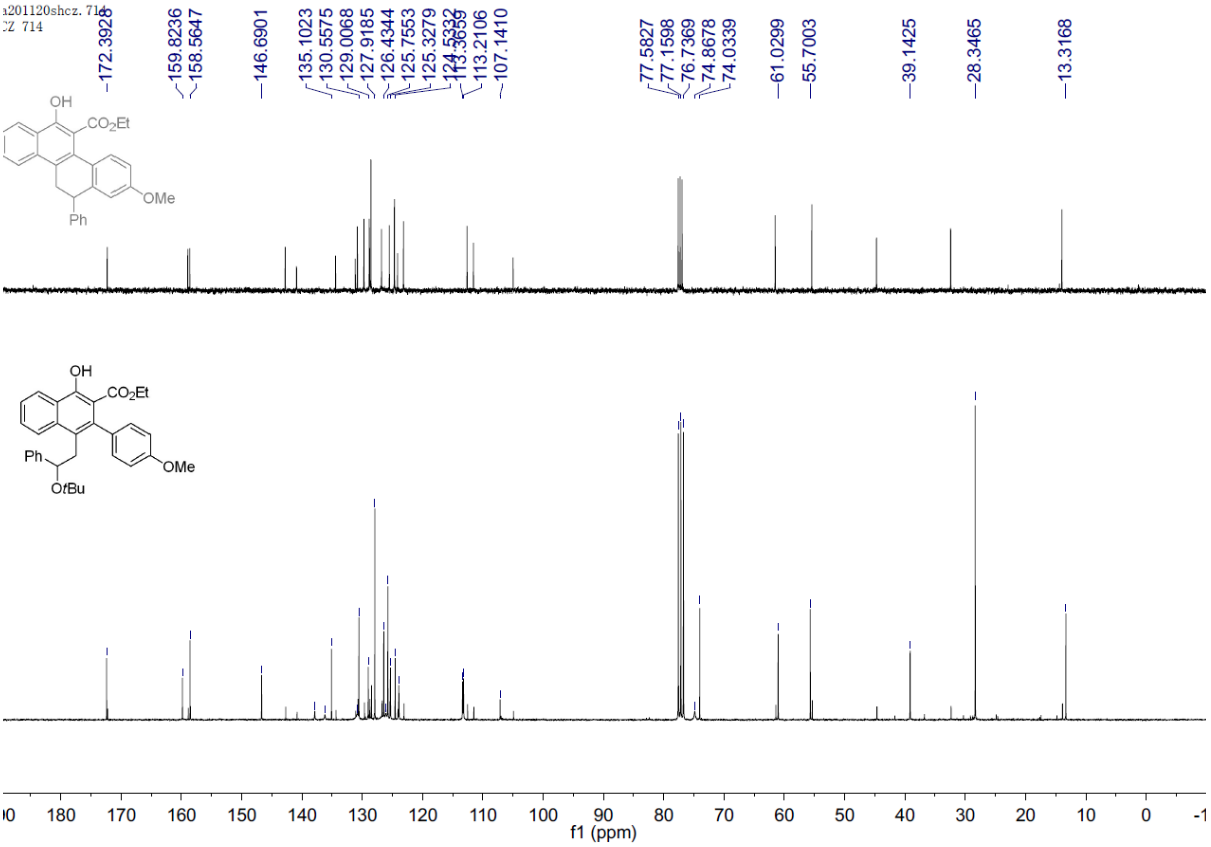

Supplementary Figure 138. ^{13}C NMR (75 MHz, $CDCl_3$) spectrum for compound 60.

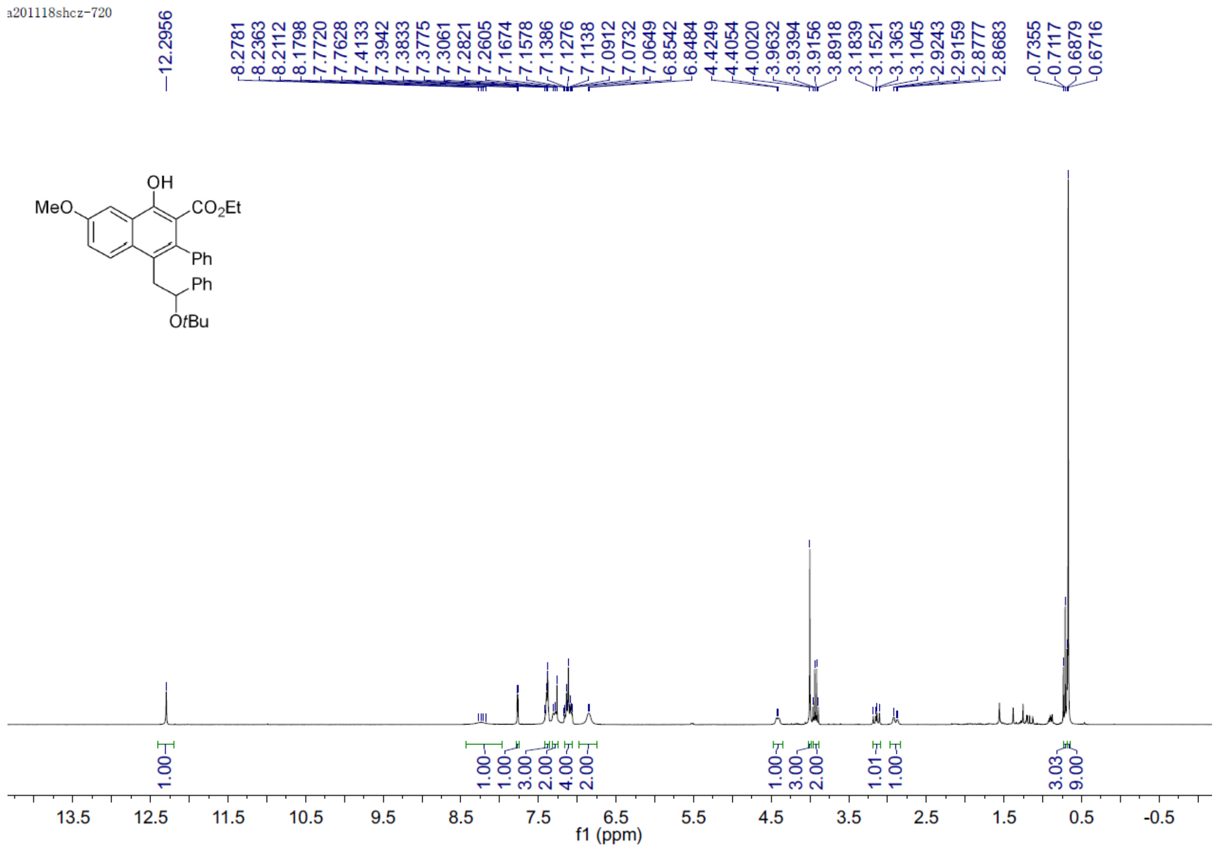

Supplementary Figure 139. ^{1}H NMR (300 MHz, $CDCl_3$) spectrum for compound 61.

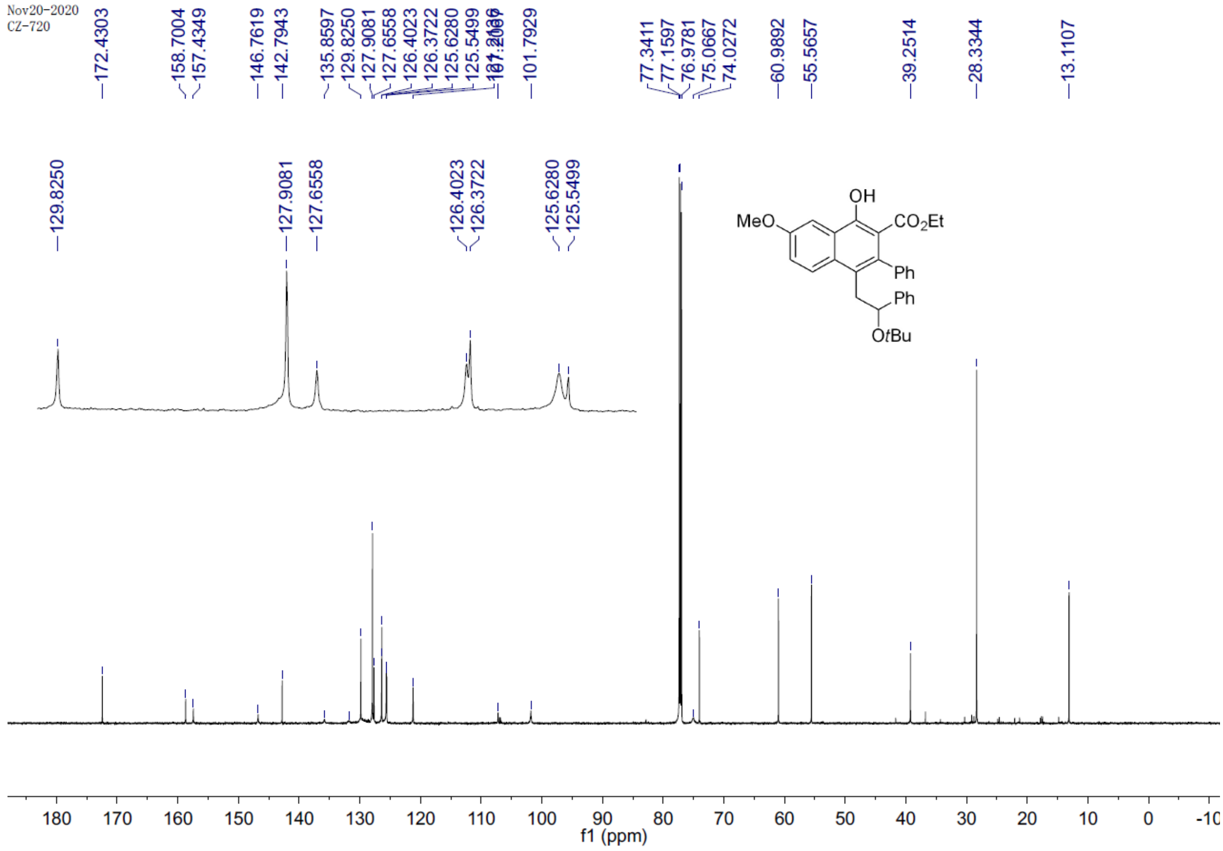

Supplementary Figure 140. ^{13}C NMR (175 MHz, $CDCl_3$) spectrum for compound 61.

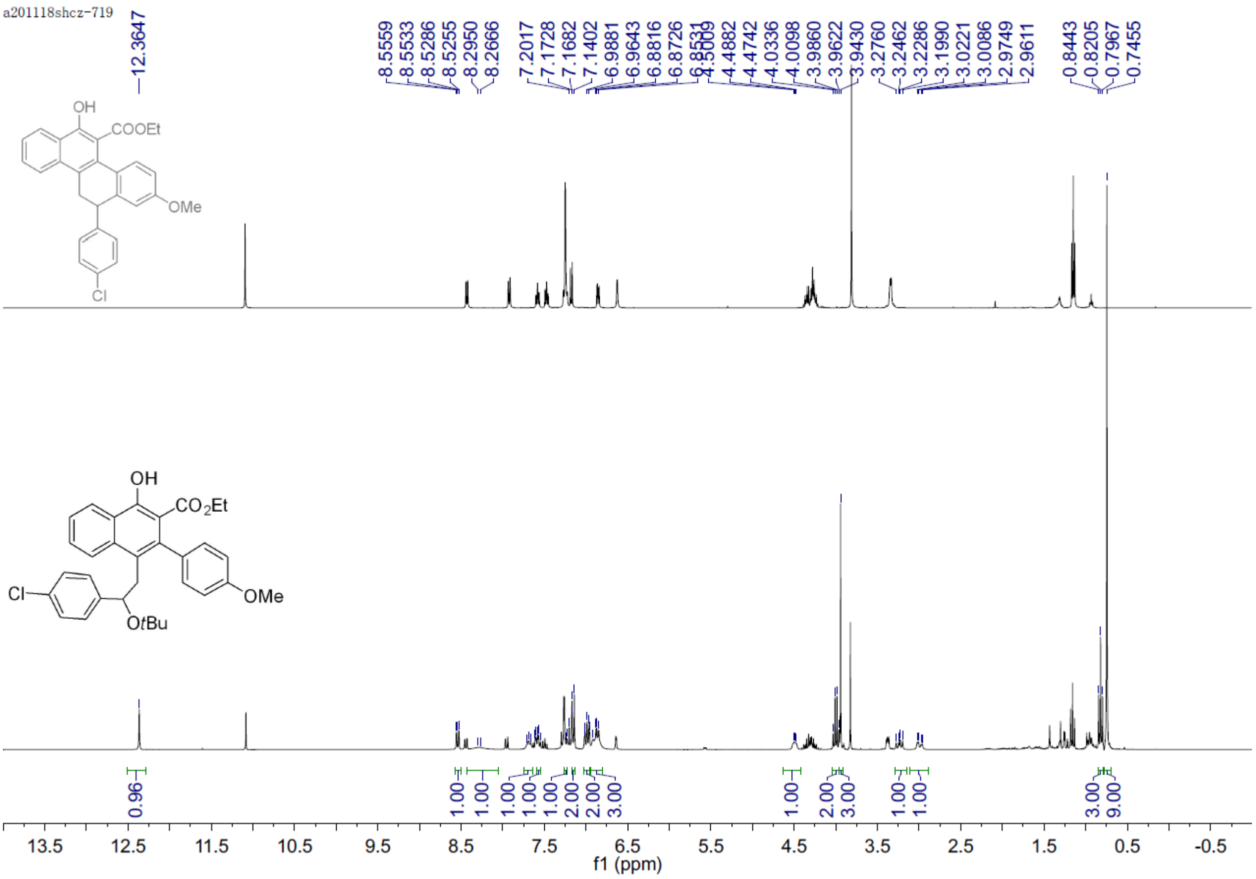

Supplementary Figure 141. ^{1}**H NMR (300 MHz, CDCl**$_3$**) spectrum for compound 62.**

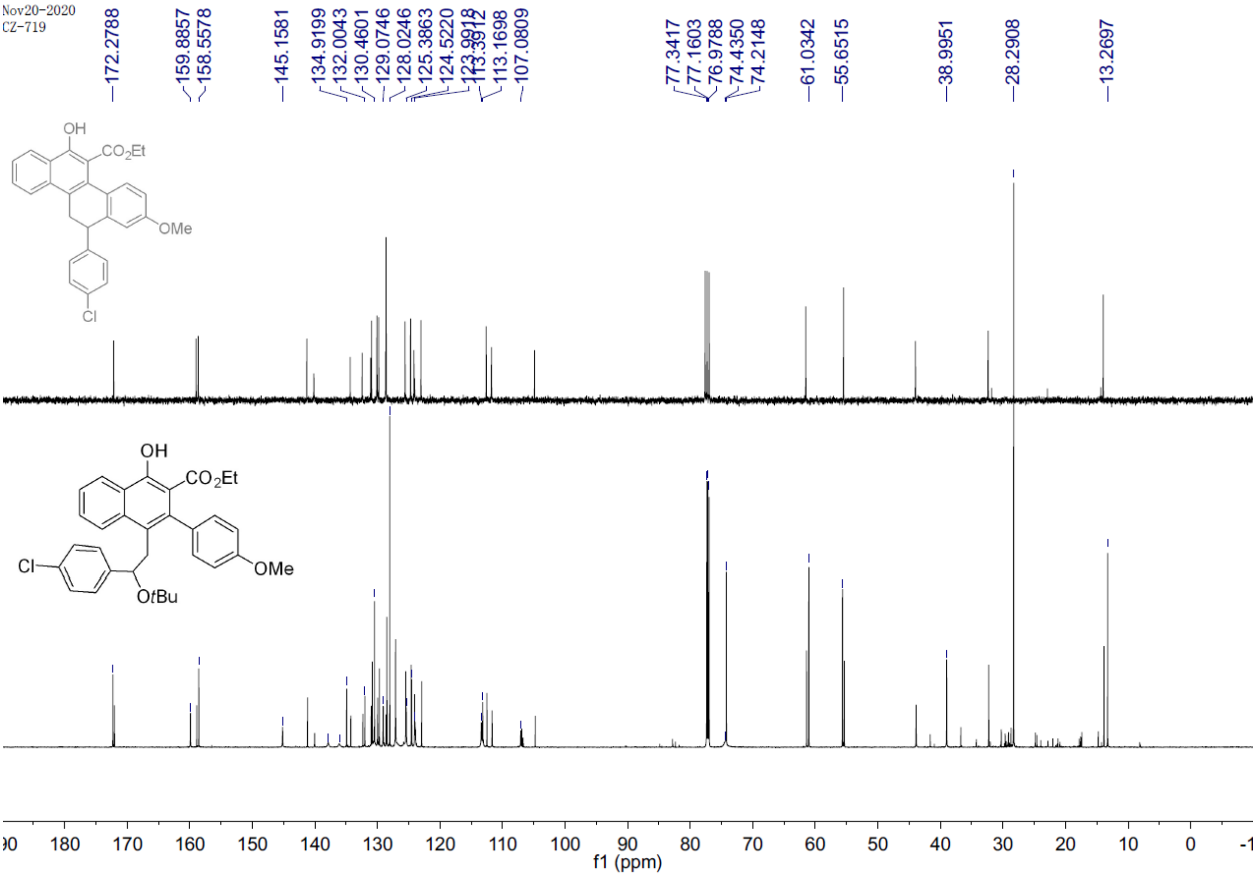

Supplementary Figure 142. ^{13}**C NMR (175 MHz, CDCl**$_3$**) spectrum for compound 62.**

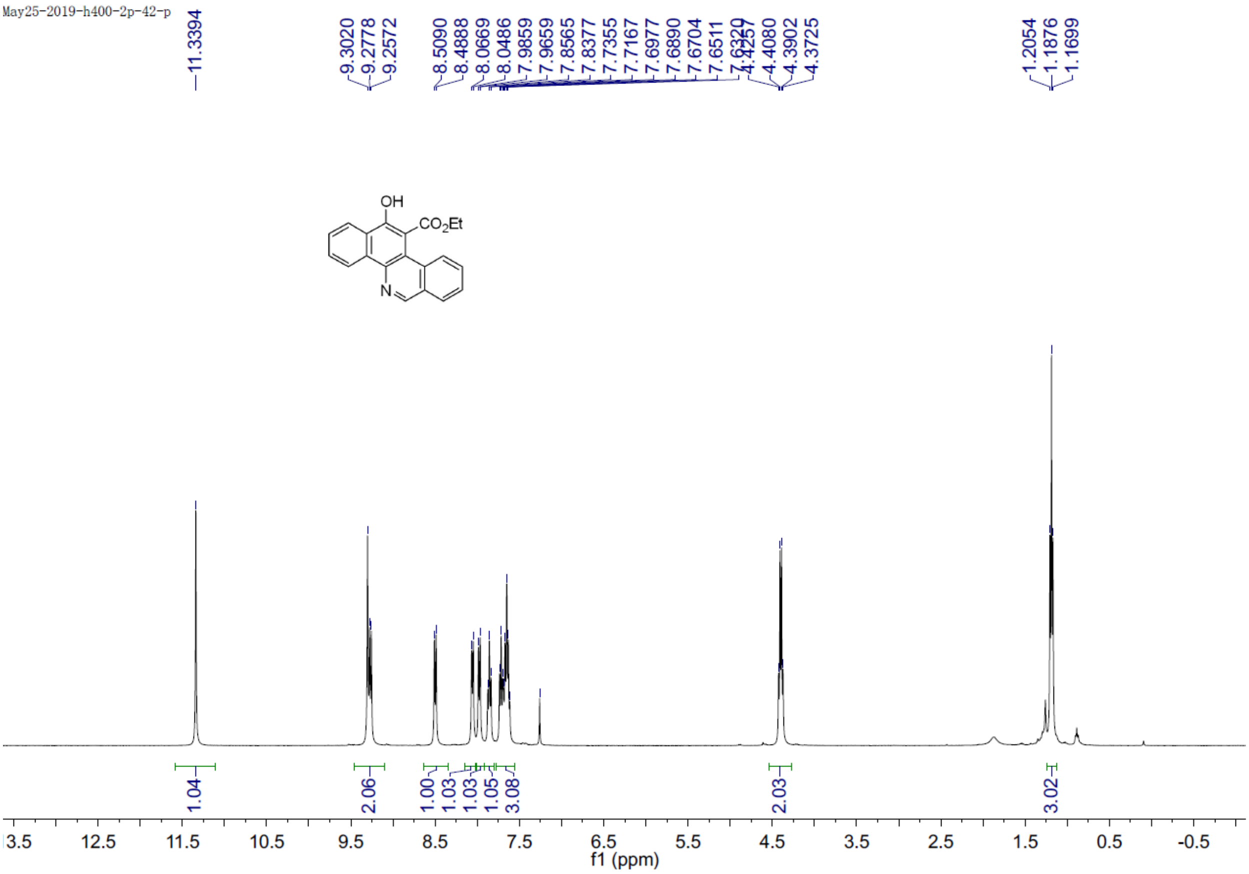

Supplementary Figure 143. ^{1}H NMR (400 MHz, $CDCl_3$) spectrum for compound 63.

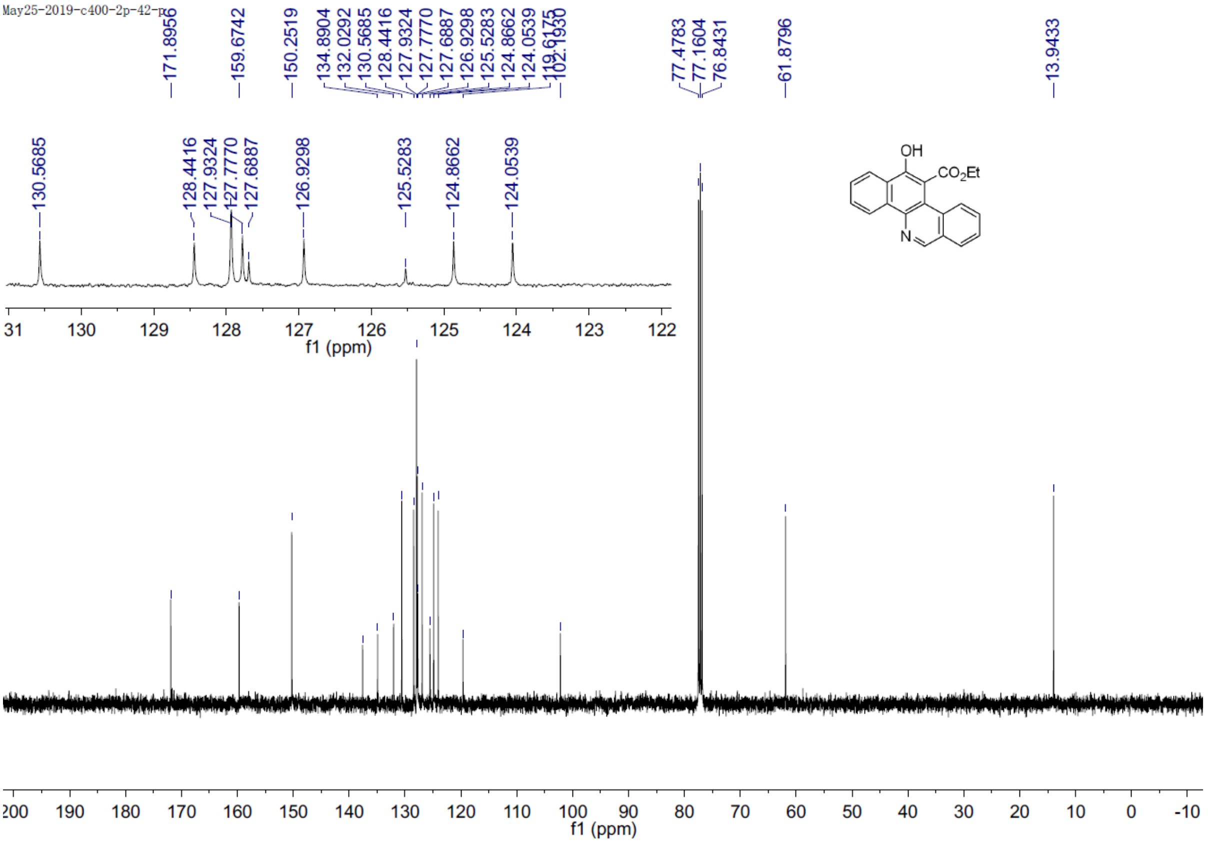

Supplementary Figure 144. ^{13}C NMR (100 MHz, $CDCl_3$) spectrum for compound 63.

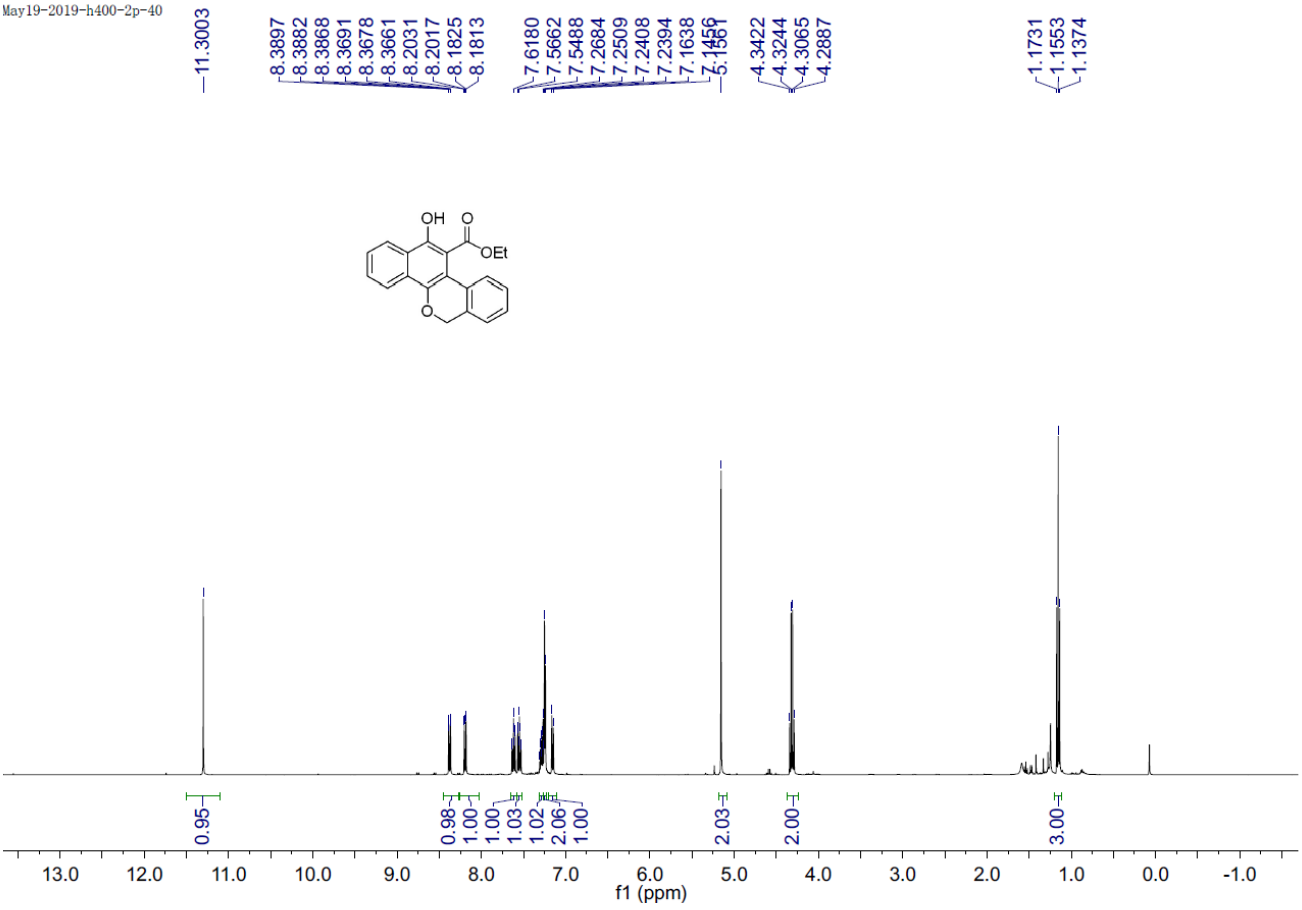

Supplementary Figure 145. ^{1}H NMR (400 MHz, $CDCl_3$) spectrum for compound 64.

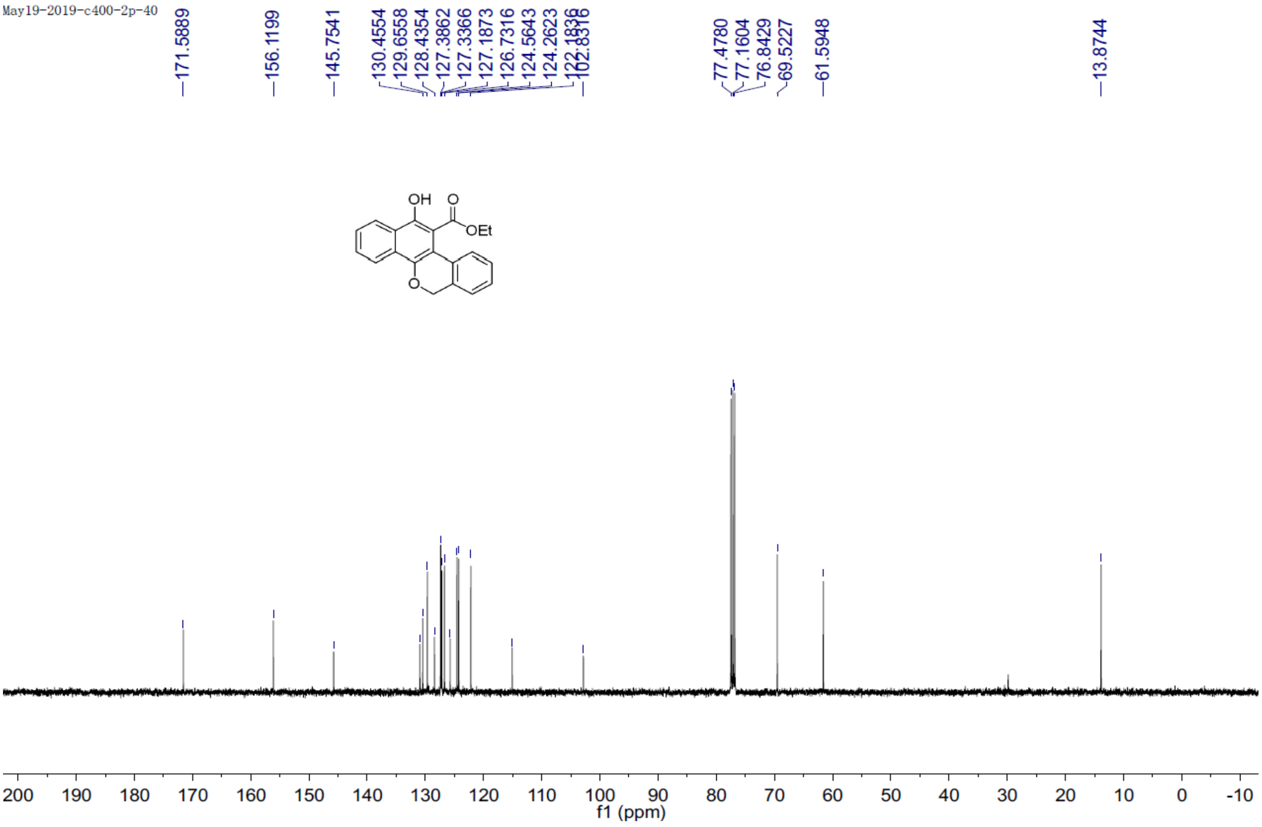

Supplementary Figure 146. ^{13}C NMR (100 MHz, $CDCl_3$) spectrum for compound 64.

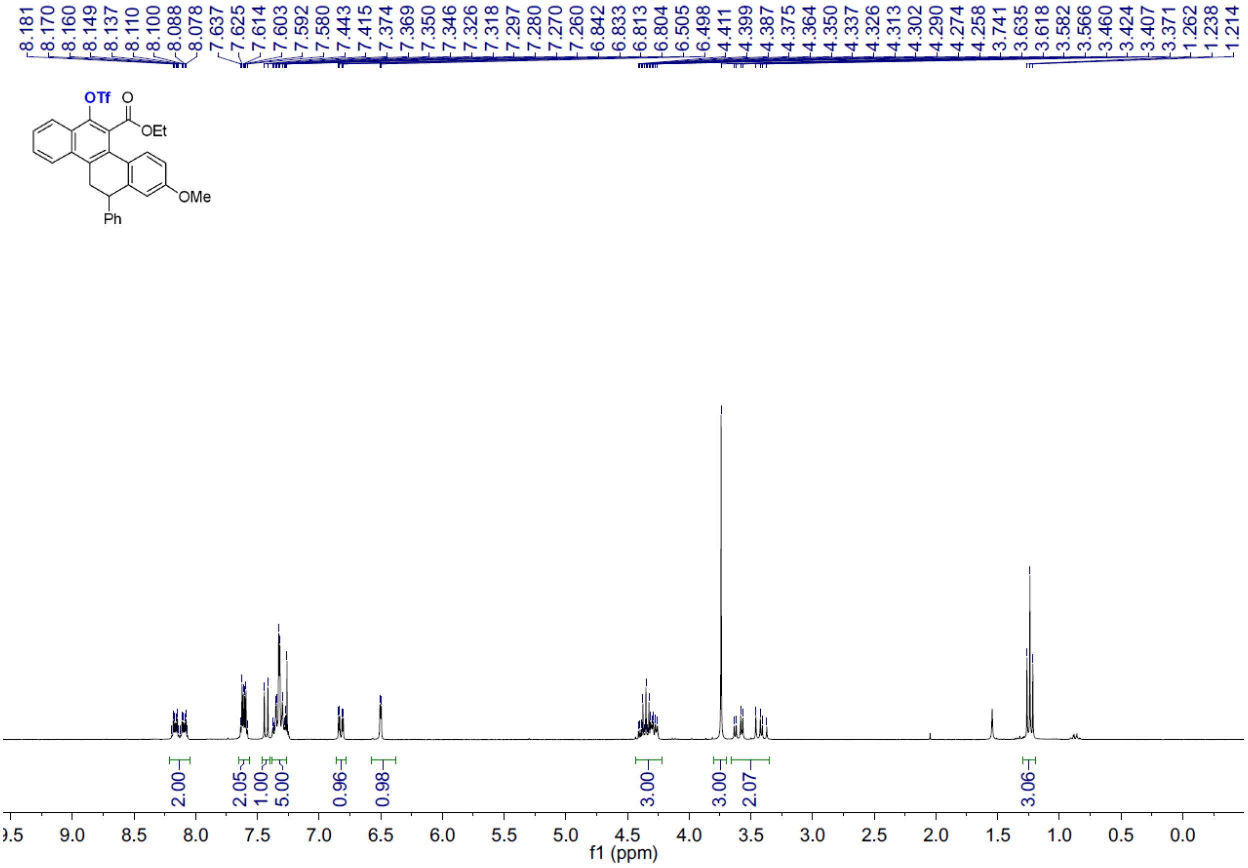

Supplementary Figure 147. ^{1}H NMR (300 MHz, $CDCl_3$) spectrum for compound 65.

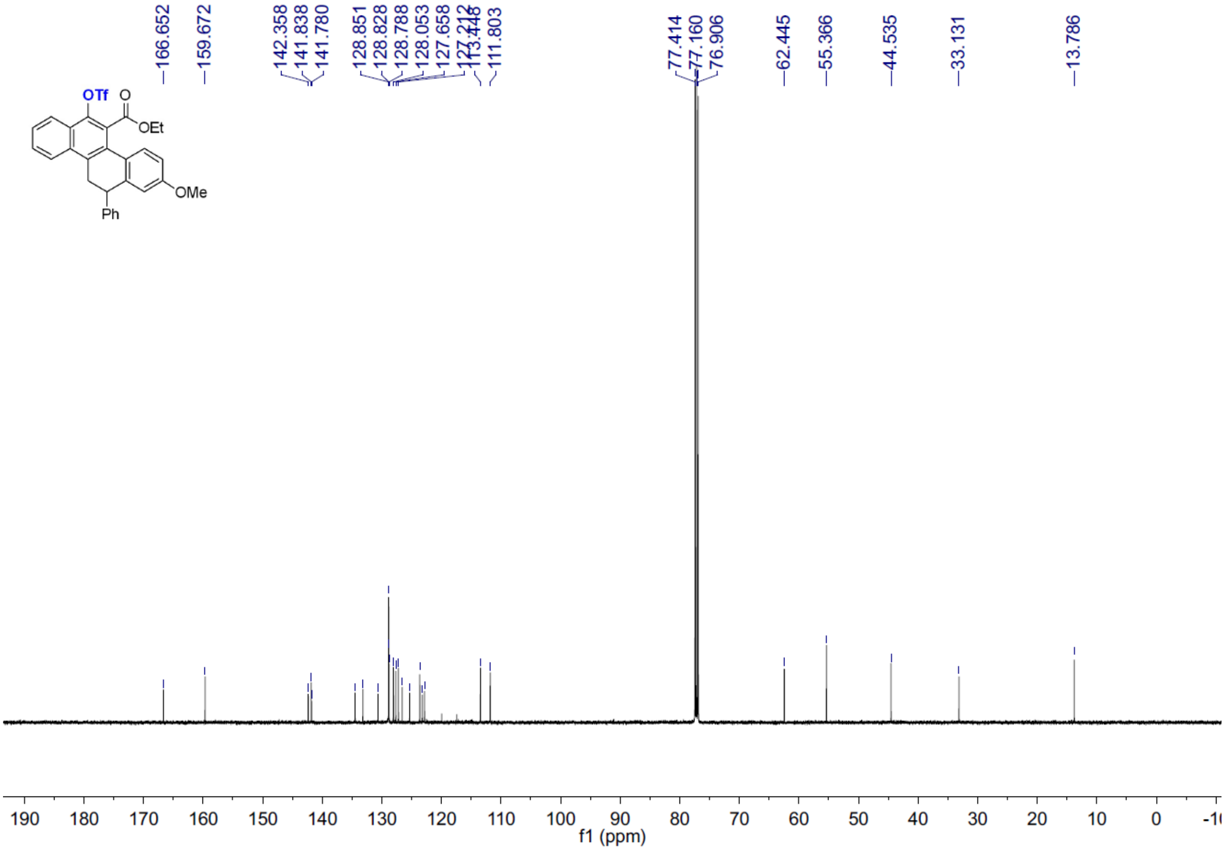

Supplementary Figure 148. ^{13}C NMR (125 MHz, $CDCl_3$) spectrum for compound 65.

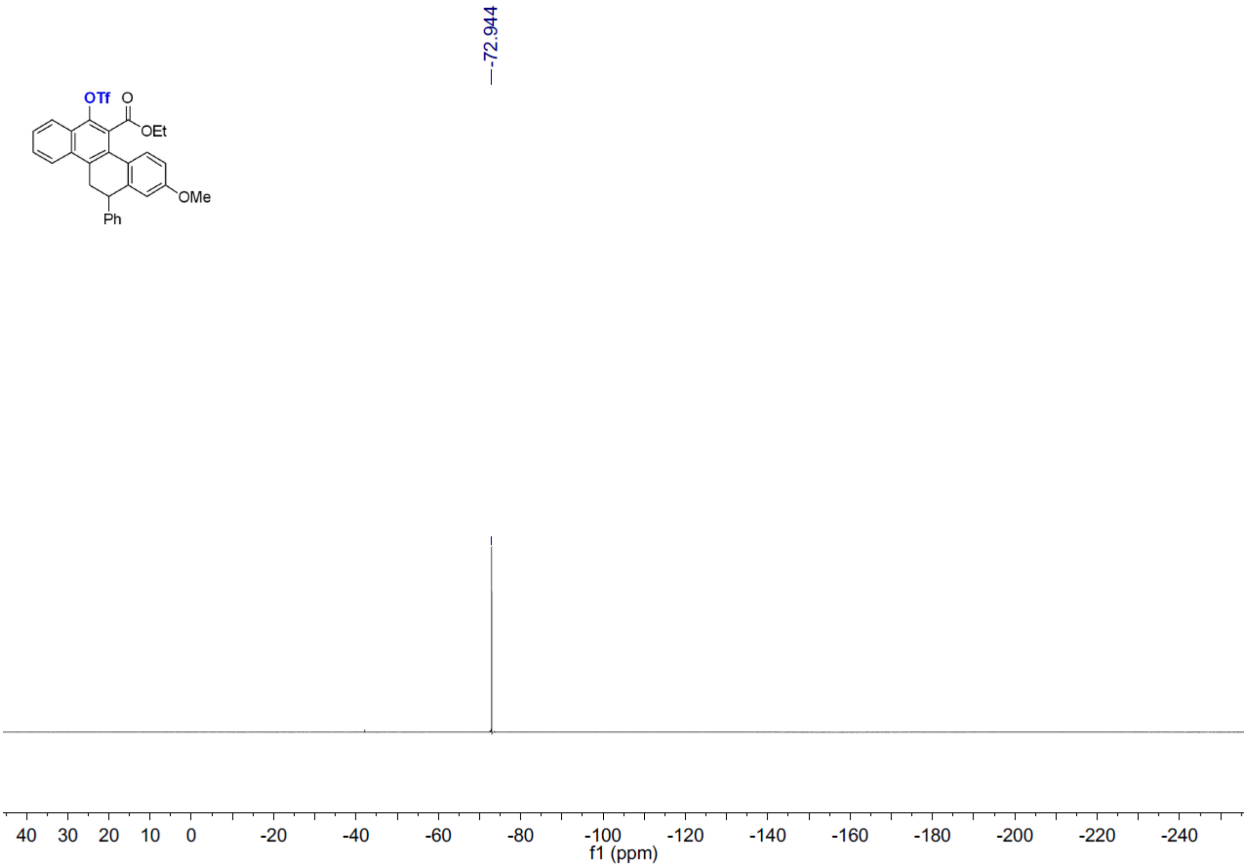

Supplementary Figure 149. ^{19}F NMR (283 MHz, $CDCl_3$) spectrum for compound 65.

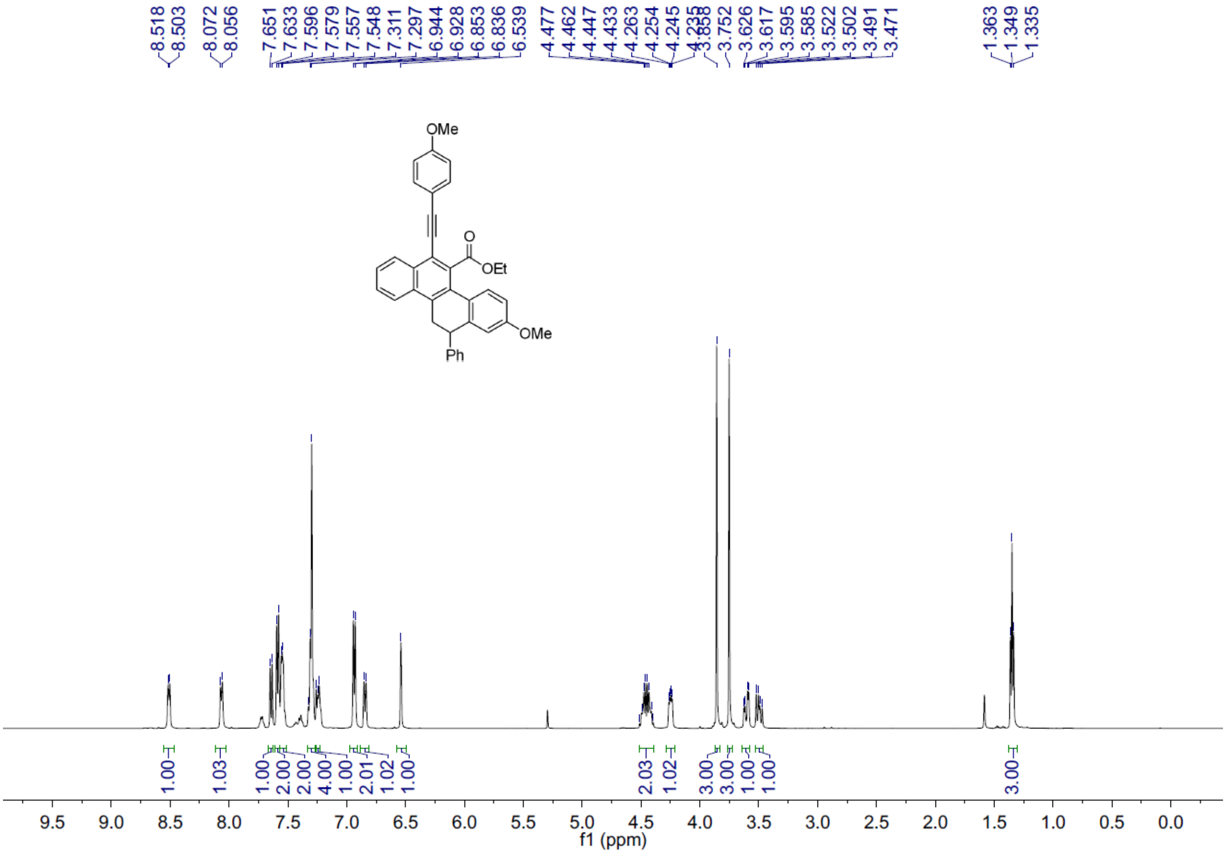

Supplementary Figure 150. ^{1}H NMR (500 MHz, $CDCl_3$) spectrum for compound 66.

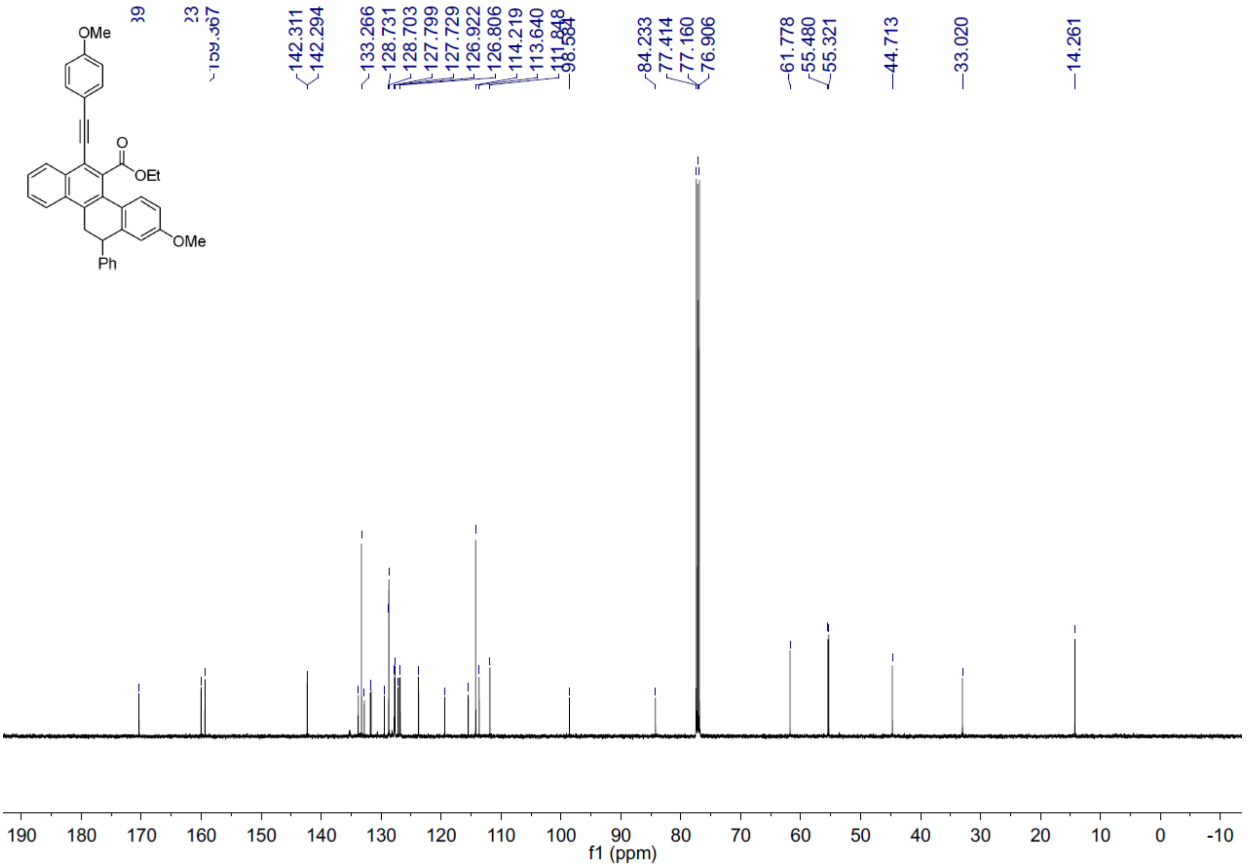

Supplementary Figure 151. ^{13}**C NMR (125 MHz, CDCl$_3$) spectrum for compound 66.**

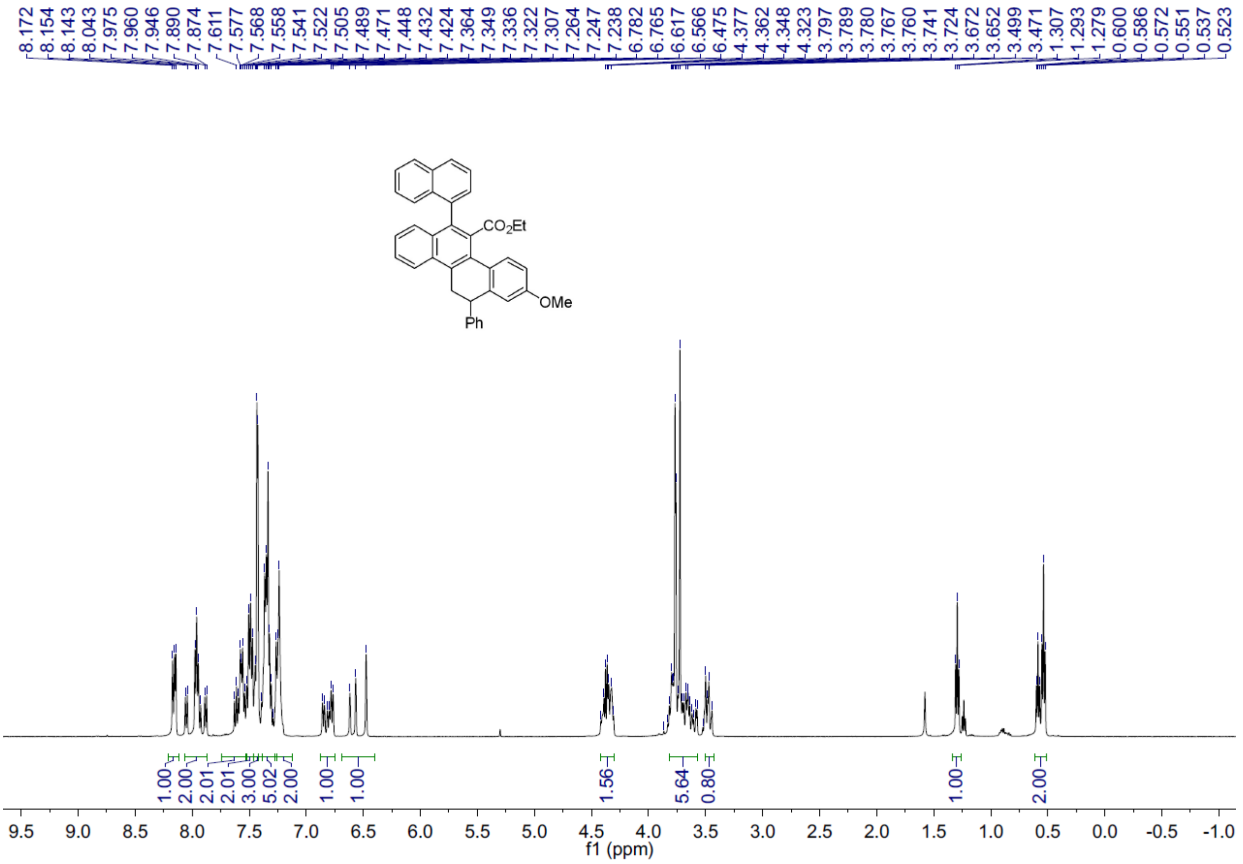

Supplementary Figure 152. ^{1}**H NMR (500 MHz, CDCl$_3$) spectrum for compound 67.**

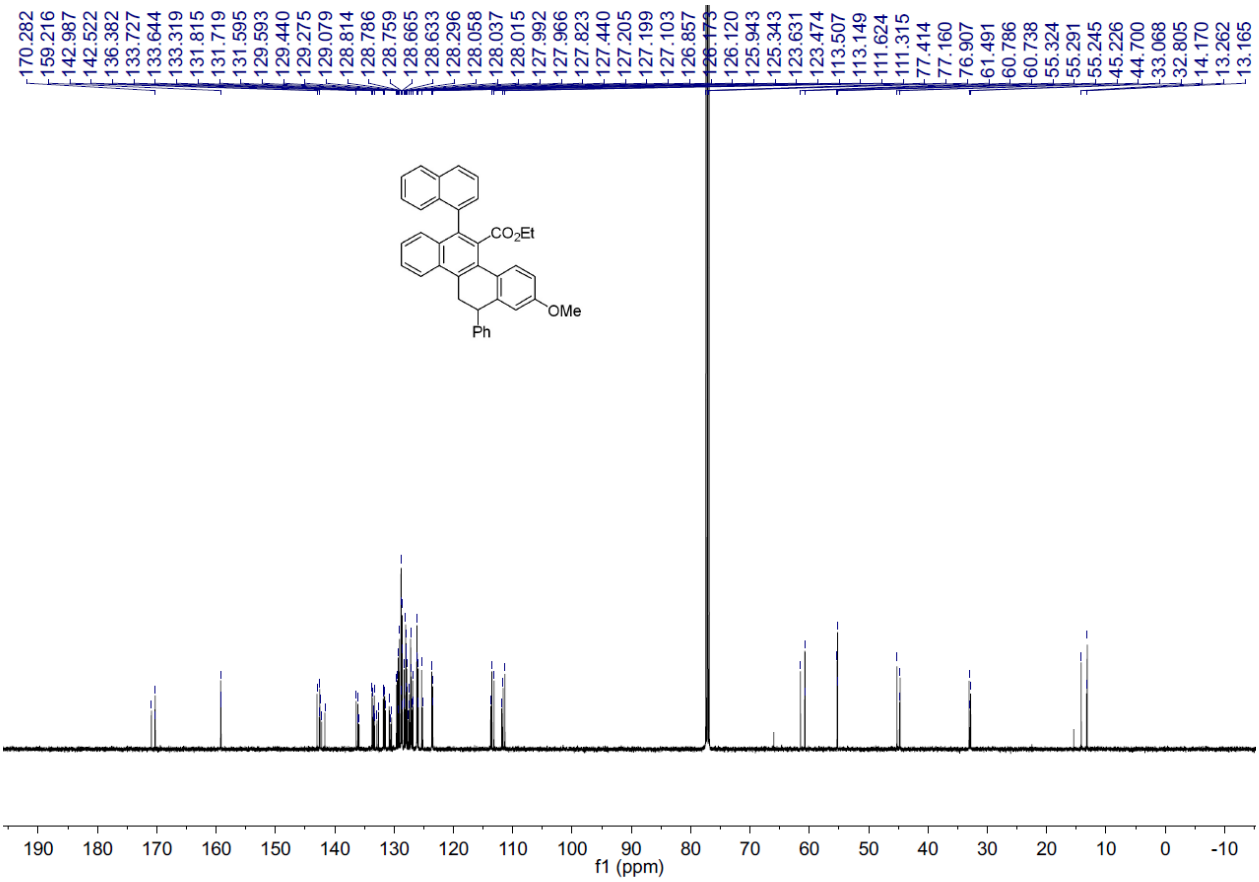

Supplementary Figure 153. ^{13}C NMR (125 MHz, CDCl$_3$) spectrum for compound 67.

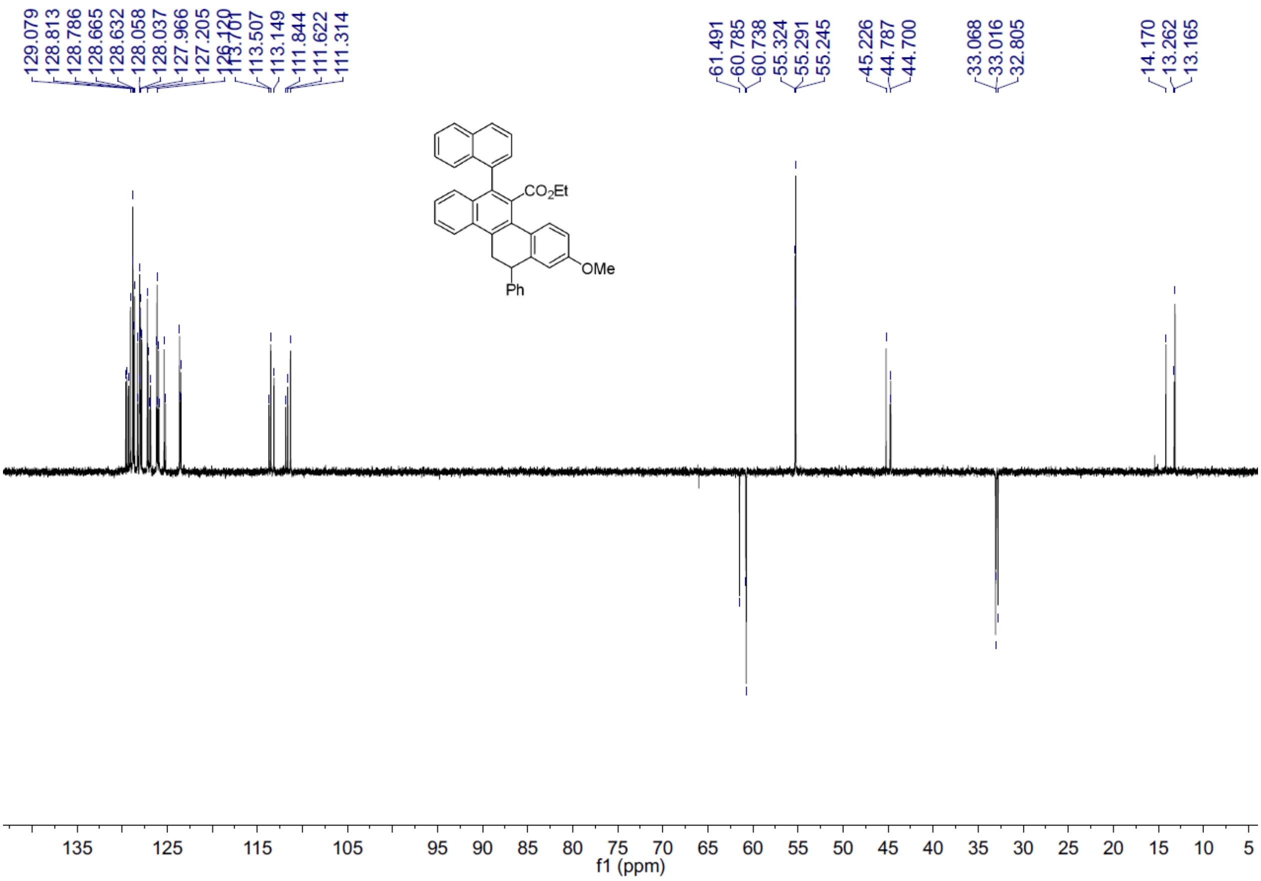

Supplementary Figure 154. Dept 135 NMR (125 MHz, CDCl$_3$) spectrum for compound 67.

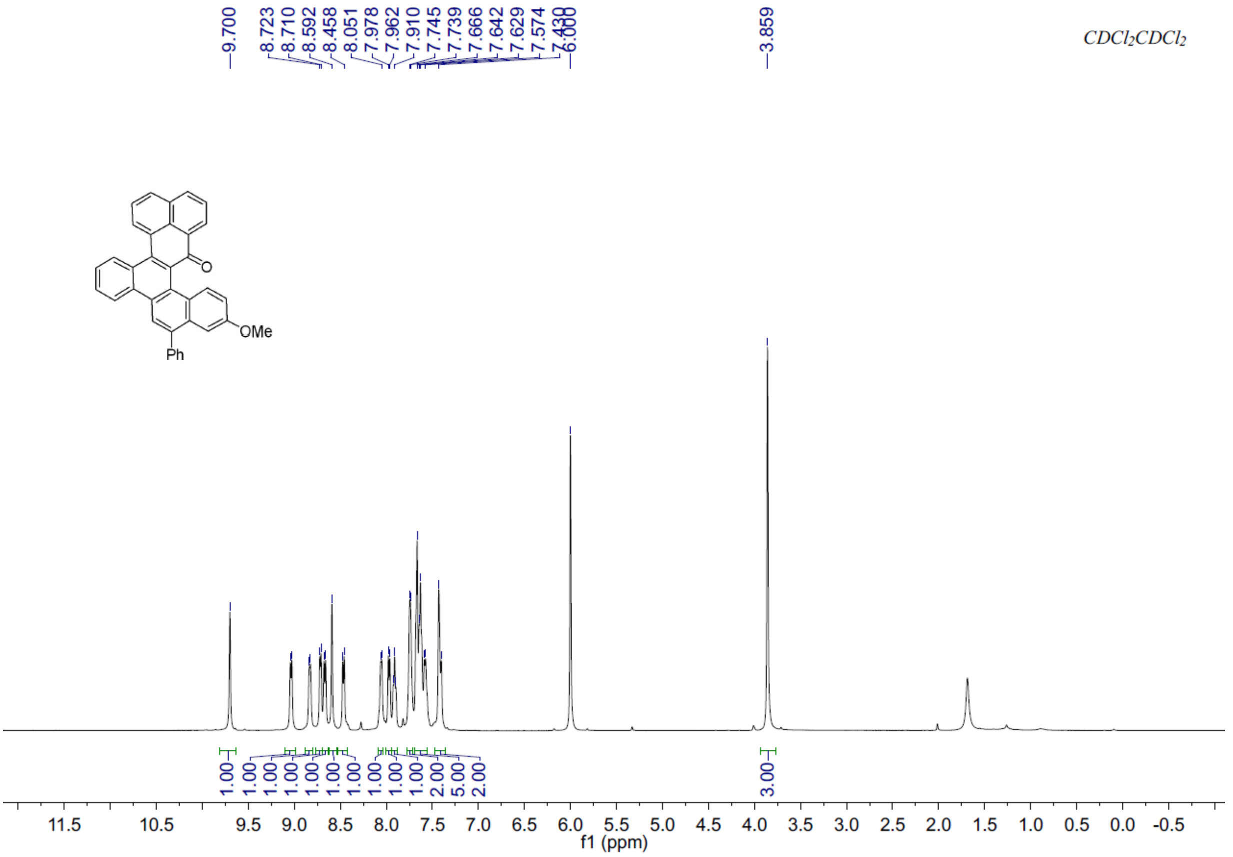

Supplementary Figure 155. ^{1}H NMR (500 MHz, $CDCl_2CDCl_2$) spectrum for compound 68.

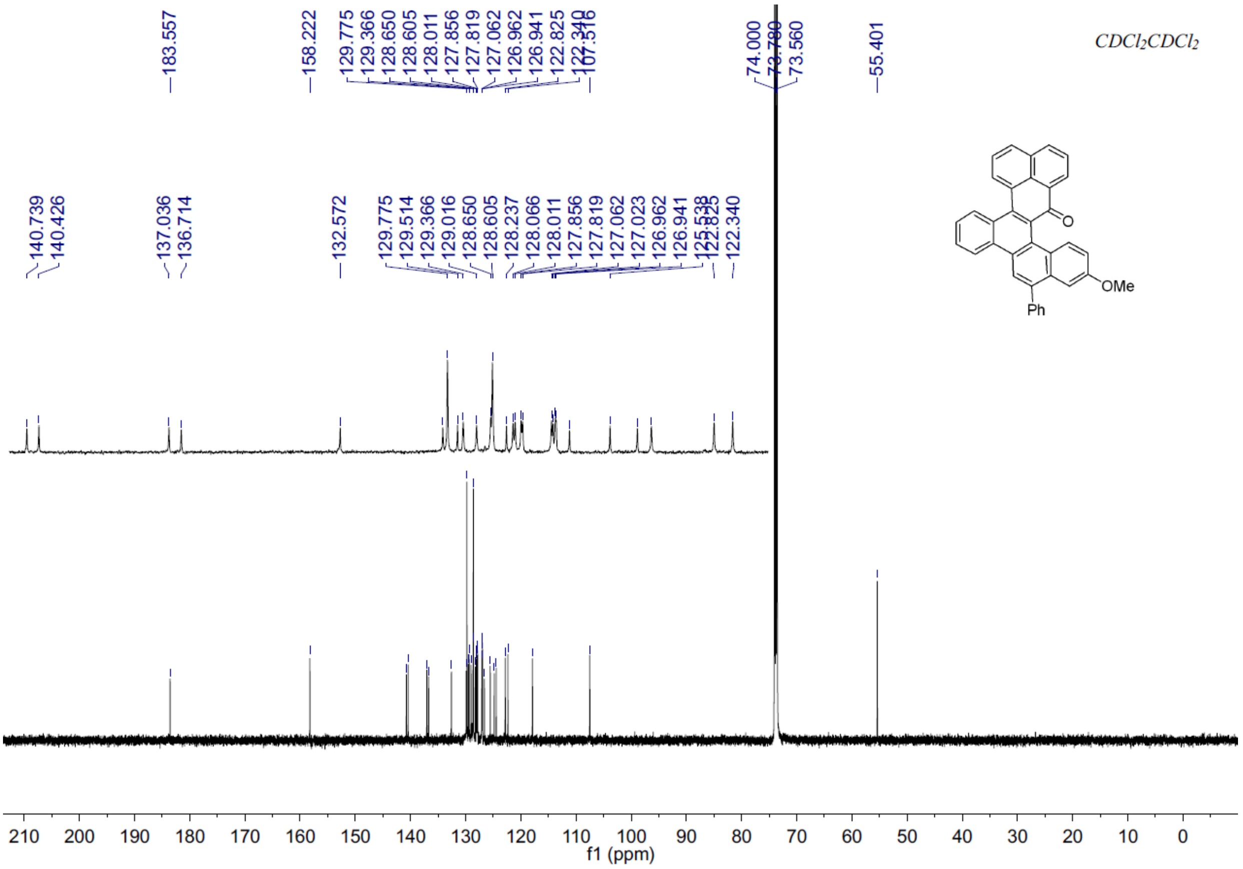

Supplementary Figure 156. ^{13}C NMR (125 MHz, $CDCl_2CDCl_2$) spectrum for compound 68.

HPLC Analysis of Racemic and Chiral 3.

Condition: hexane : 2-propanol=95:5

Flow rate = 1.0 mL/min, λ = 254 nm, Daicel Chiralpak IA

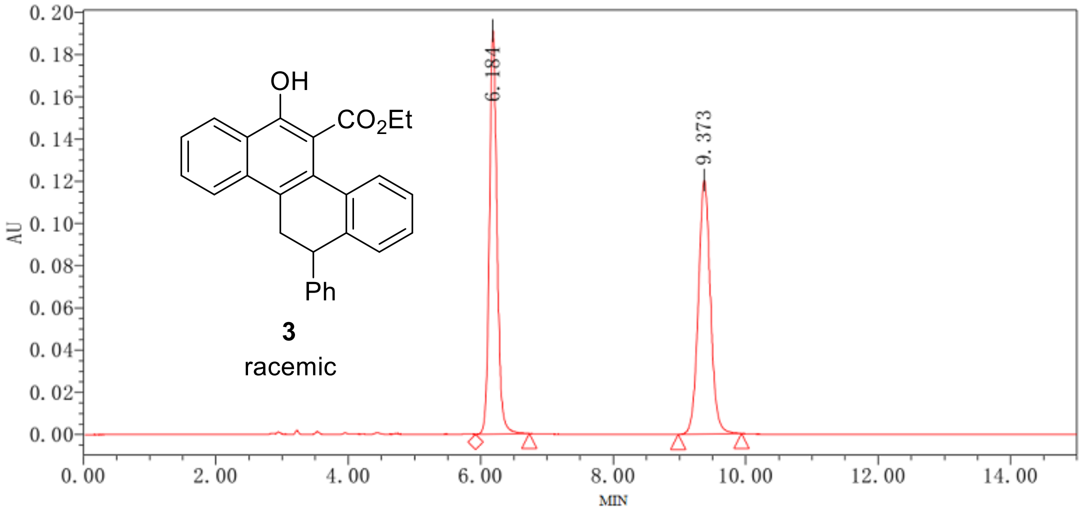

PDA Ch1 254 nm				
Peak#	Ret. Time	Area	Height	Area%
1	6.184	1533189	191241	50.063
2	9.373	1529318	120227	49.937
Total		3062507	311468	100

Supplementary Figure 157. Ethyl 6-hydroxy-12-phenyl-11,12-dihydrochrysene-5-carboxylate (*rac*-3)

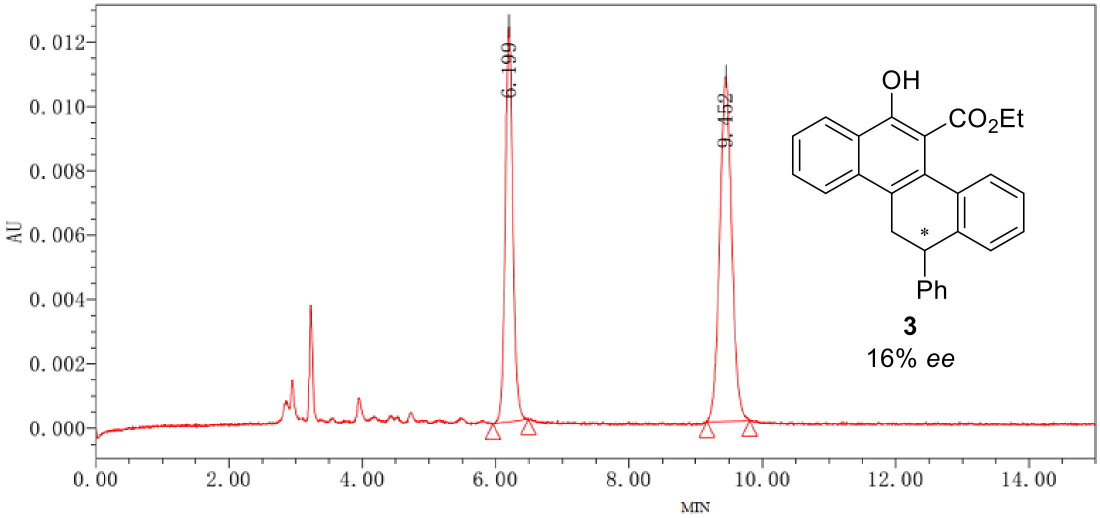

PDA Ch1 254 nm				
Peak#	Ret. Time	Area	Height	Area%
1	6.199	98455	12317	42.128
2	9.452	135247	10728	57.872
Total		233702	23045	100

Supplementary Figure 158. Ethyl 6-hydroxy-12-phenyl-11,12-dihydrochrysene-5-carboxylate (16% ee of 3)

Crystallographic Data for 23

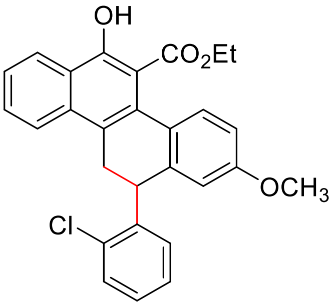

23, *CCDC*: 1828268

≡

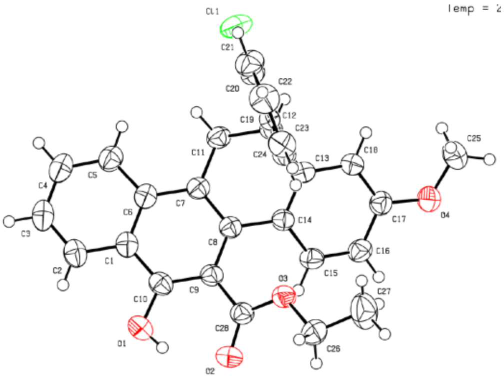

Datablock: cu_zc0207a_1_0m

Bond precision: C-C = 0.0022 A Wavelength=1.54178

Cell:	a=14.510(2)	b=7.7110(11)	c=19.858(3)
	alpha=90	beta=91.260(6)	gamma=90
Temperature:	290 K		

	Calculated	Reported
Volume	2221.3(6)	2221.4(6)
Space group	P 21/c	P 1 21/c 1
Hall group	-P 2ybc	-P 2ybc
Moiety formula	C28 H23 Cl O4	C28 H23 Cl O4
Sum formula	C28 H23 Cl O4	C28 H23 Cl O4
Mr	458.91	458.91
Dx,g cm-3	1.372	1.372
Z	4	4
Mu (mm-1)	1.799	1.799
F000	960.0	960.0
F000'	964.18	
h,k,lmax	17,9,24	17,9,23
Nref	4131	4042
Tmin,Tmax	0.682,0.698	0.530,0.753
Tmin'	0.464	

Correction method= # Reported T Limits: Tmin=0.530 Tmax=0.753
AbsCorr = MULTI-SCAN

Data completeness= 0.978 Theta(max)= 68.992

R(reflections)= 0.0385(3699) wR2(reflections)= 0.1129(4042)

S = 1.063 Npar= 301

Supplementary Figure 159. Crystallographic Data for 23.

Crystallographic Data for 34

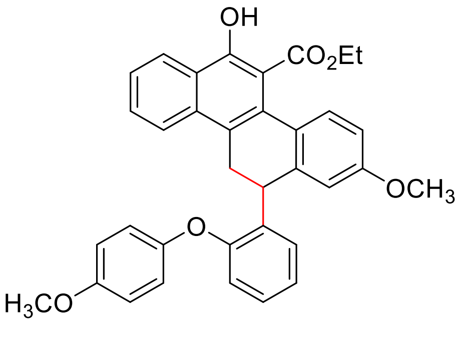

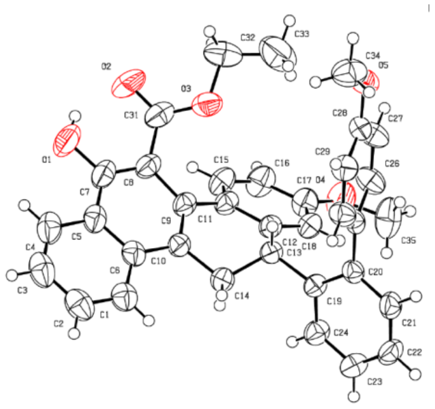

34, *CCDC*: 1849634

Datablock: t

Bond precision: C-C = 0.0041 A Wavelength=0.71073

Cell:	a=9.29800	b=19.00800	c=15.95600
	alpha=90	beta=96.7100	gamma=90
Temperature:	293 K		

	Calculated	Reported
Volume	2800.689	2801
Space group	P 21/n	P 21/n
Hall group	-P 2yn	-P 2yn
Moiety formula	C35 H30 O5	?
Sum formula	C35 H30 O5	C35 H30 O5
Mr	530.59	530.59
Dx,g cm-3	1.258	1.258
Z	4	4
Mu (mm-1)	0.083	0.083
F000	1120.0	1120.0
F000'	1120.54	
h,k,lmax	11,22,18	11,22,18
Nref	4930	4904
Tmin,Tmax		
Tmin'		

Correction method= Not given

Data completeness= 0.995 Theta(max)= 24.997

R(reflections)= 0.0751(4120) wR2(reflections)= 0.1561(4904)

S = 1.147 Npar= 365

Supplementary Figure 160. Crystallographic Data for 34.

Supplementary References

1. Wang, J. et al. Facile Synthesis of Halogenated Spiroketals via a Tandem Iodocyclization. *Org. Lett.* **16**, 2236–2239 (2014).
2. Zhang, C. et al. Gold(I)-Catalyzed Aromatization: Expeditious Synthesis of Polyfunctionalized Naphthalenes. *iScience* **21**, 499–508 (2019).
3. Bauer, A. et al. Tris(dimethylamino)phosphane as a New Ligand in Gold(I) Chemistry: Synthesis and Crystal Structures of $[(Me_2N)_3P]AuCl$, $\{[(Me_2N)_3PAu]_3O\}^+BF_4^-$, $\{[Me_2N)_3PAu]_3NP(NMe_2)_3\}^{2+}\{BF_4^-\}_2$ and the Precursor Molecule $(Me_2N)_3PNSiMe_3$. *Chem. Ber.* **130**, 323−328 (1997).
4. Jover, J. et al. Expansion of the Ligand Knowledge Base for Monodentate P-Donor Ligands (LKB-P). *Organometallics* **29**, 6245−6258 (2010).
